# Subporphyrazine scaffolds as emerging electron acceptors for long-lived charge separation

**DOI:** 10.1039/d5sc08213g

**Published:** 2026-01-19

**Authors:** Swathi Krishna, Elena Cañizares-Espada, David Guzmán, Yifan Bo, Timothy Clark, Tomás Torres, Dirk M. Guldi, M. Salomé Rodríguez-Morgade

**Affiliations:** a Department of Chemistry and Pharmacy, Profile Center FAU Solar, Interdisciplinary Center for Molecular Materials (ICMM), Friedrich-Alexander-Universität Erlangen-Nürnberg Egerlandstr. 3 Erlangen 91058 Germany dirk.guldi@fau.de; b Departamento de Química Orgánica, Universidad Autónoma de Madrid Cantoblanco Madrid 28049 Spain salome.rodriguez@uam.es tomas.torres@uam.es; c Department of Chemistry and Pharmacy, Computer-Chemie-Center (CCC) Friedrich-Alexander-Universität Erlangen-Nürnberg Nägelbachstr. 25 Erlangen 91052 Germany; d Institute for Advanced Research in Chemical Sciences (IAdChem), Universidad Autónoma de Madrid Cantoblanco 28049 Madrid Spain; e Instituto Madrileño de Estudios Avanzados (IMDEA)-Nanociencia C/Faraday 9, Cantoblanco 28049 Madrid Spain

## Abstract

Using subporphyrazines (SubPzs) as electron acceptors in the modular design, *via* metal–ligand axial coordination with ruthenium(ii) phthalocyanine (Ru(CO)Pc), was key to dictating the different photophysical evolution in four electron donor–acceptor Ru(CO)Pc-SubPz conjugates (1–4). Complementary absorptions of SubPzs and Ru(CO)Pc allowed a nearly panchromatic absorption across the visible range. The oxidizing ability of the hexasubstituted SubPz acceptors in 1–4 was tuned through their peripheral functionalization with propyl and sulfanyl groups, as well as with strong electron-accepting (*E*)-acrylate- and (*E*)-4-nitrostyryl groups, respectively. *Intra*molecular Förster Resonance Energy Transfer (*i*-FRET) and *intra*molecular charge separation (*i*-CS) upon SubPz excitation were corroborated by means of absorption, fluorescence, and electrochemical measurements. Excited-state deactivations were established using time-resolved pump-probe transient absorption spectroscopy. In 1, photoexcitation is followed by a rapid *i*-FRET and Ru(CO)Pc singlet excited state formation, which decays *via* its triplet state. For 2, excitation triggers *i*-FRET and *i*-CS and generates a singlet charge-separated state, decaying through Ru(CO)Pc triplet state. Excitation in 3 sparks *i-*FRET and *i-*CS to afford a triplet charge-separated state, where the spin evolution was confirmed by magnetic-field-dependent studies. Lastly, for 4, *i*-CS outcompetes *i*-FRET, forming a singlet charge-separated state that spin flips to a long-lived triplet charge-separated state featuring lifetimes of several microseconds.

## Abstract 2

Using subporphyrazines (SubPzs) as tunable electron acceptors, four donor–acceptor conjugates were prepared. The nature of each SubPz governed excited-state deactivation, yielding either singlet, or long-lived triplet charge-separated states.

## Graphical abstract

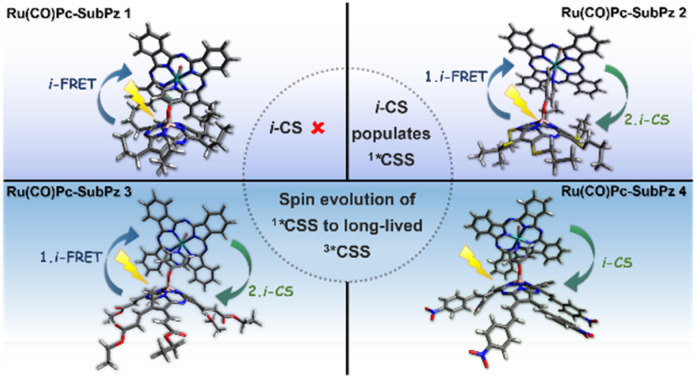

## Introduction

The quest for sustainable energy sources has led to tremendous advances in the field of solar energy conversion research in recent decades. Drawing inspiration from nature's intricate processes, artificial photosynthesis and molecular photovoltaics stand at the forefront of sustainable energy research.^1–3^ Central to their functionality are two fundamental processes, namely energy and electron transfer. Both underscore the significance of precisely tuning molecular assemblies to facilitate efficient separation of charges and transport thereof.^4^ In this regard, a plethora of energy and electron donor–acceptor (D–A) systems have been designed to realize fast charge separation and slow charge recombination.^5,6^

Porphyrinoids stand out among the many building blocks that are frequently chosen as chromophores in solar energy conversion systems, in general,^7^ and in D–A ensembles, in particular.^8^ Among them, phthalocyanines (Pcs) are of particular interest owing to their strong absorptions across the visible range of the solar spectrum,^9,10^ their small reorganization energy in electron transfer reactions, and their rich redox chemistry.^11,12^ In the context of electron acceptors, fullerenes stand out for their outstanding redox chemistry, their small reorganization energy in electron transfer reactions, their favourable nanoscale morphology, and their ability to transport electrons.^13–17^ All of the aforementioned has stimulated their extensive use as components in D–A systems made for molecular photovoltaics^13^ and artificial photosynthesis.^18–31^

The design of electron/energy donor–acceptor systems built around metal–ligand coordination has been incentivized by a structural motif that allows full control over energy/electron transfer processes.^32^ For example, ruthenium(ii) phthalocyanines (RuPcs), which are known to afford stable assemblies based on strong axial coordination, combine rapid charge separation with slow charge recombination. We have used these peculiarities to incorporate RuPcs not only into different types of solar cells,^33^ but also into D–A systems using different electron acceptors^34–37^ and as photosensitizers in photodynamic therapy (PDT).^38–40^ In these examples, RuPcs are endowed with different complementary units using a modular synthetic approach, ultimately allowing to apply an iterative design and optimize the nature of the RuPc's counterparts.^34–38^

A growing interest in non-fullerene electron acceptors^14,41^ has fueled the interest in subporphyrinoids^42,43^ because they exhibit high excited state energies and low reorganization energies in electron transfer reactions stemming from their non-planar 14 π-electron aromatic core.^44^ In fact, subphthalocyanines (SubPcs) and subnaphthalocyanines (SubNcs) are being extensively probed as integrative components in electron D–A systems^45–47^ and photovoltaics.^42,43,48,49^ The basic structure of subporphyrinoids also provides the added advantage of allowing easy and robust functionalization at the axial position. This is of great value for either tuning their specific characteristics or building complex architectures.^50–53^ Our selection of subporphyrazines (SubPzs)^54–56^ as a non-fullerene electron acceptor is guided by properties that render them particularly advantageous:

First, subtriazaporphyrins (SubPzs and SubPcs) have been found to be better electron acceptors than subporphyrins (SubPs). This is reflected by higher reduction potentials, mostly between −0.8 and −1.7 V *vs.* Fc/Fc^+^, relative to the corresponding subporphyrins ((−1.2)–(−2.2 V) *vs.* Fc/Fc^+^).^42,43^ As a matter of fact, SubPs have been used as the electron donors in dye sensitized solar cells (DSSCs).^57^

Second, among the reported subtriazaporphyrins, SubPzs afford the strongest electron acceptors upon peripheral substitution with, for example, nitro, phenyl, sulfanyl, methoxy, or amino groups.^42,43^

Third, SubPzs add superior tunability and controllability in terms of designing tailored absorption and fluorescence by taking the stronger impact that peripheral substituents exert onto the aromatic core,^54,58,59^ and negligible effects of axial substitution in their absorptions.‡‡In SubPs, however, both the absorption and fluorescence bands are influenced by the electron-donating abilities of the axial B-substituents, in good agreement with their respective Hammett inductive constants. This has been used to design a SubPz–pentacene conjugate for *intra*molecular singlet fission (*i*-SF),^60,61^ wherein the solvatochromic fluorescence of hexakis-(3,4,5-trimethoxyphenyl)subporphyrazine was a key feature to optimize the spectral overlap between the SubPz fluorescence and the pentacene-dimer absorption, and power a combination of *intra*molecular Förster Resonance Energy Transfer (*i*-FRET) and *i*-SF. In another recent example, we designed a subporphyrazine-dithienylethene photoswitch, whose near-infrared absorption was reversibly activated and deactivated with visible light.^62^

**Fig. 1:**
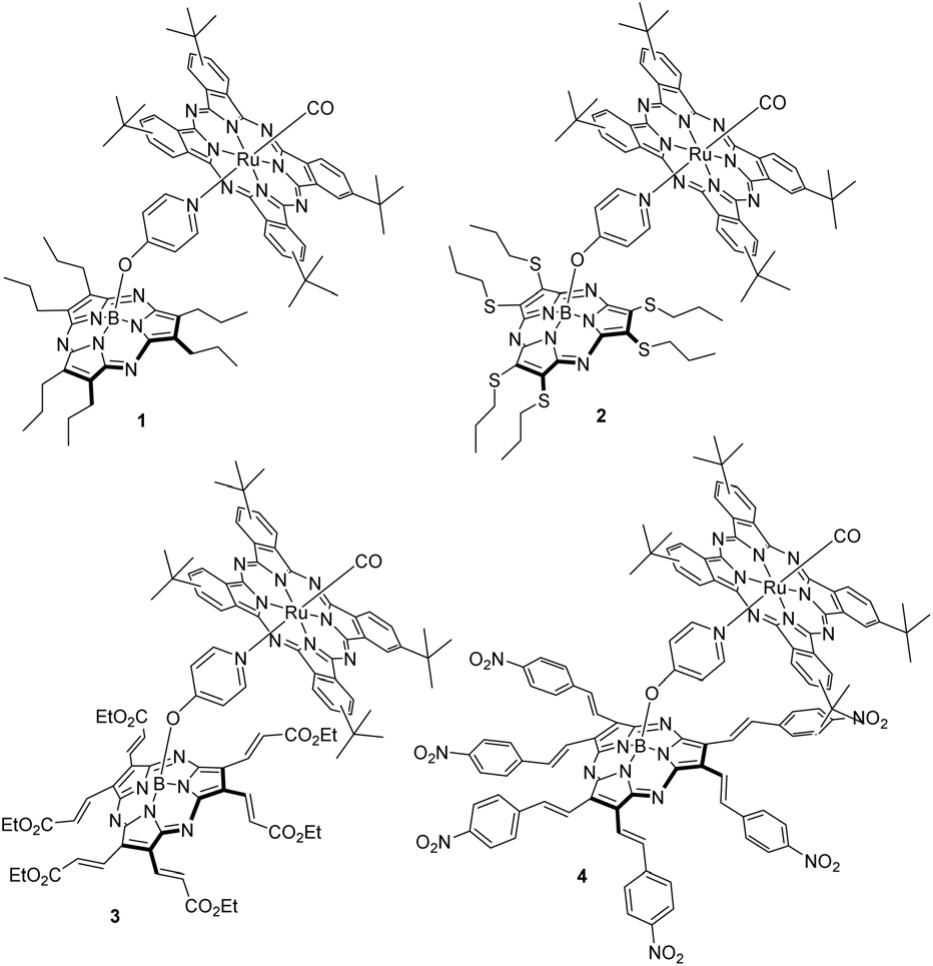
(Ru(CO)Pc-SubPz)s 1–4.

In the current work, we have designed a family of SubPzs with variable electron acceptor and donor strength. Modulation was realized by placing six ester, nitro, alkyl, or thioether units at the SubPz periphery, either directly attached to the macrocyclic core, or using vinylene/styryl linkers. By connecting the ester and nitro groups using the above-mentioned linkers, we transformed the SubPzs into strong electron acceptors.^58^ Using a modular approach, we have combined these new SubPz electron acceptors with a Ru(CO)Pc, that is, a RuPc coordinating one axial carbonyl ligand (Fig. 1). In the resulting D–A systems 1–4, SubPz and Ru(CO)Pc complementarily absorb light over a wide range of the solar spectrum, that is, from 250 to 700 nm. Their photoinduced energy and electron transfer events, including all excited state deactivation dynamics, were tested using steady-state absorption, fluorescence, electrochemistry, and time-resolved pump-probe measurements in the presence and absence of an external magnetic field.

Finally, DFT calculations were performed to back up our experimental observations. These findings contribute to the fundamental understanding of Ru(CO)Pc-SubPz conjugates and, in turn, pave the way for improved organic photovoltaics and molecular electronics.

## Results and discussion

### Synthesis

**Scheme 1:**
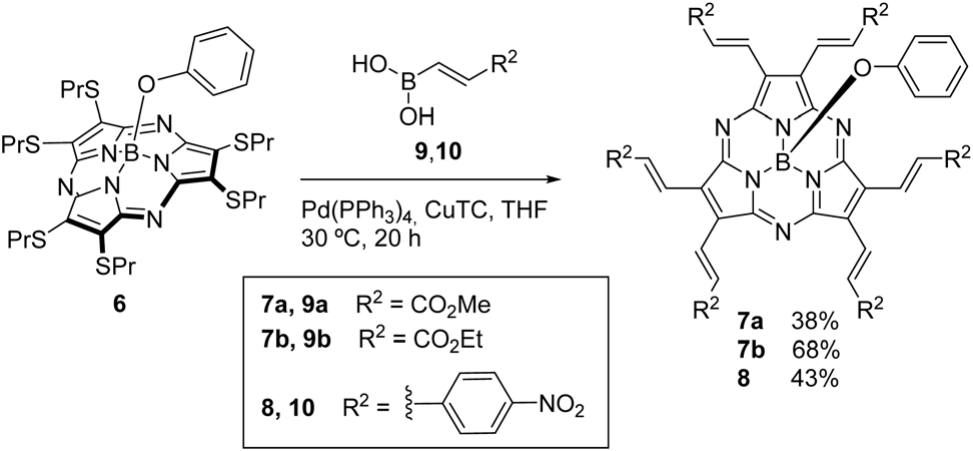
Synthesis of hexavinylene-SubPz electron acceptors 7a,b and 8.

The preparation of SubPzs 5 and 6 (Fig. S1) bearing a phenoxy group at the axial position, was carried out following reported procedures.^51,59^ The synthesis of the strongly electron accepting SubPzs 7a,b and 8 was accomplished in 38–68% and 43% yield, respectively. We used palladium-catalyzed, copper(i) thiophene-2-carboxylate (CuTC)-mediated coupling reactions of SubPz 6 with boronic acids 9a,b and 10 (Scheme 1).

To this end, the acrylate derivatives 9a,b were obtained through a hydroboration reaction of methyl and ethyl propiolate derivatives by adapting a reported procedure.^63,64^ The preparation of (*E*)-(4-nitrostyryl)boronic acid 10 was performed using the same procedure (Scheme S1). Our attempts to prepare this boronic acid by the reported procedure, treating 4-nitrophenylacetylene with catecholborane, failed.^65^

**Fig. 2:**
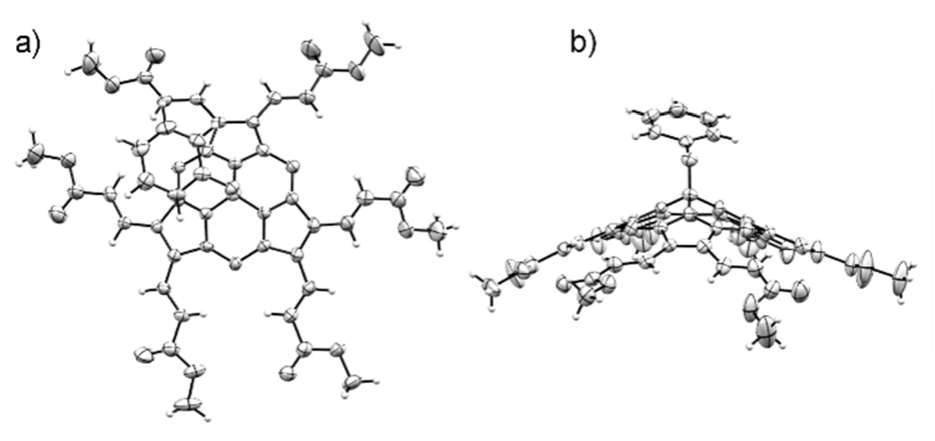
Molecular structure showing thermal ellipsoids at the 50% probability level of SubPz 7a.

A monocrystal of SubPz 7a was obtained by slow vapour diffusion of heptane into a solution of the SubPz in 1,2-dichloroethane, and the molecular structure was determined by X-ray diffraction analysis. As shown in Fig. 2, the six vinylene units of 7a are in *trans* configuration. The macrocycle displays a conical shape with a bowl depth of 1.619 Å. This is defined as the distance between the boron atom and the plane given by the six pyrrole β-carbons. As for the other SubPzs,^59^ all the dihedral angles between the vinylene units and the pyrrole plane to which they are connected are small. Five of them range between 2.34 and 6.09°, while the sixth is of 15.43°. Such a nearly planar arrangement gives rise to an extended conjugated π-system, able to electronically connect the electron-withdrawing ends with the macrocycle.

SubPzs 7a,b showed in ^1^H NMR the vinylene linker as two characteristic doublets around 8.2 and 8.0 ppm with *J* ∼16 Hz (Fig. S10 and S13, respectively). This denotes *trans*-stereochemistry, in line with the X-ray diffraction analyses. The six alkoxy units that correspond to the ester functions appeared as a singlet at *δ* = 3.95 ppm and a multiplet next to a triplet at 4.40 and 1.44 ppm, respectively. APCI HRMS confirmed the molecular ion of 7a at *m*/*z* = 841.1285–846.2528 corresponding to [M]^+^ + [M + H]^+^ (Fig. S12). The molecular ion of 7b appears in MALDI-TOF MS as two clusters at *m*/*z* = 948.3–951.3 (HRMS = 948.3185–951.3293) and *m*/*z* = 971.3–975.3 corresponding to [M + Na]^+^ and [M + 2Na]^+^, respectively (Fig. S14). Similarly, SubPz 8 displayed in ^1^H NMR the peripheral vinylene protons as two doublets at 9.15 and 8.28 ppm, accompanied by two other doublets at 8.38 and 8.16 ppm, assigned to the 4-nitrophenyl groups (Fig. S15). Interestingly, negative ion MALDI-TOF HRMS revealed [M]^−^ as a cluster at *m*/*z* = 1219.3056–1223.3170 (Fig. S16).

**Scheme 2:**
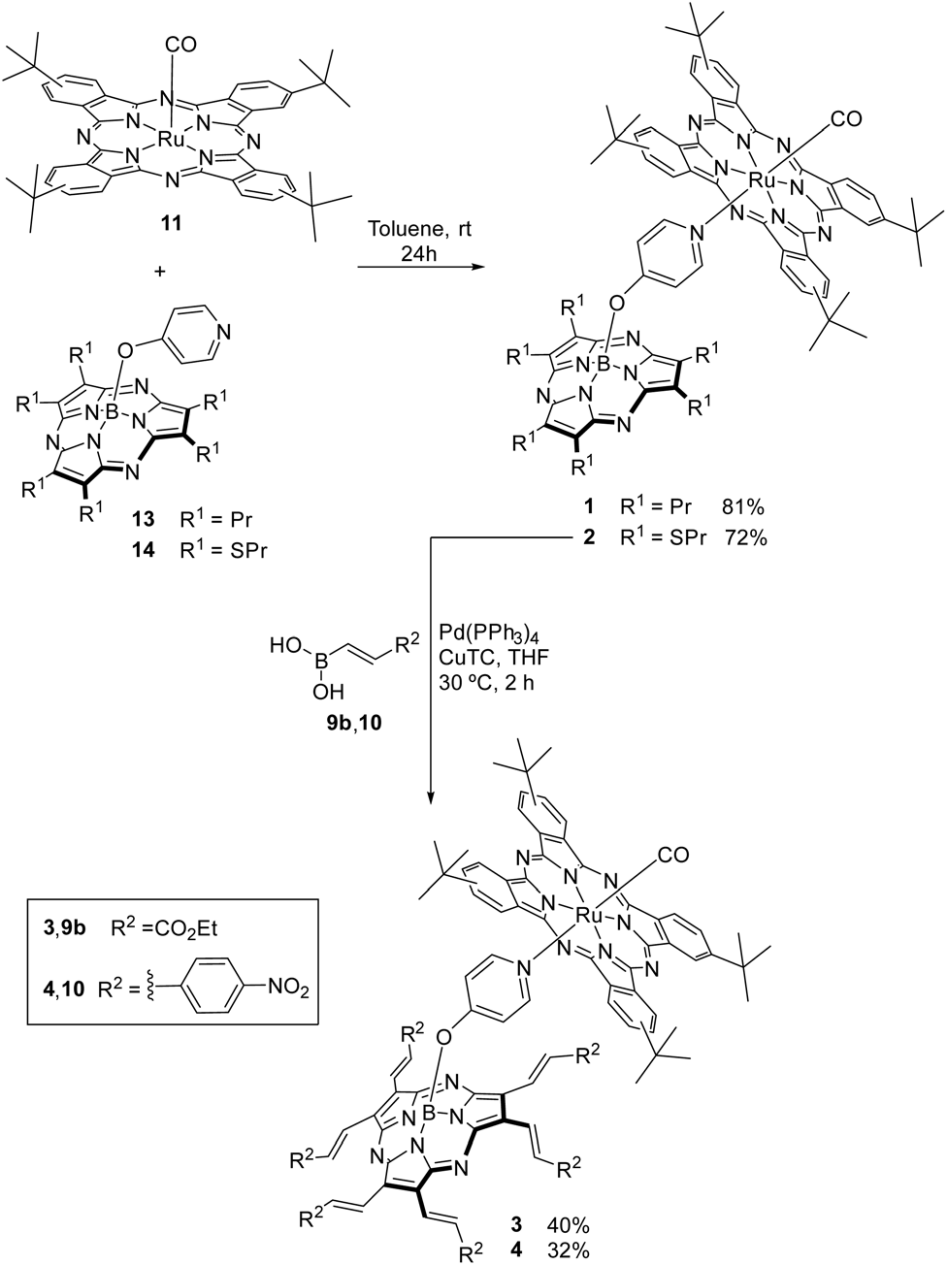
Synthetic pathways for Ru(CO)Pc-SubPz conjugates 1–4.

The Ru(CO)Pc precursor 11 (Scheme 2) was functionalized with four *tert*-butyl groups at the Pc periphery and a carbonyl group at one of the axial positions because this substitution pattern has given longer-lived charge-separated states in our previous examples.^34,36,37^

A Ru(CO)Pc ref. 12 (Fig. S1) was prepared by coordinating a pyridine at the other Ru(ii) coordination site using a reported procedure.^37^

Hexapropyl and hexasulfanyl SubPz precursors 13 and 14 bearing a pyridyloxy axial ligand were prepared in one pot by boron-assisted cyclotrimerization reaction of suitable maleonitriles, followed by substitution of the axial chloride ligand *via in situ* treatment with 4-hydroxypyridine, under standard conditions (Scheme S2).^53,59^ The ^1^H NMR spectra of 13 and 14 (Fig. S5 and S7, respectively) display the two doublets that correspond to the axial pyridyl rings and that are shielded by the SubPz diatropic ring current at 7.88 and 5.08 ppm, and 7.96 and 5.23 ppm, correspondingly.

Ru(CO)Pc-SubPz 1 and 2 were assembled by coordination of the corresponding axially substituted SubPzs 13 and 14 to Ru(CO)Pc 11 (Scheme 2) in 81% and 72%, respectively. Stoichiometric ratios of the individual chromophores were used.

Next, we focused on the preparation of the corresponding SubPz building blocks for the assembly of (Ru(CO)Pc-SubPz)s 3 and 4. SubPz 14 was subjected to Liebeskind Srogl coupling reactions with boronic acids 9b and 10 under the usual conditions for the peripheral functionalization of SubPzs with vinyl substituents.^59^ Under these conditions, the starting material disappeared within 30–120 minutes. However, the pyridyloxy-hexavinyl-SubPz was detected only in trace amounts that finally decomposed.

We hypothesized that a possible Michael addition of the pyridyl substituent of one SubPz to the strongly activated vinylene unit of another SubPz accounts for the instability. Accordingly, we envisaged that a pyridyloxy-SubPz coordinated to a Ru(CO)Pc would lose its nucleophilic character. Therefore, we performed Liebeskind Srogl cross-coupling reactions of Ru(CO)Pc-SubPz 2 with boronic acid derivatives 9b and 10 under our standard conditions (Scheme 2). The reactions were monitored by TLC, and the hexavinylation was indeed completed within two hours. 1 and 2 were purified by column chromatography on silica gel and Biobeads-SX1, respectively, while 3 and 4 were isolated in 40% and 32% yield after chromatography of the crude products on silica gel and Biobeads-SX1, respectively.

Ru(CO)Pc-SubPz 1 displayed in ESI^+^-HRMS a cluster at *m*/*z* = 1452.6953–1462.7001 (Fig. S19), which is assigned to the molecular ions [M]^+^ + [M + H]^+^, and which is accompanied by another cluster at *m*/*z* = 591.3988–594.4020. The latter corresponds to the loss of the [Ru(CO)Pc] fragment. In the ^1^H NMR spectrum, we noticed three well-resolved multiplets between 9.5 and 8.0 ppm, accounting for the aromatic protons of Ru(CO)Pc, in addition to the three multiplets corresponding to SubPz at 2.78, 1.65, and 0.89 ppm, respectively. The high field chemical shifts of the axial ligands are diagnostic of the coordination to Ru(CO)Pc. Hence, the pyridyl H-2,6 signals appear in 1 at 1.29 ppm, upfield shifted by 6.59 ppm upon coordination to Ru(CO)Pc, whereas the H-3,5 are upfield shifted by 2.19 ppm, appearing in 1 at 2.89 ppm (Fig. S17 and S18).^34–38^

2 shows in ^1^H NMR the two doublets corresponding to the pyridyl ligand at high fields, namely 3.02 and 1.36 ppm (Fig. S20). In addition, ESI^+^ HRMS exhibits the molecular ion of 2 as a cluster at *m*/*z* = 1643.5234–1657.5249 (Fig. S21), corresponding to [M + H]^+^ accompanied by a cluster at *m*/*z* = 782.2289–785.2226, assigned to the [M-Ru(CO)Pc]^+^ fragment.

Ru(CO)Pc-SubPz 3 showed in ^1^H NMR experiments the characteristic vinylene units as two doublets at 7.70 and 7.50 ppm, visibly shielded by the RuPc ring by 0.45 ppm related to the non-coordinated SubPz 7b, and close to the Pc aromatic protons between 9.4 and 8.0 ppm. A multiplet at 4.22 ppm is assigned to the OCH_2_ moieties, also slightly upfield shifted by 0.18 ppm related to the SubPz reference 7b, while H-3,5 pyridyl protons appear strongly upfield shifted at 3.15 ppm (Fig. S22), this accounting for the coordination of this ring to Ru(CO)Pc. MALDI-TOF MS shows the peak corresponding to [M]^+^ as a weak cluster at *m*/*z* = 1787.5–1800.5. It is also worth noting the presence of a weak cluster maximizing at *m*/*z* = 928.3 that corresponds to the expected molecular ion for the [SubPz-OPy]^+^ fragment in 3. Taking into account the synthetic procedure used for this conjugate, this cluster can only arise from the fragmentation of 3 under the MS conditions. MALDI-TOF HRMS could only be performed on the more intense fragmentation peaks, the most informative appearing at *m*/*z* = 1759.6230–1769.6475, assignable to [M-CO]^+^, which further proves the coordination of SubPz 7b to Ru(CO)Pc 11 (Fig. S23).

For 4, MALDI-TOF MS displays fragmentation peaks providing proofs for the assigned structure. Positive ion MALDI-TOF MS reveals a cluster at *m*/*z* = 860.3–871.4 assignable to the [M-(SubPz-OPy)]^+^ fragment, while negative ion MALDI-TOF MS shows a cluster at *m*/*z* = 1218.3–1225.3 corresponding to [M-Ru(CO)Pc]^−^ (Fig. S25). The presence of this SubPz fragment bearing a pyridyloxy axial ligand relates to 4, bearing in mind the synthetic precursors and procedure for this conjugate (Scheme 2). The ^1^H NMR spectrum of 4 is more complex than those of its congeners in that five broad multiplets between 9.5 and 7.3 ppm account for the Pc, vinylene, and nitrophenyl protons. Also here, it is possible to detect the structurally relevant pyridyl protons as two peaks at 3.76 and 1.28 ppm, respectively (Fig. S24).

### Electrochemical studies

To examine the electron accepting and donating strength of each SubPz, on one hand, and possible electronic coupling between them and the Ru(CO)Pcs, on the other hand, cyclic voltammetry (CV) and differential pulse voltammetry (DPV) were carried out with 1–4 and compared with ref. 5, 6, 7, 8, 11, 12, 13, and 14. In Fig. S26–S36, the corresponding CVs and DPVs are surveyed, and in Table 1, the corresponding values are compiled.

**Table 1:** Electrochemical reductions and oxidations *vs.* Fc/Fc^+^ in CH_2_Cl_2_, using a carbon electrode. The charge-separated state energies (*E*_CSS_) for toluene/THF as solvent were estimated using the continuum model

Compound	Potential/V, E_1/2_*vs.* Fc/Fc^+^									*E*_CSS._ DCM (eV)	*E*_CSS._ toluene (eV)
	Reduction						Oxidation				
	E^5−/6-^	E^4−/5−^	E^3−/4−^	E^2−/3−^	E^1−/2−^	E^0/1−^	E^0/1+^	E^1+/2+^	E^2+/3+^		
Ru(CO)Pc 11			—	−2.18	−1.75	−1.57	0.21	0.97	—	—	—
Ru(CO)PyPc 12	—	—	−2.13	−1.96	−1.73	−1.56	0.27	1.05	—	—	—
SubPz 5 ref. 50	—	—	—	—	—	−1.72	0.83	—	—	—	—
SubPz 13	—	—	—	—	−2.27	−1.65	0.79	0.98	—	—	—
SubPz 6	—	—	—	−2.06	−1.75	−1.25	0.89^a^	1.10^a^	—	—	—
SubPz 7a	—	—	−1.59	−1.35	−1.01	−0.76	—	—		—	—
SubPz 7b	—	—	−1.59	−1.35	−1.09	−0.75	—	—	—	—	—
SubPz^b^8	—	—	−2.63	−1.72	−1.52	−0.88	0.98	—	—	—	—
Ru(CO)Pc-SubPz 1	—	—	—	—	−2.01	−1.60	0.25	1.02	—	1.63	1.75
Ru(CO)Pc-SubPz 2	−2.14	−1.98	−1.73	−1.61	−1.52	−1.18	0.29	0.58	1.04	1.25	1.37
Ru(CO)Pc-SubPz 3	—	−2.06	−1.56	−1.31	−1.21	−0.69	0.28	0.37	1.05	0.74	0.76
Ru(CO)Pc-SubPz 4	−2.04	−1.86	−1.57	−1.44	−1.25	−0.95	0.33	1.02	—	1.05	1.05^c^

a Value taken from ref. 59.

b measured in THF.

c CSS energy value calculated for THF as solvent.

For Ru(CO)Pc, all redox processes are ligand-based rather than metal-based, in sound agreement with previous electrochemical investigations.^37^ The axial coordination of pyridine to Ru(CO)Pc 11 renders Ru(CO)PyPc 12 by about 60 mV more difficult to be oxidized. Besides, 12 features four reductions that happen to be 10 mV easier in comparison to what is seen for Ru(CO)Pc 11, which displays three reductions.

SubPz 5 exhibits a reversible oxidation and a reversible reduction at +0.83 V and −1.72 V *vs.* Fc/Fc^+^, respectively,^50^ while SubPz 6 displays the first of three reductions at −1.25 V next to oxidation peaks at +0.89 and +1.10 V *vs.* Fc/Fc^+^.^59^ Placing an axial pyridine slightly impacts the redox properties of the SubPzs. For example, 13 shows two oxidations and two reductions. The first oxidation and reduction are, hereby, noted at +0.79 and −1.65 V *vs.* Fc/Fc^+^, respectively. Likewise, peripheral functionalization in 7a,b and 8 with electron-withdrawing units alters the SubPz redox characteristics. SubPzs 7a,b show, for example, four reversible reductions between −0.75 and −1.59 V *vs.* Fc/Fc^+^. The first reduction at around −0.75 V is anodically shifted by 970 mV with respect to that of SubPz 5. SubPz 8 is also a strong electron acceptor, which is reflected by its first reduction at −0.88 V *vs.* Fc/Fc^+^. This is 840 mV less negative than the corresponding reduction of 5. With a first oxidation at +0.98 V *vs.* Fc/Fc^+^, SubPz 8 is also 150 mV more difficult to be oxidized than 5. The lower reduction potential observed for 7 relative to 8 might result from a higher coplanarity between the terminal accepting groups and the macrocycle in the hexavinylated-SubPz related to the hexastyryl-SubPz.§§Dihedral angles ranging from 2.92 to 32.7° have been observed between terminal phenyl groups and pyrrole rings in hexastyryl-SubPz. Other additional effects such as solvation, reorganization energy, and differences in the stabilization of electronic states, could together modulate the redox behaviour beyond simple electron-withdrawing strength of the ester and nitro functions, respectively.

Upon coordination to Ru(CO)Pc, the first reduction of 1 corresponds to that of Ru(CO)Pc, whereas those of 2 and 3 are SubPz-based, albeit slightly altered relative to 6 and 7b. In particular, anodic shifts of 70 and 60 mV, respectively, evolve.

The first reductions of 2, 3, and 4 with respect to that of 1, which appears at −1.60 V *vs.* Fc/Fc^+^, are subject to anodic shifts. The strongest shift of 910 mV is seen for 3, followed by 650 mV for 4. The anodic shift for all reductions in 3 and 4 evidences their strong electron-accepting character. With respect to the latter, 2, 3, and 4 show 40, 30, and 80 mV, respectively, more positive first oxidations when compared with 1.

With the oxidations and reductions at hand, the energies of the charge-separated states (*E*_css_) were determined using the continuum model for electron transfer (Table 1).^66^ An energy of 1.75 eV for 1 suggests that an electron transfer process is thermodynamically unfavorable. In contrast, values of 1.37, 0.76, and 1.05 eV for 2, 3, and 4, respectively, indicate that an electron transfer from Ru(CO)Pc to SubPz is likely to proceed. Independent confirmation of this trend stems from the HOMO–LUMO energy gaps, as obtained from DFT calculations (Fig. S73).

### Steady-state photophysical characterization

**Fig. 3:**
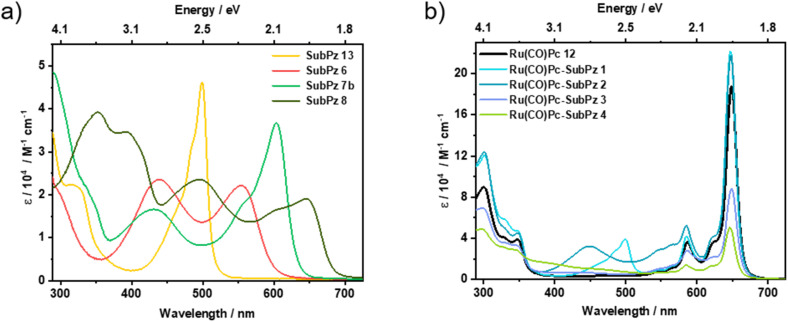
Absorption spectra of (a) SubPzs 13, 6, and 7b in toluene and 8 in THF, (b) Ru(CO)Pc 12 as well as Ru(CO)Pc-SubPz conjugates 1, 2, and 3 in toluene and 4 in THF, at room temperature.

Table 2 summarizes the absorption and fluorescence data. Initial insights into the photophysics of SubPzs 13, 6, 7a,b, and 8 were garnered through steady-state absorption and fluorescence measurements, which were recorded in toluene and tetrahydrofuran (THF) (Fig. 3). In the absorption spectrum of 5 and 13, characteristic features such as Soret-band absorptions at around 285 and 325 nm, as well as a strong Q-band absorption at 499 nm, are observable. Contrary to the redox potentials, no changes are observed in the absorption spectrum upon axial pyridyloxy substitution compared to the axial phenoxy functionalization.^53^6 and 14 exhibit three main features, two of which appear at around 280 and 560 nm and are attributed to the Soret- and Q-band absorptions, respectively (Fig. S9). The third is seen at around 440 nm and is assigned to a *n* → π* transition from the peripheral sulfanyl group to the SubPz core.^54^ In the case of 7a,b and 8, peripheral substitutions of methyl/ethyl acrylate and nitrostyrene, respectively, result in an increased π-conjugation and extend the absorptions well beyond 600–650 nm. A Soret-band absorption in the 300 nm region and an intense Q-band absorption at 602 nm are discerned for 7a,b. For 8, features at 353 and 394 nm correspond to Soret-band absorptions, while a broadened Q-band absorption is notable at 648 nm.

**Table 2:** Soret- and Q-band maxima, fluorescence maxima (*λ*_em_), fluorescence quantum yields (*φ*_F_), and singlet oxygen quantum yields (*φ*_Δ_) for Ru(CO)Pc 12, SubPzs 13, 6, 7b, and 8, as well as Ru(CO)Pc-SubPz conjugates 1, 2, 3, and 4

Compound	Soret-bands (nm)		Q-bands (nm)		*λ*_em_ a (nm)	*φ*_F_ a	*φ*_Δ_	
	SubPz	Ru(CO)Pc	SubPz	Ru(CO)Pc			*λ*_ex_ = 500 nm	*λ*_ex_ = 615 nm
Ru(CO)Pc^b^12	—	300	—	649	657	0.005	—	0.90
SubPz^b^13	285, 325	—	499	—	515	0.019	0.21	—
SubPz^b^6	300	—	550	—	508, ≈730	0.026	0.72	—
SubPz^b^7b	300	—	602	—	622	0.015	0.19	—
SubPz^c^8	353, 394	—	648	—	670	0.031	—	—
Ru(CO)Pc-SubPz^b^1	250–350		502	648	515, 657	0.006	0.82	0.96
Ru(CO)Pc-SubPz^b^2	250–350		—	648	508, 657	<0.005	0.88	0.93
Ru(CO)Pc-SubPz^b^3	250–350		—	649	622	0.009	0.18	0.25
Ru(CO)Pc-SubPz^c^4	250–350		—	649	—	<0.005	—	—

a Photo-excitation at 590 nm for Ru(CO)Pc 12 and 470 nm for SubPzs 13, 6, 7b, and 8, as well as Ru(CO)Pc-SubPz conjugates 1, 2, 3, and 4.

b measured in toluene.

c measured in THF.

Ru(CO)Pc 12 reveals, in line with literature reports, the characteristic absorption features consisting of Soret- and an intense Q-band absorptions at 300 and 649 nm, respectively.^34,37^

Axial coordination of 13 lacks any notable alterations in the overall absorption of 1 compared to the respective references. At this point, we conclude the absence of sizeable ground-state interactions. For the remaining three Ru(CO)Pc-SubPz conjugates 2–4, detectable electronic interactions are derived. For example, for 2, a 10 nm bathochromic shift of the 440 nm absorption band emerges with respect to ref. 6. In 3 and 4, the molar extinction coefficients are lower than the simple sum of the references. In addition, a red-shifted absorption band at 445 nm and a blue-shifted band at 470 nm are observed in 3 and 4, respectively (Fig. S37 and S38).¶¶With the exception of conjugate 4, no significant differences in the absorption are observed for measurements performed in THF compared to toluene. 4 is only sparingly soluble in toluene.

Initially, excited state interactions were tested in fluorescence measurements (Fig. S39–S42). A fluorescence maximum at 515 nm implies a 16 nm Stokes-shifted fluorescence for 13. The absolute fluorescence quantum yield is 0.02, much lower compared to other hexapropyl-substituted SubPzs (0.1–0.2).^50–53,56^ This points once more to appreciable interactions between SubPz and the pyridyloxy axial ligand. Photoexcitation into the Q-band absorption of 6 results in a broad fluorescence that ranges from 600 to 900 nm, for which we determined a quantum yield of 0.026. However, when photoexcited at, for example, 400 nm, 6 displays an additional albeit weak feature that is centered at 508 nm. We assign this to an emission that originates from the interactions of the sulfanyl group with the SubPz macrocycle. 7b and 8 exhibit Q-band fluorescence at 622 and 670 nm, respectively, and quantum yields of 0.015 and 0.031. Photoexcitation of Ru(CO)Pc 12 at 590 nm is associated with a weak fluorescence and a quantum yield of 0.005. It maximizes at 657 nm and shoulders at 715 nm. Time-correlated single photon counting (TCSPC) measurements revealed a fluorescence lifetime of 1.09 ns for SubPz 8, while all other SubPz and RuPc references exhibited lifetimes of less than 500 ps.

Starting with Ru(CO)Pc-SubPz 1, photoexcitation into the Q-band absorption of SubPz at 470 nm results in a fluorescence pattern that resembles that seen for 13, although the quantum yield is less than 0.01. Upon closer inspection, a weak 657 nm fluorescence is observed, which corresponds to that of Ru(CO)Pc. This is indicative of an *i*-FRET from SubPz to Ru(CO)Pc in 1. In 2, photoexcitation at 440 nm results in the same 508 nm emission that is seen when probing 6. Concomitant is a rise of the Ru(CO)Pc-centered fluorescence, which is indicative of an efficient *i*-FRET. As such, the Ru(CO)Pc-centered fluorescence comes at the expense of the broad fluorescence of SubPz in 2, in the range of 600 to 900 nm. The latter is nearly quantitatively quenched with a quantum yield well below 0.005. SubPz-centered photoexcitation of 3 results in a weakly detectable fluorescence, which, nevertheless, resembles that of SubPz 7b. Fluorescence corresponding to Ru(CO)Pc is not observed. Still, a good spectral overlap between the SubPz fluorescence and the Ru(CO)Pc absorption points to a possible *i*-FRET. Finally, photoexcitation of neither SubPz nor Ru(CO)Pc in 4 results in any sizeable fluorescence. Quantitatively overlapping absorptions from both chromophores rule out the possibility that *i*-FRET is responsible for the fluorescence quenching.

### Time-resolved photophysical characterization

To gain mechanistic and kinetic insights into the excited state deactivations, all samples were probed by means of transient absorption spectroscopy (TAS). Measurements on the femtosecond (fs-TAS) and nanosecond (ns-TAS) timescales were performed by photo-exciting SubPzs 13, 6, 7b, and 8 at 480 nm, Ru(CO)Pc 12 at 660 nm, as well as Ru(CO)Pc-SubPz 1, 2, 3, and 4 at either 480 or 660 nm to almost exclusively excite SubPz or Ru(CO)Pc, respectively.

Upon photoexcitation at 480 nm, all SubPzs displayed almost the same deactivation pathways involving three species. For hexapropyl-SubPz 13, photoexcitation leads to differential absorption spectra that include ground-state bleaching (GSB) in the range of Q-band absorption at 500 nm and excited state absorption (ESA) that is broad between 590 and 760 nm, and that has a maximum at 660 nm (Fig. S47). All of these features stem from a hot singlet excited state (*S_1_). What follows is a vibrational relaxation in 11 ps to populate the singlet excited state (S_1_). With a lifetime of 226 ps, (S_1_) decays by fluorescence, and a fraction of it is subject to intersystem crossing (ISC) to yield the corresponding triplet excited state (T_1_). As evaluated from the ns-TAS data (Fig. S48), (T_1_) deactivates to the ground state with a lifetime of 804 ns under anaerobic conditions. Albeit the difficulty in observing a clear singlet-to-triplet transformation from the differential absorption changes, (T_1_) formation was corroborated in singlet oxygen measurements.

For hexasulfanyl-SubPz 6, following its photoexcitation, GSB between 440 and 580 nm with minima at 445 and 550 nm as well as ESA spanning from 590 to 760 nm are observed (Fig. S49–S50). These features are attributed to the vibrationally hot singlet excited state (*S_1_) of SubPz. In less than 1 ps, (*S_1_) undergoes vibrational relaxation to populate (S_1_), followed by ISC within 203 ps to populate the energetically lower-lying (T_1_). This is accompanied by a slightly blue-shifted ESA, with a maximum that is centered around 628 nm. (T_1_) lives for 6.2 µs before it decays to the ground state.

The transient spectra of (*S_1_), formed upon photoexcitation of hexaacrylate-SubPz 7b, are defined by a broad GSB in the region between 540 and 640 nm with a minimum at 608 nm along with ESA at wavelengths that flank GSB (Fig. S51). Vibrational relaxation from (*S_1_) occurs within 9.3 ps and is followed by ISC within 311 ps. The correspondingly formed (T_1_) features ESA maxima at 392, 513, and 656 nm and decays within 30.9 µs (Fig. S52).

In the case of hexastyryl-SubPz 8, 480 nm photoexcitation initially populates (*S_1_). The differential spectra recorded after the photo-excitation display GSB minima at 502 and 652 nm. ESA maxima are also noted at 442, 559, and beyond 675 nm (Fig. S53). Vibrational relaxation into (S_1_) and subsequent deactivation *via* ISC to afford (T_1_) occur within 102 ps and 1.17 ns, respectively. The population of (T_1_) is accompanied by characteristic transient features, including a broadened and blue-shifted GSB between 595 and 665 nm, the rise of ESA around 400 nm, as well as blue-shifted ESA maxima at 435 and 547 nm. The (T_1_) lifetime is 8.7 µs (Fig. S54).

Upon photoexcitation of 12 at 660 nm in toluene and THF, Ru(CO)Pc displays characteristic (S_1_) features that include ESA maxima at 450, 525, 607, 692, and 743 nm next to an ESA minimum at 585 nm (Fig. S55–S58). (S_1_) is short-lived and undergoes a rapid ISC within a few picoseconds due to the heavy atom effect of ruthenium. (T_1_), which is formed accordingly, features ESA maxima at 515 and 730 nm, in addition to a GSB minimum at 623 nm. (T_1_) lifetime is several microseconds.

In the context of exciting conjugates 1, 2, 3, and 4, we first focus on the discussions of 480 nm photo-excitation experiments.

**Fig. 4:**
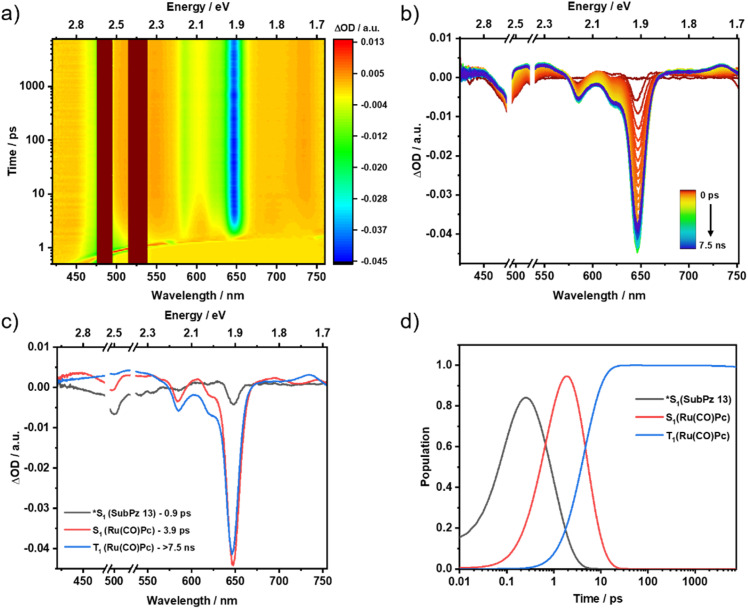
fs-TAS raw data from pump-probe experiments and the corresponding global sequential analysis for Ru(CO)Pc-SubPz conjugate 1, following 480 nm photoexcitation in argon-saturated toluene at room temperature. (a) Heat map of fs-TAS raw data. (b) Differential absorption spectra at time delays between 0 ps and 7.5 ns. (c) Evolution-associated spectra with their corresponding lifetimes, obtained from the deconvolution of the fs-TAS data. (d) Relative populations of the respective species.

Photoexcitation of 1 is SubPz-centered as evidenced by GSB at 500 nm and ESA between 590 and 760 nm (Fig. 4). In addition, the weak 648 nm GSB is Ru(CO)Pc-centered and corroborates strong couplings between SubPz and Ru(CO)Pc. We assign the first species to (*S_1_). Compared to (*S_1_) of ref. 13, it is very short-lived. It is within 0.9 ps that it decays by means of populating the second species, whose features match those of Ru(CO)Pc (S_1_). Such a fast transformation confirms an efficient *i*-FRET from SubPz to Ru(CO)Pc. Considering the fluorescence quantum yield of the hexapropyl-SubPz energy donor 13, and the energy donor–acceptor distance obtained from DFT calculations, an *i*-FRET rate constant of 6.2 ×10^12^ s^−1^ was estimated (Table S1). Subsequently, an ISC yields (T_1_) within 3.9 ps as the third species before recovery of (S_0_) follows (Fig. S59). Notably, *i*-CS is thermodynamically unfeasible to occur from any species, that is, SubPz (*S_1_), Ru(CO)Pc (S_1_), and Ru(CO)Pc (T_1_), as the charge-separated state (CSS) is above 1.7 eV.

**Fig. 5:**
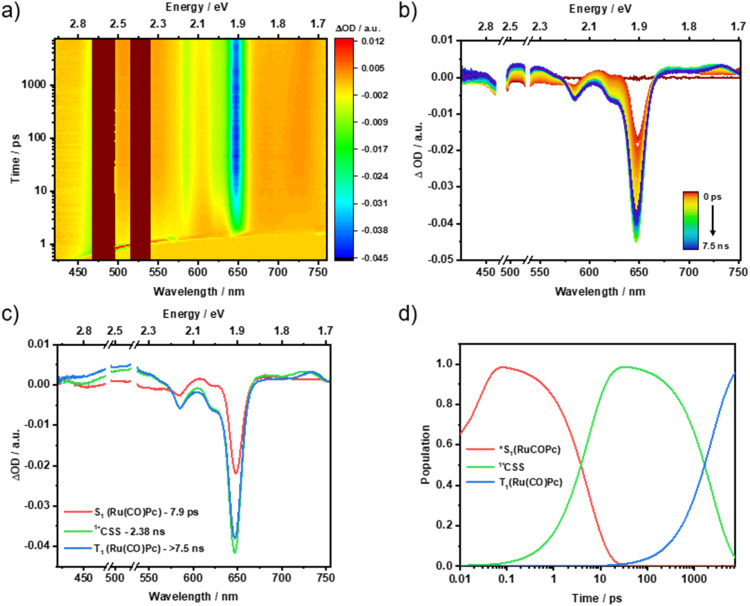
fs-TAS raw data from pump-probe experiments and the corresponding global sequential analysis for Ru(CO)Pc-SubPz conjugate 2, following 480 nm photoexcitation in argon-saturated toluene at room temperature. (a) Heat map of fs-TAS raw data. (b) Differential absorption spectra at time delays between 0 ps and 7.5 ns. (c) Evolution-associated spectra with their corresponding lifetimes, obtained from the deconvolution of the fs-TAS data. (d) Relative populations of the respective species.

As summarized in Fig. 5, for 2, the first species, which is formed following 480 nm photoexcitation, displays GSB at 650 nm and ESAs at 525, 607, 690, and 742 nm that exactly agree with what is seen for Ru(CO)Pc-centered (S_1_). This confirms our findings from the fluorescence measurements that the SubPz-centered (*S_1_) formed upon photoexcitation undergoes an ultrafast energy transfer to (S_1_) of Ru(CO)Pc. It all happens on time-scales faster than the time resolution of our experimental setup. Rather than the fast ISC seen in the Ru(CO)Pc ref. 12, by which (T_1_) is formed, we notice that the conclusion of (S_1_) decay is linked to the evolution of a 725 nm ESA maximum. A comparison of this second species with the spectroelectrochemical oxidation of Ru(CO)Pc 12 underlines the close match with the fingerprints of the one-electron oxidized form of Ru(CO)Pc (Fig. S43). Turning to the one-electron reduced form of SubPz 6, we take the 450 nm GSB minimum as evidence for an *intra*molecular charge separation (*i*-CS). Thus, the second species is attributed to the singlet charge-separated state (^1^*CSS) Ru(CO)Pc˙^+^-SubPz˙^−^ of 2, whose energy is 1.37 eV, below that of SubPz 6 (S_1_) and Ru(CO)Pc 12 (S_1_). With a lifetime of 2.38 ns, (^1^*CSS) undergoes charge recombination to yield the Ru(CO)Pc-centered (T_1_) at 1.32 eV as the third species and eventually (S_0_) (Fig. S60).

**Fig. 6:**
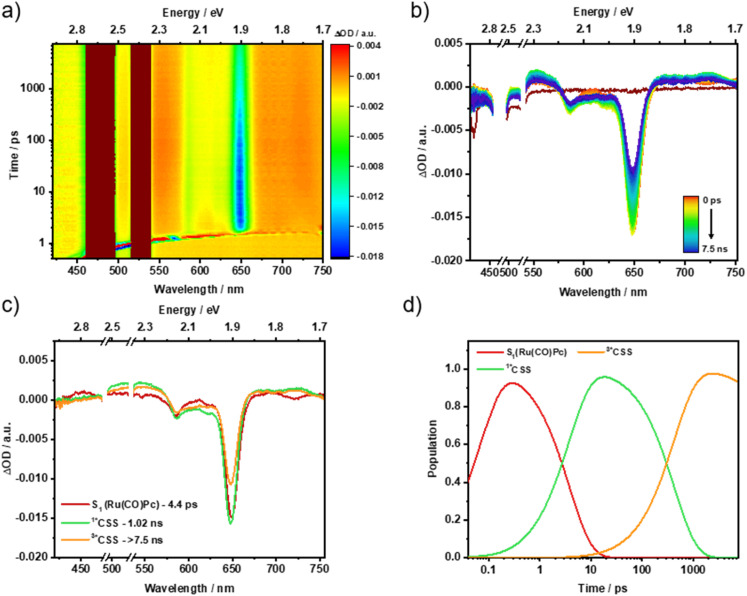
fs-TAS raw data from pump-probe experiments and the corresponding global sequential analysis for Ru(CO)Pc-SubPz conjugate 3, following 480 nm photoexcitation in argon-saturated toluene at room temperature. (a) Heat map of fs-TAS raw data. (b) Differential absorption spectra at time delays between 0 ps and 7.5 ns. (c) Evolution-associated spectra with their corresponding lifetimes, obtained from the deconvolution of the fs-TAS data. (d) Relative populations of the respective species.

For Ru(CO)Pc-SubPz 3, 480 nm photoexcitation generates Ru(CO)Pc-centered (S_1_) as the first species, which exhibits ESAs in the 450, 515, 610, 695, and 740 nm regions along with a GSB at 650 nm. This serves as direct evidence of an ultrafast energy transfer process that takes place from the SubPz-centered (S_1_) to the Ru(CO)Pc (S_1_). Following the *i*-FRET, it is within 4.4 ps that what appears like (^1^*CSS) is being formed by means of *i*-CS as the main deactivation channel. Spectroscopic fingerprints of the one-electron oxidized form of Ru(CO)Pc and the one-electron reduced form of SubPz 7b at 725 and 466/675 nm, respectively, support this assignment (Fig. S45). 1.02 ns is the lifetime of (^1^*CSS), and its energy is 0.82 eV. The third species is spectrally identical to the second species. In other words, both of them are (*CSS) with either an overall singlet (^1^*CSS) or triplet (^3^*CSS) character. (^3^*CSS) lives for 39 ns and its fate is charge recombination to reinstate (S_0_) (Fig. 6). As corroborated in the ns-TAS measurements, a minor fraction of Ru(CO)Pc (S_1_) also undergoes ISC to afford Ru(CO)Pc-centered (T_1_) at 1.32 eV, which lives for 3.2 µs (Fig. S61).

**Fig. 7:**
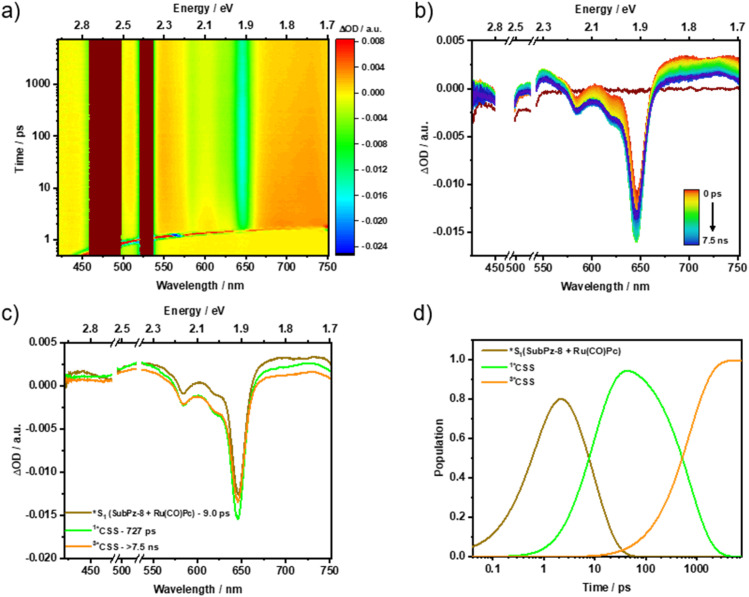
fs-TAS raw data from pump-probe experiments and the corresponding global sequential analysis for Ru(CO)Pc-SubPz conjugate 4, following 480 nm photoexcitation in argon-saturated THF at room temperature. (a) Heat map of fs-TAS raw data. (b) Differential absorption spectra at time delays between 0 ps and 7.5 ns. (c) Evolution-associated spectra with their corresponding lifetimes, obtained from the deconvolution of the fs-TAS data. (d) Relative populations of the respective species.

Photoexcitation of 4 induces differential absorption changes that are best described as the superimposition of Ru(CO)Pc (S_1_) and SubPz 8 (S_1_) with ESAs at 430, 530, 675, and 735 nm regions, and a GSB at 650 nm. Considering the lower molar extinction coefficient of 4 relative to the individual chromophores, it is evident that SubPz, as well as Ru(CO)Pc, contribute to the absorption at 480 nm. Accordingly, we assign the initially formed species to a mixture of vibrationally hot (*S_1_) states of both Ru(CO)Pc and SubPz 8. Following its decay in 9 ps, the emergence of a second species is noted, which reveals ESA maxima at 437, 535, and 729 nm and a GSB minimum at 650 nm (Fig. 7). These features clearly relate to the one-electron oxidized form of Ru(CO)Pc and the one-electron reduced form of SubPz 8 (Fig. S46). Thus, the second species is the product of *i-*CS, that is, (^1^*CSS), and its energy is 1.04 eV. In 727 ps, (^1^*CSS) evolves into an even longer-lived species with, however, identical spectroscopic features. Like our conclusion with 3, (^1^*CSS) spin flips in 4 to yield (^3*^CSS) as the third species. The triplet nature renders it very long-lived, as the Ru(CO)Pc-centered (T_1_) is, with 1.32 eV too high in energy, and the underlying recovery of (S_0_) takes 2.1 µs (Fig. S62).

660 nm photoexcitation of 1 and 2 results exclusively in Ru(CO)Pc (S_1_), including ESA maxima at 452, 606, 697, and 744 nm (Fig. S63–S66). It is within 5 ps that from this first species ISC occurs and Ru(CO)Pc (T_1_) is populated. Characteristics of this second species are ESA maxima at 515 and 730 nm. The finding of a ground state recovering from Ru(CO)Pc (T_1_) in 1 is in sound agreement with the outcome of the 480 nm photoexcitation experiments. For 2, however, the lack of *i*-CS implies that ISC outcompetes it. We believe that the difference between these two pathways relates to an excess of vibrational energy that Ru(CO)Pc (S_1_) carries over due to *i*-FRET once 480 nm photoexcited, and that Ru(CO)Pc (S_1_) lacks if 660 nm photoexcited. From Ru(CO)Pc (T_1_), the recovery of the ground state happens to occur on a timescale of up to 5 µs.

In stark contrast, Ru(CO)Pc (S_1_) which is formed as the first species by photoexciting 3 deactivates in 4.1 ps *via* a branching mechanism to lead to the second and third species. On one hand, (^1^*CSS) formation is the major reaction channel. Evidence for (^1^*CSS) comes from broad ESAs in the 400 to 570 nm range and an ESA maximum at 723 nm (Fig. S67–S68). On the other hand, in a minor pathway, Ru(CO)Pc (S_1_) is subject to ISC and yields Ru(CO)Pc (T_1_), for which a singlet oxygen quantum yield of 0.25 evolves. In 628 ps, (^1^*CSS) spin flips to yield (^3^*CSS). (^3^*CSS), which is the fourth species, undergoes charge recombination to populate the ground state in 44 ns.

**Fig. 8:**
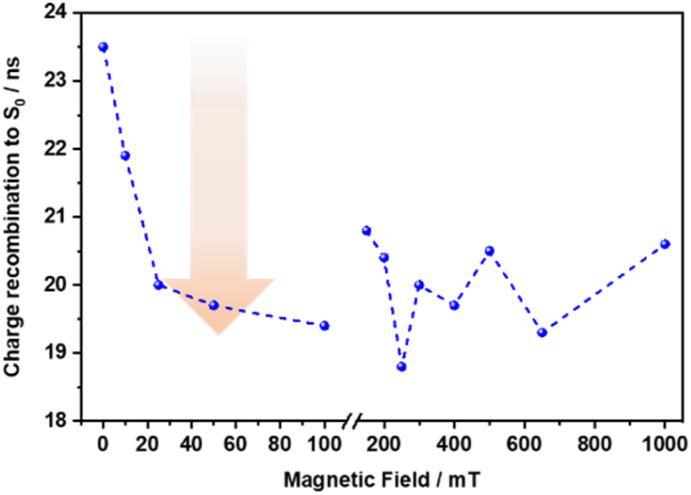
(^3^*CSS) lifetime of 660 nm photoexcited Ru(CO)Pc-SubPz conjugate 3 as a function of applied magnetic field.

To elucidate the spin evolution of the charge-separated state, the influence of an external magnetic field on the charge recombination dynamics was investigated. This was done by recording the nanosecond transient absorption spectra by varying the magnetic field from 0 to 1000 mT. Upon 660 nm photoexcitation of 3, the charge recombination of (^3^*CSS) to the ground state is completed within 23.5 ns.||||This lifetime is slightly shorter compared to the previously observed value of 44 ns, potentially due to the influence of oxygen under the experimental conditions. Notably, the (^3^*CSS) lifetime species showed a 17% reduction upon increasing the magnetic field from 0 to 100 mT. This can be attributed to the hyperfine coupling in the low-field regime, which plays a crucial role in charge recombination by facilitating singlet-triplet mixing in charge-separated states.^67,68^ At magnetic fields exceeding 100 mT, the Zeeman interaction progressively dominates over hyperfine coupling, thereby suppressing singlet-triplet mixing.^69^ Consequently, (^3^*CSS) lifetime remains relatively constant and shows no discernible trend in the higher magnetic field regime (Fig. 8).**480 nm photoexcitation of 3 posed some challenges, primarily due to its low extinction coefficient. These limitations necessitated the use of high laser excitation powers exceeding 1 µJ, which led to the gradual decomposition of the compound. Nevertheless, a similar trend in reduction of the (^3^*CSS) lifetime was observed initially upon increasing the magnetic field up to 5 mT (Fig. S71).

**Fig. 9:**
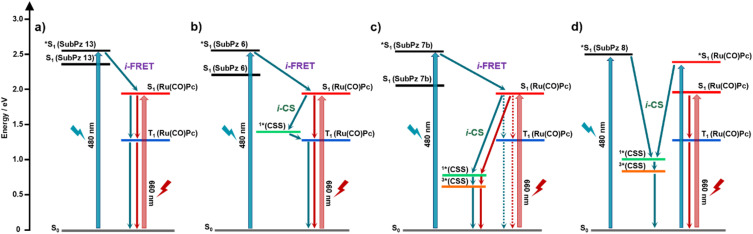
Energy diagrams depicting the deactivation pathways upon photoexciting either SubPz or Ru(CO)Pc in the Ru(CO)Pc-SubPz conjugates (a) 1, (b) 2, and (c) 3 in toluene, and (d) 4 in THF. The blue and the red arrows correspond to the deactivation pathways upon 480 and 660 nm excitations, respectively. The dotted lines represent the deactivation of a minor fraction. We note that the exact energy difference between ^1^*(CSS) and ^3^*(CSS) is unknown and the difference shown in the graph is for clarity purposes only.

In the case of Ru(CO)Pc-SubPz 4, photoexcitation generates Ru(CO)Pc (S_1_). Unlike 480 nm photoexcitation, this first species is localized in nature. This is the rationale why it decays with a 6.1 ps lifetime *via* ISC instead of *i*-CS. Spectroscopic and kinetic features of Ru(CO)Pc (T_1_) are ESA maxima at 515 and 730 nm and a lifetime of 4.8 µs, respectively (Fig. S69–S70).††††As with 3, magnetic-field-dependent ns-TAS measurements of 4 under 480 nm photoexcitation were hindered by its significantly lower extinction coefficient, which necessitated high laser excitation powers and led to sample decomposition under experimental conditions.Fig. 9 summarizes the excited-state deactivation pathways observed for all the conjugates.

## Conclusion

SubPz 5, 6, 7a,b, and 8 were utilized as versatile electron acceptors in a modular strategy that is based on coordinating them axially to a Ru(CO)Pc electron donor *via* pyridyloxy substituents. The oxidizing strength and the absorption cross section of the hexasubstituted SubPzs in the electron donor–acceptor conjugates 1–4 were adjusted by peripheral functionalization. This was essential to control the photophysical behavior of 1–4. SubPzs 6, 7a,b, and 8 are, in contrast to 5, good electron-acceptors, which is evident when inspecting their reduction strength. The excited state interactions involve an interplay of *intra*molecular Förster resonance energy transfer (*i*-FRET) and *intra*molecular charge separation (*i*-CS). On one hand, fluorescence spectroscopy provided initial insights into *i*-FRET, where the SubPz-centered excitation displayed fluorescence that stemmed from a Ru(CO)Pc-centered species. On the other hand, shifts observed in the absorptions and changes in the relevant HOMO–LUMO energies derived from electrochemistry and DFT calculations gave clear evidence for sizeable electronic couplings as a means to power *i*-CS. Deeper insights into the exact deactivation mechanism were obtained through time-resolved pump-probe transient absorption measurements.

SubPz photoexcitation in Ru(CO)Pc-SubPz 1 results exclusively in a rapid *i*-FRET with an underlying rate constant of 6.2 × 10^12^ s^−1^. Whereas for 2 and 3, an ultrafast *i*-FRET followed by an *i*-CS is the modus operandi. The absence of any spectral overlap between the fluorescence of SubPz 8 and the absorption of Ru(CO)Pc shuts down *i*-FRET in 4. As a matter of fact, *i*-CS from Ru(CO)Pc to SubPz 8 evolves as the predominant deactivation pathway. It is imperative to note that in the case of 2, the deactivation of the charge-separated state proceeds *via* the Ru(CO)Pc triplet excited state. For 3 and 4, however, an interconversion between the singlet (^1^*CSS) and triplet charge-separated (^3^*CSS) states enables lifetimes all the way up to 2.1 µs.

## Author contributions

E. C.-E. and D. G. synthesized the studied molecules. S. K. performed the photophysical studies. M. S. R.-M., T. T., and D. M. G. designed the research. Y. B. and T. C. conducted theoretical calculations. S. K., M. S. R.-M., and D. M. G. analyzed the data and wrote the paper. All authors discussed the results and commented on the manuscript.

## Conflicts of interest

There are no conflicts to declare.

## Supplementary Material

SC-017-D5SC08213G-s001

SC-017-D5SC08213G-s002

## Data Availability

CCDC 2463809 (for 7a) contains the supplementary crystallographic data for this paper.^70^ The data supporting this article have been included in the main manuscript as well as in the supplementary information (SI). Supplementary information: structures of all the synthesized systems, experimental procedures, theoretical studies, and full chemical, electrochemical, photophysical and crystallographic characterization. See DOI: https://doi.org/10.1039/d5sc08213g.
