# Supplementary material for: Subporphyrazine scaffolds as emerging electron acceptors for long-lived charge separation
Source: Chem Sci. 2026 Jan 19;17(11):5563–75. doi: 10.1039/d5sc08213g (PMC12833611; doi:10.1039/d5sc08213g)
Supplement: SC-017-D5SC08213G-s001 [file SC-017-D5SC08213G-s001.pdf]

Supplementary Information (SI) for Chemical Science.
This journal is © The Royal Society of Chemistry 2026

SUPPORTING INFORMATION

Subporphyrazine Scaffolds as Emerging Electron Acceptors for Long-Lived Charge Separation

Swathi Krishna,[†a] Elena Cañizares-Espada,[†b] David Guzmán,[b] Yifan Bo,[a] Timothy Clark,[c] Tomás Torres,*[b,d,e] Dirk M. Guldi*[a] and M. Salomé Rodríguez-Morgade*[b,d]

[a]*Department of Chemistry and Pharmacy, Profile Center FAU Solar, Interdisciplinary Center for Molecular Materials (ICMM), Friedrich-Alexander-Universität Erlangen-Nürnberg, Egerlandstr. 3, 91058 Erlangen, Germany.*

[b]*Departamento de Química Orgánica, Universidad Autónoma de Madrid, Cantoblanco, 28049 Madrid, Spain.*

[c]*Department of Chemistry and Pharmacy, & Computer-Chemie-Center (CCC) Friedrich-Alexander-Universität Erlangen-Nürnberg, Nägelbachstr. 25, 91052 Erlangen, Germany.*

[d]*Institute for Advanced Research in Chemical Sciences (IAdChem), Universidad Autónoma de Madrid, Cantoblanco, 28049 Madrid, Spain.*

[e]*Instituto Madrileño de Estudios Avanzados (IMDEA)-Nanociencia, C/Faraday 9, Cantoblanco, 28049 Madrid, Spain.*

†*These authors contributed equally to this work.*

CONTENTS

Figure S1. Chemical structures of all compounds discussed in this work S3
1. General Experimental Procedures S4
2. Synthetic procedures and characterization S6
Synthesis of Boronic Acid **10** S6
Synthesis of Boron (III) 4-Pyridyloxy[2,3,7,8,12,13-hexapropylsubporphyrazinato] (**13**) S7
Synthesis of Boron (III) 4-Pyridyloxy[2,3,7,8,12,13-hexa(propylthio)subporphyrazinato] (**14**) S7
Synthesis of Boron(III) Phenoxy[2,3,7,8,12,13-hexa(*(E)*-3-methoxy-3-oxoprop-1-en-1-yl)subporphyrazinato] (**7a**) S8
Synthesis of Boron(III) Phenoxy[2,3,7,8,12,13-hexa(*(E)*-3-ethoxy-3-oxoprop-1-en-1-yl)-subporphyrazinato] (**7b**) S8
Synthesis of Boron(III) Phenoxy[2,3,7,8,12,13-hexa[*(E)*-4-nitrostyryl]subporphyrazinato] (**8**) S9
Synthesis of Ru(CO)Pc-SubPz **1** S9
Synthesis of Ru(CO)Pc-SubPz **2** S10
Synthesis of Ru(CO)Pc-SubPz **3** S10
Synthesis of Ru(CO)Pc-SubPz **4** S11
3. Selected Spectra S12
4. Electrochemical data S33
5. Steady-state absorption and fluorescence data S39
6. Spectroelectrochemical data S42
7. Transient absorption studies S44
8. FRET parameters S68
9. DFT calculations S69
10. Optimized structures S71
11. Crystallographic data S84
12. References S95

Figure S1. Chemical structures of all compounds discussed in this work.

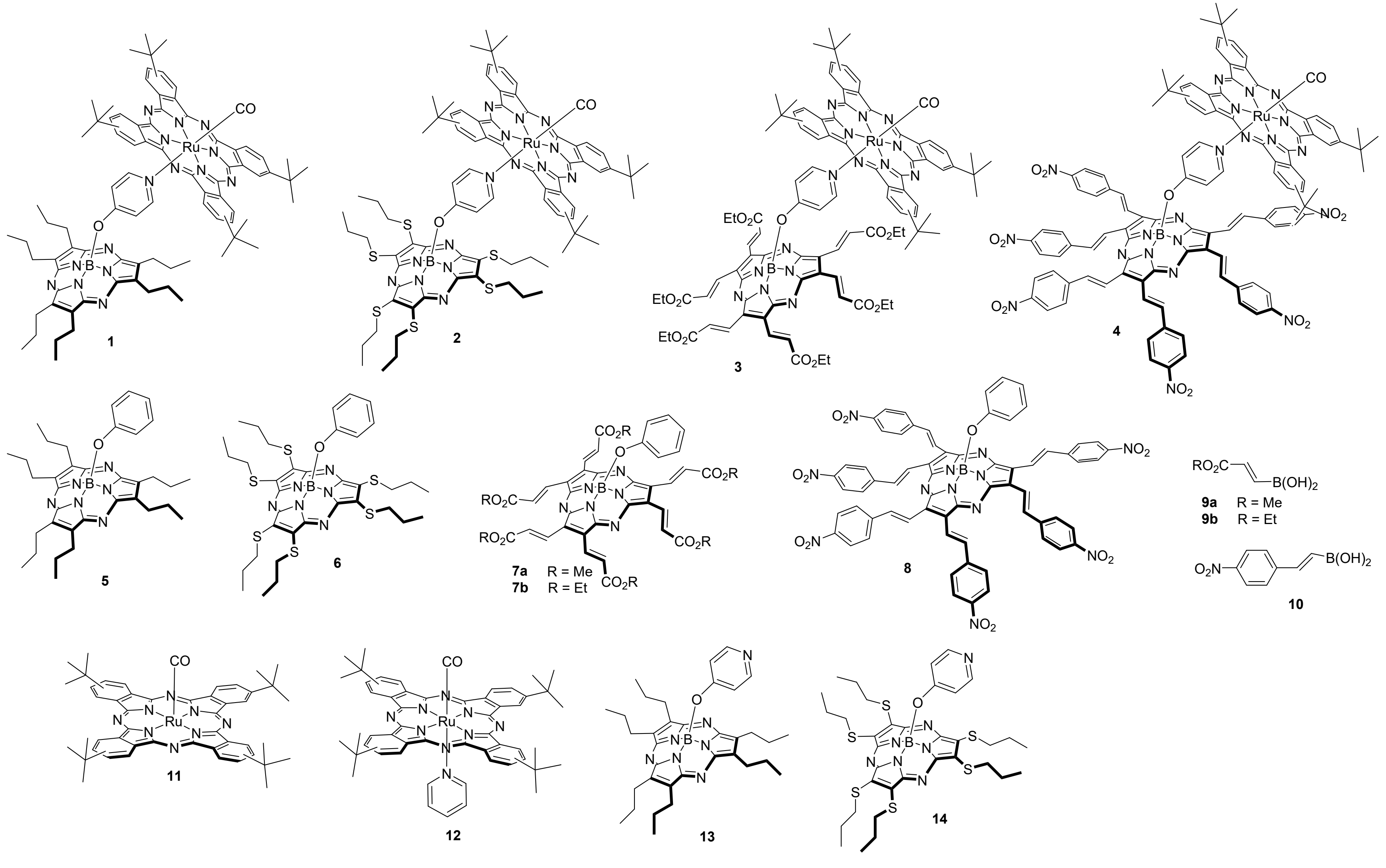

1. General Experimental Procedures.

Synthesis.

All manipulations were carried out under an argon atmosphere. Irradiations were conducted under argon in Hellma quarzglas high performance fluorescence cells (117.100-QS) with 10 mm light path, and in 5 mm diam. NMR tubes. The light sources were monochromatic SMD3528 LED lights, using 520 nm (600 lm) green light and, and 580 nm (310 lm) yellow light, respectively. The distance between the light source and the reaction mixture was of 3.5 cm. Column chromatographies were conducted on silica gel Merck-60 (230-400 mesh, 60 Å), and gel permeation chromatographies were performed on Biobeads SX1. TLC was performed on aluminium sheets pre-coated with silica gel 60 F_{254} (E. Merck). Chemicals were purchased from Aldrich Chemical Co., Alfa Aesar (Thermo Fisher Scientific), and TCI Europe N. V. and used as received without further purification. "Synthetic grade" solvents were used for chemical reactions, column chromatography purifications, and "anhydrous grade" for reactions under dry conditions. Additionally, some solvents were further dried by distillation with sodium/benzophenone (THF) or with solvent purifying system by Innovative Technology Inc. MD-4-PS. Boronic acids **9a,b** were prepared following the reported procedure.[1,2] 2,3-bis(propylthio)maleonitrile[3] and 2,3-dipropylmaleonitrile,[4] and CuTC[3] were prepared following reported procedures.[3,5] SubPzs **5** and **6** were prepared following reported procedures. Ru(CO)Pcs **11** and **12** were prepared using reported procedures.[6,7]

Instrumental Analyses.

^{1}H NMR and **^{13}C NMR** were recorded on Bruker AC-300 (300 MHz) and Bruker AC-400 (400 MHz) spectrometers using as deuterated solvent, $CDCl_3$ or toluene-d_8. The temperature was actively controlled at 298 K. Chemical shifts are measured in ppm relative to tetramethylsilane (TMS).

UV/Vis spectra were recorded with a Jasco V-660-Spectrophotometer, and a Shimadzu UV-1900i UV–vis double beam spectrophotometer.

IR spectra were recorded with Agilent Technologies Cary 630 FTIR, or using a Bruker Alpha II spectrometer by attenuated total reflection (ATR).

Mass spectra (**MS**) were acquired by MALDI-TOF technique in SidI, using a Bruker REFLEX III with a nitrogen laser operating at 337 nm, or using Atmospheric Pressure Chemical Ionization (APCI) as ionization method and using a Q-TOF analyzer.

X-Ray diffraction spectra were done in SidI with a Bruker KAPPA APEX II CCD goniometer with kappa geometry and Mo source (λ= 0.71073 Å). Data were corrected with SADABS program. The intensities were calculated with SAINT program and the structures were resolved with SHELXS and refined with SHELXL.

Fluorescence spectra were recorded using FS5 spectrofluorometer from Edinburgh Instruments. The measurements were performed in the wavelength range of 500 – 900 nm, with a slit width of 5 nm and integration time of 0.1 s. The data were processed in Fluoracle software. All measurements were performed in 10 × 10 mm quartz cuvettes, at room temperature. Absolute fluorescence quantum yield measurements were recorded using the SC-30 Integrating Sphere module with the FS5 spectrofluorometer and calculated in the Fluoracle software.

Singlet oxygen phosphorescence were recorded on Horiba Jobin Yvon FluoroLog3 spectrometer with Symphony II detector in combination with an iHR320 imaging spectrometer. The samples were purged with oxygen for 20 – 30 mins. Singlet oxygen quantum yields were calculated using the relative method, with C60 in air-equilibrated toluene as reference (Φ_Δ^{ref} = 0.98 ± 0.05).[8] Φ_Δ^s was determined using the following equation:

$$\Phi_\Delta^s = \Phi_\Delta^{ref} \cdot \frac{A_{ref}}{A_s} \cdot \frac{E_s}{E_{ref}} \cdot \frac{\tau_{\Delta,\ ref}}{\tau_{\Delta,\ s}} \#(1)$$

where A is the optical density at the excitation wavelength, E is the integrated emission of singlet oxygen signal, and τ_Δ is the singlet oxygen lifetime in the respective solvent. The subscripts 'ref' and 's' refer to the reference and sample, respectively.

Time-correlated single photon counting (TCSPC) measurements were carried out using Horiba Jobin Yvon FluoroLog3 emission spectrometer with Hamamatsu MCP photomultiplier (R33809U-50), to determine the excited state lifetimes. Supercontinuum white light laser from NKT-Photonics was used for excitation. The samples were measured in 10 × 10 mm quartz cuvettes, and purged with argon for 20 mins before each measurement. Data collection and lifetime analyses were performed on DataStation and DAS6 softwares, respectively.

Electrochemistry measurements were performed at room temperature in a potentiostat/galvanostat Autolab PGStat30. Measurements were carried out in a home-built one-compartment cell with a three-electrode configuration, containing 0.1 M tetrabutylammonium hexafluorophosphate ($TBAPF_6$) as supporting electrolyte. A platinum electrode was used as the working electrode, a platinum wire as the counterelectrode, and a $Ag/AgNO_3$ (in CH_3CN) electrode was used as reference. Prior to each voltammetric measurement the cell containing dry DCM or THF was degassed under argon atmosphere for about 10

min. The electrochemical measurements were performed by using a concentration of approximately 0.1-0.2 mmol of the corresponding compound, and ferrocene was added as an internal reference. All the potentials were given relative to the Fc/Fc^+ couple with a scan rate at 100 mV/s.

The energies of charge-separated states were calculated using the continuum model.[9]

$$E_{CSS} = e(E_{ox} - E_{red}) - \frac{e^2}{4\pi\epsilon_s\epsilon_0 R_{DA}} + \frac{e^2}{8\pi\epsilon_0}\left(\frac{1}{r_D} + \frac{1}{r_A}\right)\left(\frac{1}{\epsilon_s} - \frac{1}{\epsilon_s^{'}}\right)\#(2)$$

where E_{ox} and E_{red} refer to the oxidation and reduction potentials, respectively. ϵ_s and $\epsilon_s^{'}$ refer to the dielectric constants of solvent used for time-resolved and electrochemical measurements, respectively. ϵ_s values for THF and DCM are 7.58 and 8.93, respectively. For toluene, due to its quadrupolar rather than dipolar nature, the relative permittivity cannot fully describe the solvent-molecule interactions. Therefore, an apparent permittivity of 3.5 was used.[10,11] r_D and r_A correspond to the spherical radii of the donor and acceptor, respectively, and are determined from the DFT calculations. r_D was estimated to be 6.63 Å, whereas r_A for the Ru(CO)Pc-SubPz conjugates **1**, **2**, **3** and **4** were calculated to be 4.65, 4.72, 7.09 and 8.38 Å, respectively. R_{DA} is the center-to-center donor-acceptor distance. R_{DA} was estimated to be 7.48, 7.48, 7.19 and 7.20 Å for the Ru(CO)Pc-SubPz conjugates **1**, **2**, **3** and **4**, respectively. For ion pairs with R_{DA} at the van-der-Waals limits of ~ 3 Å, the assumption fails, resulting in considerable deviations.[12]

Spectroelectrochemistry measurements were performed using AvaSpec-UV/VIS/NIR two-channel broad band spectrometer from Avantes, containing a balanced deuterium-halogen lamp (AVALIGHT-DH-S-BAL). A three-electrode setup, comprising a platinum gauze as working electrode, a platinum wire as counter electrode and a silver wire as reference electrode, was used. Potentials were applied using FRA 2 µAutolab Type III potentiostat from Metrohm. The measurements were conducted in a thin-layer cuvette with a path length of 1 mm. 0.1 M tetrabutylammonium hexafluorophosphate was used as supporting electrolyte. The data were recorded using Avasoft and NOVA 1.10 softwares.

Femtosecond and nanosecond transient absorption spectroscopy measurements were performed using the pump/probe systems HELIOS (0 to 7500 ps) and EOS (1 ns to 440 µs) from Ultrafast Systems. The laser source was Astrella-F-1K Ti:Sapphire amplifier (800 nm central wavelength, 1 kHz repetition rate, 5.0 W power output, 80 fs pulse width, 5 mJ pulse energy) from Coherent. A fraction of 1.2 mJ of the fundamental is used for pump beam generation via the TOPAS Prime from Light Conversion with standard NirUVis extension. A depolarizer was placed in the pump beam to avoid rotational dynamics. White light probe for femtosecond measurements was generated by focusing a fraction of the fundamental 800 nm output onto a 2 mm sapphire crystal after passing it through a delay line. The white light for the nanosecond transient measurements came from a supercontinuum laser source (fundamental at 1064 nm, 2 kHz repetition rate, 1 ns pulsewidth). For the measurements, the pump energy was varied between 500 and 1000 nJ. Samples were taken in 2 x 10 mm quartz cuvettes and purged with argon for 20 min. The magnetic-field-dependent transient absorption measurements were carried out by coupling the EOS TAPPS detection unit to a type 3480 electromagnet from GMW associates, which is powered by a 1 kW bipolar power supply (BOP-36-28MG; Kepco Inc.). Experiments were performed by increasing the magnetic field strengths of up to 1 T. Samples for measurements under an applied magnetic field were purged with argon and taken in a 2 x 2 mm cuvette. For all measurements, optical densities (OD) of the samples were between 0.2 and 0.4 at the excitation wavelength. Global/target analyses of the resulting data were performed using the GloTarAn software with the R package TIMP.[13,14]

2. Synthetic procedures and characterization

Scheme S1. Synthesis of boronic acid **10**

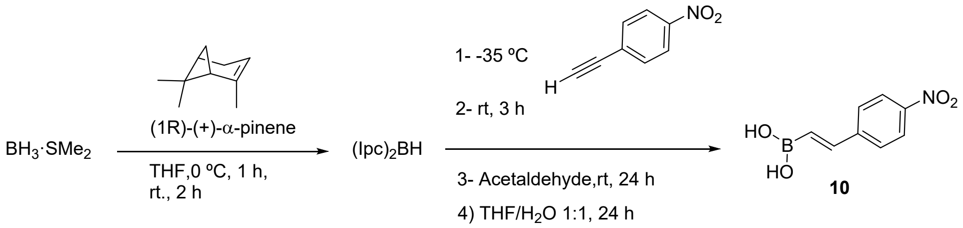

***(E)*-(4-nitrostyryl)boronic acid (10)**

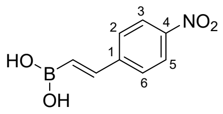

In a 250 mL round bottom flask, borane-dimethyl sulfide (1.70 mL, 18 mmol) was dissolved in anhydrous THF (5 mL) and the mixture was cooled at 0 °C under argon. (*1R*)-(+)-R-pinene (9.72 mL, 61 mmol) were added dropwise and the mixture was stirred at 0 ºC for one hour, followed by stirring at room temperature for two additional hours. After cooling to -35 ºC a solution of 4-ethynylnitrobenzene (2.5 g, 17 mmol) in THF (1 mL) was added and the mixture was stirred at the same temperature for 45 minutes, followed by stirring at room temperature for 3 hours. Acetaldehyde (20 mL, 357 mmol) and the mixture was stirred at room temperature for 16 hours. The mixture was rotary evaporated and a (1:1 v/v) mixture of THF/water (10 mL) was added, followed by stirring at room temperature for 16 hours. The solvent was rotary evaporated and pentane (30 mL) was added to the residue, upon which a white solid precipitated and was washed with pentane, affording **10** (3.21 g, 80 %). ^{1}H NMR (300 MHz, MeOD-d_4, δ ppm): 8.22 (d, *J* = 8.8 Hz, 2H, $H^{3,5}$), 7.75 (d, *J* = 8.5 Hz, 2H, $H^{2,6}$), 7.39 (d, *J* = 17.4 Hz, 1H, =CH), 6.62 (d, *J* = 18.7 Hz, 1H, =CH), 3.35 (s, 2H, $B(OH)_2$). ^{13}C NMR (300 MHz, MeOD-d_4, δ ppm): 148.9, 145.3, 138.8, 135.4, 128.7, 124.9. FT-IR (ATR) υ (cm^{-1}): 3197 (O-H), 2939, 2919 (C=C-H), 1596, 1508, 1340 (C-NO_2), 1193, 1105, 991 (B-O), 820, 743 (NO_2), 548. HRMS (APCI$^-$, DCM): *m/z* calc. for $C_{16}H_{12}N_2O_4$ [2 x M – $B(OH)_2$]$^-$: 296.0797; found 296.0809.

Scheme S2. Synthesis of pyridyloxy-SubPzs **13** and **14**.

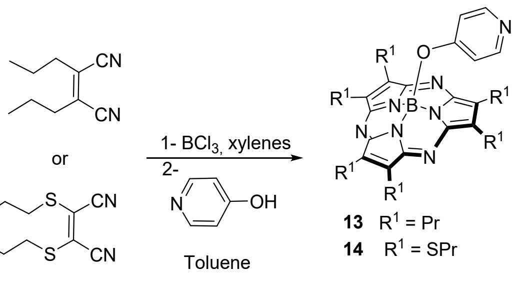

Boron (III) 4-Pyridyloxy[2,3,7,8,12,13-hexapropylsubporphyrazinato] (13)

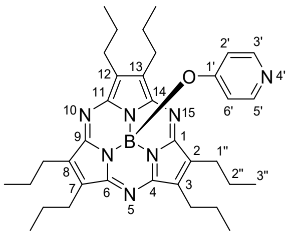

To dipropylmaleonitrile (200 mg, 1.23 mmol) a 1.0 M solution of BCl_3 in *p*-xylene (1.23 mL, 1.23 mmol) was added under Argon and the solution was heated at 135 ºC for 1 hour. The solution was evaporated at reduced pressure and after *in situ* addition of 4-hydroxypyridine (951 mg, 10 mmol), followed by anhydrous toluene (4 mL) the mixture was refluxed for 2 hours. After cooling to room temperature, the toluene solution was filtered through a short Celite path. Column chromatography on silica gel using a (100:1 v/v) mixture of $CHCl_3$/MeOH as eluent, afforded SubPz **13** (57 mg, 23 %) as an orange solid. ^{1}H NMR (300 MHz, $CDCl_3$, δ ppm): 7.88 (d, J = 6.3 Hz, 2H, $H^{3',5'}$), 5.08 (d, J = 6.3 Hz, 2H, $H^{2',6'}$), 3.21-2.99 (m, 12H, $H^{1''}$), 2.10 – 1.94 (m, 12H, $H^{2''}$), 1.18 (t, J = 7.4 Hz, 18H, $H^{3''}$). ^{13}C NMR (75.5 MHz, $CDCl_3$, δ ppm): 149.02, 148.99, 145.66, 128.83, 125.00, 124.53. UV/Vis ($CHCl_3$, λ_{max}/nm, log ε/dm^3 mol^{-1} cm^{-1}): 287 (4.0), 328 (3.8), 500 (4.1). Fluorescence ($CHCl_3$, nm) λ_{ex} = 478; λ_{em} = 529. FT-IR (ATR) υ (cm^{-1}): 2960, 2932 (C=C-H), 2871 (CH_2, CH_3), 1714, 1593 (C=N), 1459, 1298 (C-O), 1161, 1088 (B-O), 1004, 726, 530. MS (MALDI-TOF$^+$, DCTB): *m/z* 1086.7-1091.8 [M][M – OPy]$^+$, 590.4-594.4 [M]$^+$ + [M + H]$^+$, 496.4-499.4 [M – OPy]$^+$.

Boron (III) 4-Pyridyloxy[2,3,7,8,12,13-hexa-(propylthio)subporphyrazinato] (14)

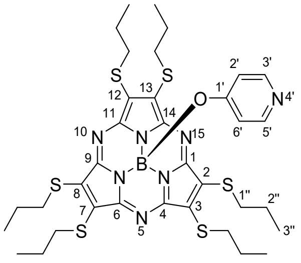

To 2,3-*bis*-(propylsulfanyl)maleonitrile (452 mg, 2.0 mmol) a 1.0 M solution of BCl_3 de (2.0 mL, 2.0 mmol) in *p*-xylene was added under argon and the solution was heated at 135 ºC for 1 h. After evaporation of the mixture at reduced pressure, 4-hydroxypyridine (951 mg, 10 mmol) in anhydrous toluene (4 mL) was added *in* situ and the mixture was refluxed for 2 additional hours. After cooling to room temperature, the toluene solution was filtered through a short path of Celite. The solvent was rotary evaporated and the residue chromatographed on silica gel using a (100:1 v/v) mixture of $CHCl_3$/MeOH as the eluent. Size exclusion chromatography on Biobeads using toluene as the eluent afforded SubPz **14** (23 mg, 4.5 %) as a red solid. ^{1}H NMR (300 MHz, $CDCl_3$, δ ppm): 7.97 (d, J = 3.2 Hz, 2H, $H^{3',5'}$), 5.22 (d, J = 6.0 Hz, 2H, $H^{2'6'}$), 4.16-3.59 (2m, 12H, $H^{1''}$), 2.03-1.90 (m, 12H, $H^{2''}$), 1.18 (t, J = 7.3 Hz, 18H, $H^{3''}$). UV/Vis ($CHCl_3$, λ_{max}/nm, log ε/dm^3 mol^{-1} cm^{-1}): 283 (4.5), 443 (4.6), 558 (4.4). Fluorescence ($CHCl_3$, nm): λ_{ex} = 545; λ_{em} = 585. FT-IR (ATR) υ (cm^{-1}): 2937, 2925 (C=C-H), 2867 (CH_2, CH_3), 1668, 1594 (C=N), 1479, 1448, 1344, 1260 (C-O), 1170, 1109, 1010 (B-O), 813, 748 (C-S), 578, 541. MS (MALDI-TOF$^+$, DCTB): *m/z* = 1470.3-1479.3 [M][M – OPy]$^+$, 782.2-787.2 [M]$^+$ + [M + H]$^+$, 688.2-693.2 [M – OPy]$^+$.

Boron(III) Phenoxy[2,3,7,8,12,13-hexa(*(E)*-3-methoxy-3-oxoprop-1-en-1-yl)subporphyrazinato] (7a)

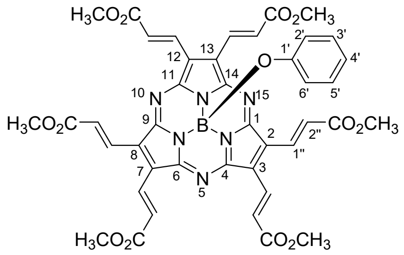

Hexa(propylthio)subporphyrazine **6** (44 mg, 52 μmol), boronic acid **9a** (136 mg, 1.05 mmol), $Pd(PPh_3)_4$ (36 mg, 31 μmol) and CuTC (178 mg, 0.94 mmol) were added into the flask and purged with argon for 10 min, followed by the addition of anhydrous THF (6 mL) *via* cannula. The reaction mixture was stirred at 30 ºC for 20 h under argon atmosphere, cooled to room temperature and passed through a short silica gel column using THF as eluent. The solvent was rotary evaporated and the residue was purified by silica gel column chromatography (mixture of *n*-hexane/CH_2Cl_2/ethyl acetate 4:4:2) giving pure SubPz **7a** (16.6 mg, 38%) as a blue solid. ^{1}H NMR (400 MHz, $CDCl_3$, δ ppm): 8.14 (d, J = 16.0 Hz, 6H, $H^{2''}$), 7.98 (d, J = 16.0 Hz, 6H, $H^{1''}$), 6.86 (t, J = 8.0 Hz, 2H, $H^{3',5'}$), 6.73 (t, J = 8.0 Hz, 1H, $H^{4'}$), 5.41 (d, J = 8.0 Hz, 2H, $H^{2',6'}$), 3.94 ppm (s, 18H; -OMe). ^{13}C NMR (100.6 MHz, $CDCl_3$, δ ppm): 166.6, 155.5, 151.8, 131.6, 131.0, 129.3, 127.9, 122.2, 118.7, 52.2. UV/Vis ($CHCl_3$, λ_{max}/nm, log ε/dm^3 mol^{-1} cm^{-1}): 280 (4.7), 426 (4.3), 600 (4.6). Fluorescence ($CHCl_3$, nm): λ_{ex} = 500; λ_{em} = 622. FT-IR (ATR) υ (cm^{-1}): 2951, 2925, 2854, 1715, 1623, 1502, 1460, 1434, 1260, 1173, 1068, 975, 871, 733 cm^{-1}. HRMS (APCI, MeOH, +): *m/z* calc. for $C_{42}H_{35}BN_6O_{13}$ $[M]^+$: 841.2391; found: 841.2285.

XRD: Single crystals suitable for X-ray diffraction analysis were obtained by vapor diffusion of isooctane into its 1,2-dichloroethane solution. Crystallographic data and some refining details are summarized on Tables S1-S8.

Boron(III) Phenoxy[2,3,7,8,12,13-hexa(*(E)*-3-ethoxy-3-oxoprop-1-en-1-yl)-subporphyrazinato] (7b)

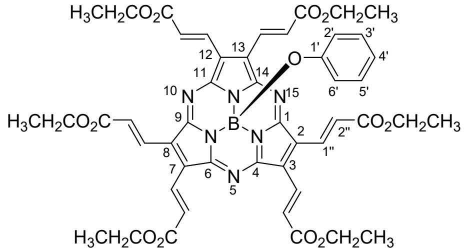

A solution of SubPz **6** (10 mg, 12.6 μmol), boronic acid **9b** (29.8 mg, 229 μmol), $Pd(PPh_3)_4$ (9 mg, 7.7 μmol) and CuTC (43.8 mg, 229 μmol) in anhydrous THF (6 mL) under argon was stirred at 30 ºC for 20 h protected from light. The mixture was allowed to reach room temperature and then, passed through a short silica gel column using THF as eluent. The solvent was rotary evaporated and the residue was purified by silica gel column chromatography (mixture of *n*-heptane/ethyl acetate 2:1). The product was subjected to size exclusion chromatography on Biobeads using toluene as the eluent affording 8 mg of SubPz **7b** (68 %) as a blue solid. ^{1}H NMR (300 MHz, $CDCl_3$, δ ppm): 8.14 (d, J = 16.0 Hz, 6H, $H^{2''}$), 7.98 (d, J = 16.0 Hz, 6H, $H^{1''}$), 6.86 (t, J = 8.0 Hz, 2H, $H^{3',5'}$), 6.73 (t, J = 8.0 Hz, 1H, $H^{4'}$), 5.41 (d, J = 8.0 Hz, 2H, $H^{2',6'}$), 4.40 (m, 12H, OCH_2CH$_3$), 1.44 (t, J = 7.2 Hz, OCH$_2$$CH_3$). UV/Vis ($CHCl_3$, λ_{max}/nm, log ε/dm^3 mol^{-1} cm^{-1}): 290 (4.7), 436 (4.3), 603 (4.6). Fluorescence ($CHCl_3$, nm): λ_{ex} = 580; λ_{em} = 620. FT-IR (ATR) υ (cm^{-1}): 2942, 2925, 2851, 1710, 1622, 1462, 1367, 1249, 1158, 1030, 974, 871, 727. MS (MALDI-TOF$^+$, DCTB+NaI): *m/z* = 948.3-951.3 $[M + Na]^+$, 971.3-974.3 $[M + 2Na]^+$. HRMS (MALDI-TOF$^+$, DCTB + PPGNa 1000 + NaI): *m/z* calc. for $C_{48}H_{49}BN_6NaO_{13}$ $[M + Na]^+$: 948.3223; found 948.3185.

Boron(III) Phenoxy[2,3,7,8,12,13-hexa[*(E)*-4-nitrostyryl]subporphyrazinato] (8)

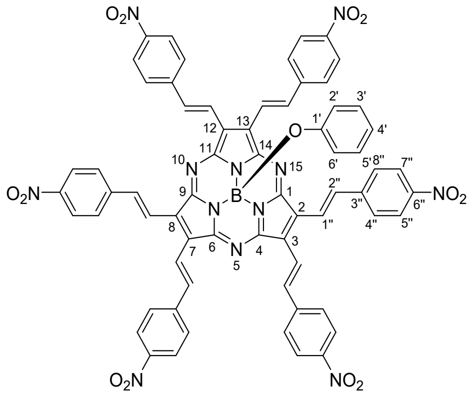

A solution of SubPz **6** (18 mg, 17.4 µmol), boronic acid **10** (62.4 mg, 322 µmol), $Pd(PPh_3)_4$ (11.1 mg, 10.8 µmol) and CuTC CuTC (55.6 mg, 320 µmol) in anhydrous THF (6 mL) under argon was stirred at 30 ºC for 2 h protected from light. The mixture was allowed to reach room temperature and then, passed through a short Celite column. The solvent was rotary evaporated, the residue was dissolved in CHCl3 and precipitated by addition of hexane (10:1 v/v of $CHCl_3$/hexane). The solid was chromatographed on Biobeads using THF as the eluent affording 12 mg of SubPz **8** (43 %) as a green solid. ^{1}H NMR (300 MHz, THF-d_8, δ ppm): 9.15 (d, J = 16.2 Hz, 6H, $H^{1''}$), 8.39 (d, J = 8.7 Hz, 12H, $H^{5'',7''}$), 8.29 (d, J = 16.2 Hz, 6H, $H^{2''}$), 8.16 (d, J = 8.8 Hz, 12H, $H^{4'',8''}$), 6.88-6.81 (m, 1H, $H^{4'}$), 6.60 (d, J = 7.7 Hz, 2H, $H^{3',5'}$), 5.08 (d, J = 7.0 Hz, 2H, $H^{2',6'}$). UV/Vis ($CHCl_3$, λ_{max}/nm, log ε/dm^3 mol^{-1} cm^{-1}): 354 (4.0), 398 (3.9), 499 (3.7), 642 (3.7). Fluorescence ($CHCl_3$, nm): λ_{ex} = 625; λ_{em} = 665. FT-IR (ATR) υ (cm^{-1}): 3057, 2923, 2840, 1591, 1510, 1430, 1352, 1337, 1077, 977, 967, 845, 746, 700, 523. MS (MALDI-TOF$^-$, DCTB): *m/z* 1219.3-1223 [M]$^-$. HRMS (MALDI-TOF$^-$), DCTB + PPGNa 1000 + NaI): *m/z* calc. for $C_{66}H_{41}BN_{12}O_{13}$ [M]$^-$: 1219.3051; found 1219.3056.

Ru(CO)Pc-SubPz 1

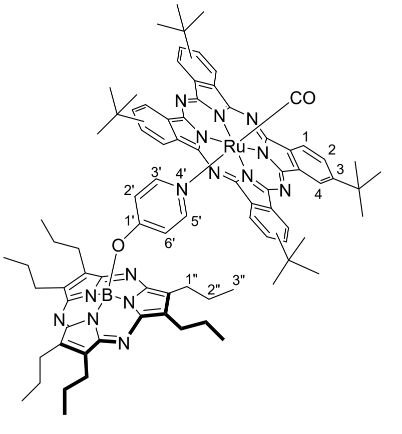

A deaerated solution of SubPz **13** (10 mg, 16.8 µmol and Ru(CO)Pc **11** (14.5 mg, 16.8 µmol) in anhydrous toluene (8 mL) was stirred at room temperature under argon and protected from light for 16 h. After rotary evaporation of the mixture, the residue was subjected to column chromatography on silica gel using (3:1) mixture of heptane/AcOEt. Further purification by gel permeation chromatography on Biobeads using toluene as the eluent afforded Ru(CO)Pc-SubPz **1** (20 mg, 81 %) as a blue solid. ^{1}H NMR (300 MHz, $CDCl_3$, δ ppm): 9.4-9.1 (m, 8H, $H^{1,4}$), 8.10 (d, J = 8.1 Hz, 4H, $H^{2,3}$), 2.90 (d, J = 7.1 Hz, 2H, $H^{2',6'}$), 2.79 (t, J = 7.0 Hz, 12H, $H^{1''}$), 1.77 (m, 36H, *t*-Bu), 1.73-1.60 (m, 12H, $H^{2''}$), 1.29 (d, J = 7.1 Hz, 2H, $H^{3',5'}$), 0.89 (t, J = 7.3 Hz, 18H, $H^{3''}$). UV/Vis ($CHCl_3$, λ_{max}/nm, log ε/dm^3 mol^{-1} cm^{-1}): 292 (3.8), 306 (3.8), 333 (3.5), 350 (3.3), 503 (3.2), 591 (3.3), 652 (4.0). FT-IR (ATR) υ (cm^{-1}): 3062, 2958, 2928, 2868, 1970 (C=O), 1613, 1456, 1323, 1256, 1119, 1089, 983, 828, 761, 696, 529. HRMS (ESI$^+$, MeOH + 0.1% formic acid) *m/z* 1452.6953-1462.7001 [M$^+$] + [M + H]$^+$; calc. for $C_{84}H_{95}BN_{15}O_2{}^{96}Ru$ [M + H]: 1452.6070; Found: 1452.6953.

Ru(CO)Pc-SubPz 2

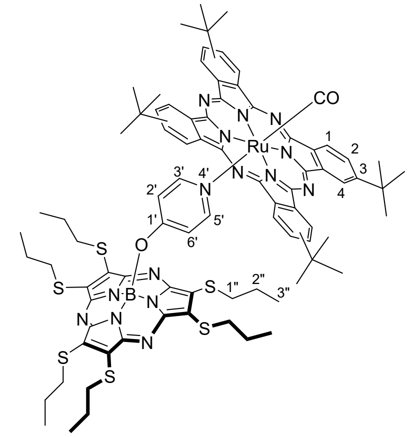

A deaerated solution of SubPz **14** (20 mg, 25.5 μmol) and Ru(CO)Pc **11** (22.1 mg, 25.5 μmol) in anhydrous toluene (8 mL) was stirred at room temperature under argon and protected from light for 16 h. After rotary evaporation of the mixture, the residue was subjected to column chromatography on silica gel using (3:1) mixture of heptane/AcOEt. Further purification by gel permeation chromatography on Biobeads using toluene as the eluent afforded Ru(CO)Pc-SubPz **2** (30 mg, 72 %) as a blue solid. ^{1}H NMR (300 MHz, $CDCl_3$, δ ppm): 9.4 - 9.1 (m, 8H, $H^{1,4}$), 8.10 (d, *J* = 8.2 Hz, 4H, $H^{2,3}$), 3.71-3.22 (2m, 12H, $H^{1''}$), 3.02 (d, *J* = 7.1 Hz, 2H, $H^{2',6'}$), 1.78-1.76 (m, 36H, *t*-Bu), 1.36 (d, *J* = 7.1 Hz, 2H, $H^{3',5'}$), 1.20 (t, *J* = 7.3 Hz, 12H, $H^{2''}$), 0.96 (t, *J* = 7.3 Hz, 18H, $H^{3''}$). UV/Vis ($CHCl_3$, λ_{max}/nm, log ε/dm^3 mol^{-1} cm^{-1}): 304 (4.8), 350 (4.4), 448 (4.3), 565 (4.3), 590 (4.4), 652 (5.0). FT-IR (ATR) υ (cm^{-1}): 3074, 2958, 2924, 2865, 1969 (C=O), 1612, 1487, 1316, 1257, 1123, 1053, 989, 830, 752, 670, 576, 531. HRMS (ESI$^+$, MeOH + 0.05% formic acid) *m/z* 1643.5234-1657.5249 [M$^+$] + [M + H]$^+$; calc. for $C_{84}H_{94}BN_{15}O_2{}^{96}RuS_6$ [M$^+$]: 1643.5317; Found: 1643.5234.

Ru(CO)Pc-SubPz 3

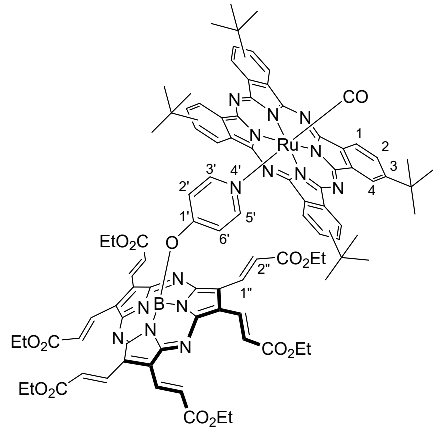

A deaerated solution of Ru(CO)Pc-SubPz **2** (30 mg, 18.2 μmol) the boronic acid **9b** (51.8 mg, 0.36 mmol), $Pd(PPh_3)_4$ (12.6 mg, 10.9 μmol), CuTC (62.3 mg, 0.33 mmol) in anhydrous THF (6 mL) under argon and protected from light was stirred at 30 ºC for 1 h. After cooling to room temperature, the mixture was passed through a short pad of Celite. The solution was rotary evaporated and the residue was subjected to column chromatography on silica gel using (3:2) mixture of heptane/AcOEt, affording Ru(CO)Pc-SubPz **3** (13 mg, 40 %) as a turquoise blue solid. ^{1}H NMR (300 MHz, $CDCl_3$, δ ppm): 9.35-9.17 (m, 8H, $H^{1,4}$), 8.11-8.09 (m, 4H, $H^{2,3}$), 7.87 (d, *J* = 16.0 Hz, 6H, $H^{1''}$), 7.64 (d, *J* = 16.0 Hz, 6H, $H^{2''}$), 4.26-4.19 (m, 12H, O*CH₂*CH₃), 3.15 (d, *J* = 6.0 Hz, 2H, $H^{2',6'}$), 1.77-1.76 (3s, 36H, *t*-Bu), 1.3-1.4 (m, 18H, $OCH_2\mathit{CH_3}$). UV/Vis ($CHCl_3$, λ_{max}/nm, log ε/dm^3 mol^{-1} cm^{-1}): 298 (4.0), 342 (3.6), 433 (3.1), 548 (3.2), 594 (3.7), 652 (4.0). FT-IR (ATR) υ (cm^{-1}): 3052), 2958, 2864, 1971 (C=O), 1713 (O-C=O), (C=N), 1439, 1434, 1281), 1183, 1095, 744, 692, 499. MS (MALDI-TOF$^+$, DCTB): *m/z* = 1787.5-1800.5 [M$^+$], 1759.5-1769.5 [M – CO]$^+$, 927.3-929.3 [M – Ru(CO)Pc + H]$^+$. HRMS (MALDI-TOF$^+$, DCTB)

m/z 1759.6230-1769.6475 [M – CO]$^+$, calc. for $C_{95}H_{94}BN_{15}O_{13}{}^{96}Ru$ [M$^+$]: 1759.6333; Found: 1759.6230; 949.3123-952.3222 [M – Ru(CO)Pc + Na]$^+$, calc. for $C_{47}H_{46}BN_7NaO_{13}$ [M – Ru(CO)Pc + Na]$^+$: 949.3175; Found: 949.3123.

Ru(CO)Pc-SubPz 4

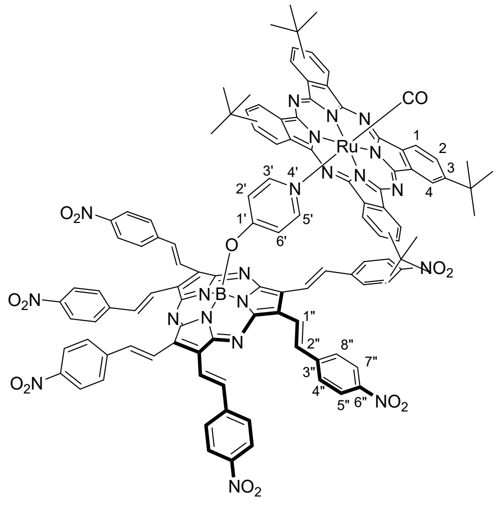

A deaerated solution of Ru(CO)Pc-SubPz **2** (25 mg, 15.1 μmol) the boronic acid **10** (57.9 mg, 0.30 mmol), $Pd(PPh_3)_4$ (10.5 mg, 9.08 μmol), CuTC (51.9 mg, 0.27 mmol) in anhydrous THF (6 mL) under argon and protected from light was stirred at 30 ºC for 1.5 h. After cooling to room temperature, the mixture was passed through a short pad of Celite. The solution was rotary evaporated and the residue was subjected to size exclusion chromatography on Biobeads using THF as the eluent, affording Ru(CO)Pc-SubPz **4** (10 mg, 32 %) as a dark green solid. ^{1}H NMR (300 MHz, $CDCl_3$, δ ppm): 9.3-9.2 (m, 14H, H$^{1,4,1''}$), 8.3-8.2 (m, 6H, H$^{2''}$), 8.06 (d, *J* = 7.2 Hz, 4H, H^{2,3}), 7.7-7.4 (m, 24H, H$^{4'',5'',7'',8''}$), 3.76 (d, *J* = 7.2 Hz, 2H, H$^{2',6'}$), 1.70 (broad s, 36H, *t*-Bu), 1.3-1.2 (m, 2H, H$^{3',5'}$). UV/Vis ($CHCl_3$, λ_{max}/nm, log ε/dm^3 mol^{-1} cm^{-1}): 305 (3.9), 333 (3.7), 350 (3.7), 400 (3.4), 485 (3.3), 592 (3.5), 652 (4.0). FT-IR (ATR) υ (cm^{-1}): 3055, 2959, 2926, 2867, 1955 (C=O), 1589, 1515, 1430, 1337 (C-NO_2), 1098, 1025, 823, 750, 688, 524. MS (MALDI-TOF$^-$, DCTB): 1220.3-1225.3 [M – Ru(CO)Pc]$^-$; MS (MALDI-TOF$^+$, DCTB): *m/z* = 860.3-871.4 [M – (SubPz-OPy)]$^+$.

3. Selected Spectra

Figure S2. ^{1}H NMR spectrum of boronic acid **10** in CD_3OD.

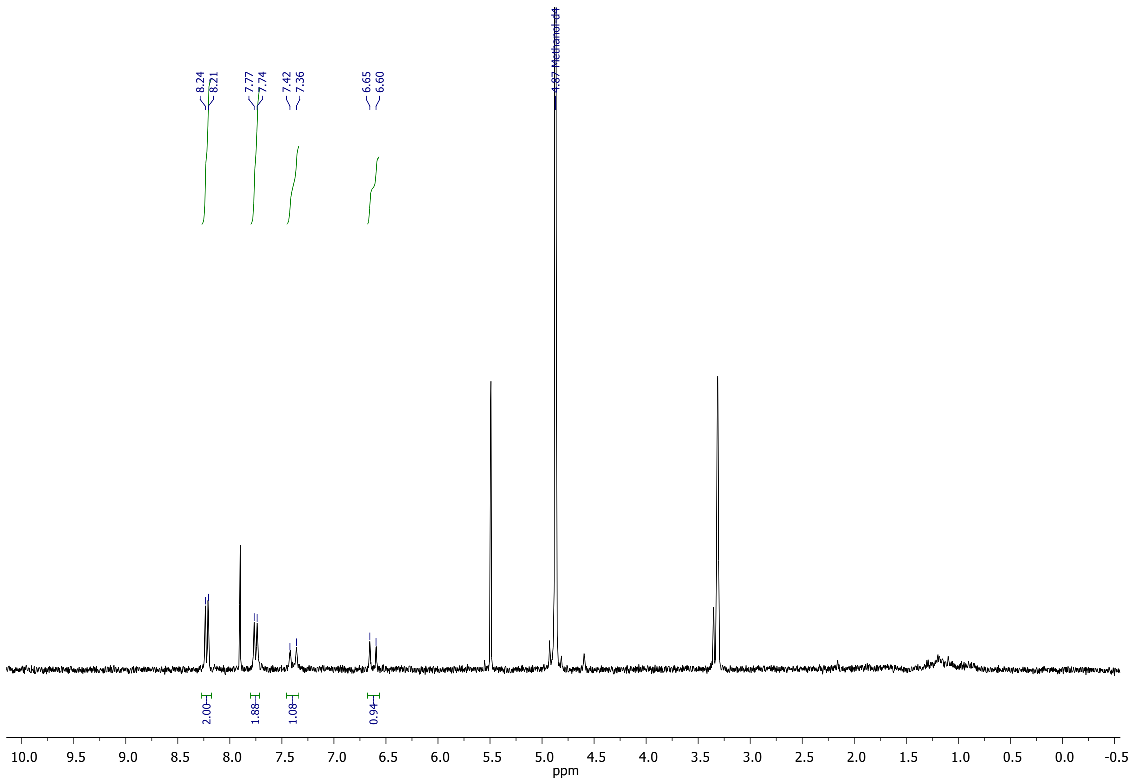

Figure S3. ^{13}C NMR spectrum of **10** in CD_3OD.

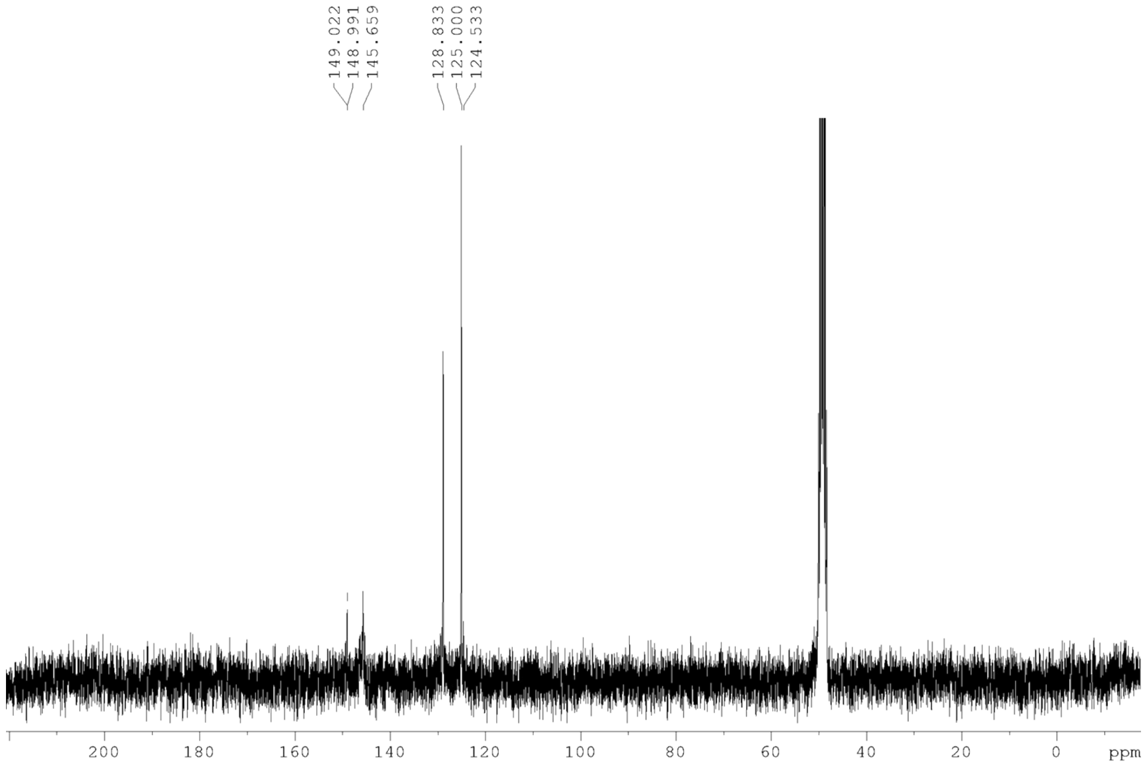

Figure S4. HRMS spectrum (APCI⁻) of **10**.

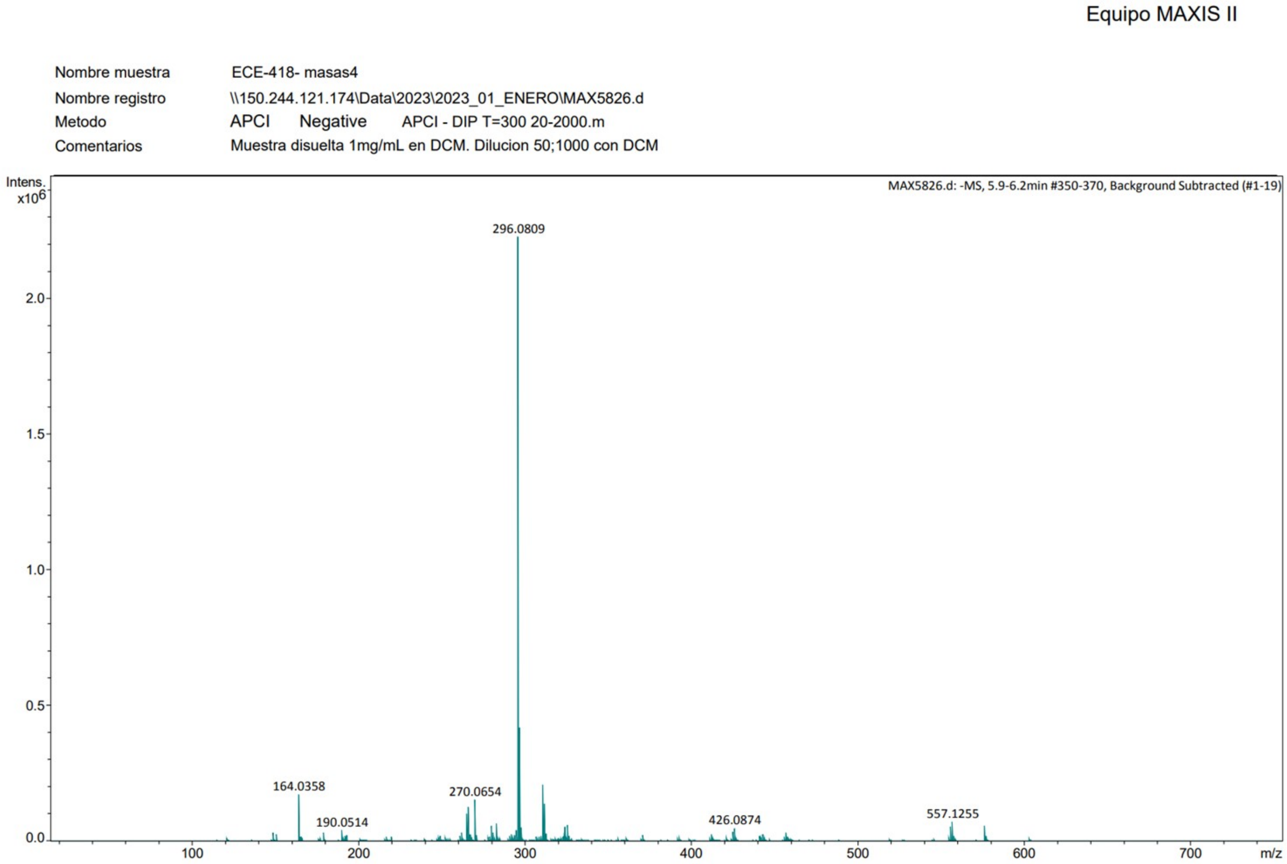

Figure S5. ^{1}H NMR spectrum of SubPz **13** in $CDCl_3$.

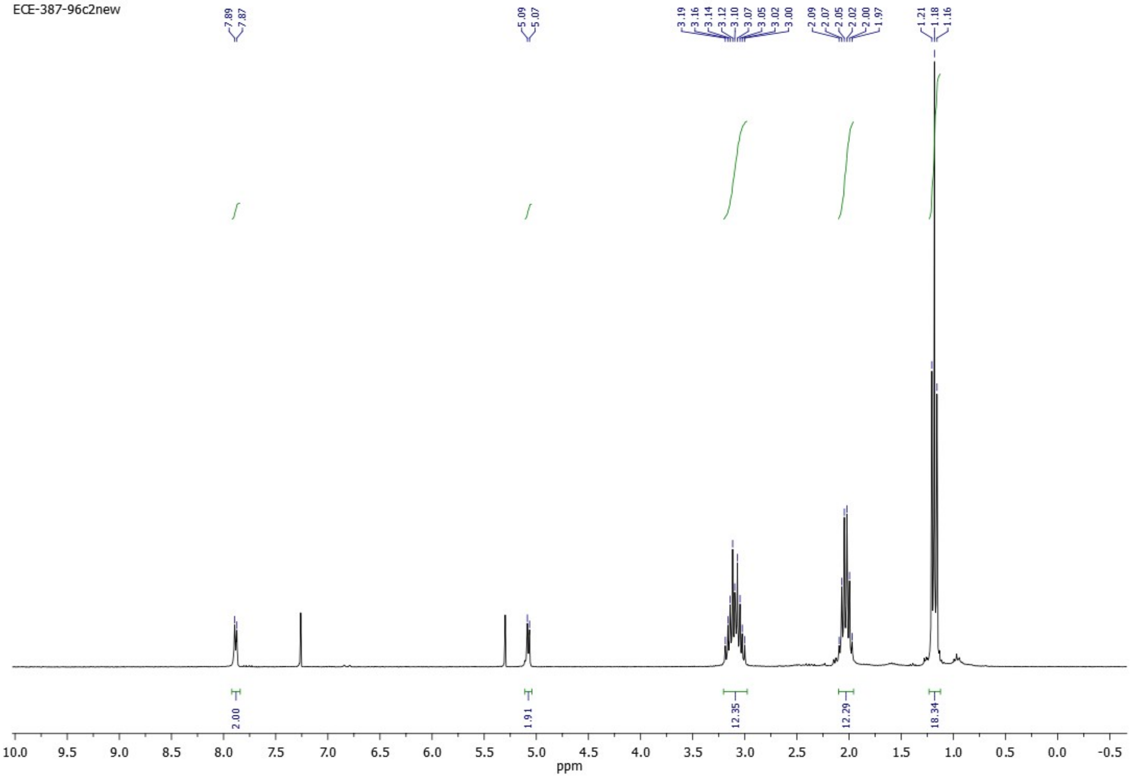

Figure S6. MS spectra (MALDI-TOF) of **13** showing the experimental isotopic patterns compared to the expected clusters (Upper: found; Lower: calculated).

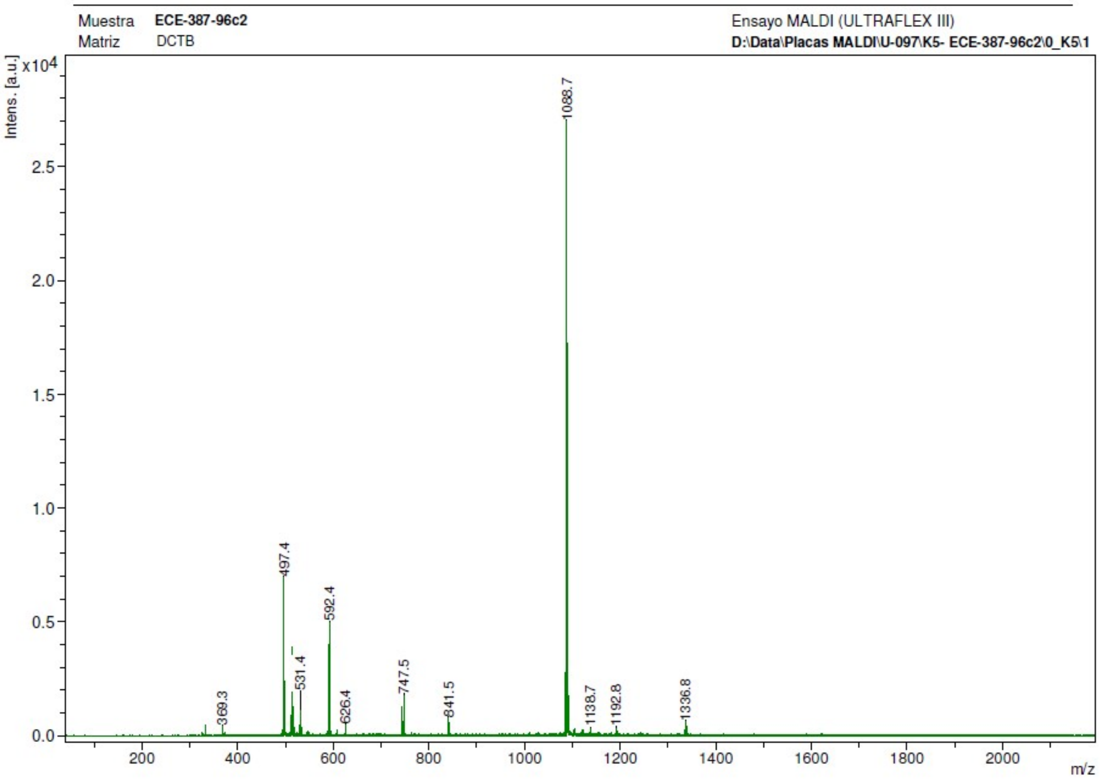

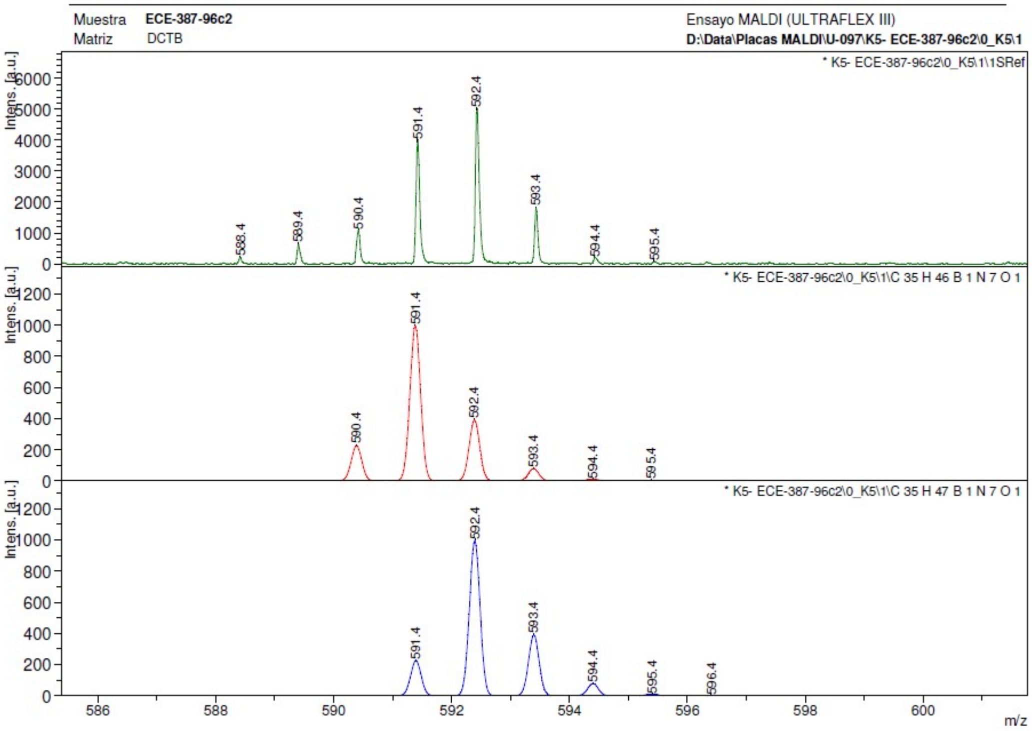

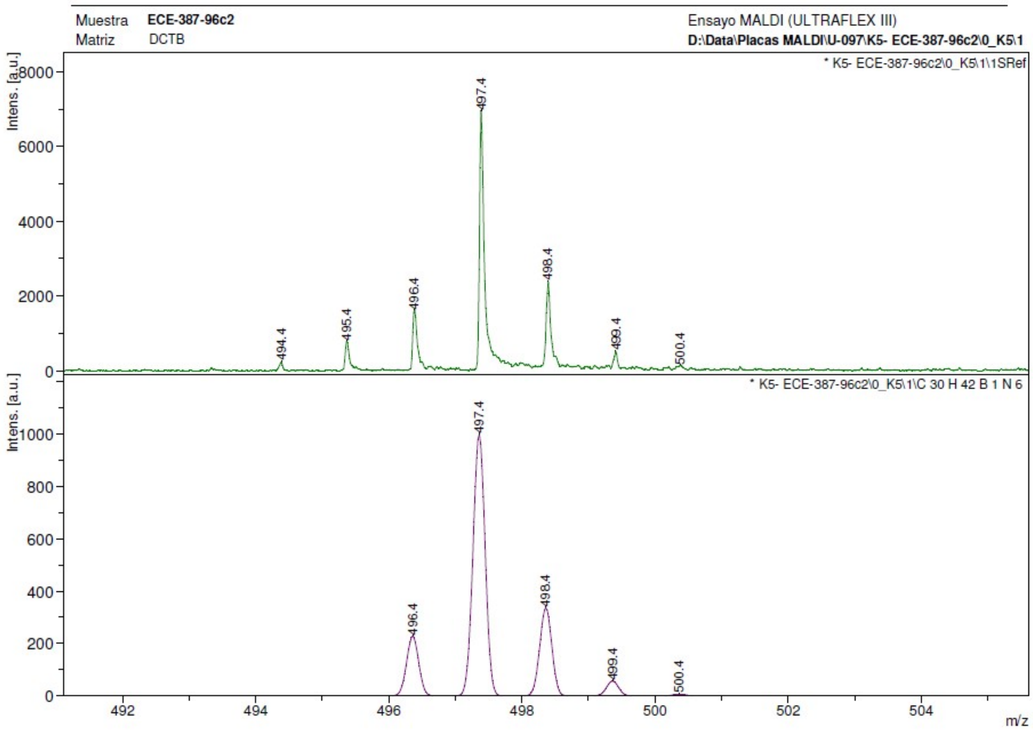
Muestra ECE-387-96c2
Matriz DCTB
Ensayo MALDI (ULTRAFLEX III)
D:\Data\Placas MALDI\U-097\K5- ECE-387-96c2\0_K5\1
* K5- ECE-387-96c2\0_K5\1\1SRef
* K5- ECE-387-96c2\0_K5\1\C 30 H 42 B 1 N 6
Intens. [a.u.]
m/z
494.4
495.4
496.4
497.4
498.4
499.4
500.4

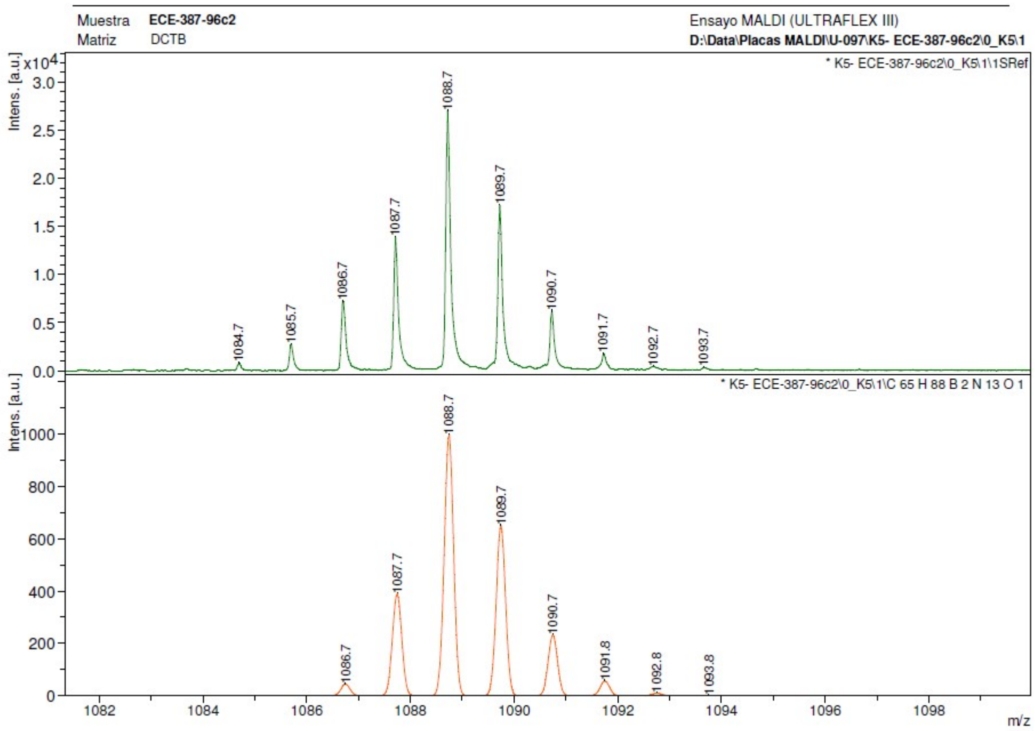
Muestra ECE-387-96c2
Matriz DCTB
Ensayo MALDI (ULTRAFLEX III)
D:\Data\Placas MALDI\U-097\K5- ECE-387-96c2\0_K5\1
* K5- ECE-387-96c2\0_K5\1\1SRef
* K5- ECE-387-96c2\0_K5\1\C 65 H 88 B 2 N 13 O 1
Intens. [a.u.]
x10^4
m/z
1084.7
1085.7
1086.7
1087.7
1088.7
1089.7
1090.7
1091.7
1092.7
1093.7
1091.8
1092.8
1093.8

Figure S7. ^1H NMR spectrum ($CDCl_3$) of **14**.

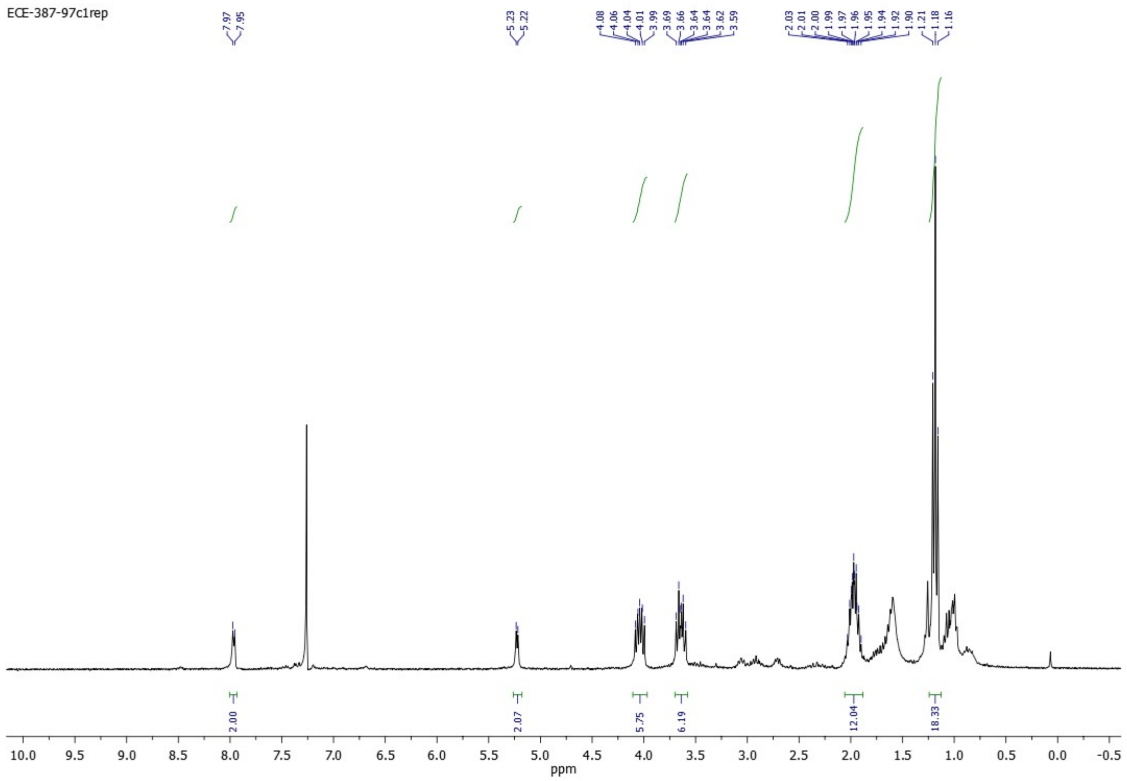

Figure S8. MS spectra (MALDI-TOF) of **14** showing the experimental isotopic patterns compared to the expected clusters (Upper: found; Lower: calculated).

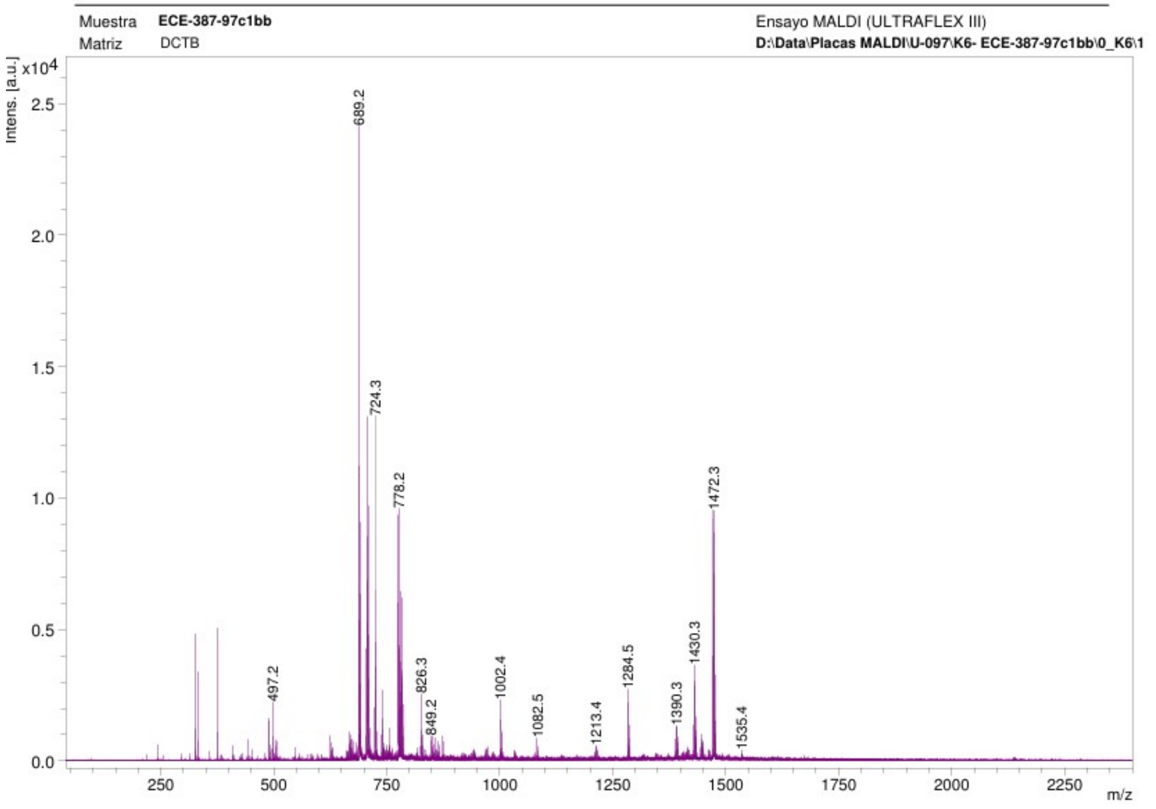

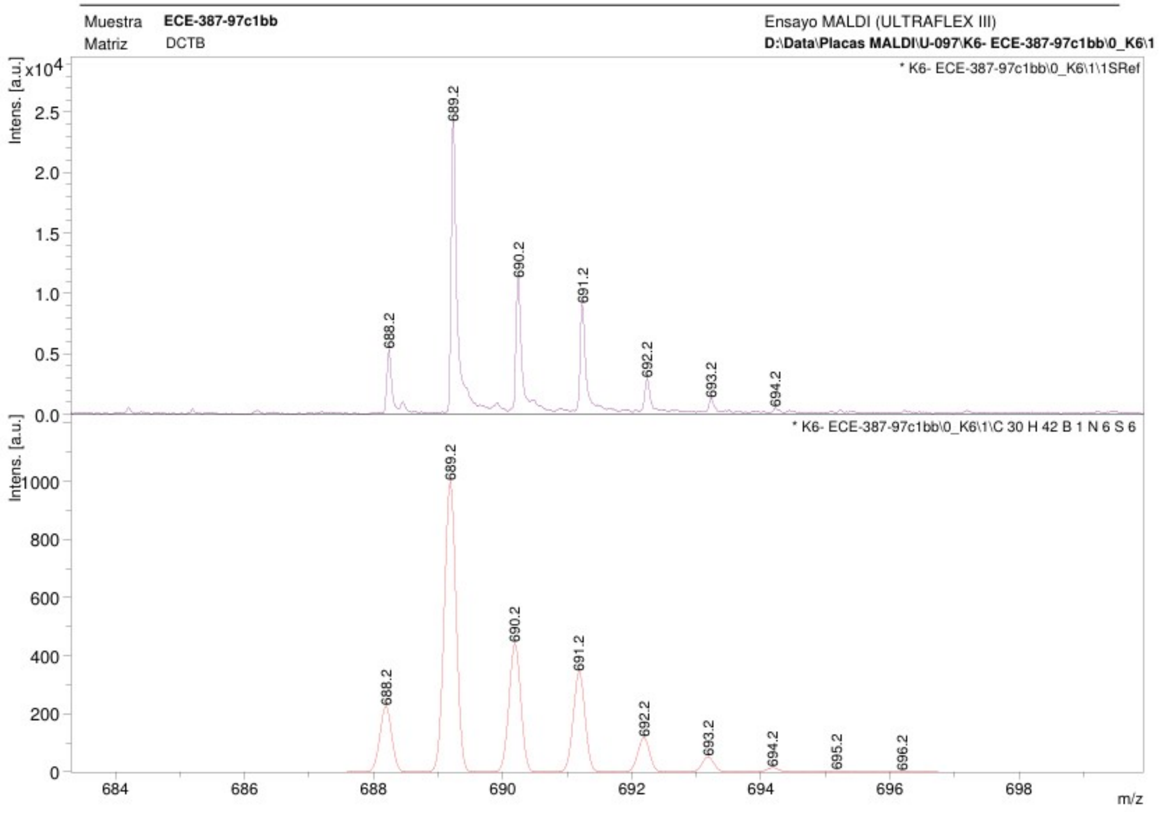

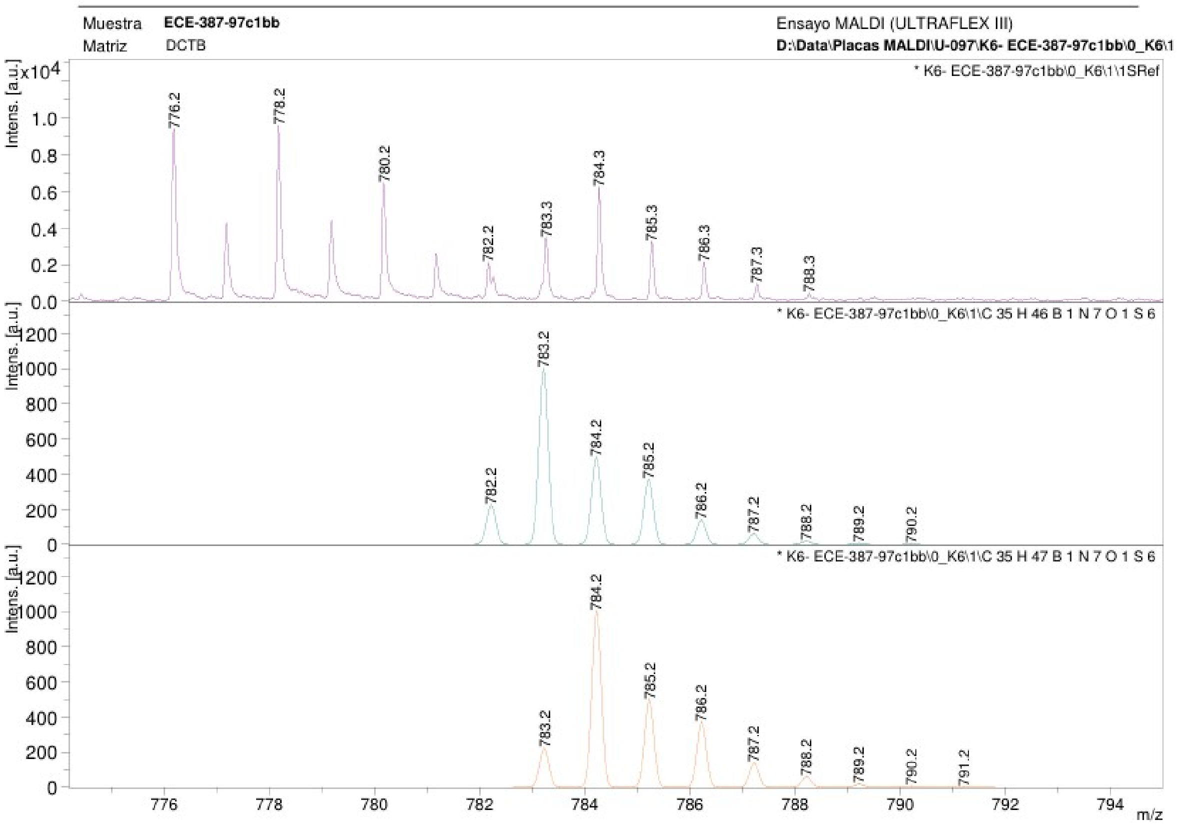

Muestra ECE-387-97c1bb
Matriz DCTB
Ensayo MALDI (ULTRAFLEX III)
D:\Data\Placas MALDI\U-097\K6- ECE-387-97c1bb\0_K6\1
* K6- ECE-387-97c1bb\0_K6\1\1SRef
* K6- ECE-387-97c1bb\0_K6\1\C 35 H 46 B 1 N 7 O 1 S 6
* K6- ECE-387-97c1bb\0_K6\1\C 35 H 47 B 1 N 7 O 1 S 6
Intens. [a.u.]
m/z

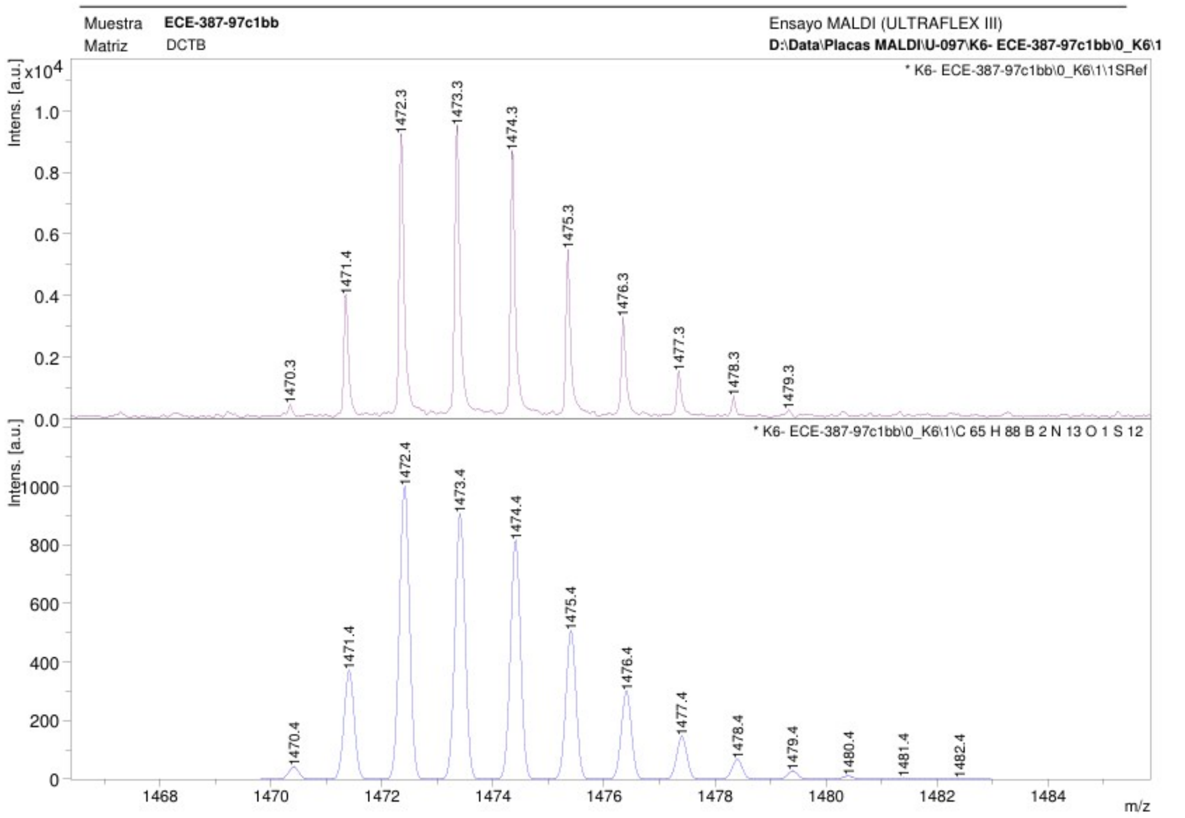

Muestra ECE-387-97c1bb
Matriz DCTB
Ensayo MALDI (ULTRAFLEX III)
D:\Data\Placas MALDI\U-097\K6- ECE-387-97c1bb\0_K6\1
* K6- ECE-387-97c1bb\0_K6\1\1SRef
* K6- ECE-387-97c1bb\0_K6\1\C 65 H 88 B 2 N 13 O 1 S 12
Intens. [a.u.]
m/z

Figure S9. UV/Vis absorption spectrum of SubPz **14** in $CHCl_3$.

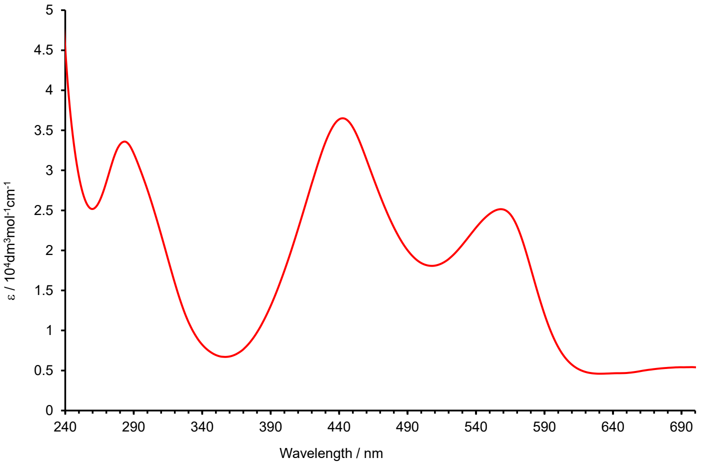

Figure S10. ^{1}H NMR spectrum of SubPz **7a** in $CDCl_3$.

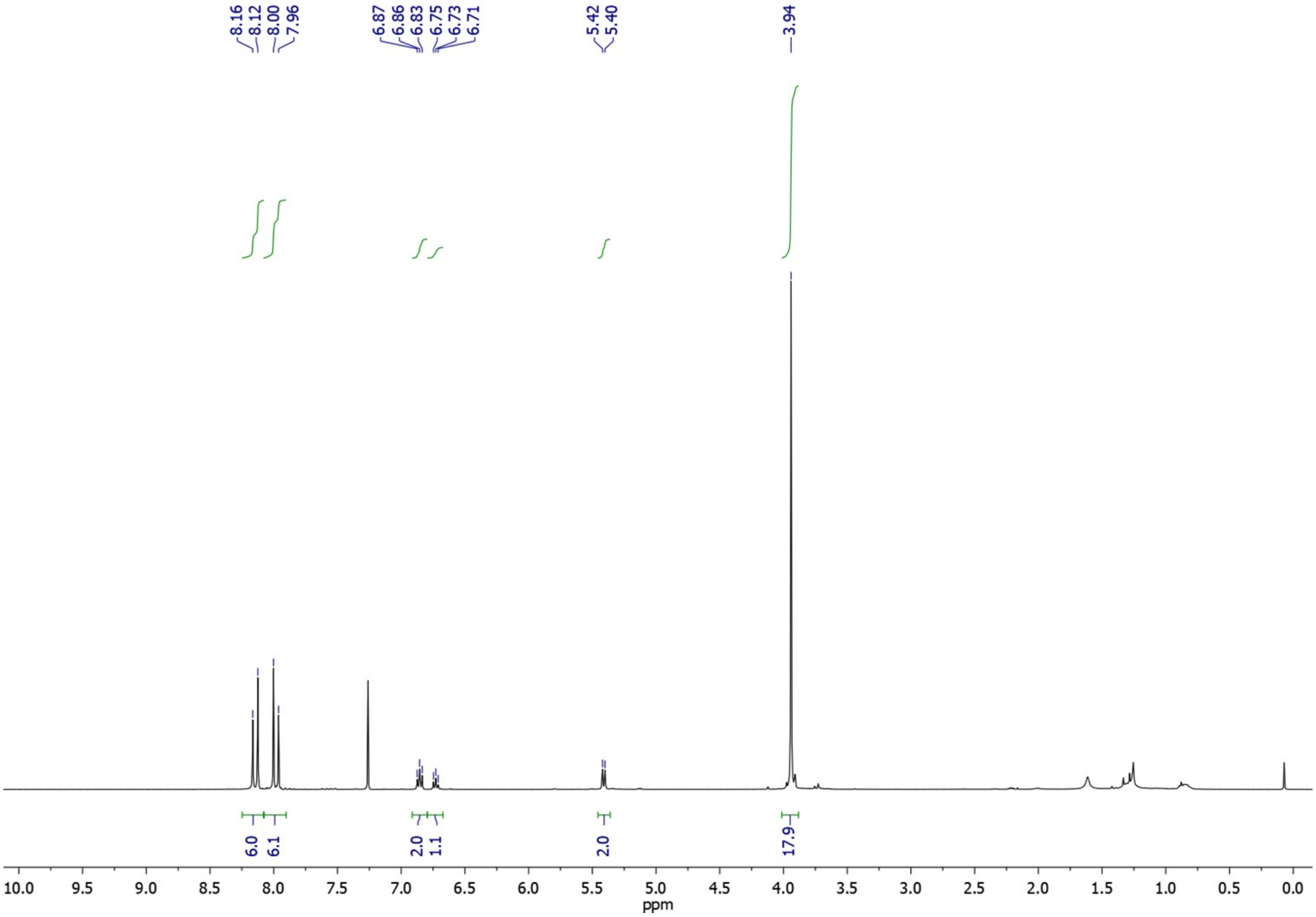

Figure S11.

a) UV/Vis absorption spectrum of SubPz **7a** in $CHCl_3$. **b)** Normalized UV-Vis absorption (solid line) and fluorescence emission (dotted line) spectra of **7a** in $CHCl_3$.

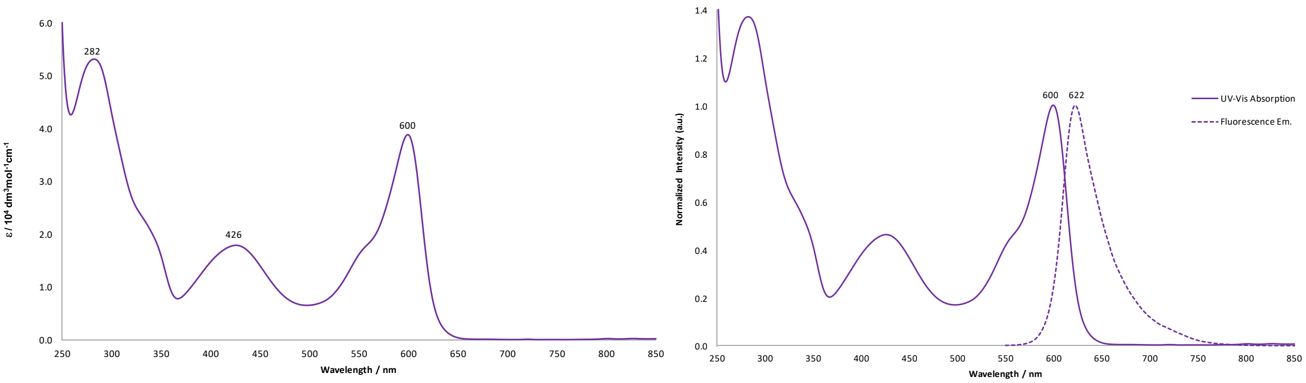

Figure S12. MS and HRMS spectra ($APCI^+$) of **7a** showing the experimental isotopic patterns compared to the expected clusters (Upper: found; Lower: calculated).

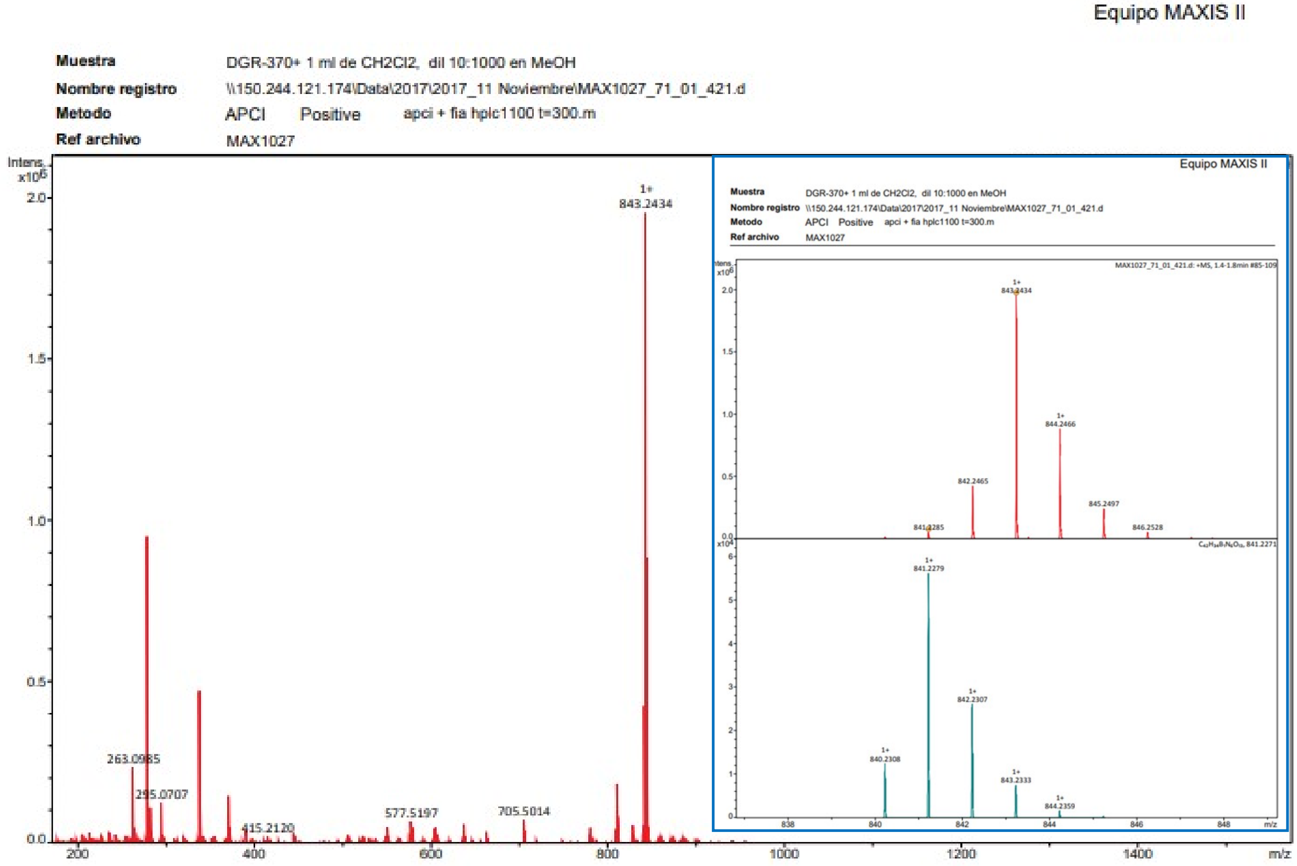

Figure S13. ^{1}H NMR spectrum of **7b** in $CDCl_3$.

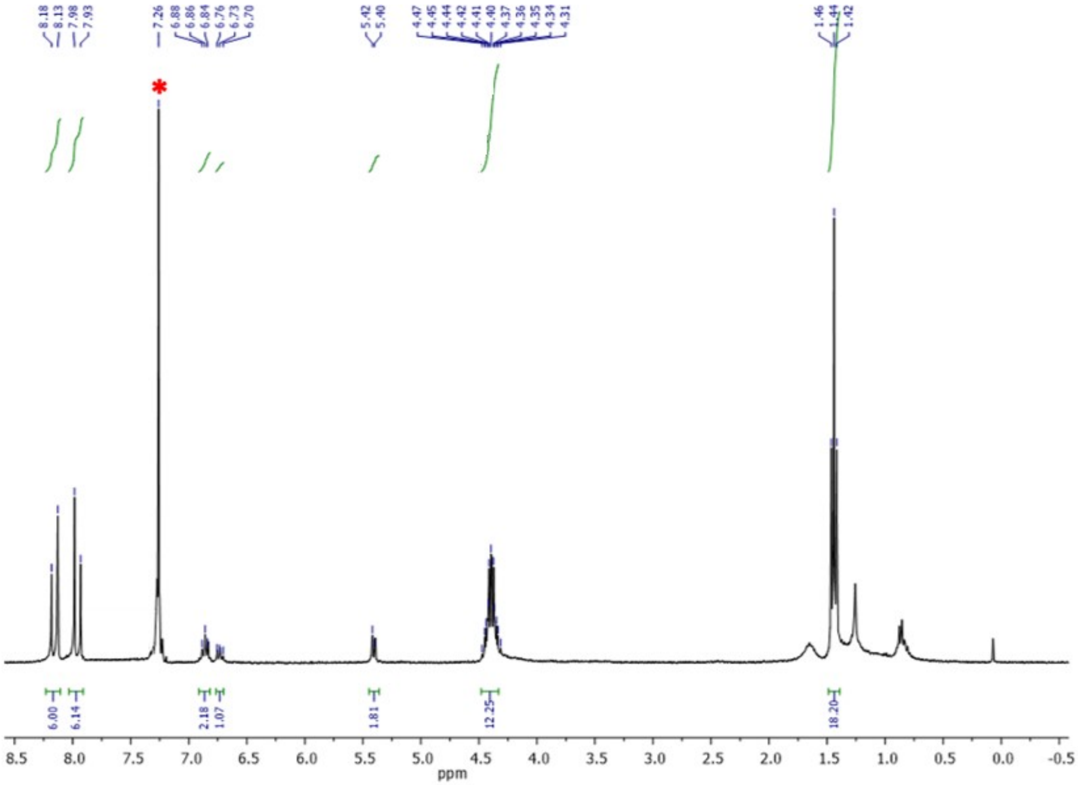

Figure S14. MS and HRMS spectra (MALDI-TOF) of **7b** showing the experimental isotopic patterns compared to the expected clusters (Upper: found; Lower: calculated).

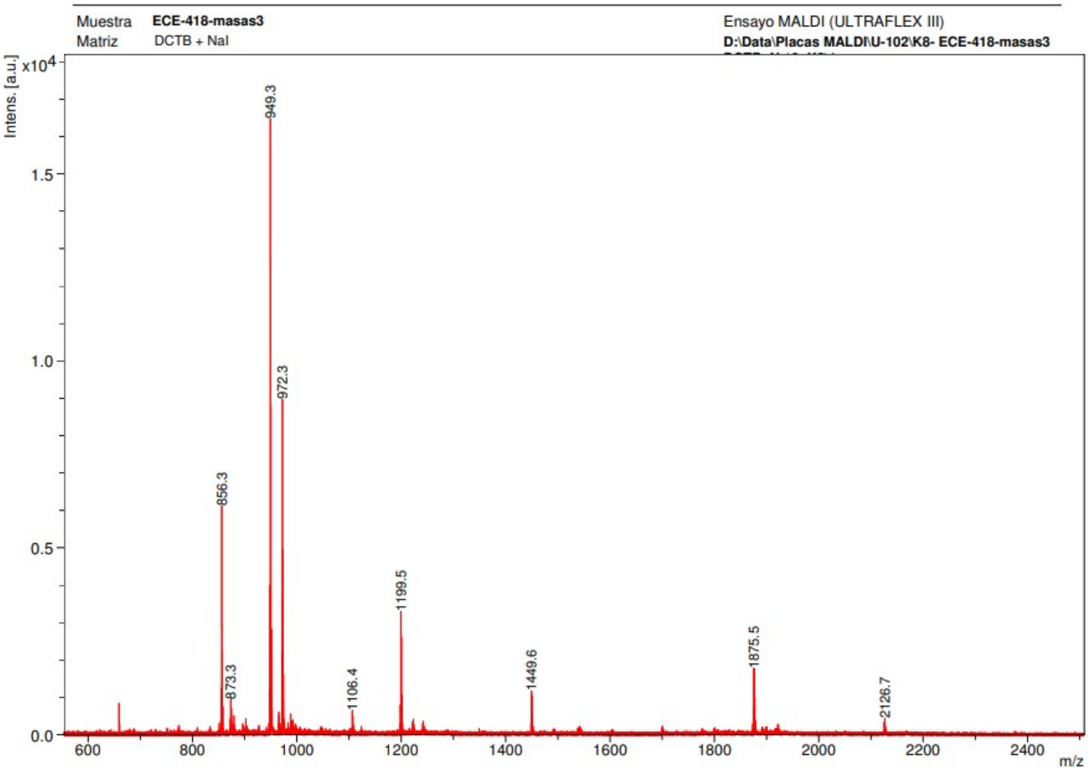

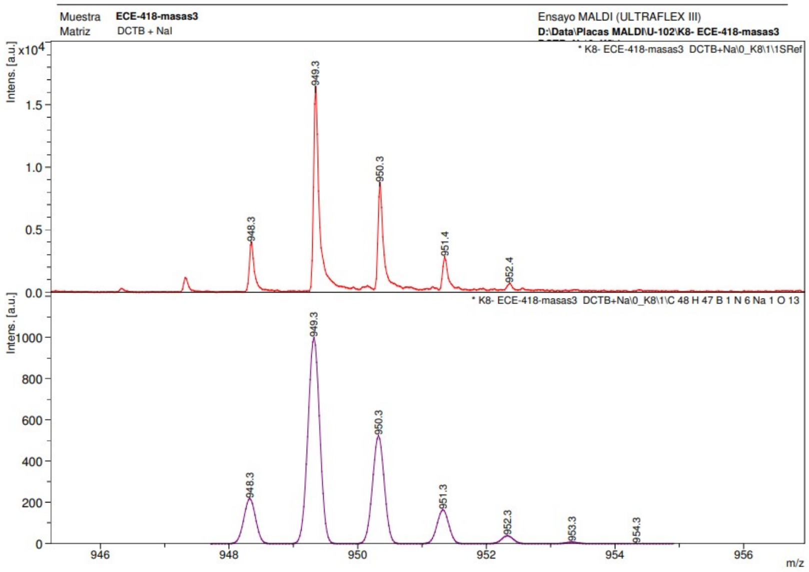
Muestra ECE-418-masas3
Matriz DCTB + NaI
Ensayo MALDI (ULTRAFLEX III)
D:\Data\Placas MALDI\U-102\K8- ECE-418-masas3
* K8- ECE-418-masas3 DCTB+Na\0_K8\1\1SRef
* K8- ECE-418-masas3 DCTB+Na\0_K8\1\C 48 H 47 B 1 N 6 Na 1 O 13
Intens. [a.u.]
x10^4
948.3
949.3
950.3
951.4
952.4
951.3
952.3
953.3
954.3
m/z

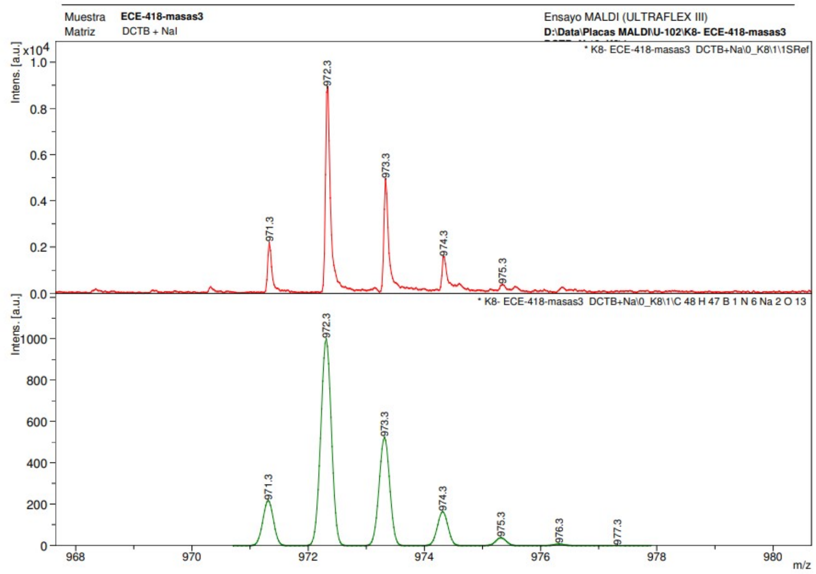
Muestra ECE-418-masas3
Matriz DCTB + NaI
Ensayo MALDI (ULTRAFLEX III)
D:\Data\Placas MALDI\U-102\K8- ECE-418-masas3
* K8- ECE-418-masas3 DCTB+Na\0_K8\1\1SRef
* K8- ECE-418-masas3 DCTB+Na\0_K8\1\C 48 H 47 B 1 N 6 Na 2 O 13
Intens. [a.u.]
x10^4
971.3
972.3
973.3
974.3
975.3
976.3
977.3
m/z

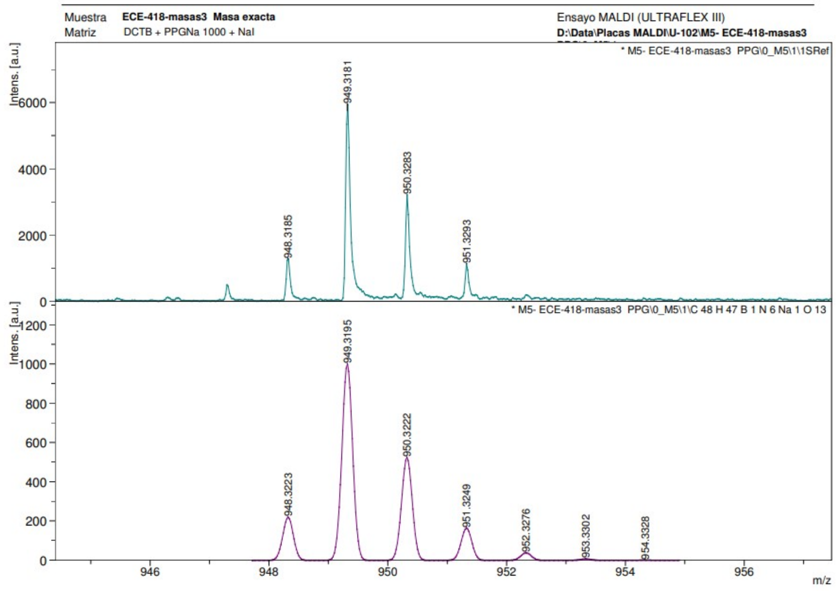
Muestra ECE-418-masas3 Masa exacta
Matriz DCTB + PPGNa 1000 + NaI
Ensayo MALDI (ULTRAFLEX III)
D:\Data\Placas MALDI\U-102\M5- ECE-418-masas3
* M5- ECE-418-masas3 PPG\0_M5\1\1SRef
* M5- ECE-418-masas3 PPG\0_M5\1\C 48 H 47 B 1 N 6 Na 1 O 13
Intens. [a.u.]
948.3185
949.3181
950.3283
951.3293
948.3223
949.3195
950.3222
951.3249
952.3276
953.3302
954.3328
m/z

Figure S15. ^{1}H NMR spectrum of SubPz **8** in THF-d_8.

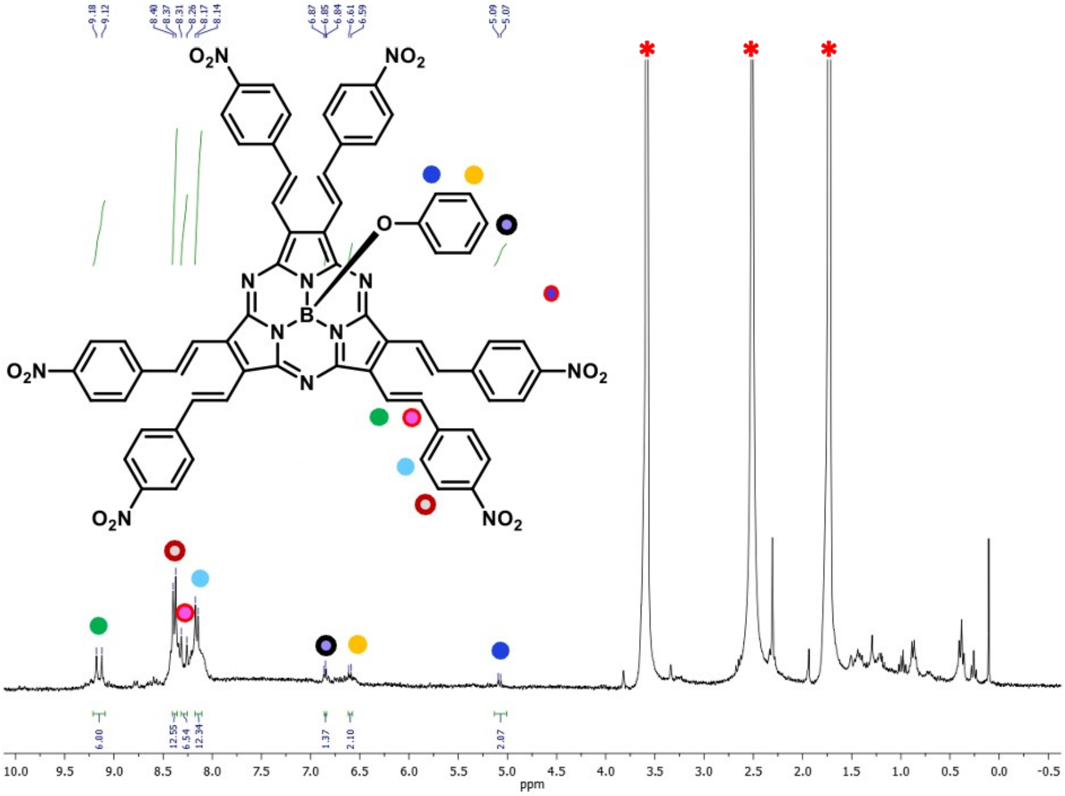

Figure S16. MS and HRMS (MALDI-TOF) spectra of SubPz **8** showing the experimental isotopic patterns compared to the expected clusters (Upper: found; Lower: calculated).

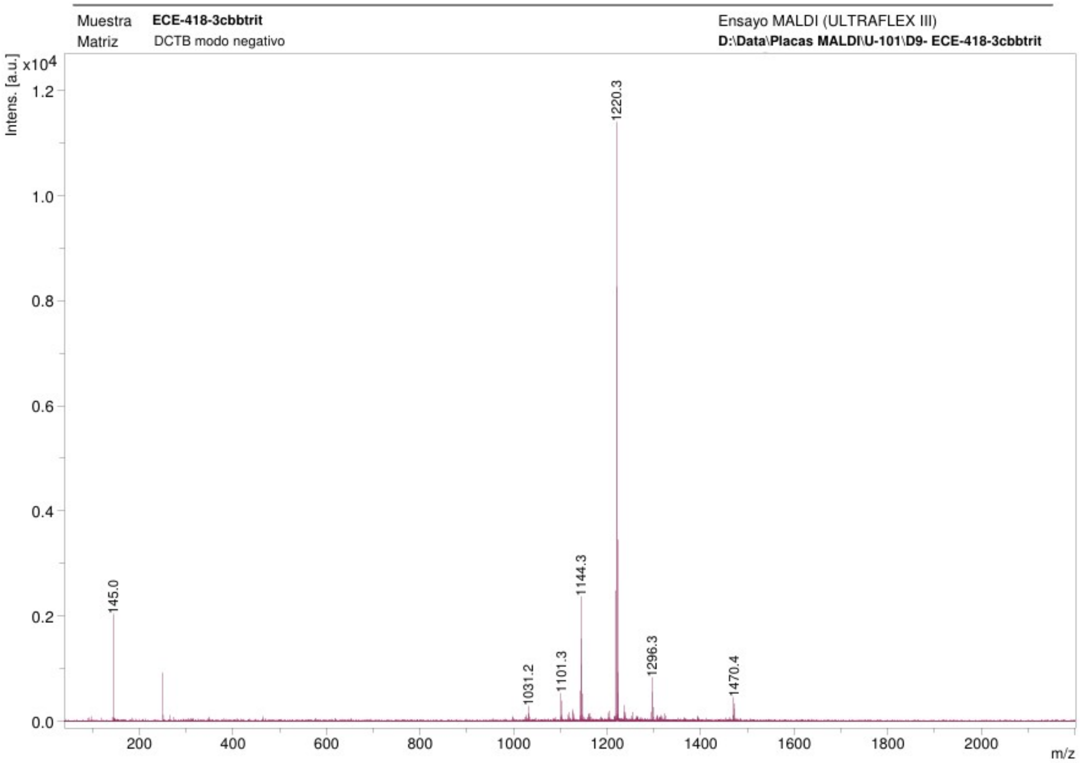

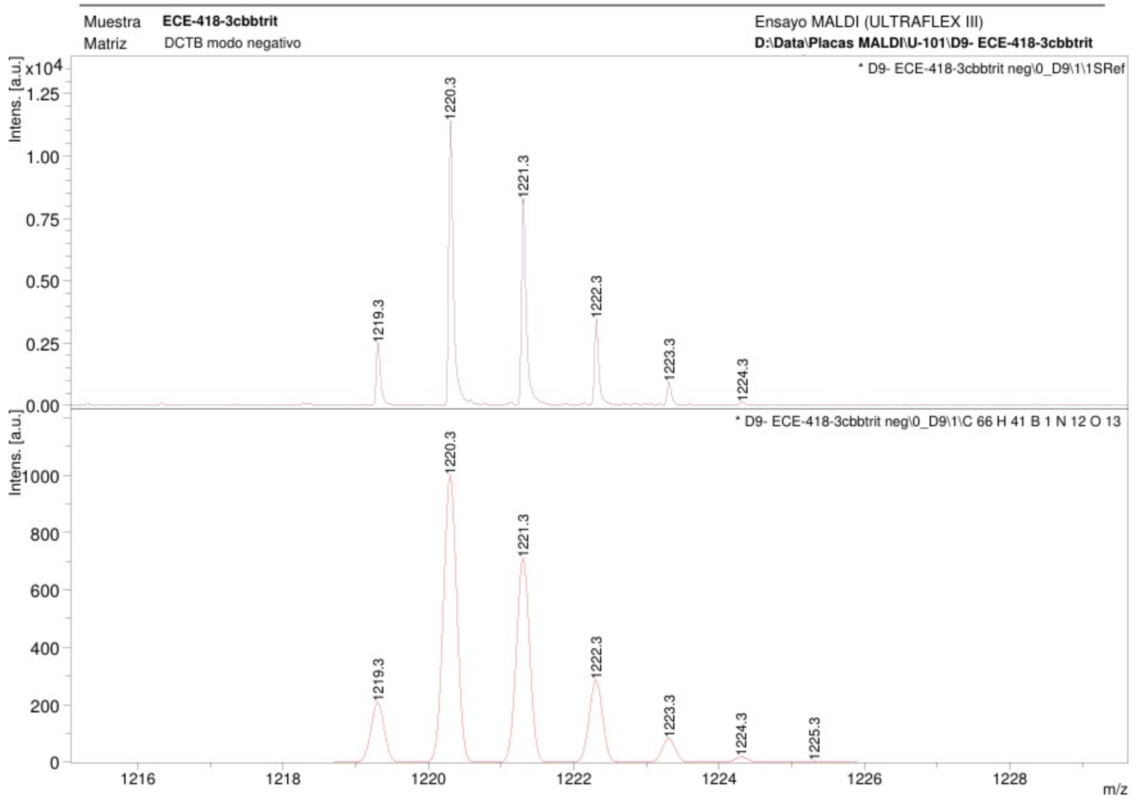
Muestra ECE-418-3cbbtrit
Matriz DCTB modo negativo
Ensayo MALDI (ULTRAFLEX III)
D:\Data\Placas MALDI\U-101\D9- ECE-418-3cbbtrit
* D9- ECE-418-3cbbtrit neg\0_D9\1\1SRef
Intens. [a.u.]
x10^4
1.25
1.00
0.75
0.50
0.25
0.00
1219.3
1220.3
1221.3
1222.3
1223.3
1224.3
* D9- ECE-418-3cbbtrit neg\0_D9\1\C 66 H 41 B 1 N 12 O 13
Intens. [a.u.]
1000
800
600
400
200
0
1219.3
1220.3
1221.3
1222.3
1223.3
1224.3
1225.3
1216
1218
1220
1222
1224
1226
1228
m/z

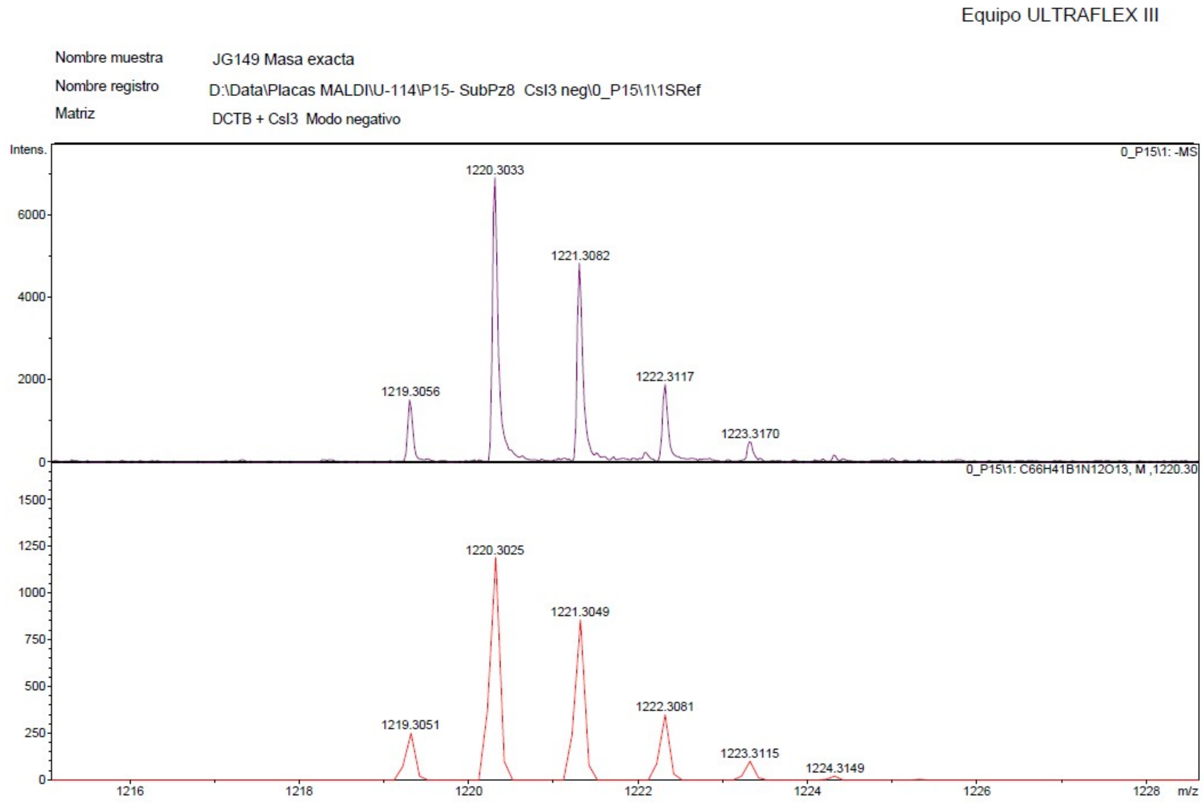
Equipo ULTRAFLEX III
Nombre muestra JG149 Masa exacta
Nombre registro D:\Data\Placas MALDI\U-114\P15- SubPz8 CsI3 neg\0_P15\1\1SRef
Matriz DCTB + CsI3 Modo negativo
Intens.
0_P15\1: -MS
6000
4000
2000
0
1219.3056
1220.3033
1221.3082
1222.3117
1223.3170
0_P15\1: C66H41B1N12O13, M ,1220.30
1500
1250
1000
750
500
250
0
1219.3051
1220.3025
1221.3049
1222.3081
1223.3115
1224.3149
1216
1218
1220
1222
1224
1226
1228
m/z

Figure S17. ^{1}H NMR spectrum of Ru(CO)Pc-SubPz **1** in $CDCl_3$.

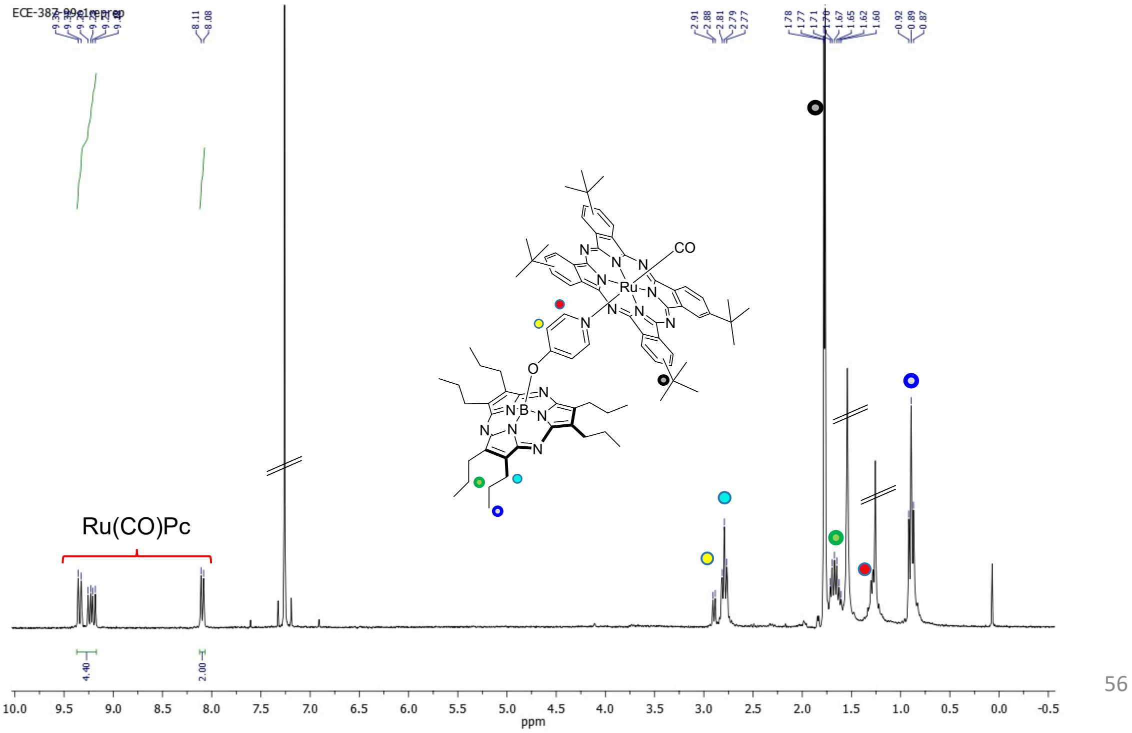

Figure S18. Comparative ^{1}H NMR spectra of Ru(CO)Pc-SubPz **1** and SubPz precursor **13**.

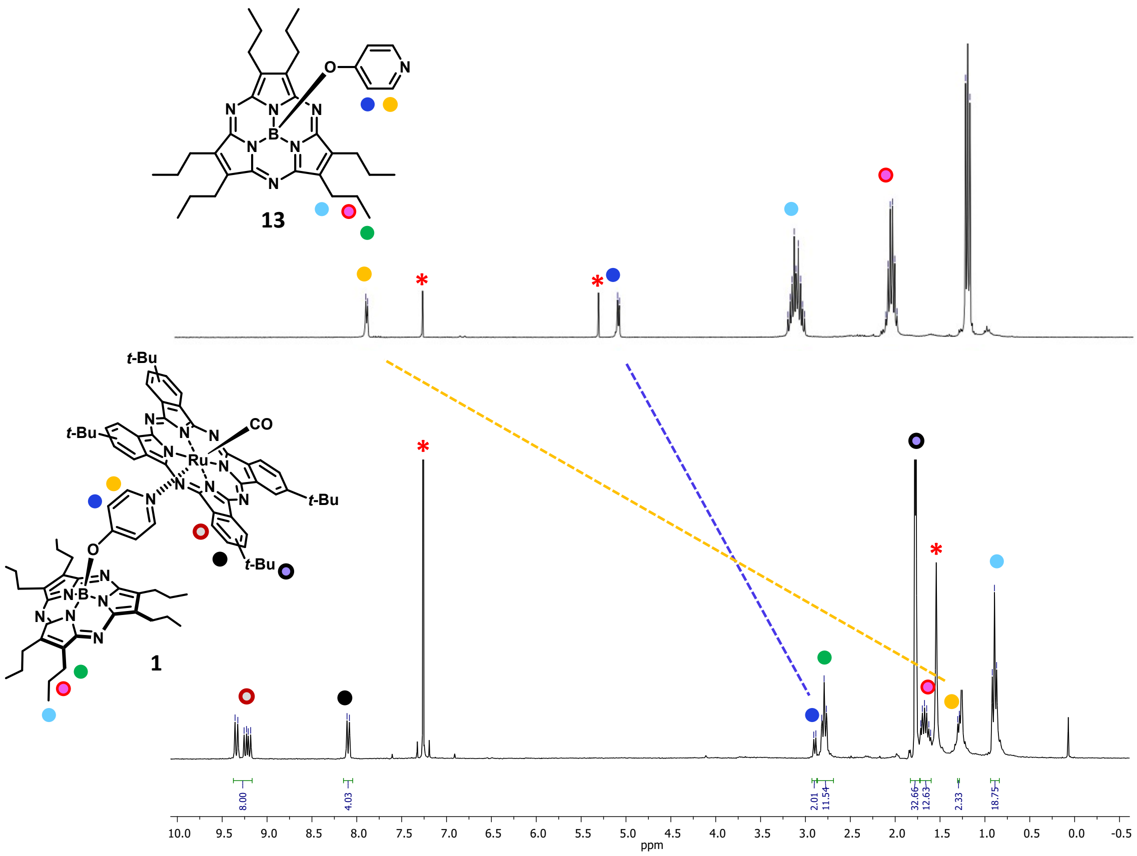

Figure S19. HRMS (ESI$^+$-TOF) spectrum of Ru(CO)Pc-SubPz **1** showing the experimental isotopic patterns compared to the expected clusters (Upper: found; Lower: calculated).

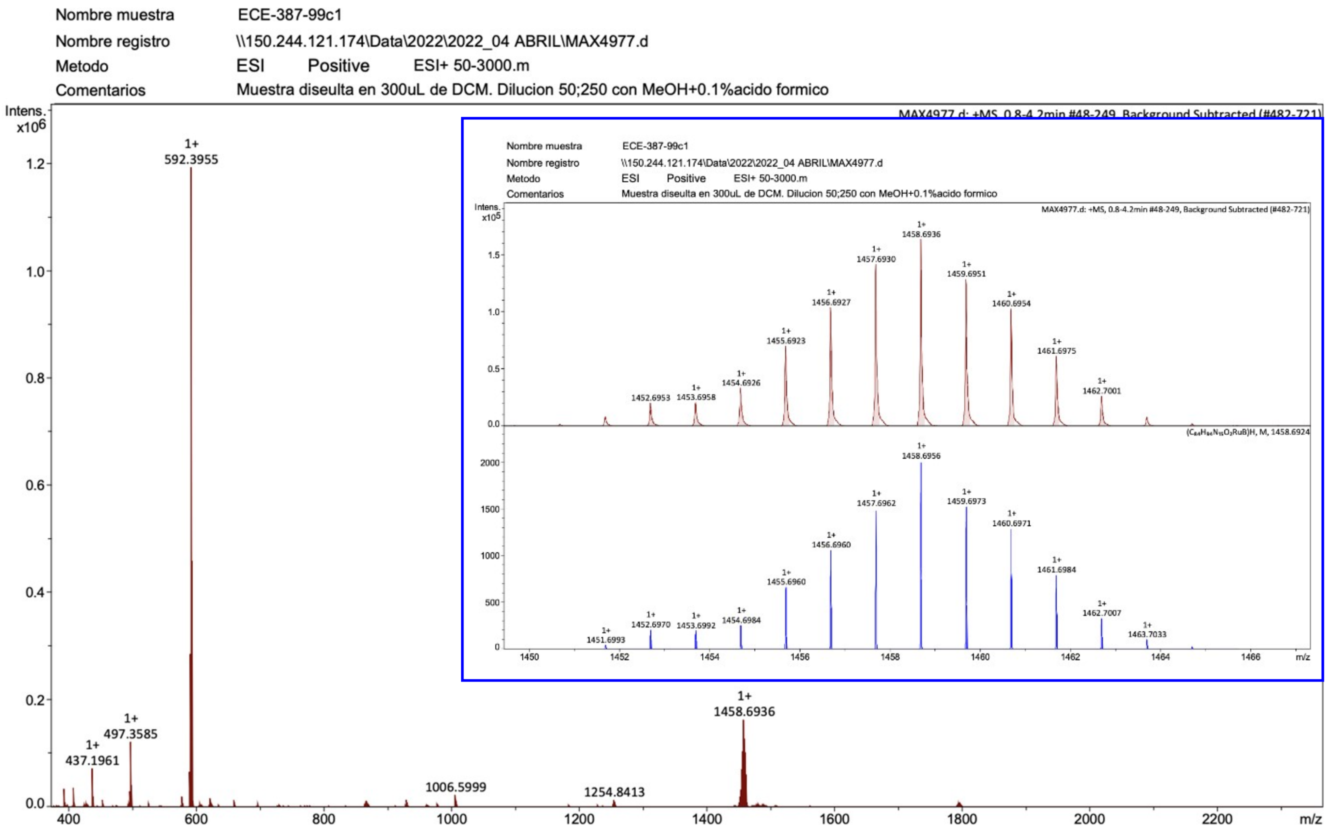

Figure S20. ^{1}H NMR spectrum of Ru(CO)Pc-SubPz **2** in $CDCl_3$.

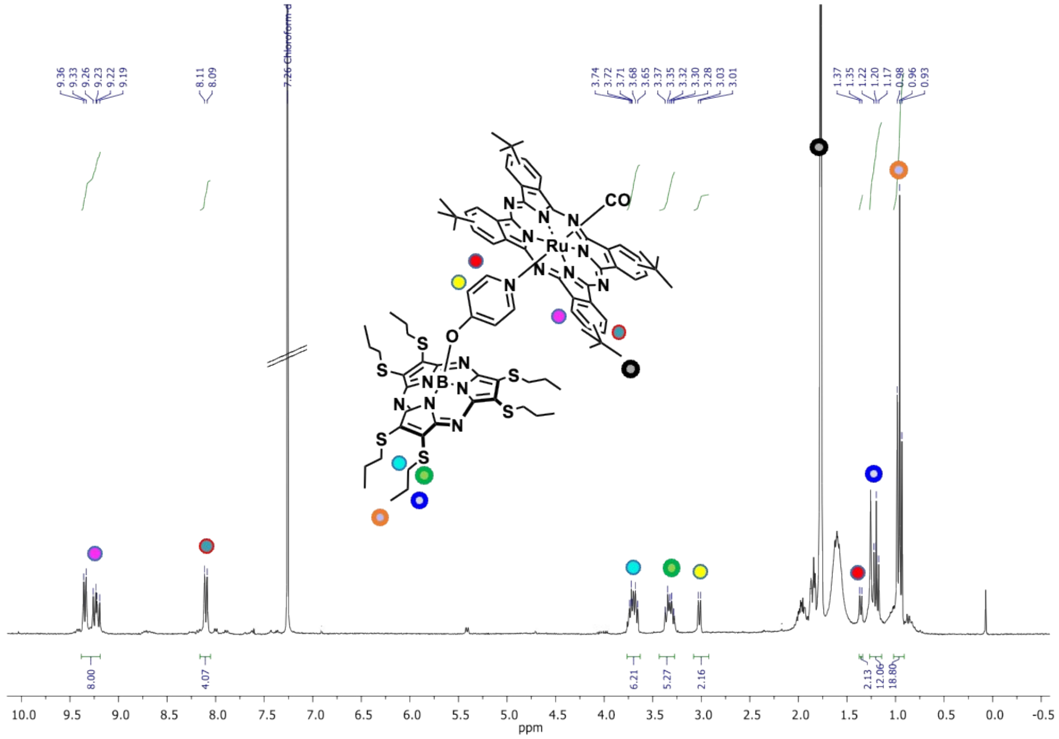

Figure S21. HRMS (ESI^{+}-TOF) of Ru(CO)Pc-SubPz **2** showing the experimental isotopic patterns compared to the expected clusters (Upper: found; Lower: calculated).

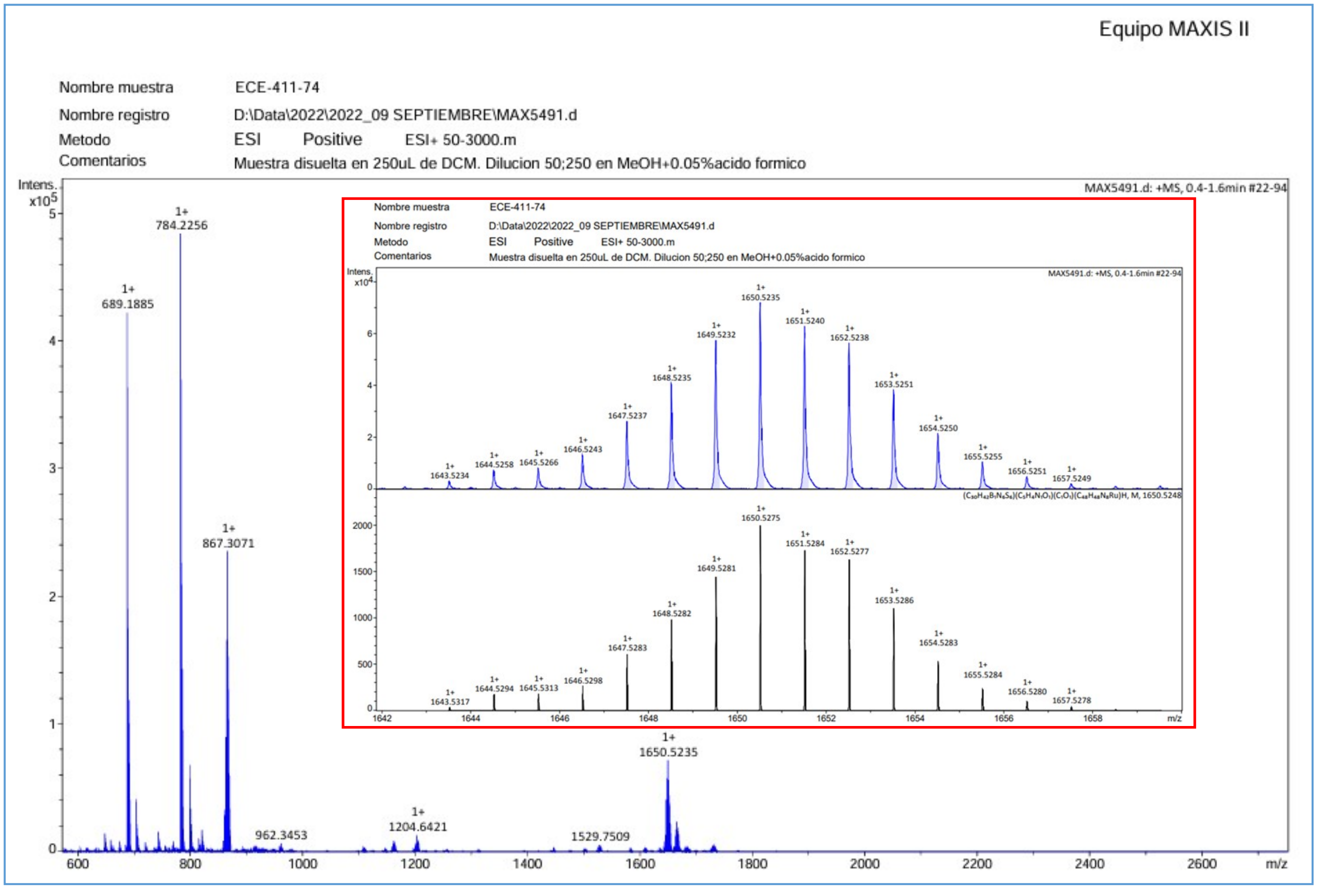

Figure S22. ^{1}H NMR spectrum of Ru(CO)Pc-SubPz **3** in $CDCl_3$. *= heptane

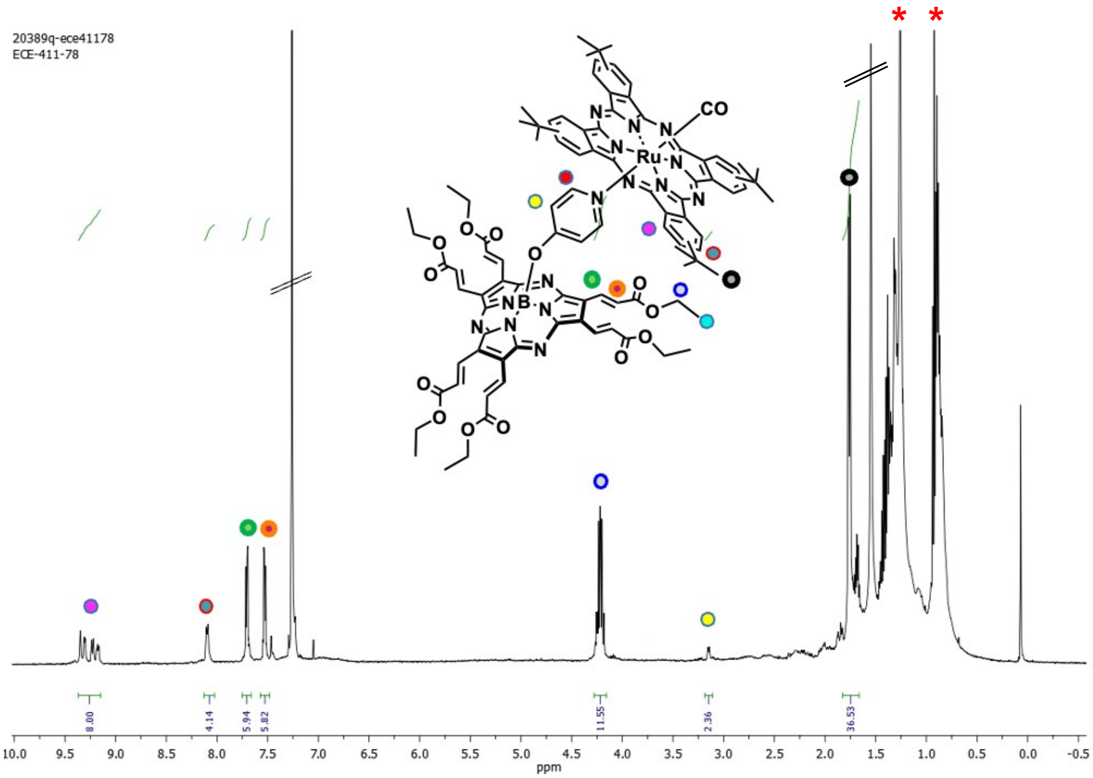

Figure S23. MS and HRMS (MALDI-TOF) of Ru(CO)Pc-SubPz **3** showing the experimental isotopic patterns compared to the expected clusters (Upper: found; Lower: calculated).

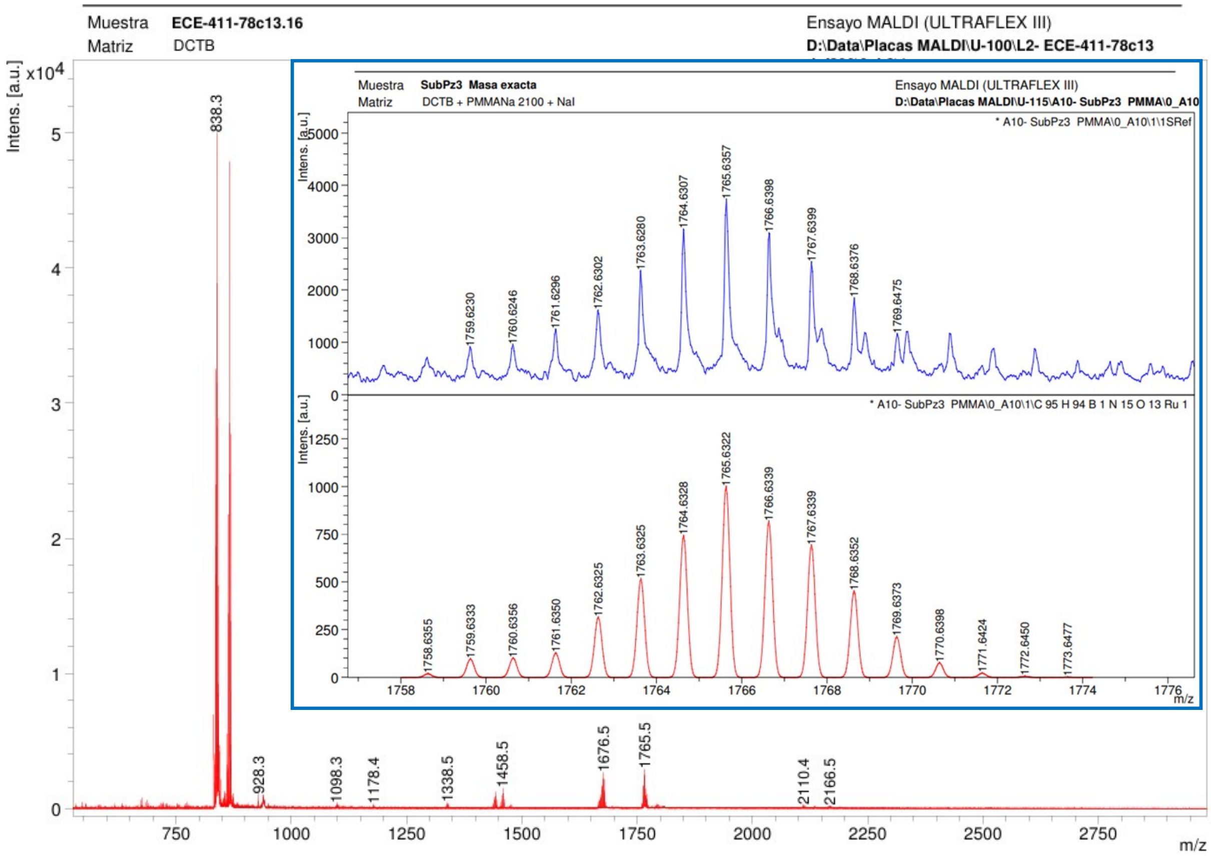

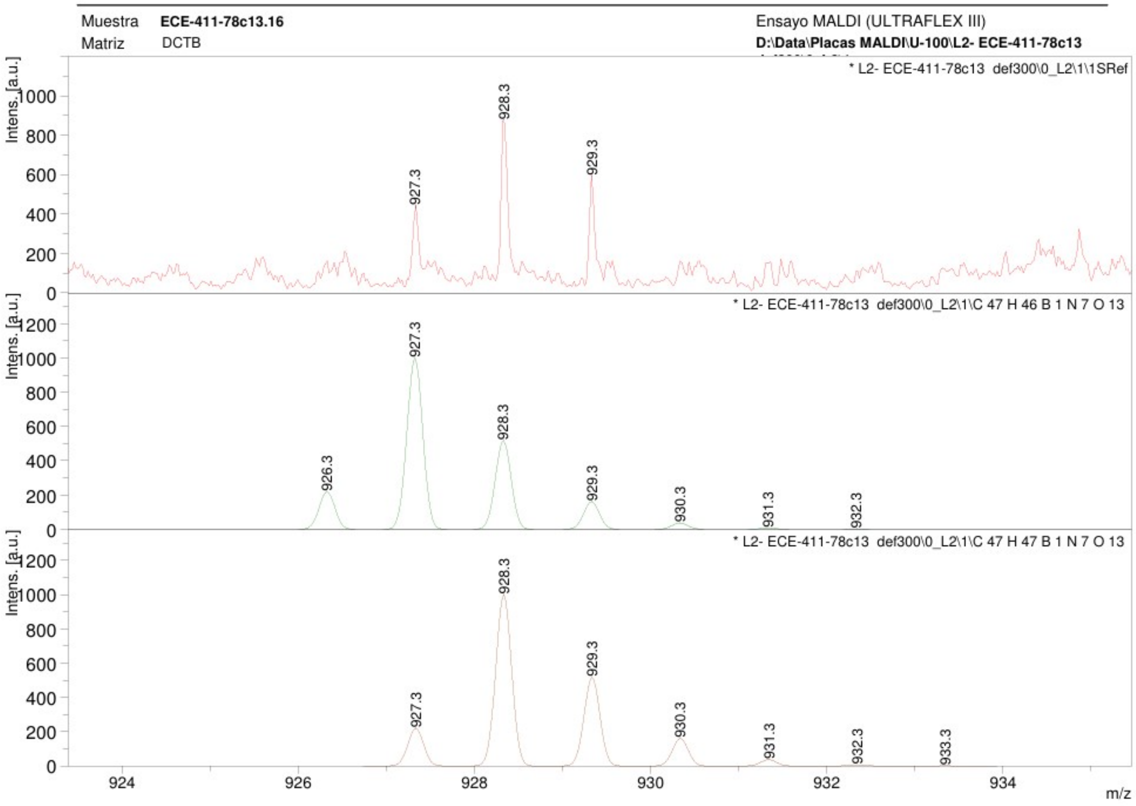
Muestra ECE-411-78c13.16
Matriz DCTB
Ensayo MALDI (ULTRAFLEX III)
D:\Data\Placas MALDI\U-100\L2- ECE-411-78c13
* L2- ECE-411-78c13 def300\0_L2\1\1SRef
* L2- ECE-411-78c13 def300\0_L2\1\C 47 H 46 B 1 N 7 O 13
* L2- ECE-411-78c13 def300\0_L2\1\C 47 H 47 B 1 N 7 O 13
Intens. [a.u.]
m/z

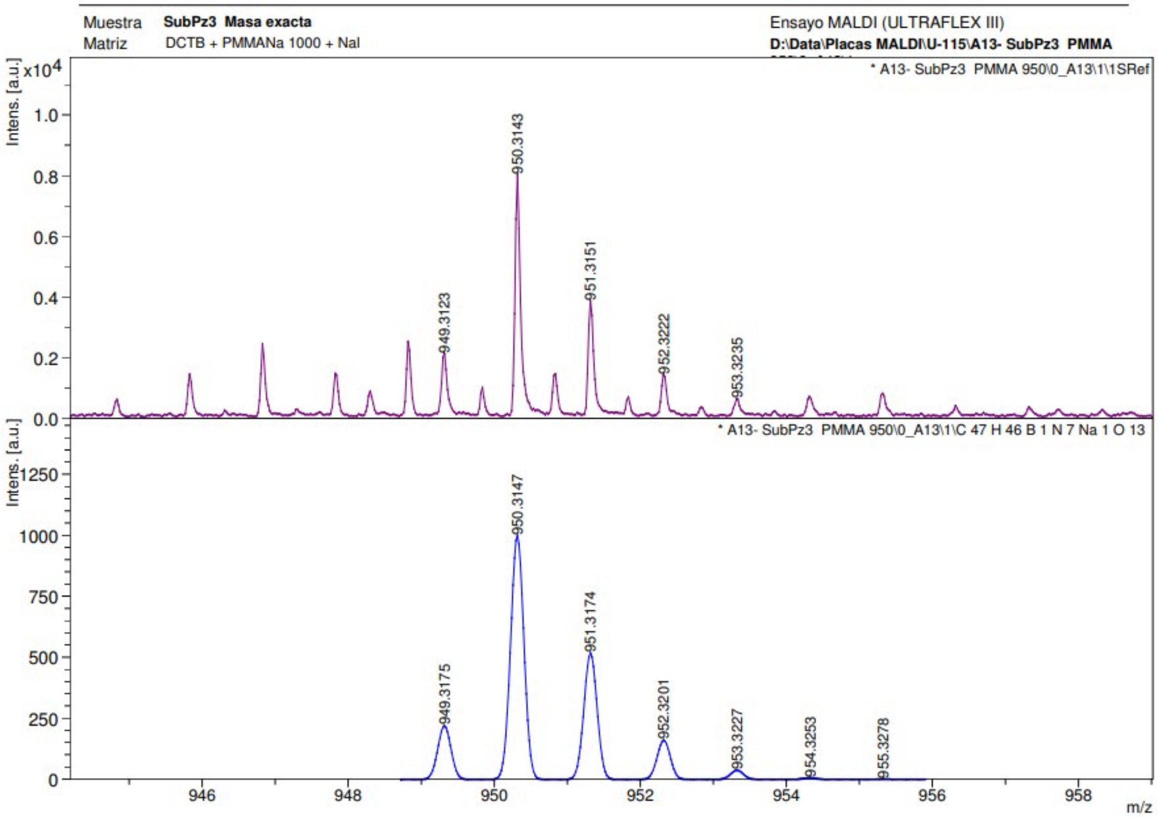
Muestra SubPz3 Masa exacta
Matriz DCTB + PMMANa 1000 + NaI
Ensayo MALDI (ULTRAFLEX III)
D:\Data\Placas MALDI\U-115\A13- SubPz3 PMMA
* A13- SubPz3 PMMA 950\0_A13\1\1SRef
* A13- SubPz3 PMMA 950\0_A13\1\C 47 H 46 B 1 N 7 Na 1 O 13
Intens. [a.u.]
x10^4
m/z

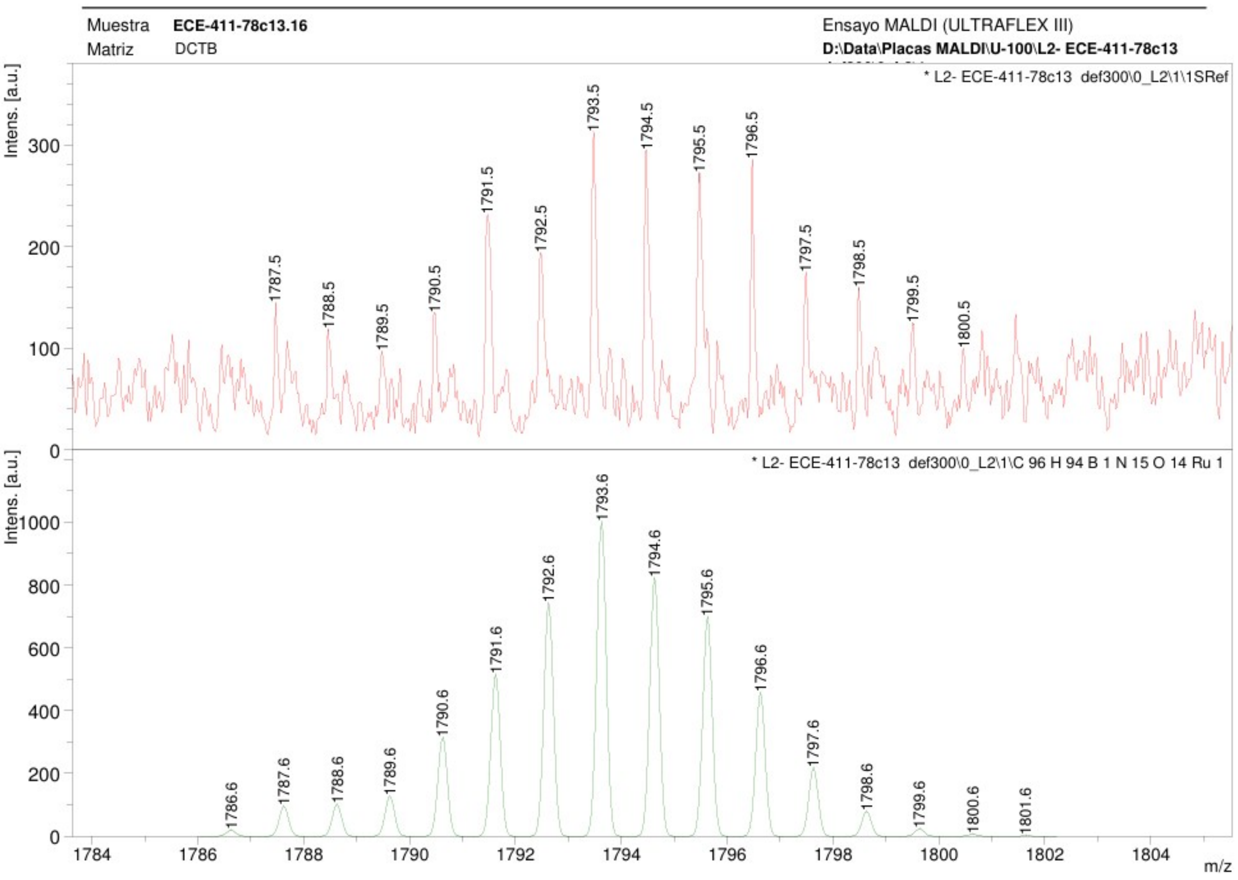

Muestra ECE-411-78c13.16
Matriz DCTB
Ensayo MALDI (ULTRAFLEX III)
D:\Data\Placas MALDI\U-100\L2- ECE-411-78c13
* L2- ECE-411-78c13 def300\0_L2\1\1SRef
* L2- ECE-411-78c13 def300\0_L2\1\C 96 H 94 B 1 N 15 O 14 Ru 1
Intens. [a.u.]
m/z

Figure S24. ^{1}H NMR spectrum of Ru(CO)Pc-SubPz **4** in $CDCl_3$. *= chloroform

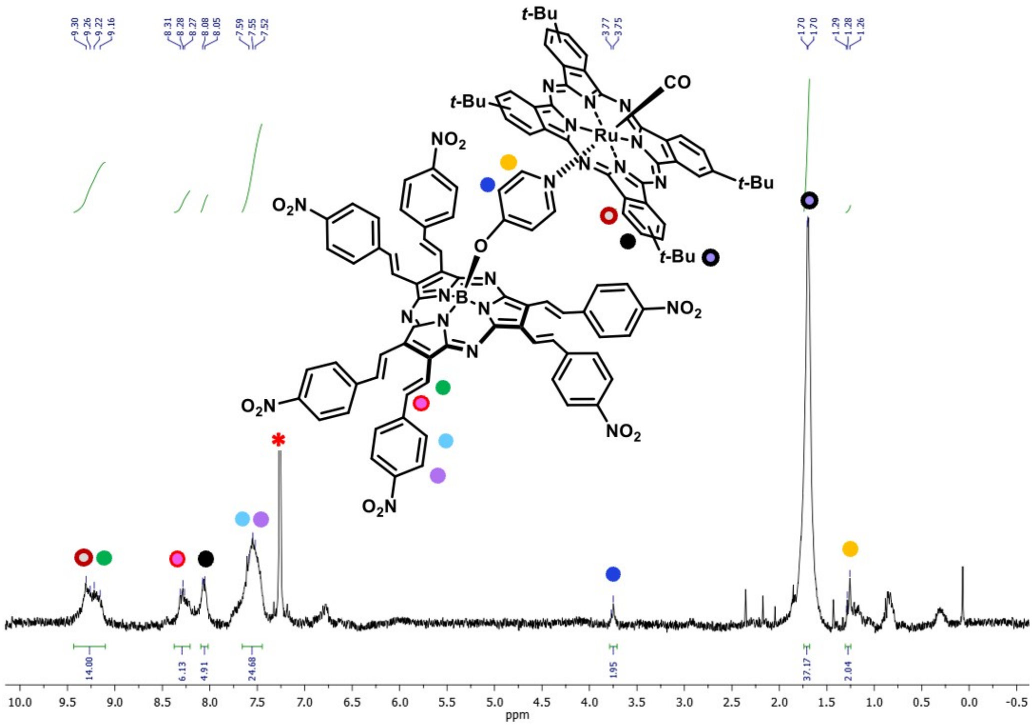

Figure S25. MS (MALDI-TOF^{+}) and MS (MALDI-TOF^{-}) of Ru(CO)Pc-SubPz **4** showing the experimental isotopic patterns compared to the expected clusters (Upper: found; Lower: calculated).

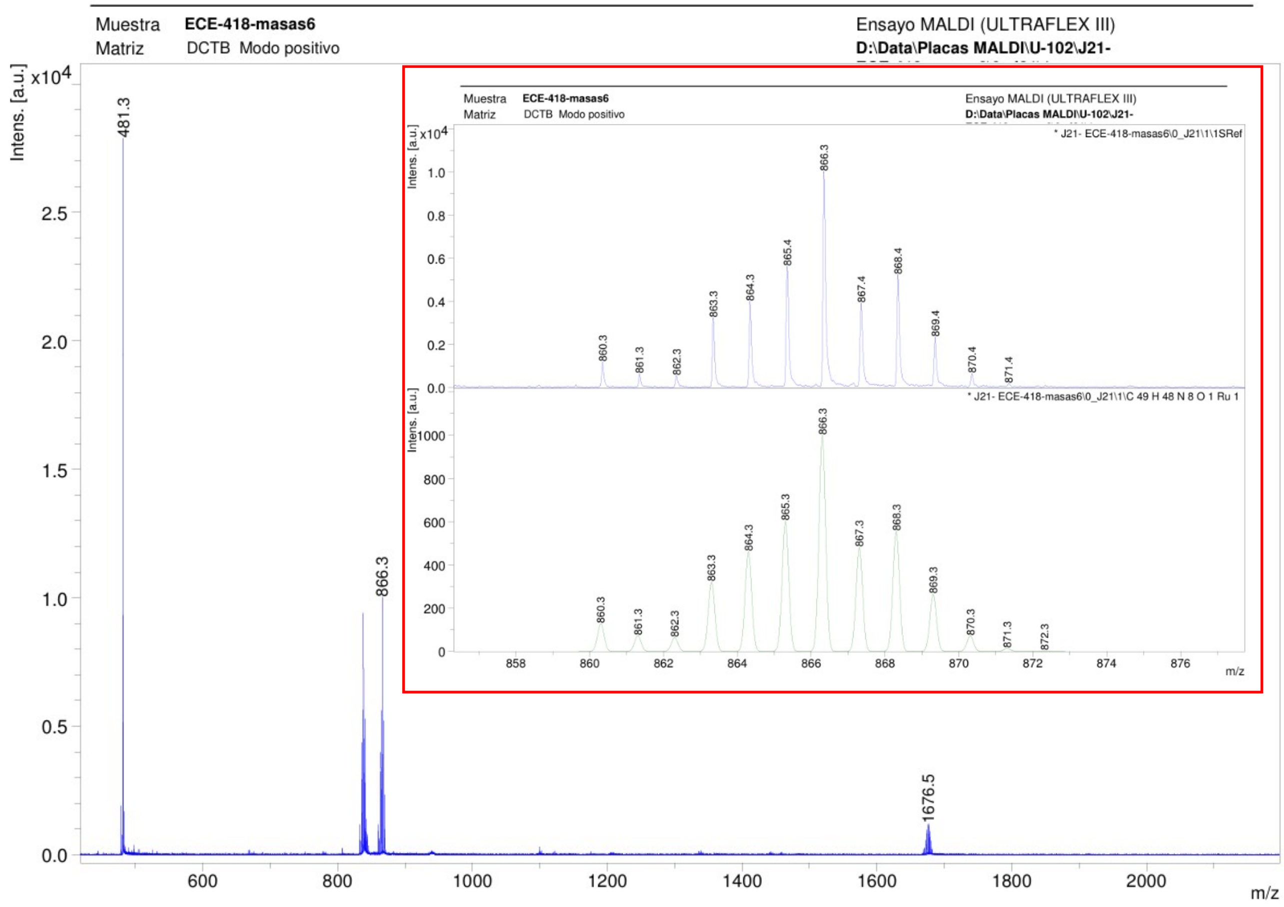

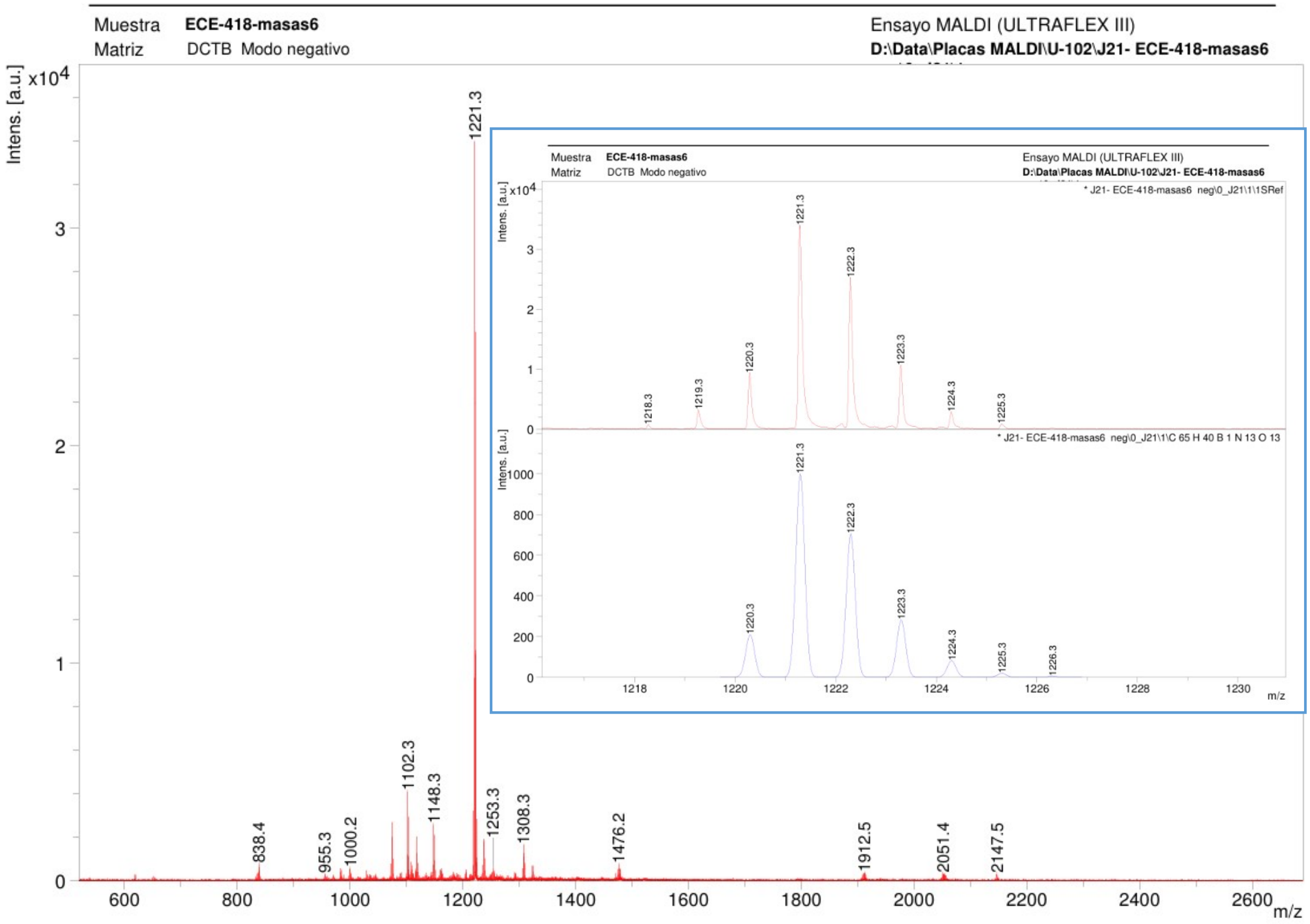

Muestra ECE-418-masas6
Matriz DCTB Modo negativo
Ensayo MALDI (ULTRAFLEX III)
D:\Data\Placas MALDI\U-102\J21- ECE-418-masas6
Intens. [a.u.]
x10^4
m/z
* J21- ECE-418-masas6 neg\0_J21\1\1SRef
* J21- ECE-418-masas6 neg\0_J21\1\C 65 H 40 B 1 N 13 O 13

4. Electrochemical data

Figure S26. Cyclic voltammograms and differential pulse voltammograms of Ru(CO)Pc **11**. Solvent: CH_2Cl_2; scan rate: 100 mVs^{-1}; working electrode: GC; counter electrode: platinum wire; reference electrode: $Ag/AgNO_3$; electrolyte: 0.1M Bu_4NPF_6.

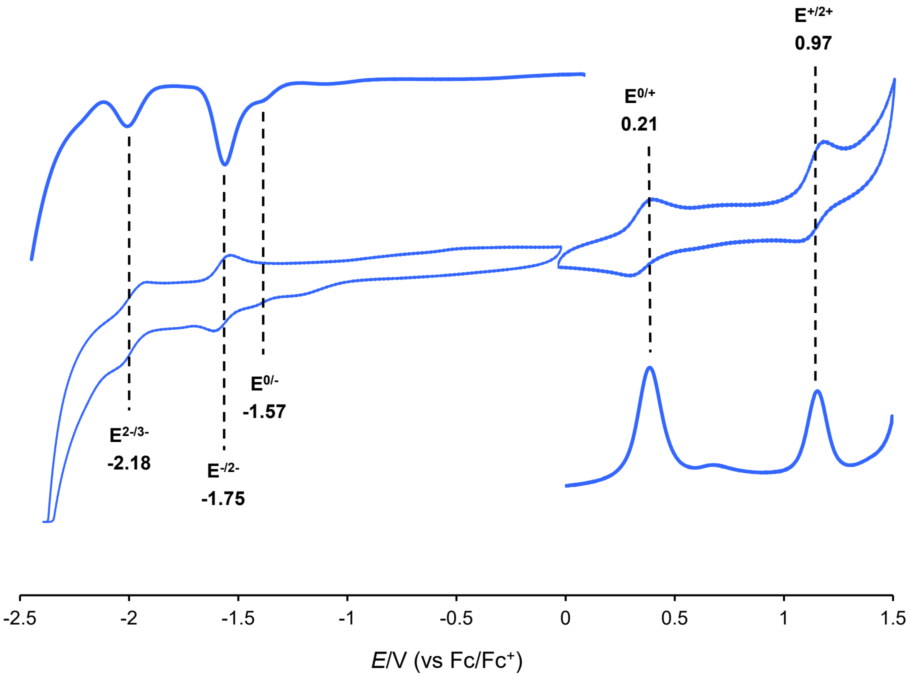

Figure S27. Cyclic voltammograms and differential pulse voltammograms of Ru(CO)PyPc **12**. Solvent: CH_2Cl_2; scan rate: 100 mVs^{-1}; working electrode: GC; counter electrode: platinum wire; reference electrode: $Ag/AgNO_3$; electrolyte: 0.1M Bu_4NPF_6.

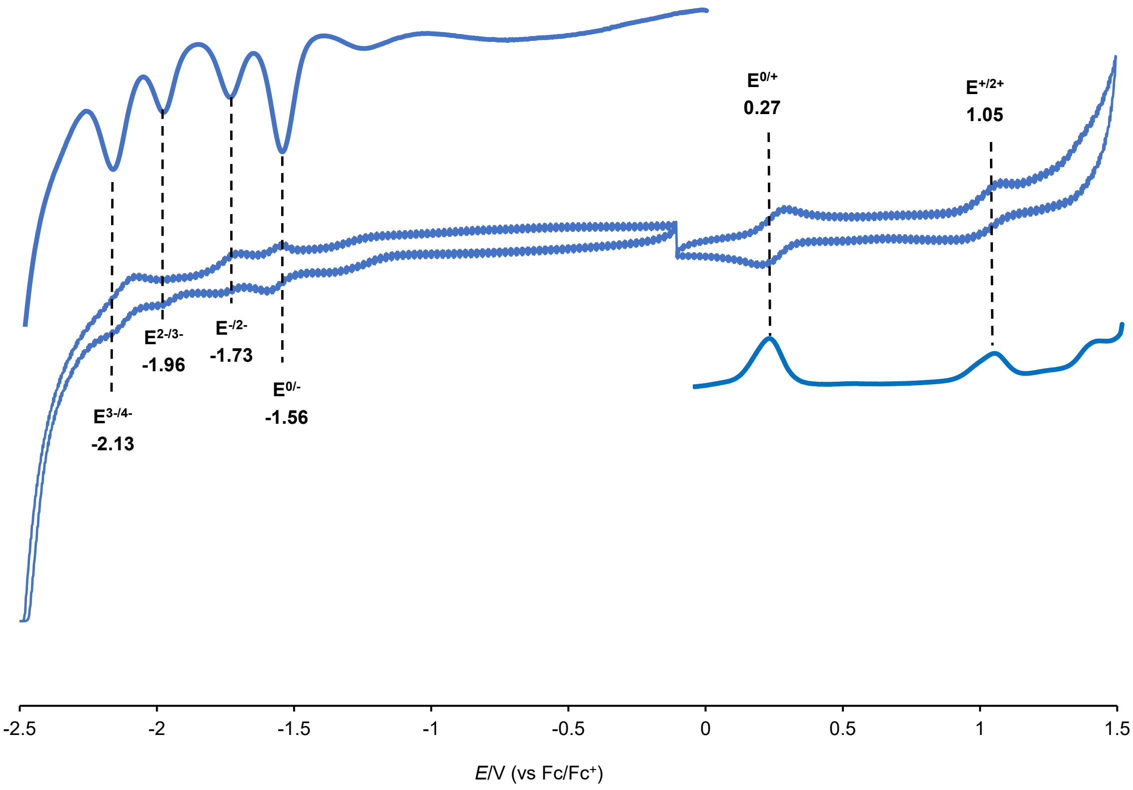

Figure S28. Cyclic voltammograms and differential pulse voltammograms of SubPz **13**. Solvent: CH_2Cl_2; scan rate: 100 mVs^{-1}; working electrode: GC; counter electrode: platinum wire; reference electrode: $Ag/AgNO_3$; electrolyte: 0.1M Bu_4NPF_6.

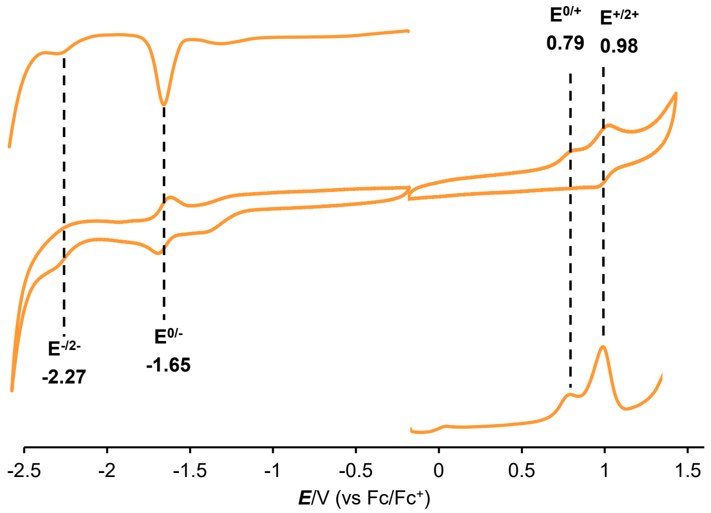

Figure S29. Cyclic voltammograms and differential pulse voltammograms of SubPz **6**. Solvent: CH_2Cl_2; scan rate: 100 mVs^{-1}; working electrode: GC; counter electrode: platinum wire; reference electrode: $Ag/AgNO_3$; electrolyte: 0.1M Bu_4NPF_6.

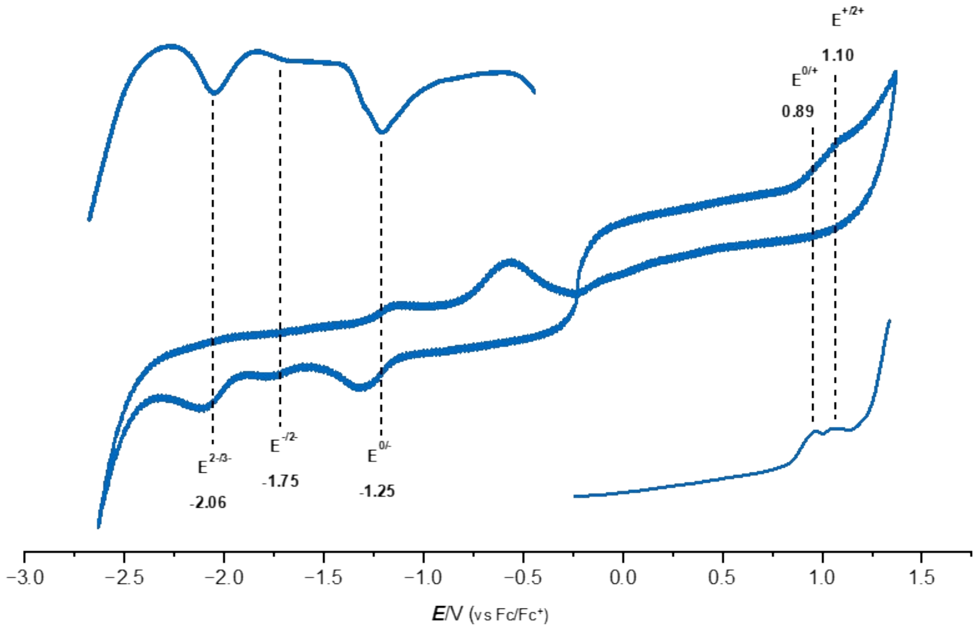

Figure S30. Cyclic voltammograms and differential pulse voltammograms of SubPz **7a**. Solvent:CH_2Cl_2; scan rate: 100mVs^{-1}; working electrode: GC; counter electrode: platinum wire; reference electrode: Ag/$AgNO_3$; electrolyte: 0.1M Bu_4NPF_6.

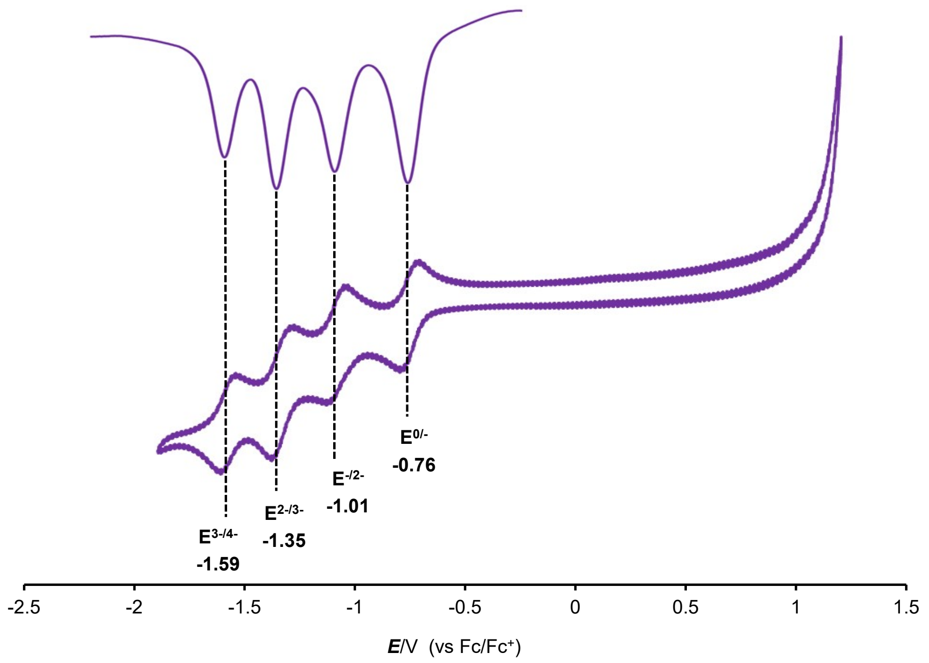

Figure S31. Cyclic voltammograms and differential pulse voltammograms of SubPz **7b**. Solvent: CH_2Cl_2; scan rate: 100 mVs^{-1}; working electrode: GC; counter electrode: platinum wire; reference electrode: Ag/$AgNO_3$; electrolyte: 0.1M Bu_4NPF_6.

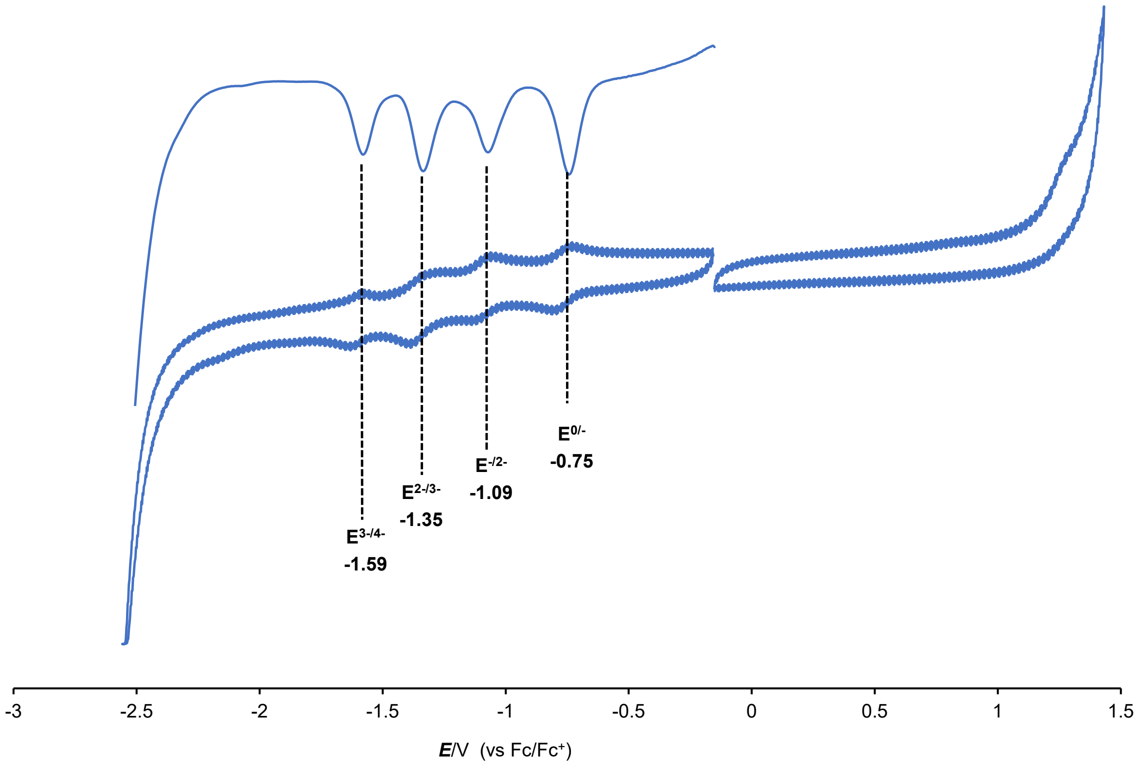

Figure S32. Cyclic voltammograms and differential pulse voltammograms of SubPz **8**. Solvent: THF; scan rate: 100 mVs^{-1}; working electrode: GC; counter electrode: platinum wire; reference electrode: Ag/AgNO$_3$; electrolyte: 0.1M Bu$_4$NPF$_6$.

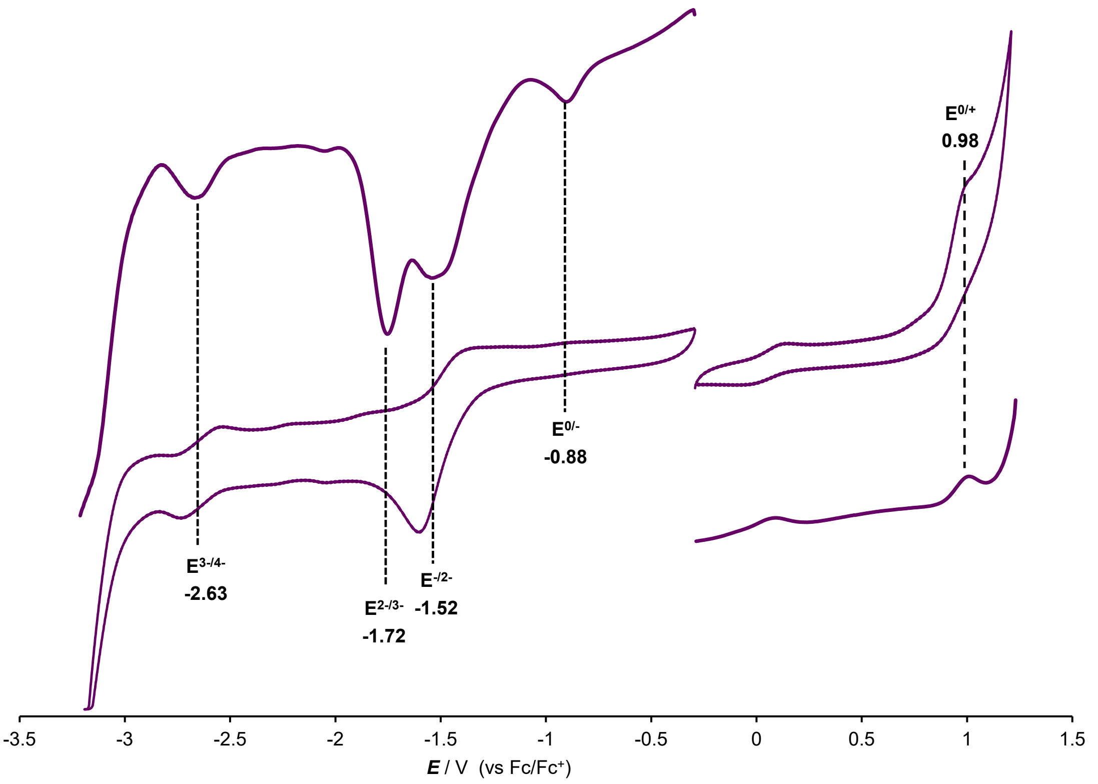

Figure S33. Cyclic voltammograms and differential pulse voltammograms of Ru(CO)Pc-SubPz **1**. Solvent: CH_2Cl_2; scan rate: 100 mVs^{-1}; working electrode: GC; counter electrode: platinum wire; reference electrode: Ag/AgNO$_3$; electrolyte: 0.1M Bu$_4$NPF$_6$.

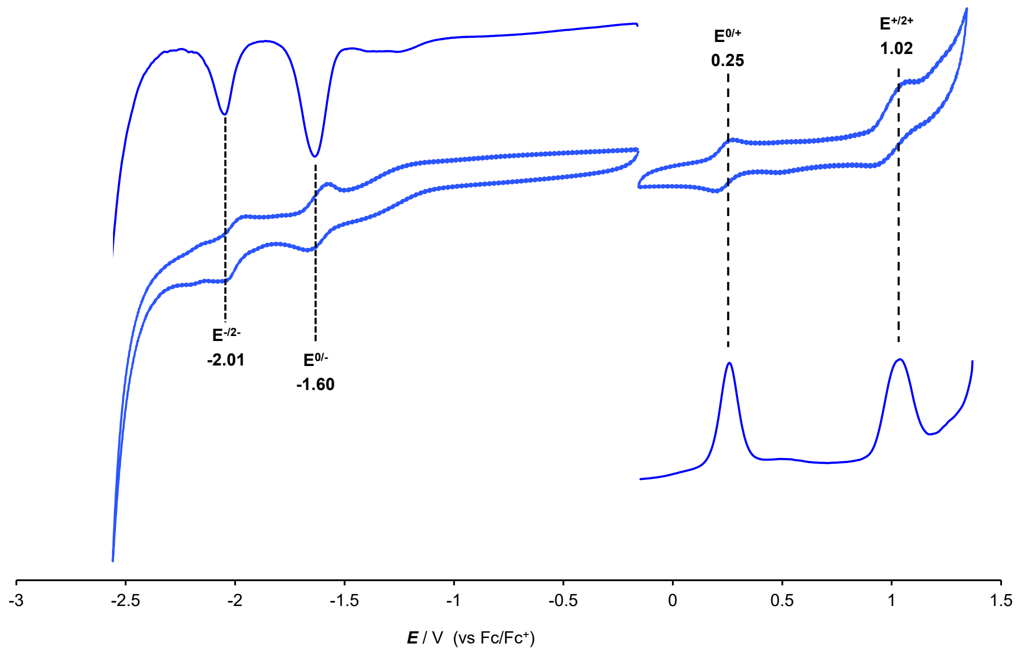

Figure S34. Cyclic voltammograms and differential pulse voltammograms of Ru(CO)Pc-SubPz **2**. Solvent: CH_2Cl_2; scan rate: 100 mVs^{-1}; working electrode: GC; counter electrode: platinum wire; reference electrode: $Ag/AgNO_3$; electrolyte: 0.1M Bu_4NPF_6.

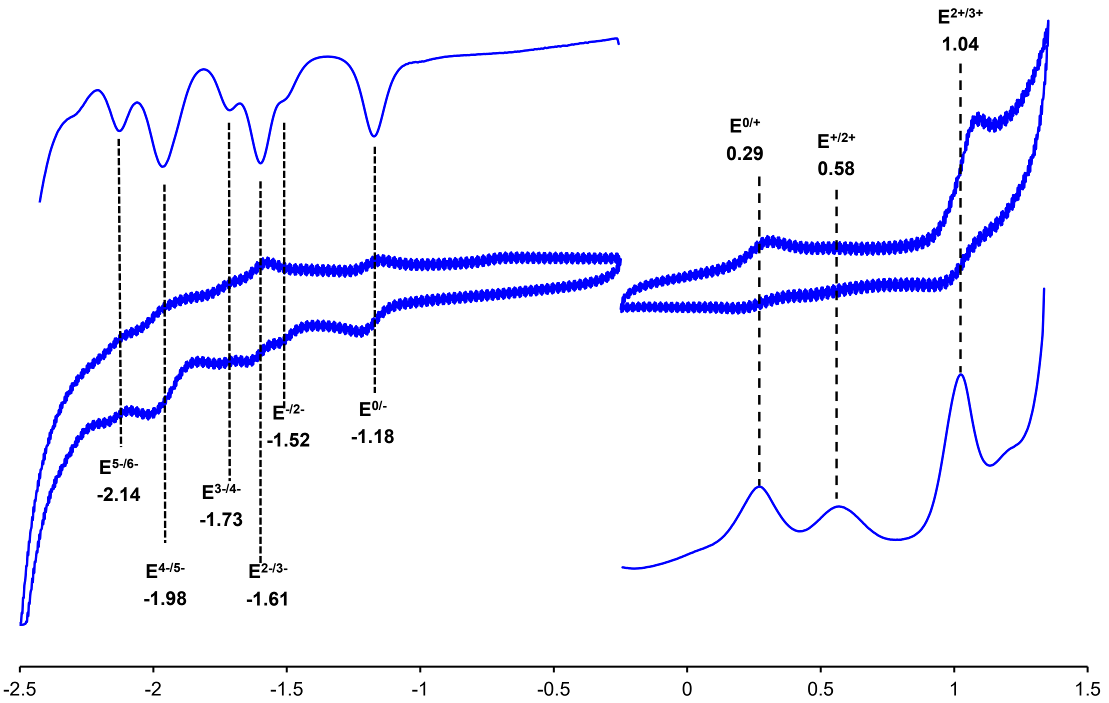

Figure S35. Cyclic voltammograms and differential pulse voltammograms of Ru(CO)Pc-SubPz **3**. Solvent: CH_2Cl_2; scan rate: 100 mVs^{-1}; working electrode: GC; counter electrode: platinum wire; reference electrode: $Ag/AgNO_3$; electrolyte: 0.1M Bu_4NPF_6.

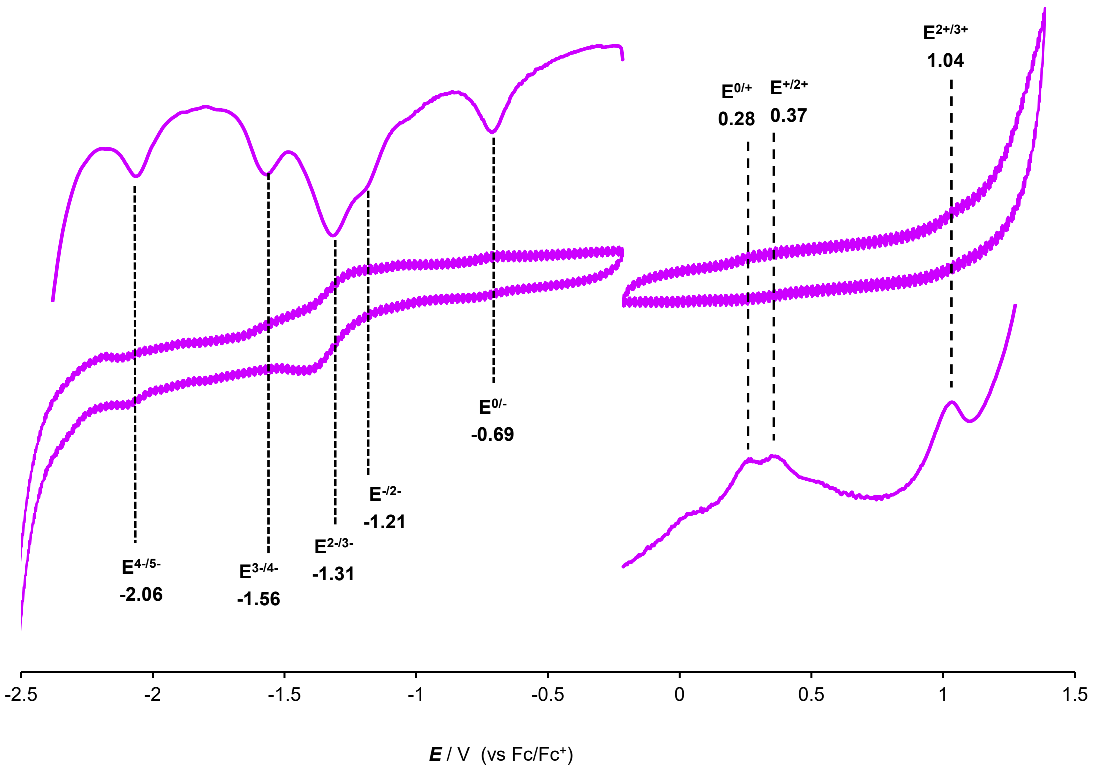

Figure S36. Cyclic voltammograms and differential pulse voltammograms of Ru(CO)Pc-SubPz **4**. Solvent: CH_2Cl_2; scan rate: 100 mVs^{-1}; working electrode: GC; counter electrode: platinum wire; reference electrode: Ag/$AgNO_3$; electrolyte: 0.1M Bu_4NPF_6.

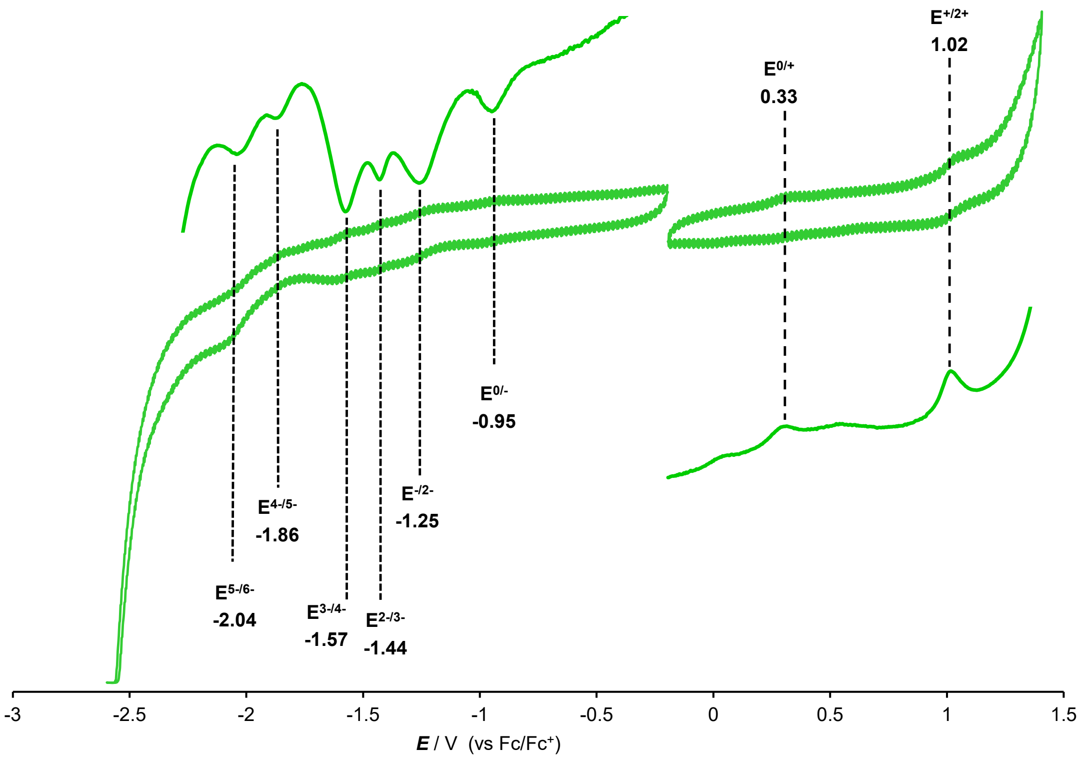

5. Steady-state absorption and fluorescence data

Figure S37. (a-c) Absorption spectra of Ru(CO)Pc **12**, SubPzs **13**, **6**, **7b**, and the corresponding Ru(CO)Pc-SubPz conjugates **1**, **2**, and **3**, measured in toluene at room temperature. (d) Normalized absorption spectra of Ru(CO)Pc **12** and Ru(CO)Pc-SubPz **3** in toluene.

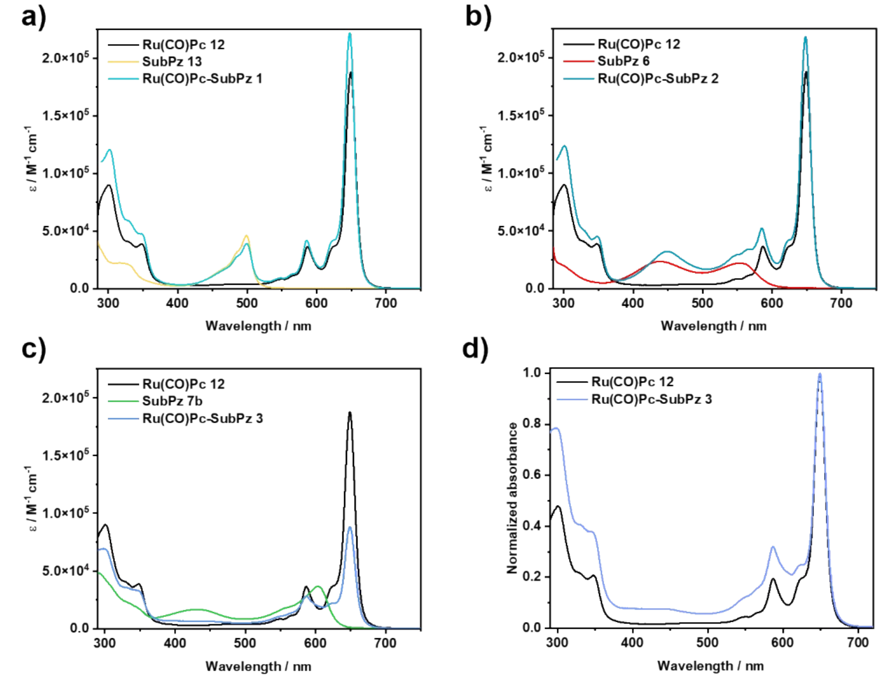

Figure S38. (a-d) Absorption spectra of Ru(CO)Pc **12**, SubPzs **13**, **6**, **7b**, and **8** and the corresponding Ru(CO)Pc-SubPz conjugates **1**, **2**, **3** and **4**, measured in THF at room temperature. (e) Normalized absorption spectra of Ru(CO)Pc **12** and Ru(CO)Pc-SubPz **4** in THF.

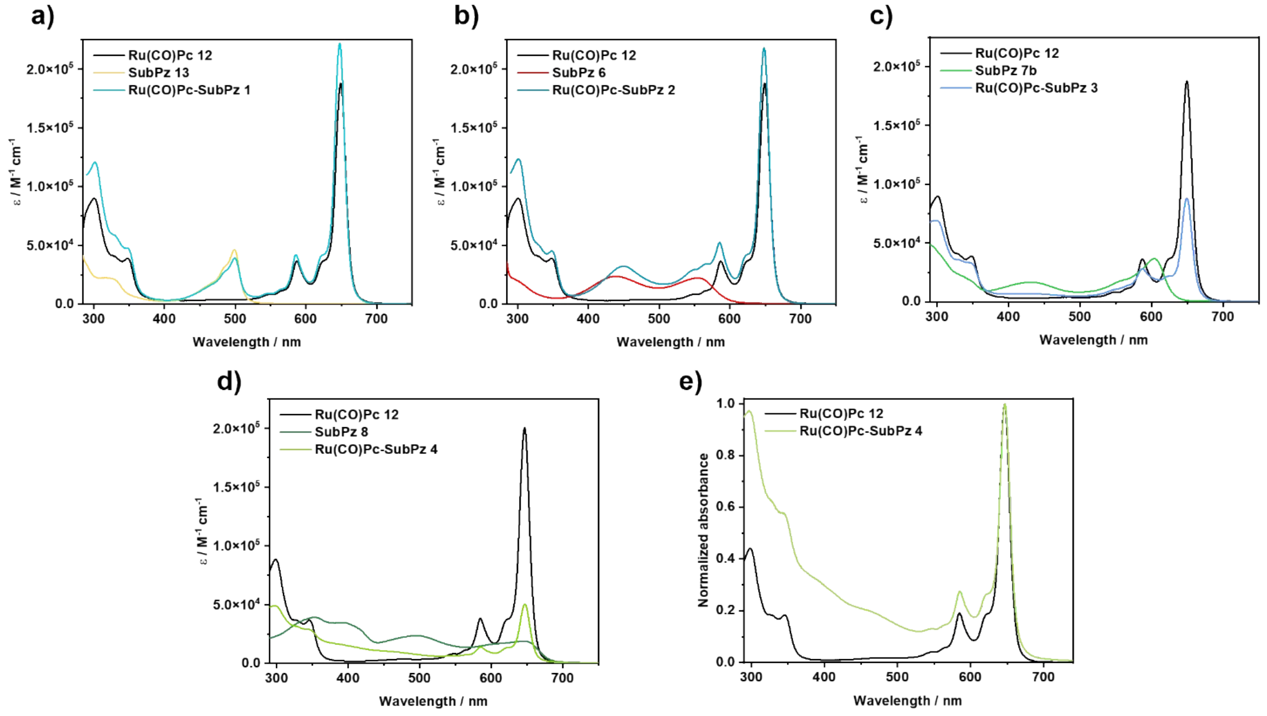

Figure S39. Fluorescence spectra of SubPz **13**, Ru(CO)Pc-SubPz **1** and Ru(CO)Pc **12** samples measured in (a,b) toluene and (c,d) THF at room temperature, by exciting at (a,c) 470 nm and (b,d) 590 nm.

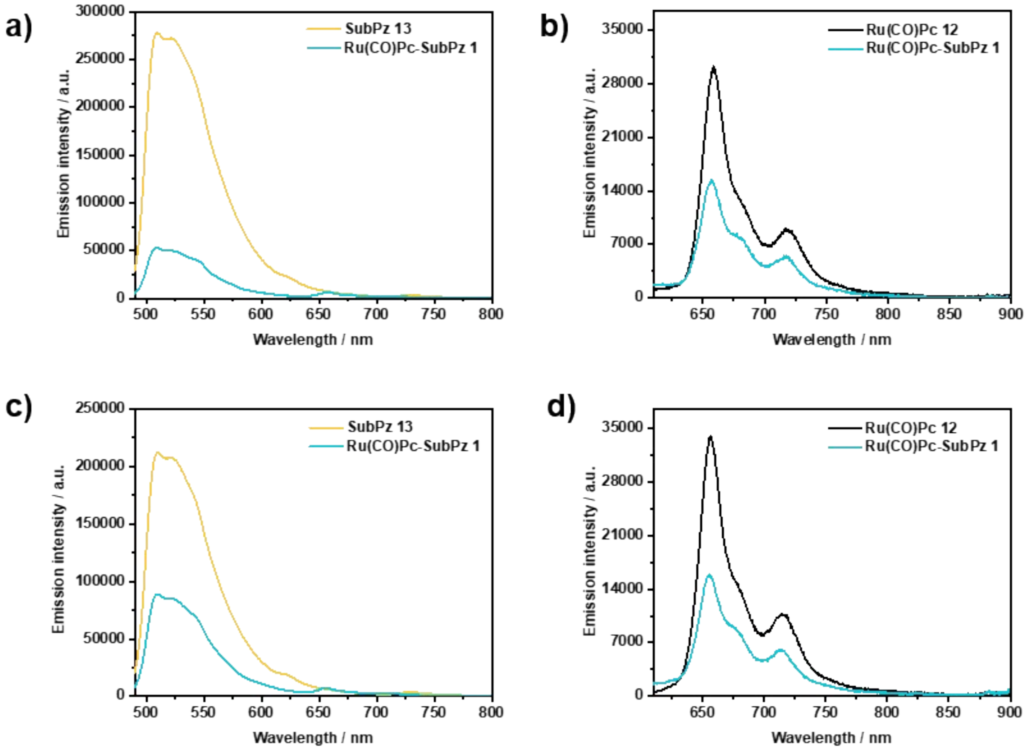

Figure S40. Fluorescence spectra of SubPz **6**, Ru(CO)Pc-SubPz **2** and Ru(CO)Pc **12** samples measured in (a,b,c) toluene and (d,e,f) THF at room temperature, by exciting at (a,d) 400 nm, (b,e) 440 nm and (c,f) 590 nm.

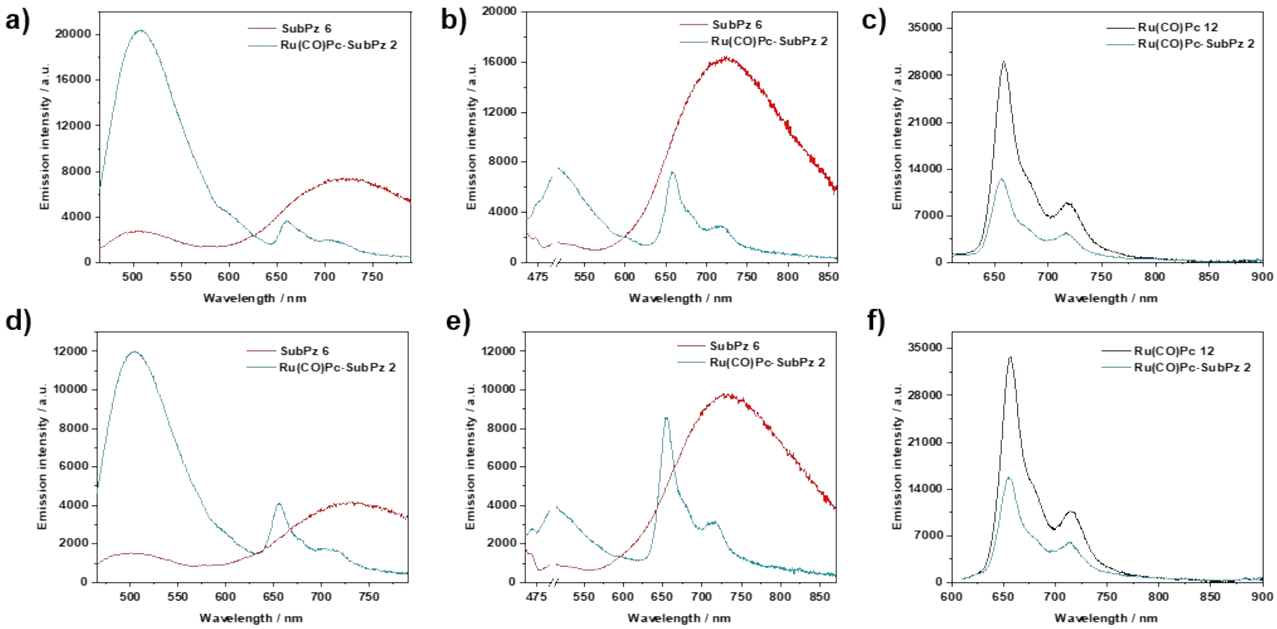

Figure S41. Fluorescence spectra of SubPz **7b**, Ru(CO)Pc-SubPz **3** and Ru(CO)Pc **12** samples measured in (a,b) toluene and (c,d) THF at room temperature, by exciting at (a,c) 470 nm and (b,d) 590 nm.

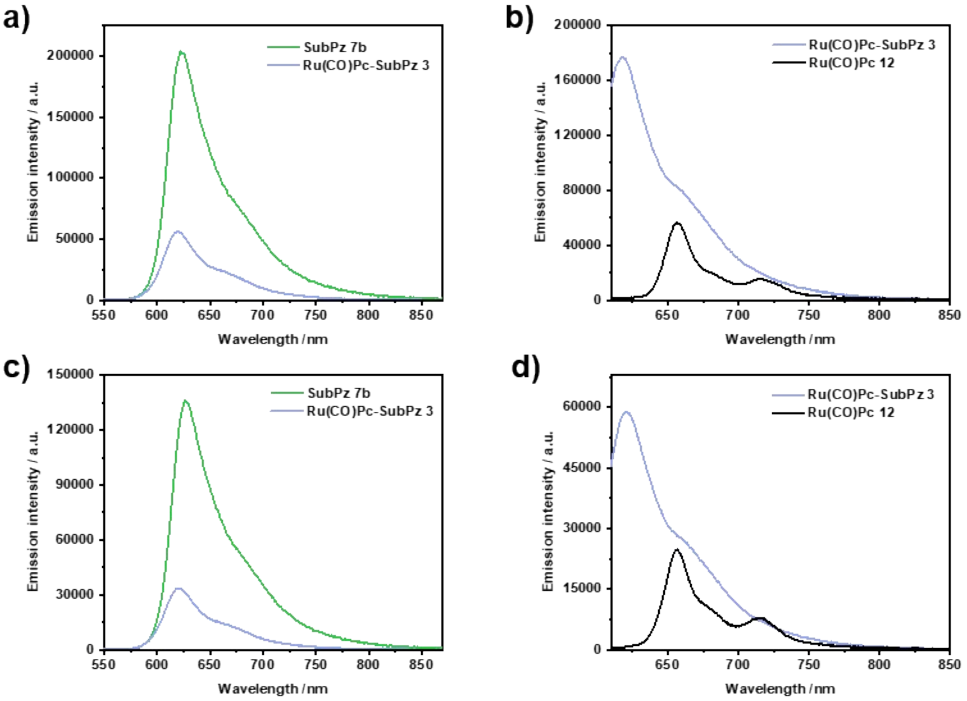

Figure S42. Fluorescence spectra of SubPz **8**, Ru(CO)Pc-SubPz **4** and Ru(CO)Pc **12** samples measured in THF at room temperature, by exciting at (a) 440 nm and (b) 590 nm.

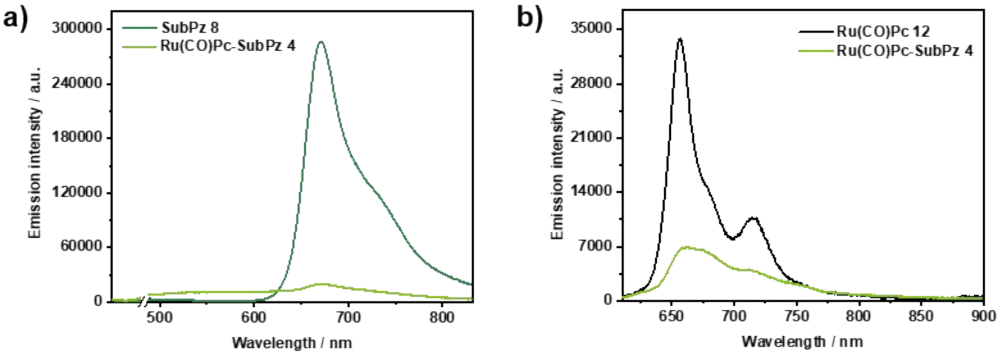

6. Spectroelectrochemical data

Figure S43. Absorption spectra as well as the differential absorption spectrum recorded upon oxidation of Ru(CO)Pc **12** in toluene:acetonitrile (10:1) mixture (0.1M $TBAPF_6$ as electrolyte).

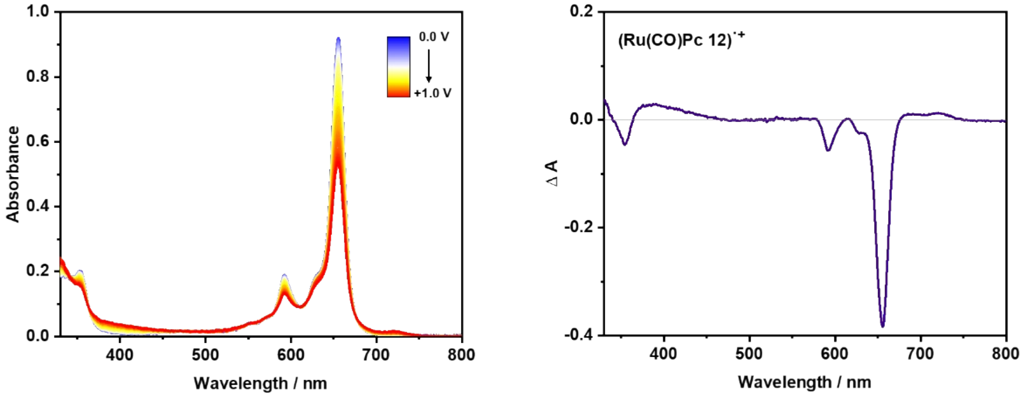

Figure S44. Absorption spectra as well as the differential absorption spectrum recorded upon reduction of SubPz **6** in toluene:acetonitrile (10:1) mixture (0.1M $TBAPF_6$ as electrolyte).

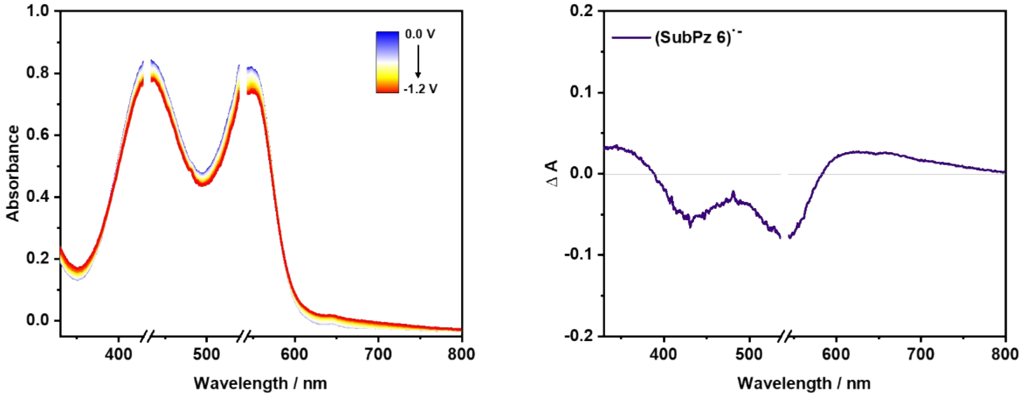

Figure S45. Absorption spectra as well as the differential absorption spectrum recorded upon reduction of SubPz **7b** in toluene:acetonitrile (10:1) mixture (0.1M $TBAPF_6$ as electrolyte).

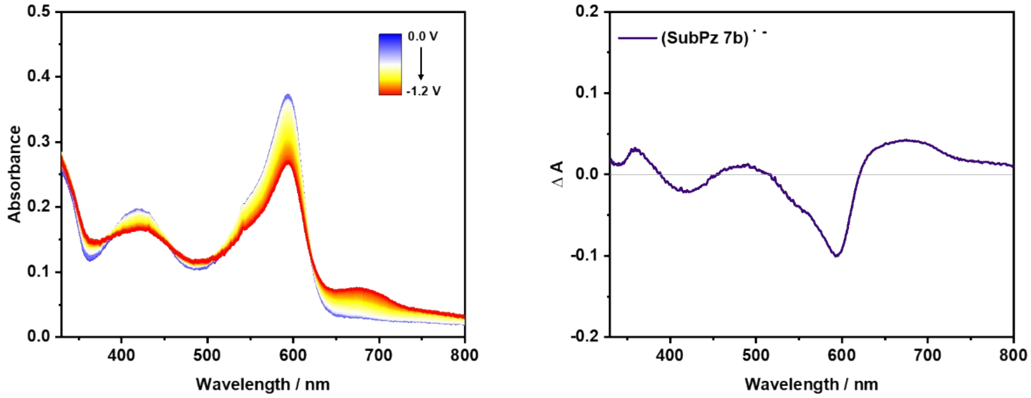

Figure S46. Absorption spectra as well as the differential absorption spectrum recorded upon reduction of SubPz **8** in THF (0.1M $TBAPF_6$ as electrolyte).

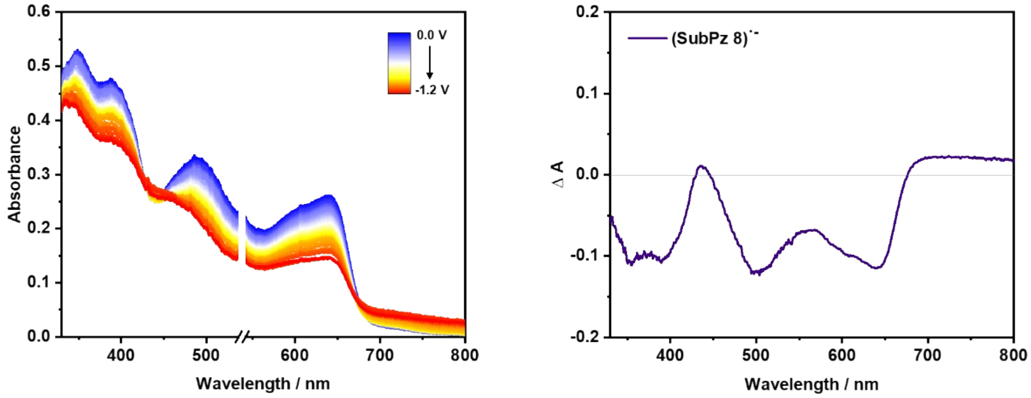

7. Transient absorption studies

Figure S47. fs-TAS raw data from pump-probe experiments and the corresponding global sequential analysis for SubPz **13**, following 480 nm photoexcitation in argon-saturated toluene at room temperature. (a) Heat map of fs-TAS raw data. (b) Differential absorption spectra at time delays between 0 ps and 7.5 ns. (c) Evolution-associated spectra with their corresponding lifetimes, obtained from the deconvolution of the fs-TAS data. (d) Relative populations of the respective species.

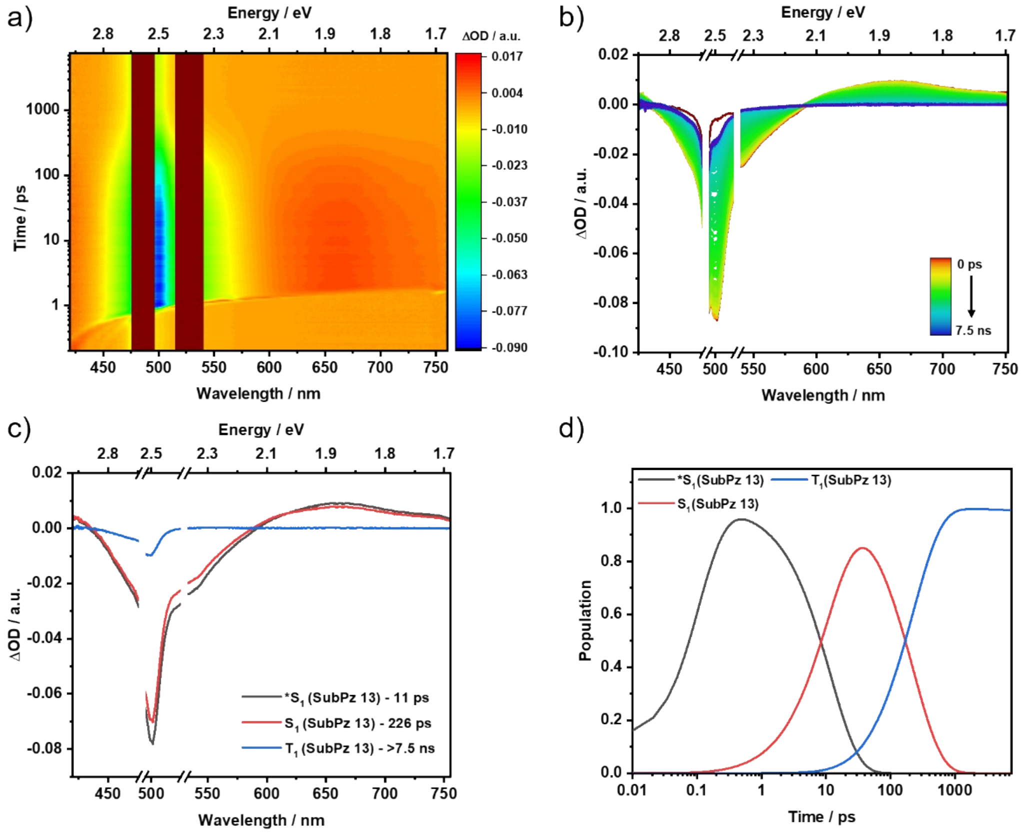

Figure S48. ns-TAS raw data from pump-probe experiments and the corresponding global sequential analysis for SubPz **13**, following 480 nm photoexcitation in argon-saturated toluene at room temperature. (a) Heat map of ns-TAS raw data. (b) Differential absorption spectra at time delays between 1 ns and 400 µs. (c) Evolution-associated spectrum with the corresponding lifetime, obtained from the deconvolution of the ns-TAS data. (d) Relative population of the deconvoluted species.

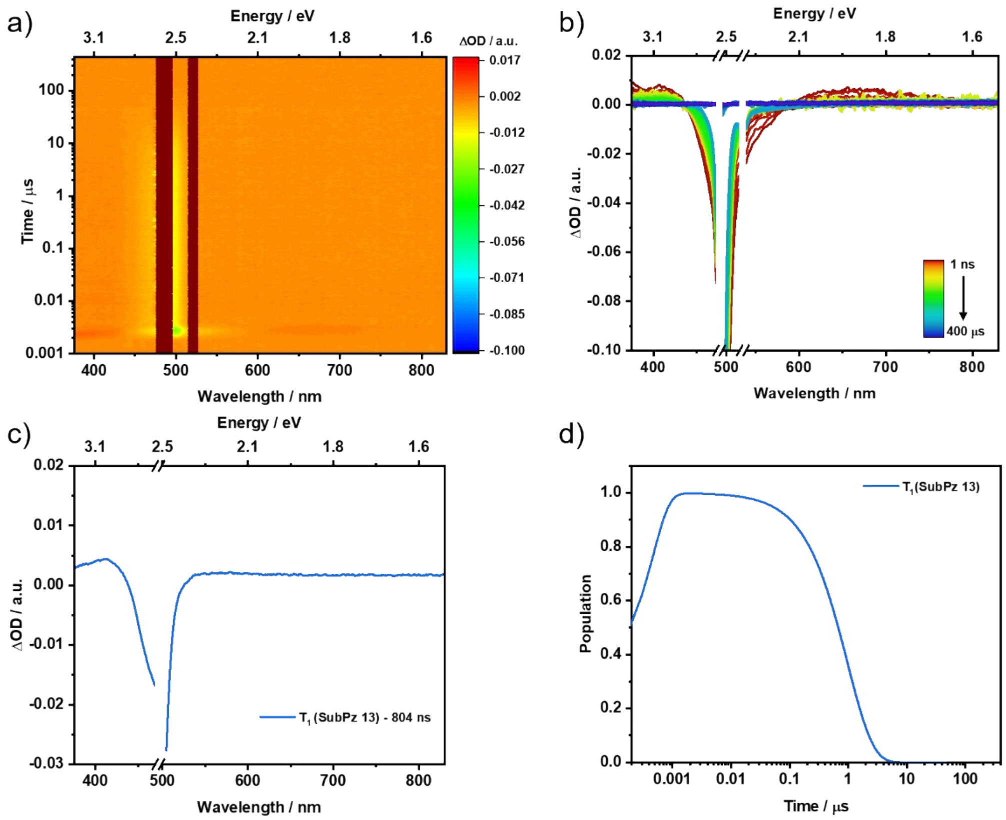

Figure S49. fs-TAS raw data from pump-probe experiments and the corresponding global sequential analysis for SubPz **6**, following 480 nm photoexcitation in argon-saturated toluene at room temperature. (a) Heat map of fs-TAS raw data. (b) Differential absorption spectra at time delays between 0 ps and 7.5 ns. (c) Evolution-associated spectra with their corresponding lifetimes, obtained from the deconvolution of the fs-TAS data. (d) Relative populations of the respective species.

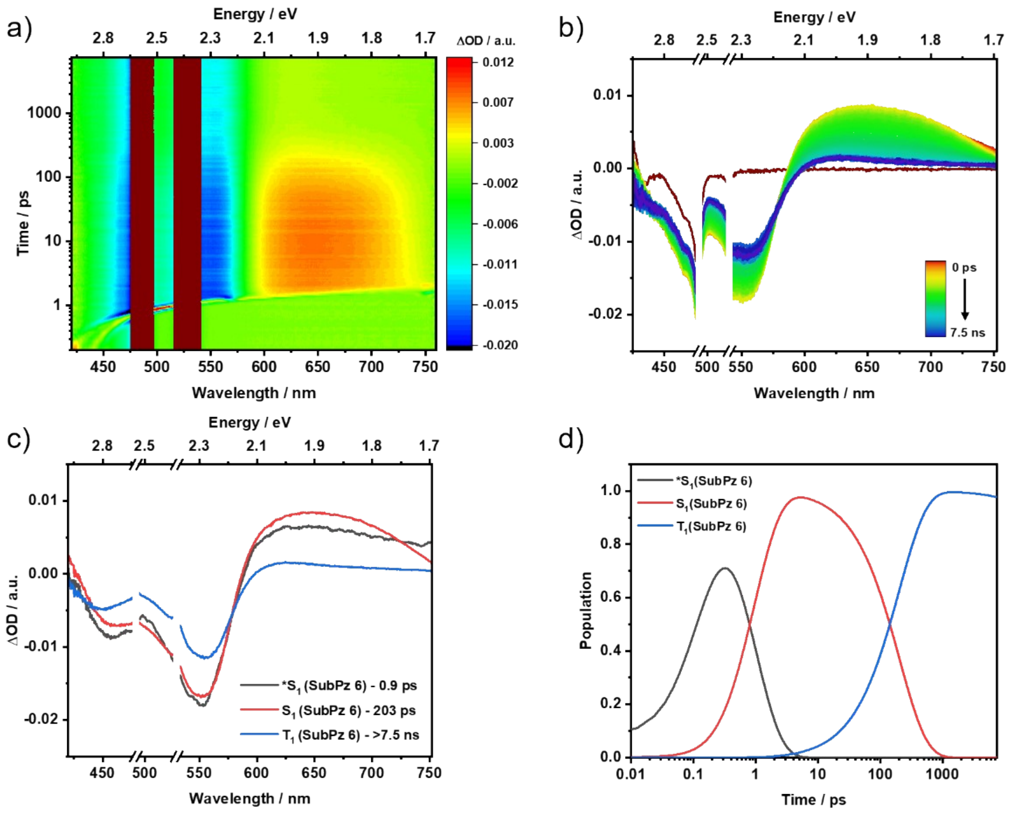

Figure S50. ns-TAS raw data from pump-probe experiments and the corresponding global sequential analysis for SubPz **6**, following 480 nm photoexcitation in argon-saturated toluene at room temperature. (a) Heat map of ns-TAS raw data. (b) Differential absorption spectra at time delays between 1 ns and 400 μs. (c) Evolution-associated spectrum with the corresponding lifetime, obtained from the deconvolution of the ns-TAS data. (d) Relative population of the deconvoluted species.

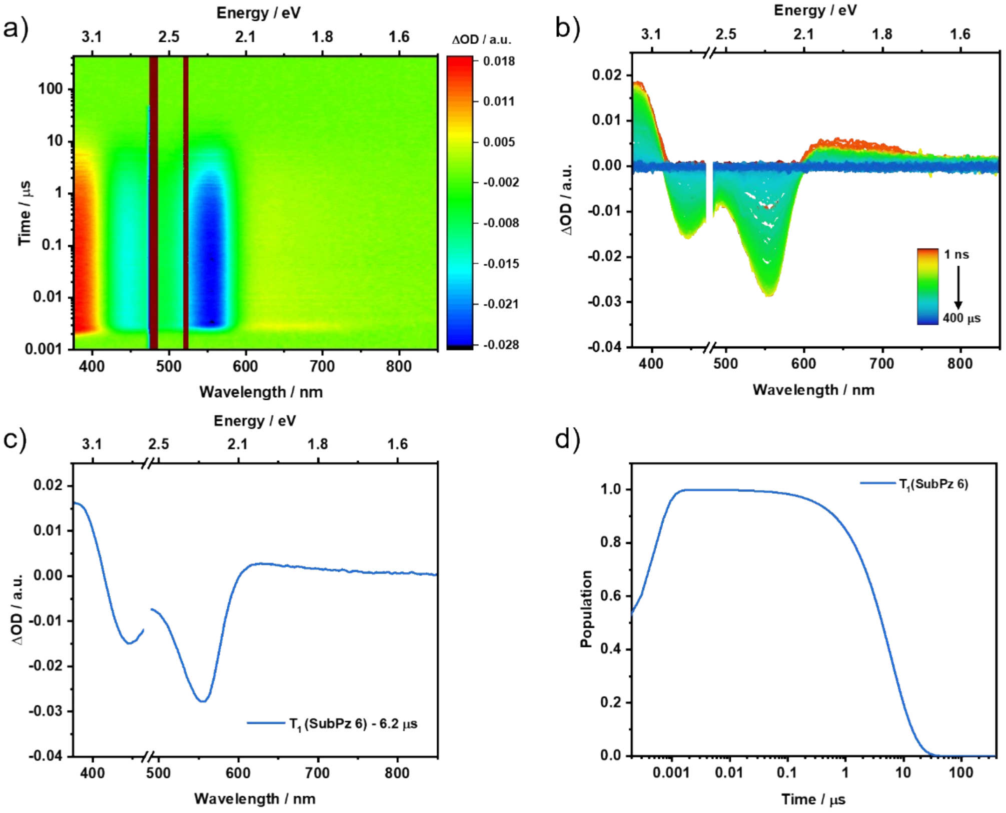

Figure S51. fs-TAS raw data from pump-probe experiments and the corresponding global sequential analysis for SubPz **7b**, following 480 nm photoexcitation in argon-saturated toluene at room temperature. (a) Heat map of fs-TAS raw data. (b) Differential absorption spectra at time delays between 0 ps and 7.5 ns. (c) Evolution-associated spectra with their corresponding lifetimes, obtained from the deconvolution of the fs-TAS data. (d) Relative populations of the respective species.

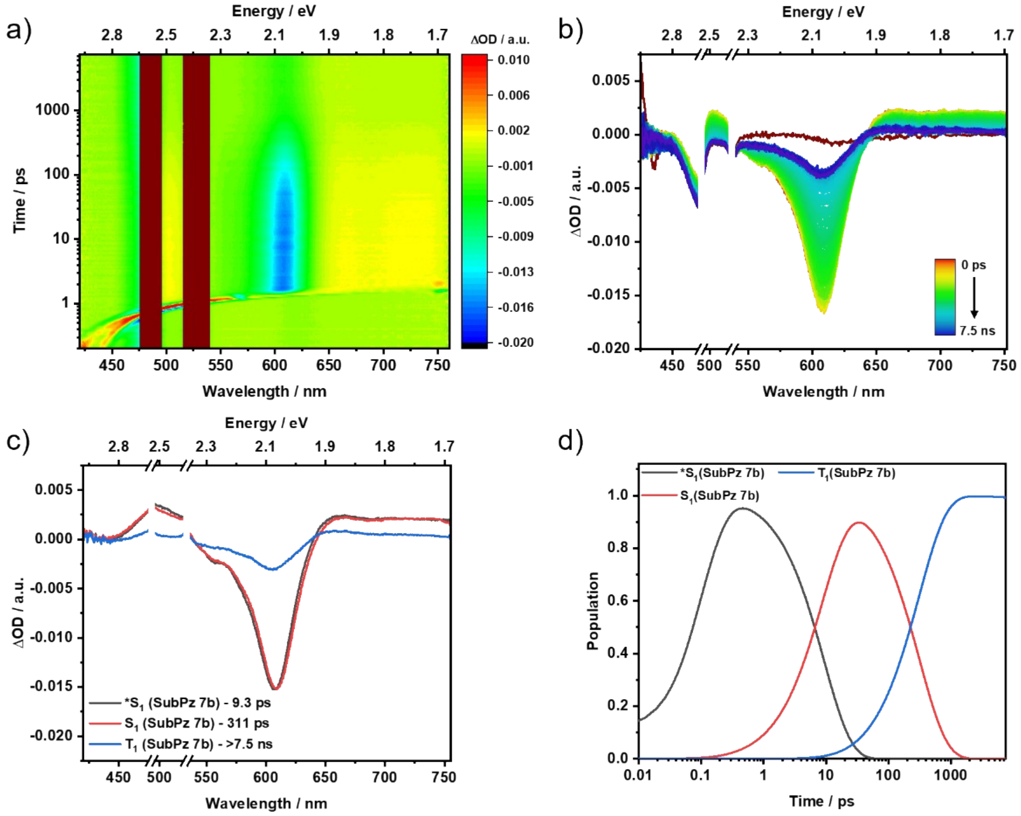

Figure S52. ns-TAS raw data from pump-probe experiments and the corresponding global sequential analysis for SubPz **7b**, following 480 nm photoexcitation in argon-saturated toluene at room temperature. (a) Heat map of ns-TAS raw data. (b) Differential absorption spectra at time delays between 1 ns and 350 µs. (c) Evolution-associated spectrum with the corresponding lifetime, obtained from the deconvolution of the ns-TAS data. (d) Relative population of the deconvoluted species.

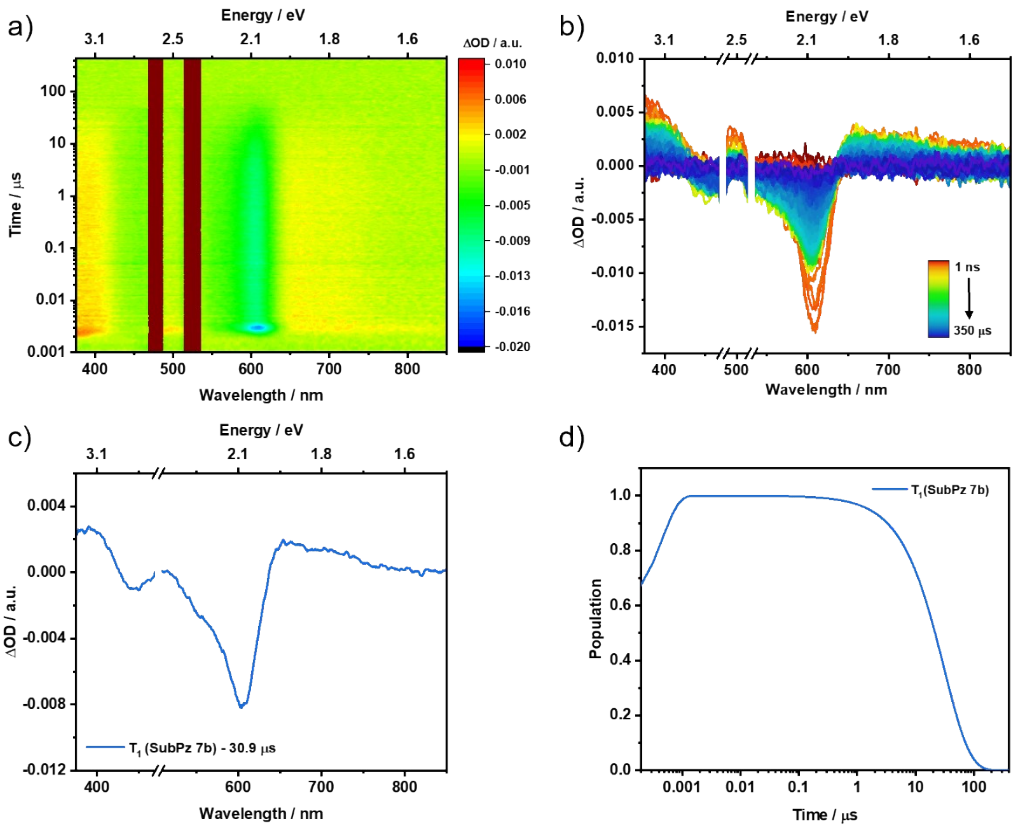

Figure S53. fs-TAS raw data from pump-probe experiments and the corresponding global sequential analysis for SubPz **8**, following 480 nm photoexcitation in argon-saturated THF at room temperature. (a) Heat map of fs-TAS raw data. (b) Differential absorption spectra at time delays between 0 ps and 7.5 ns. (c) Evolution-associated spectra with their corresponding lifetimes, obtained from the deconvolution of the fs-TAS data. (d) Relative populations of the respective species.

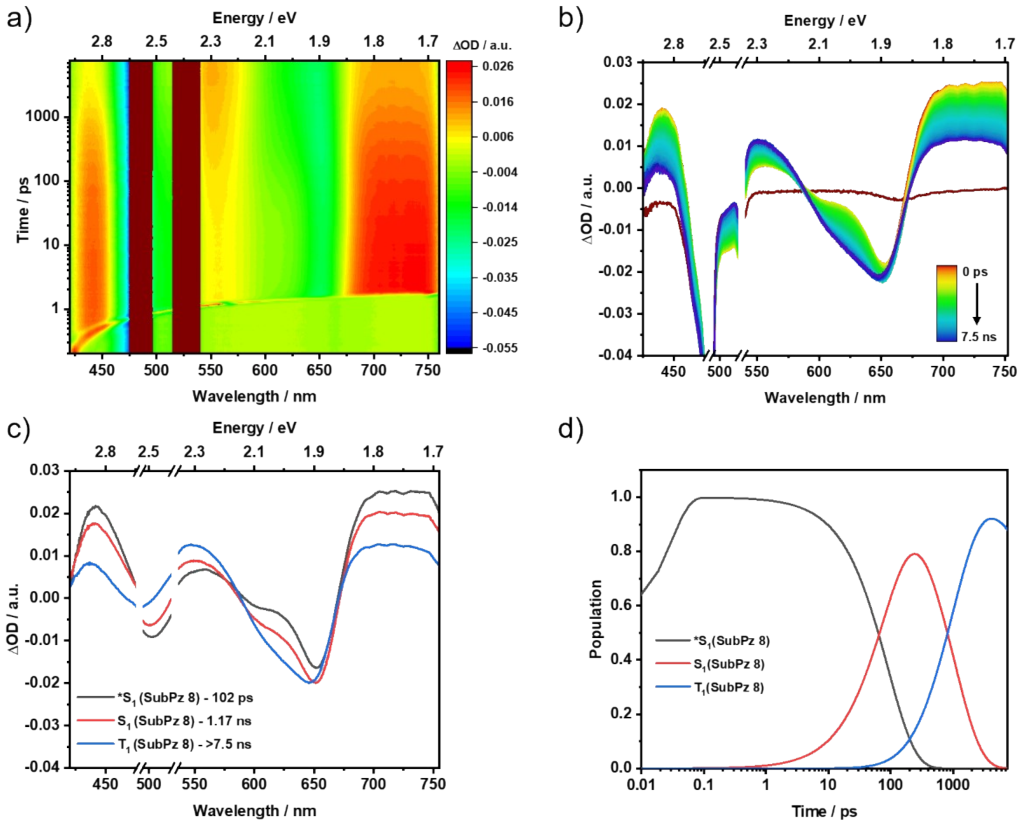

Figure S54. ns-TAS raw data from pump-probe experiments and the corresponding global sequential analysis for SubPz **8**, following 480 nm photoexcitation in argon-saturated THF at room temperature. (a) Heat map of ns-TAS raw data. (b) Differential absorption spectra at time delays between 1 ns and 350 µs. (c) Evolution-associated spectra with their corresponding lifetimes, obtained from the deconvolution of the ns-TAS data. (d) Relative population of the respective species.

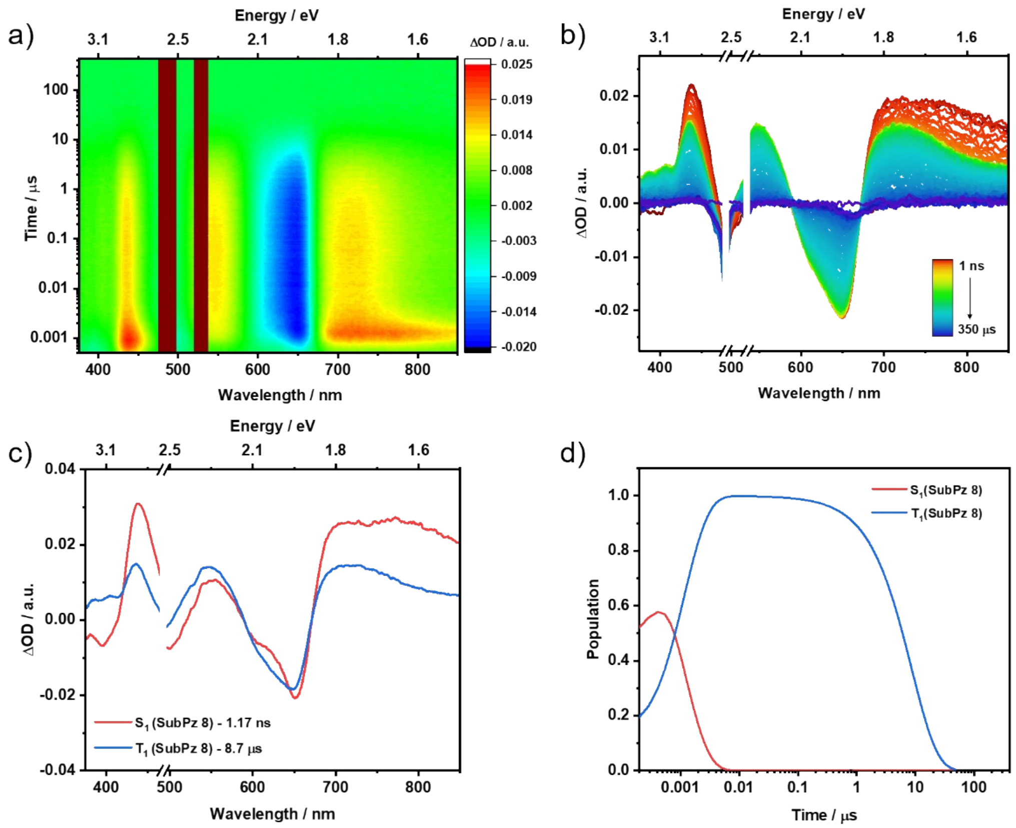

Figure S55. fs-TAS raw data from pump-probe experiments and the corresponding global sequential analysis for Ru(CO)Pc **12**, following 660 nm photoexcitation in argon-saturated toluene at room temperature. (a) Heat map of fs-TAS raw data. (b) Differential absorption spectra at time delays between 0 ps and 7.5 ns. (c) Evolution-associated spectra with their corresponding lifetimes, obtained from the deconvolution of the fs-TAS data. (d) Relative populations of the respective species.

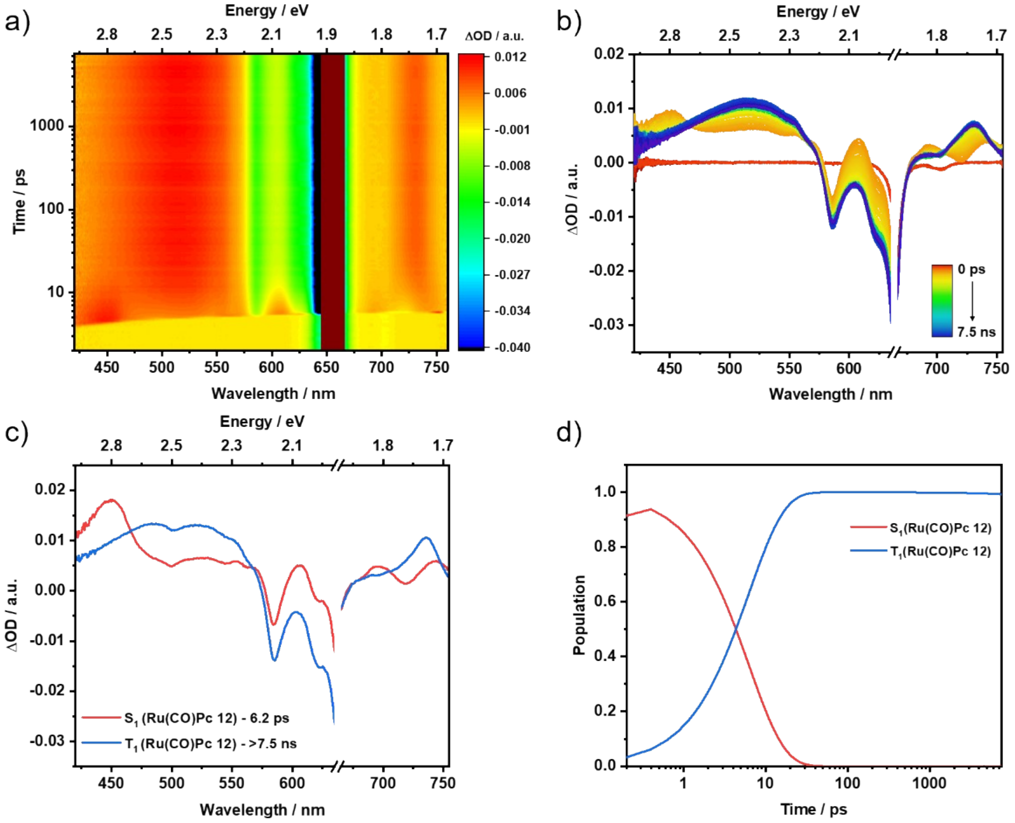

Figure S56. ns-TAS raw data from pump-probe experiments and the corresponding global sequential analysis for Ru(CO)Pc **12**, following 660 nm photoexcitation in argon-saturated toluene at room temperature. (a) Heat map of ns-TAS raw data. (b) Differential absorption spectra at time delays between 1 ns and 350 µs. (c) Evolution-associated spectrum with the corresponding lifetime, obtained from the deconvolution of the ns-TAS data. (d) Relative population of the deconvoluted species.

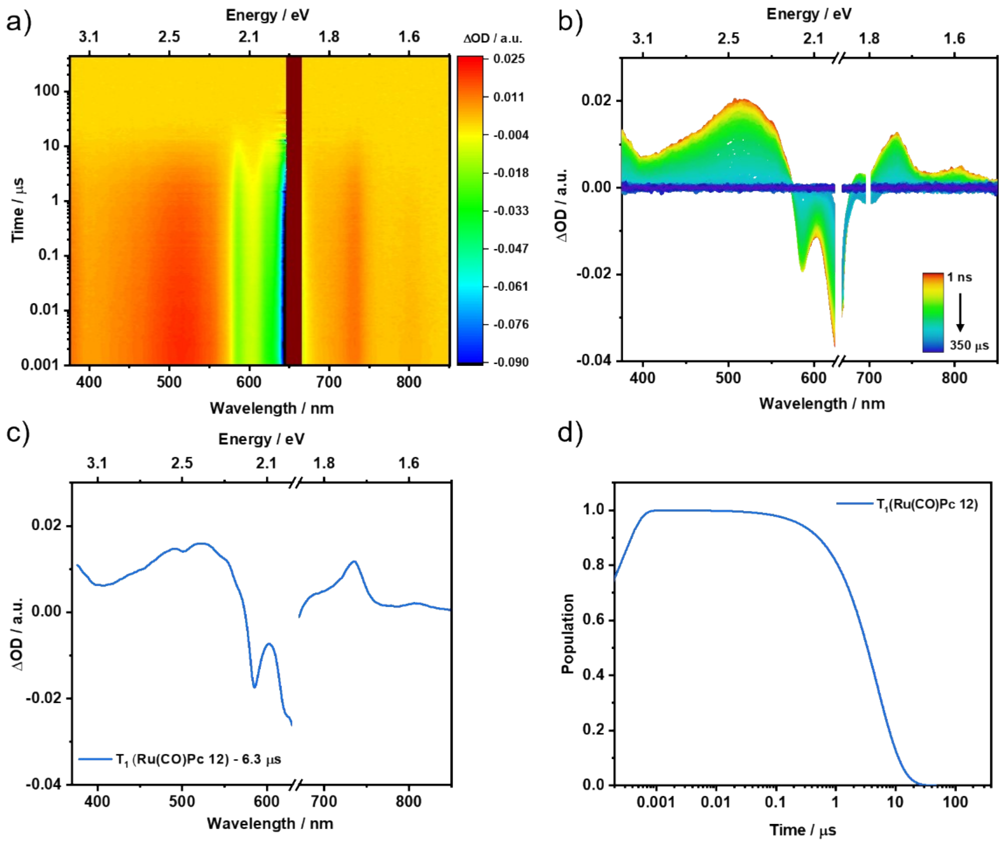

Figure S57. fs-TAS raw data from pump-probe experiments and the corresponding global sequential analysis for Ru(CO)Pc **12**, following 660 nm photoexcitation in argon-saturated THF at room temperature. (a) Heat map of fs-TAS raw data. (b) Differential absorption spectra at time delays between 0 ps and 7.5 ns. (c) Evolution-associated spectra with their corresponding lifetimes, obtained from the deconvolution of the fs-TAS data. (d) Relative populations of the respective species.

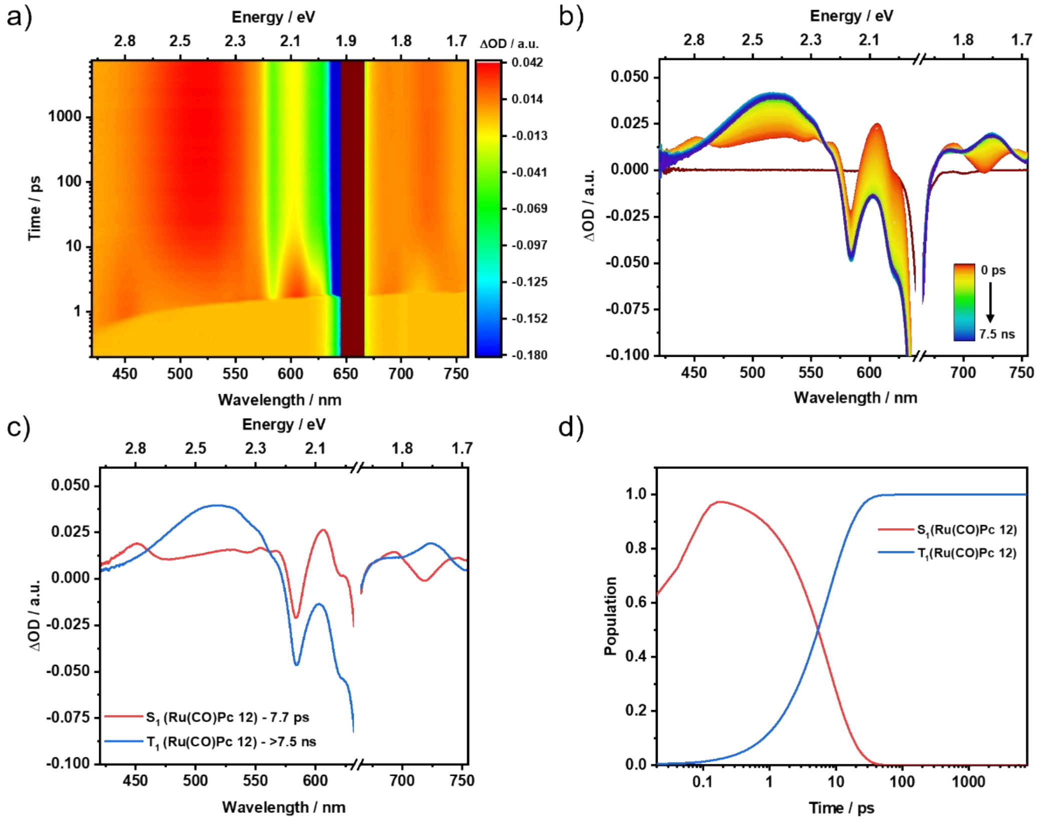

Figure S58. ns-TAS raw data from pump-probe experiments and the corresponding global sequential analysis for Ru(CO)Pc **12**, following 660 nm photoexcitation in argon-saturated THF at room temperature. (a) Heat map of ns-TAS raw data. (b) Differential absorption spectra at time delays between 1 ns and 350 µs. (c) Evolution-associated spectrum with the corresponding lifetime, obtained from the deconvolution of the ns-TAS data. (d) Relative population of the deconvoluted species.

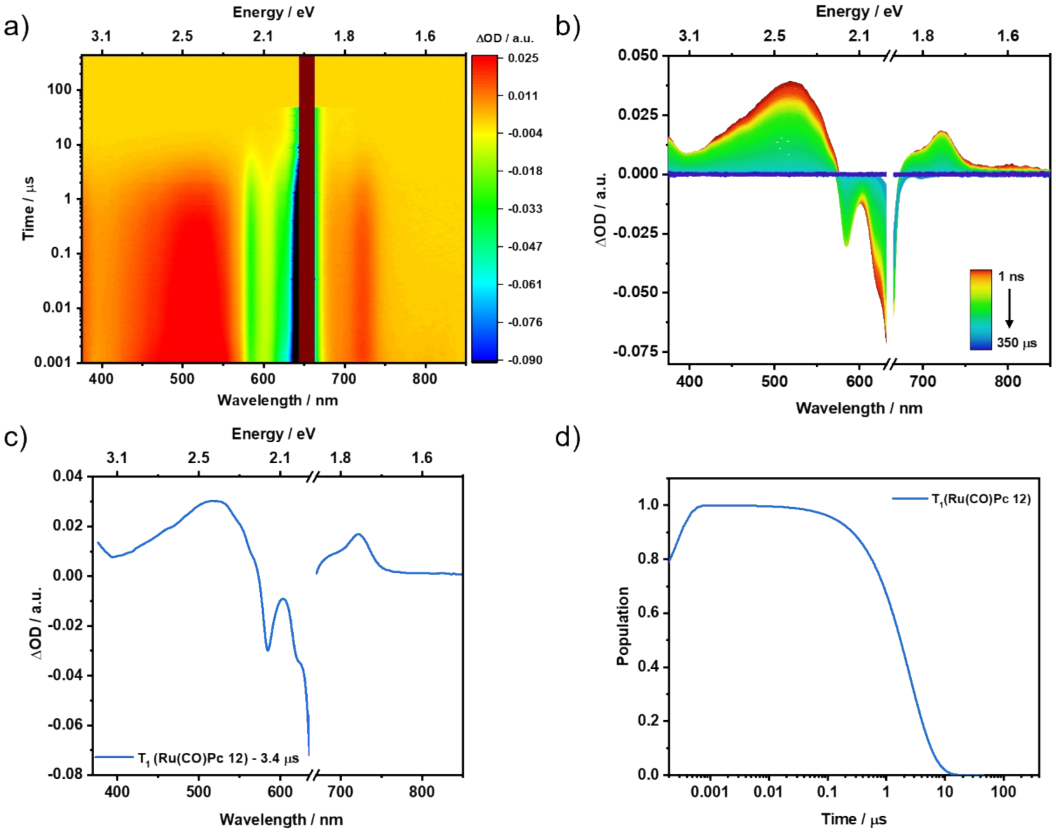

Figure S59. ns-TAS raw data from pump-probe experiments and the corresponding global sequential analysis for conjugate Ru(CO)Pc-SubPz **1**, following 480 nm photoexcitation in argon-saturated toluene at room temperature. (a) Heat map of ns-TAS raw data. (b) Differential absorption spectra at time delays between 1 ns and 350 µs. (c) Evolution-associated spectrum with the corresponding lifetime, obtained from the deconvolution of the ns-TAS data. (d) Relative population of the deconvoluted species.

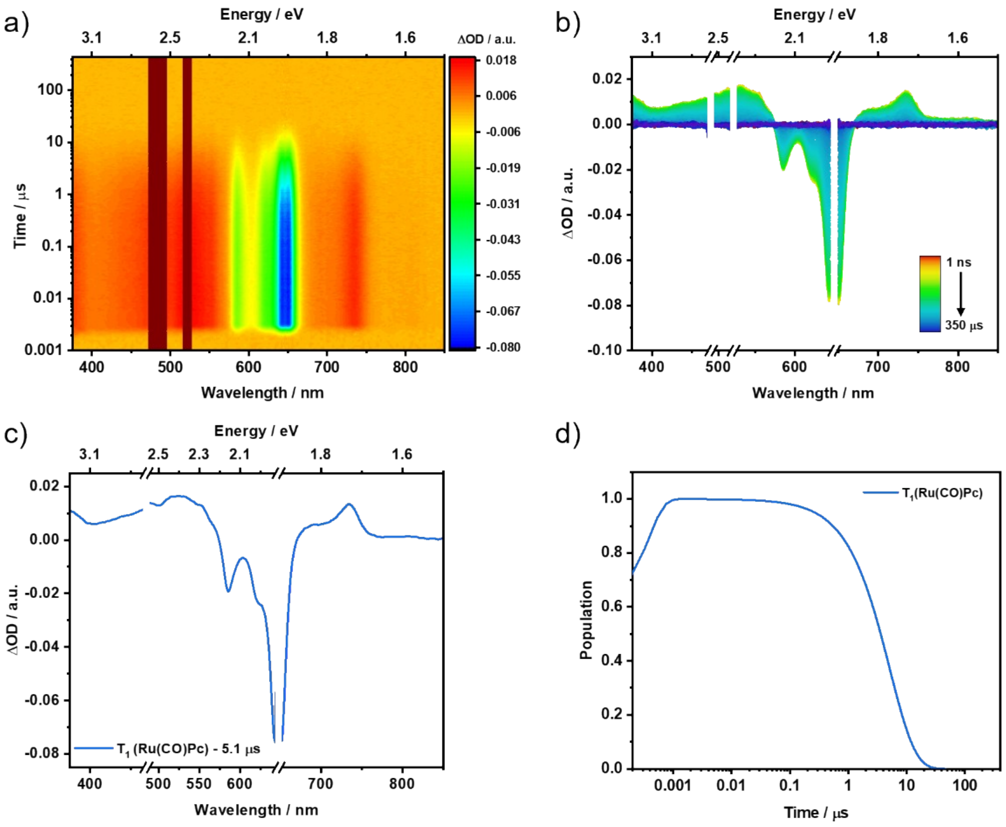

Figure S60. ns-TAS raw data from pump-probe experiments and the corresponding global sequential analysis for conjugate Ru(CO)Pc-SubPz **2**, following 480 nm photoexcitation in argon-saturated toluene at room temperature. (a) Heat map of ns-TAS raw data. (b) Differential absorption spectra at time delays between 1 ns and 350 µs. (c) Evolution-associated spectra with their corresponding lifetimes, obtained from the deconvolution of the ns-TAS data. (d) Relative populations of the respective species.

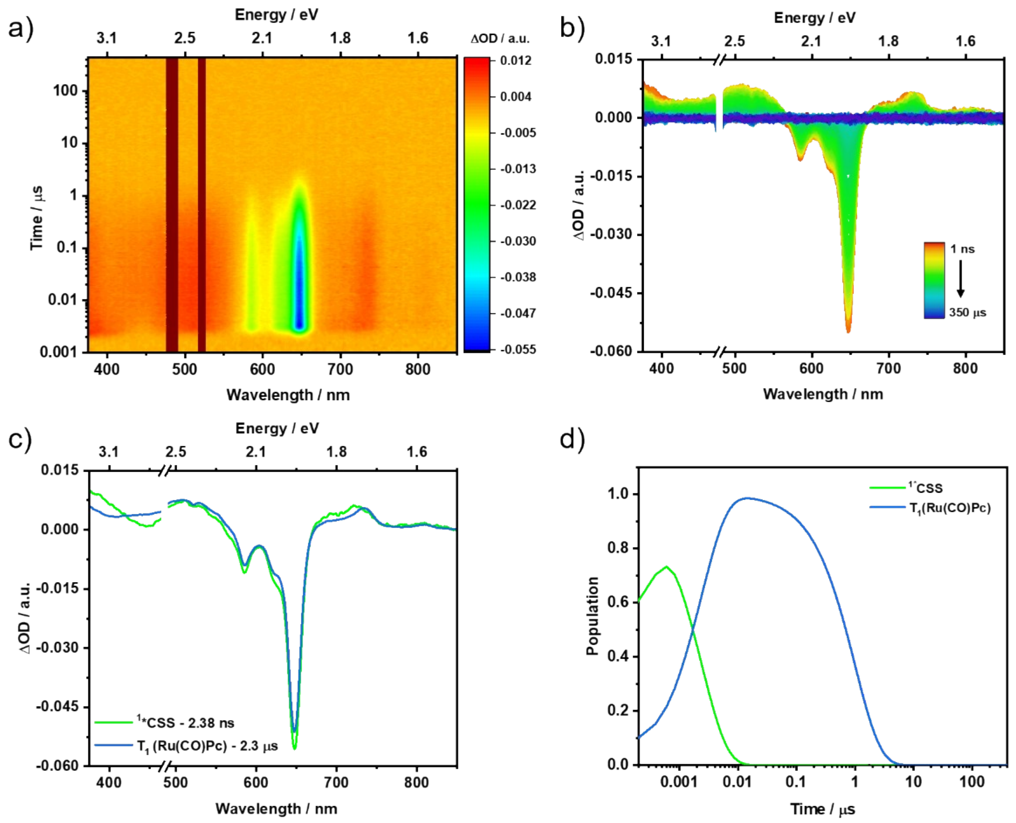

Figure S61. ns-TAS raw data from pump-probe experiments and the corresponding global target analysis for conjugate Ru(CO)Pc-SubPz **3**, following 480 nm photoexcitation in argon-saturated toluene at room temperature. (a) Heat map of ns-TAS raw data. (b) Differential absorption spectra at time delays between 1 ns and 350 μs. (c) Species-associated spectra with their corresponding lifetimes, obtained from the deconvolution of the ns-TAS data. (d) Relative population of the respective species.

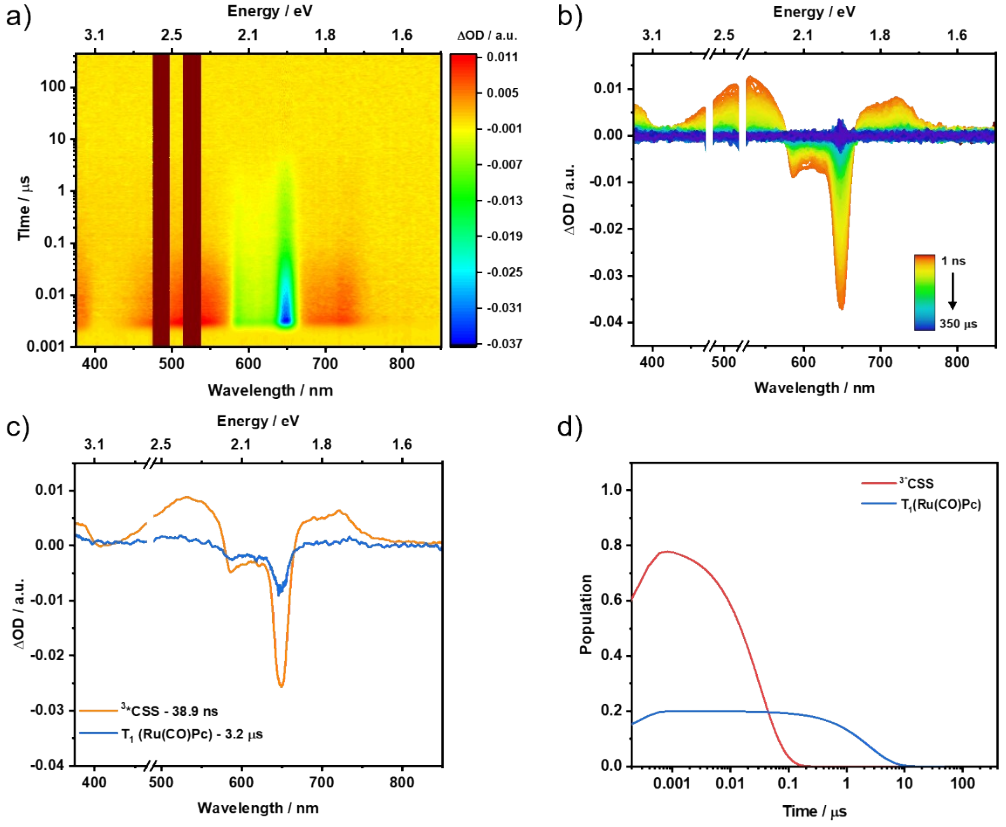

Figure S62. ns-TAS raw data from pump-probe experiments and the corresponding global sequential analysis for conjugate Ru(CO)Pc-SubPz **4**, following 480 nm photoexcitation in argon-saturated THF at room temperature. (a) Heat map of ns-TAS raw data. (b) Differential absorption spectra at time delays between 1 ns and 350 μs. (c) Evolution-associated spectra with their corresponding lifetimes, obtained from the deconvolution of the ns-TAS data. (d) Relative population of the respective species.

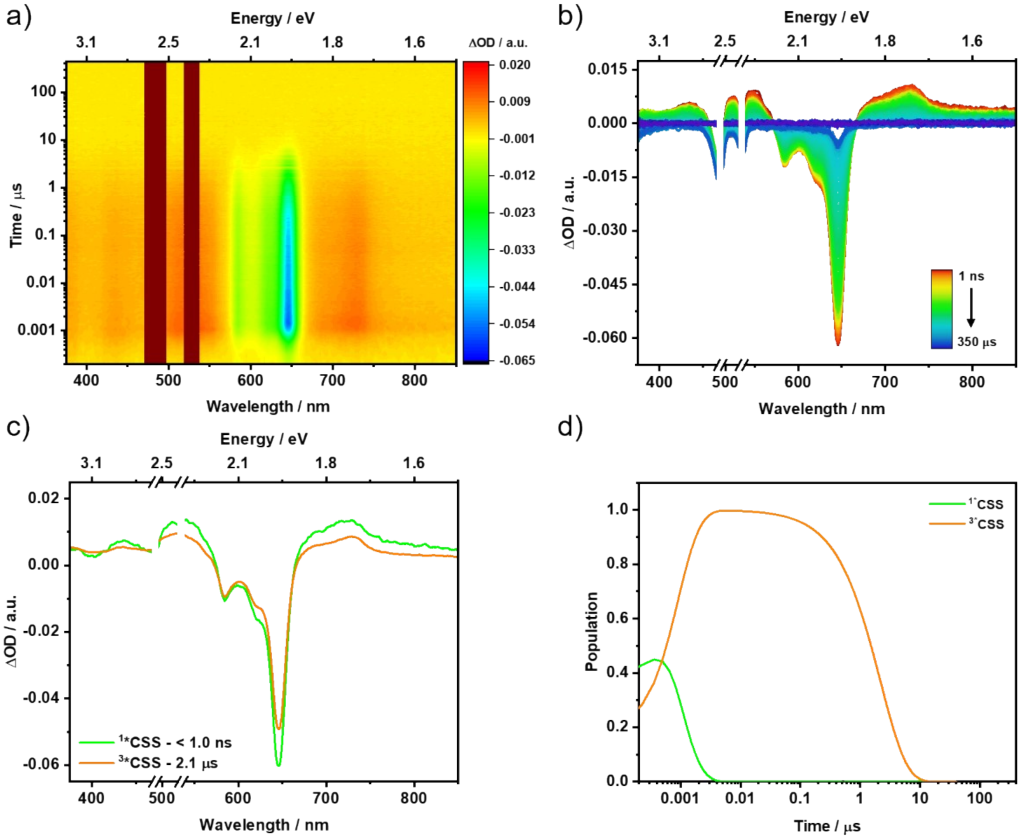

Figure S63. fs-TAS raw data from pump-probe experiments and the corresponding global sequential analysis for conjugate Ru(CO)Pc-SubPz **1**, following 660 nm photoexcitation in argon-saturated toluene at room temperature. (a) Heat map of fs-TAS raw data. (b) Differential absorption spectra at time delays between 0 ps and 7.5 ns. (c) Evolution-associated spectra with their corresponding lifetimes, obtained from the deconvolution of the fs-TAS data. (d) Relative populations of the respective species.

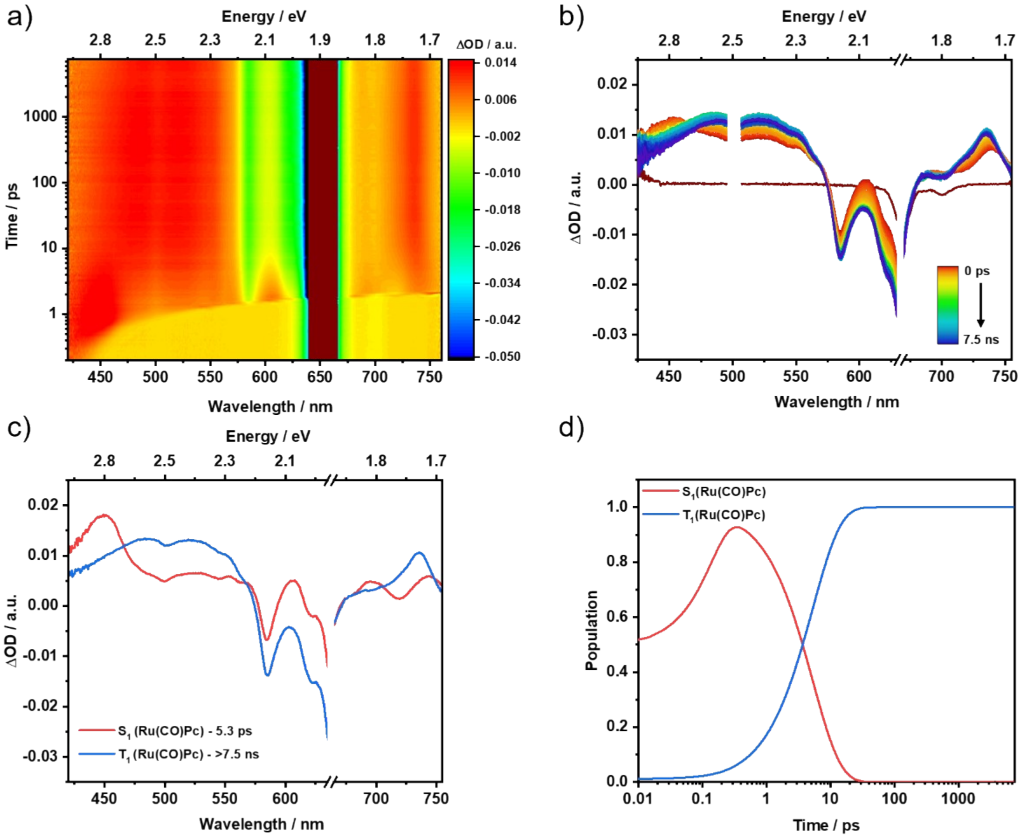

Figure S64. ns-TAS raw data from pump-probe experiments and the corresponding global sequential analysis for conjugate Ru(CO)Pc-SubPz **1**, following 660 nm photoexcitation in argon-saturated toluene at room temperature. (a) Heat map of ns-TAS raw data. (b) Differential absorption spectra at time delays between 1 ns and 350 µs. (c) Evolution-associated spectrum with the corresponding lifetime, obtained from the deconvolution of the ns-TAS data. (d) Relative population of the deconvoluted species.

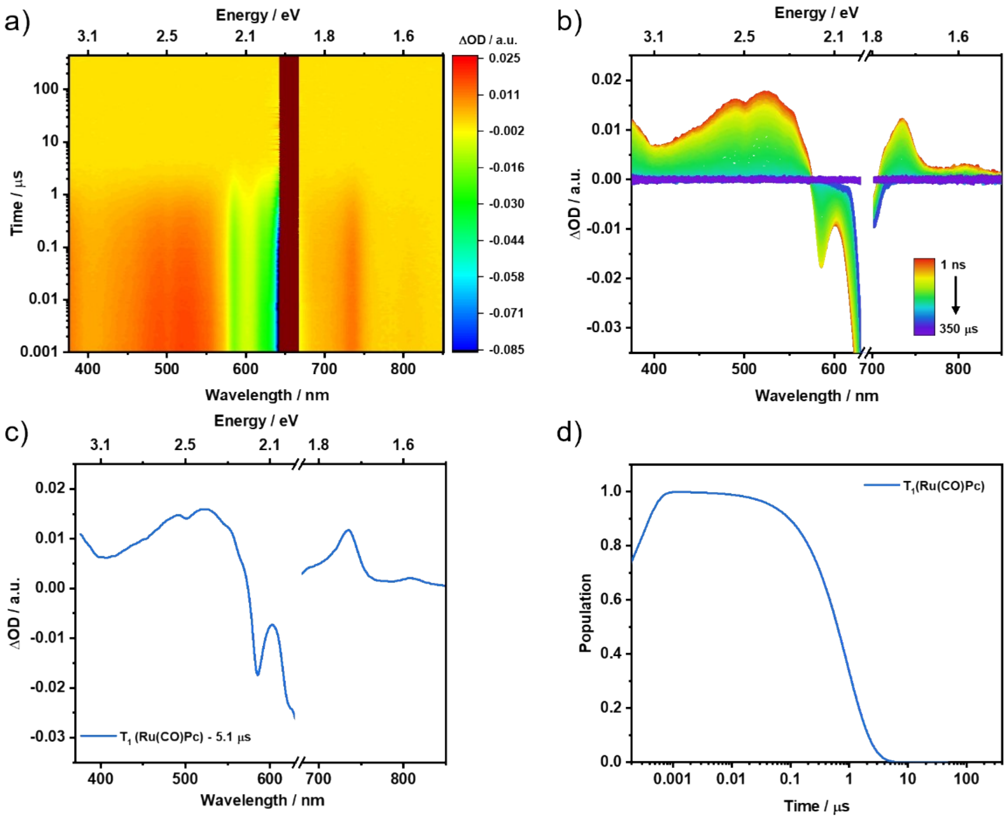

Figure S65. fs-TAS raw data from pump-probe experiments and the corresponding global sequential analysis for conjugate Ru(CO)Pc-SubPz **2**, following 660 nm photoexcitation in argon-saturated toluene at room temperature. (a) Heat map of fs-TAS raw data. (b) Differential absorption spectra at time delays between 0 ps and 7.5 ns. (c) Evolution-associated spectra with their corresponding lifetimes, obtained from the deconvolution of the fs-TAS data. (d) Relative populations of the respective species.

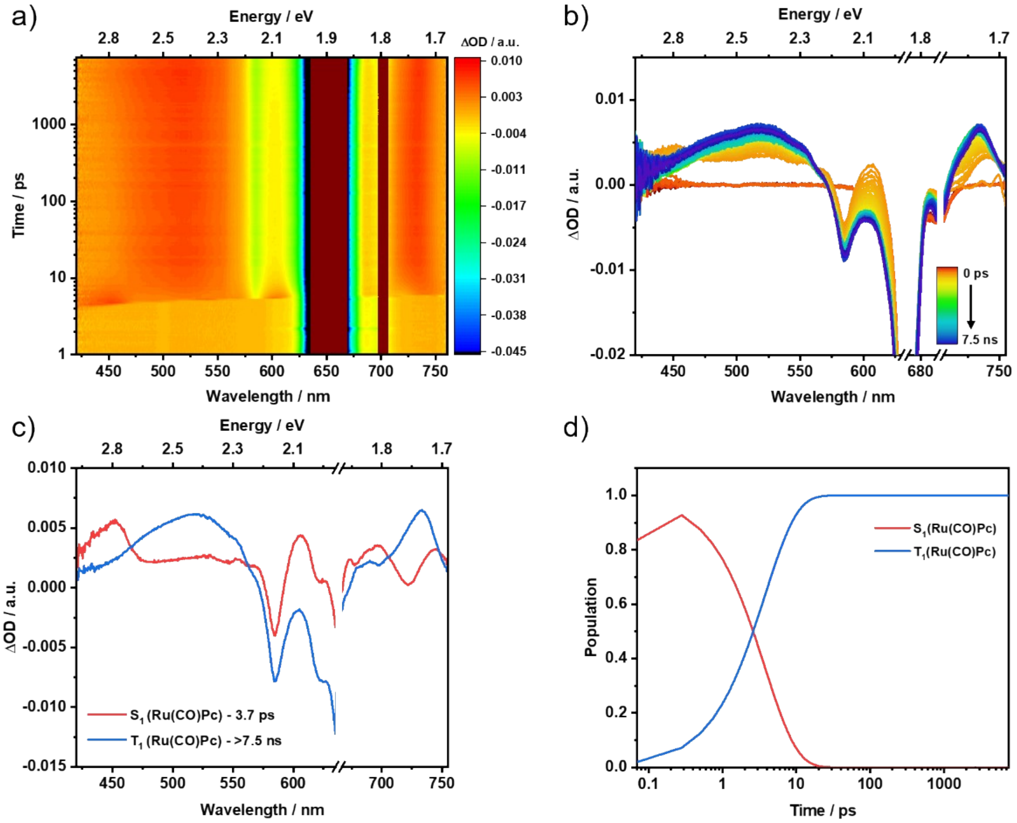

Figure S66. ns-TAS raw data from pump-probe experiments and the corresponding global sequential analysis for conjugate Ru(CO)Pc-SubPz **2**, following 660 nm photoexcitation in argon-saturated toluene at room temperature. (a) Heat map of ns-TAS raw data. (b) Differential absorption spectra at time delays between 1 ns and 350 µs. (c) Evolution-associated spectrum with the corresponding lifetime, obtained from the deconvolution of the ns-TAS data. (d) Relative population of the deconvoluted species.

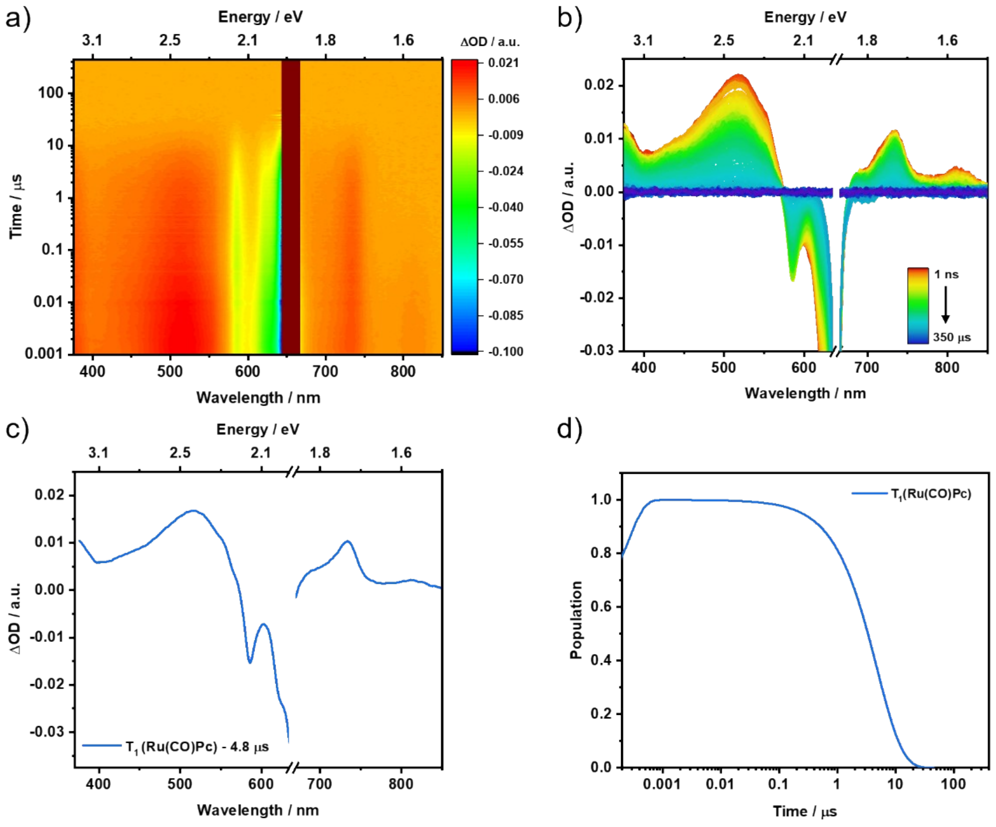

Figure S67. fs-TAS raw data from pump-probe experiments and the corresponding target analysis for conjugate Ru(CO)Pc-SubPz **3**, following 660 nm photoexcitation in argon-saturated toluene at room temperature. (a) Heat map of fs-TAS raw data. (b) Differential absorption spectra at time delays between 0 ps and 7.5 ns. (c) Evolution-associated spectra with their corresponding lifetimes, obtained from the deconvolution of the fs-TAS data. (d) Relative populations of the respective species.

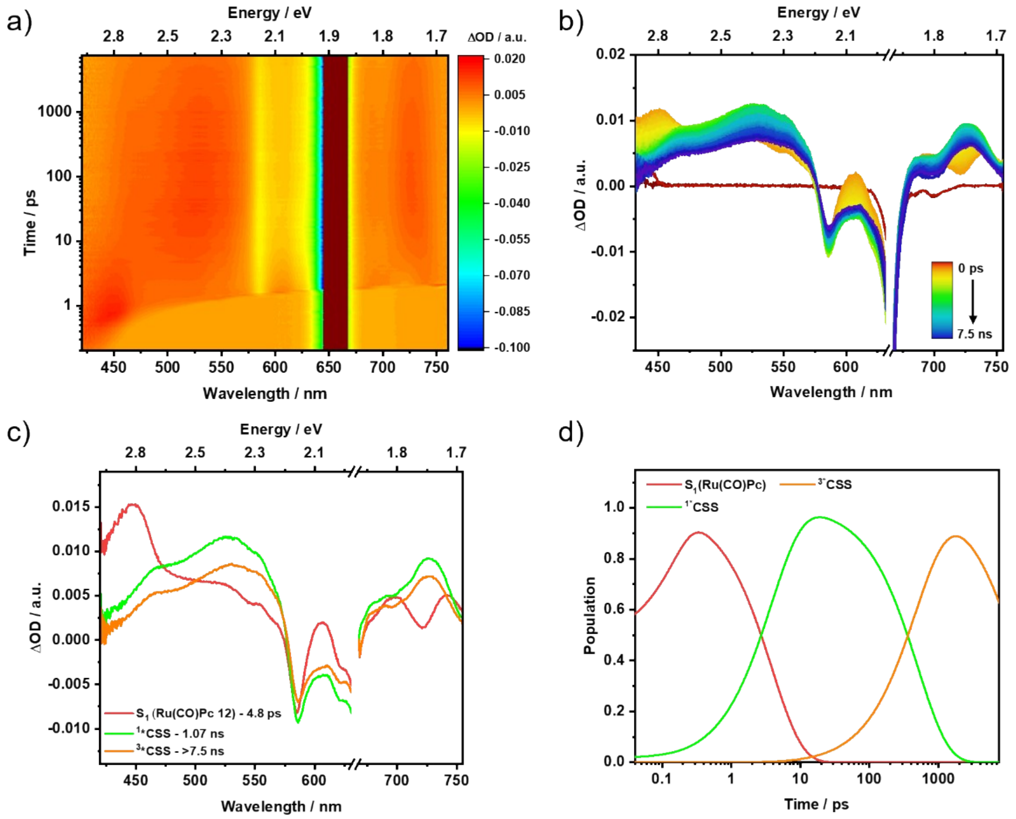

Figure S68. ns-TAS raw data from pump-probe experiments and the corresponding target analysis for conjugate Ru(CO)Pc-SubPz **3**, following 660 nm photoexcitation in argon-saturated toluene at room temperature. (a) Heat map of ns-TAS raw data. (b) Differential absorption spectra at time delays between 1 ns and 350 µs. (c) Evolution-associated spectra with their corresponding lifetimes, obtained from the deconvolution of the ns-TAS data. (d) Relative populations of the respective species.

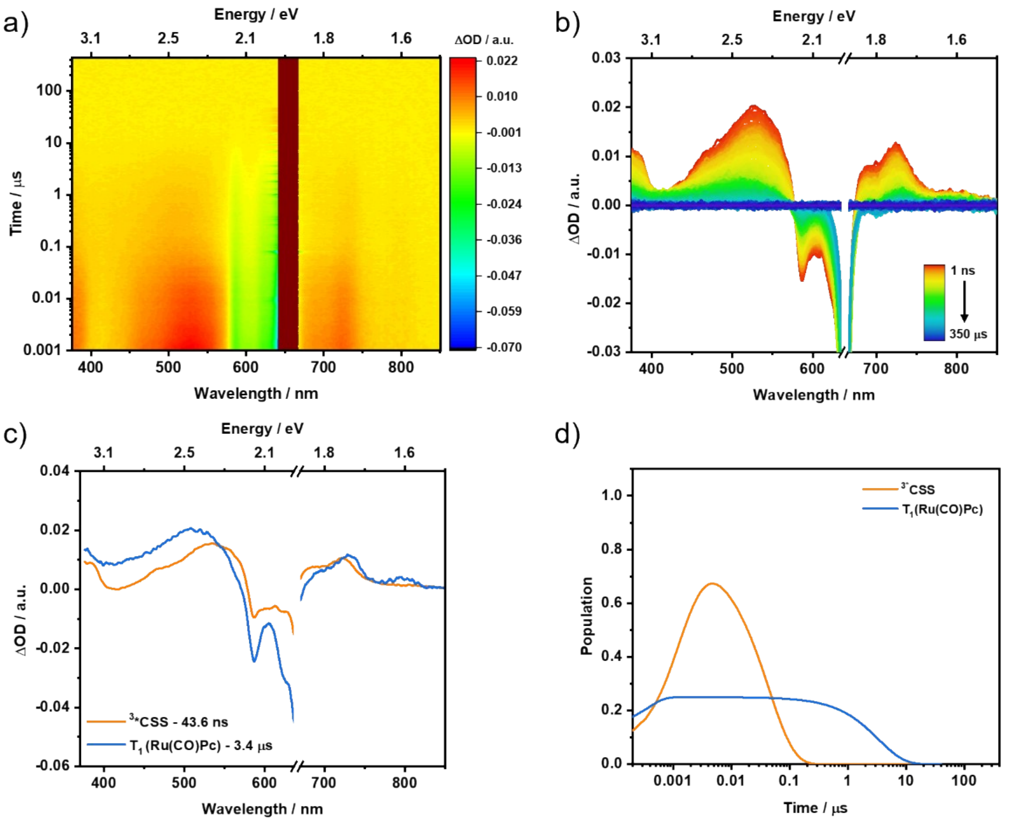

Figure S69. fs-TAS raw data from pump-probe experiments and the corresponding global sequential analysis for conjugate Ru(CO)Pc-SubPz **4**, following 660 nm photoexcitation in argon-saturated THF at room temperature. (a) Heat map of fs-TAS raw data. (b) Differential absorption spectra at time delays between 0 ps and 7.5 ns. (c) Evolution-associated spectra with their corresponding lifetimes, obtained from the deconvolution of the fs-TAS data. (d) Relative populations of the respective species.

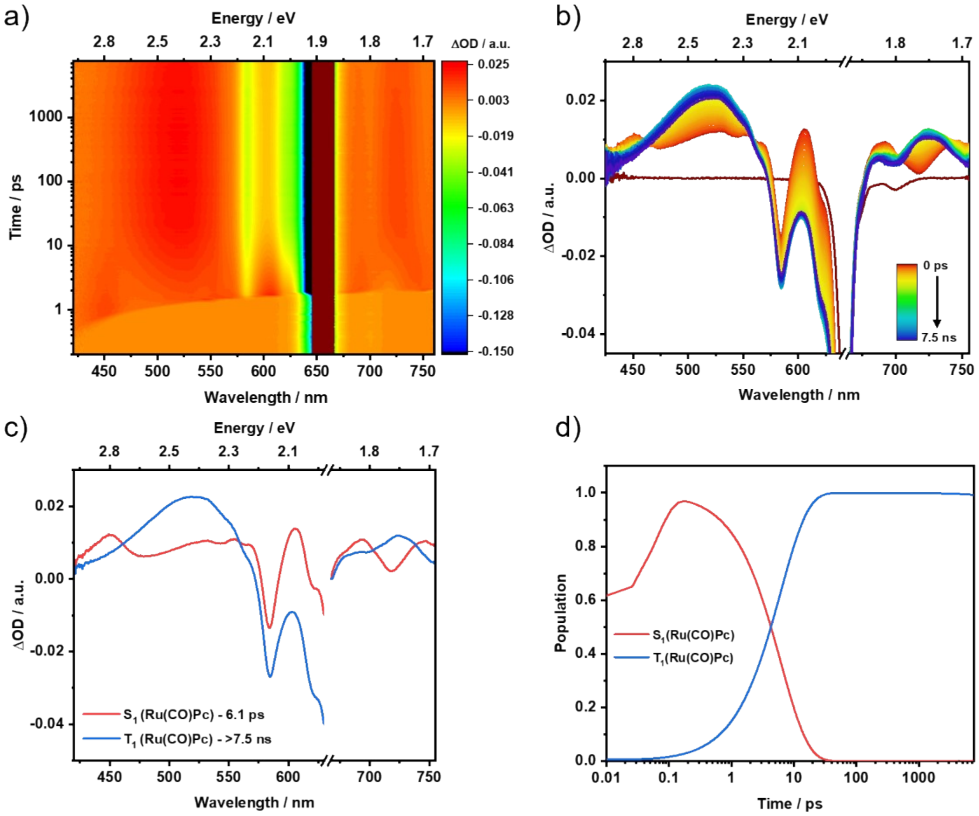

Figure S70. ns-TAS raw data from pump-probe experiments and the corresponding global sequential analysis for conjugate Ru(CO)Pc-SubPz **4**, following 660 nm photoexcitation in argon-saturated THF at room temperature. (a) Heat map of ns-TAS raw data. (b) Differential absorption spectra at time delays between 1 ns and 350 µs. (c) Evolution-associated spectrum with the corresponding lifetime, obtained from the deconvolution of the ns-TAS data. (d) Relative population of the deconvoluted species.

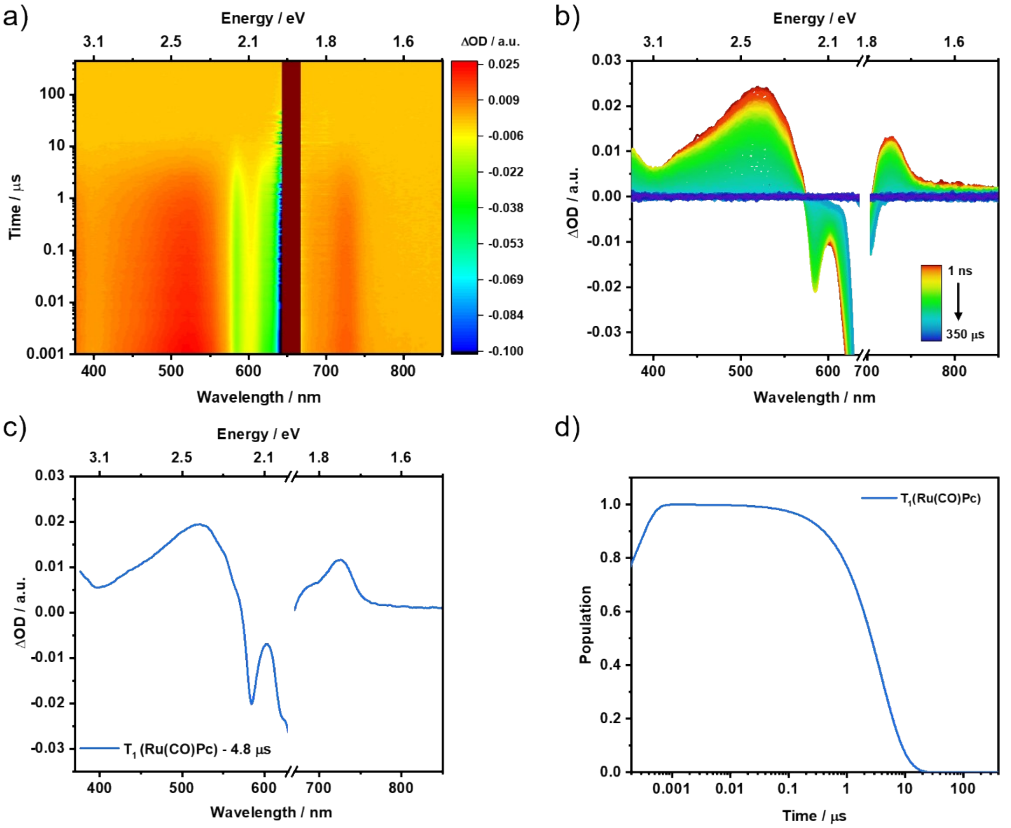

Figure S71. Species-associated spectra with the corresponding lifetimes, obtained from the deconvolution of the ns-TAS data of conjugate Ru(CO)Pc-SubPz **3**, following 480 nm photoexcitation in argon-saturated toluene at room temperature, with an applied magnetic field of (a) 0 mT, (b) 2 mT, and (c) 5 mT.

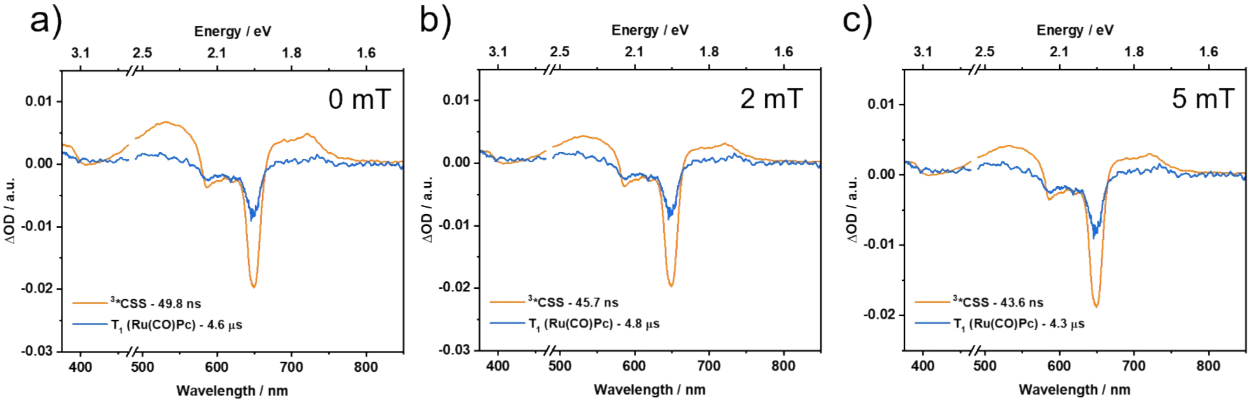

8. FRET parameters

According to Förster formalism, the efficiency of energy transfer is given by the following equation:[15]

$$E = \frac{R_0^6}{R_0^6 + r^6} \#(3)$$

where r is the donor-acceptor distance and R_0 is the Förster distance, i.e., the distance between donor and acceptor at which the FRET efficiency is 50 %.

Energy transfer efficiency can also be calculated using emission intensity and lifetime values using the following equations.

$$E = 1 - \frac{F_{DA}}{F_D} \#(4)$$

$$E = 1 - \frac{\tau_{DA}}{\tau_D} \#(5)$$

where F_{DA} and F_D are the fluorescence intensities of the donor in the presence and absence of the acceptor, respectively. τ_{DA} and τ_D refer to the lifetime of the donor in the presence and absence of the acceptor, respectively.

In this work, Ru(CO)Pc-SubPz conjugates **1** and **3** showed more than 80% energy transfer efficiency when calculated using equation (4). It was, however, not possible to determine the efficiency values for the conjugate **2** using the same method. Also, the fluorescence lifetime values of the donor in the presence of the acceptor (τ_{DA}) could not be obtained from the transient absorption measurements at 480 nm photoexcitation.

The Förster distance R_0 can be calculated using the equation:

$$R_0 = \left(\frac{9000[ln10]\varphi_D \kappa^2 J(\lambda)}{128\pi^5 N n^4} \right)^{1/6} \#(6)$$

where φ_D is the quantum yield of the donor, κ^2 describes the relative orientation of the transition dipoles of the donor and acceptor in space, n is the refractive index of the medium, N is the Avogadro constant and $J(\lambda)$ is the overlap integral. For randomly arranged transition dipoles, κ^2 value is usually taken as 2/3. The integral of spectral overlap between the donor emission and the acceptor absorption multiplied by wavelength λ to the fourth power gives the overlap integral, J,

$$J(\lambda) = \int_0^\infty F_D(\lambda)\varepsilon_A(\lambda)\lambda^4 d\lambda \#(7)$$

where $F_D(\lambda)$ is the normalized donor emission spectrum and ε_A is the molar extinction coefficient of the acceptor.

The rate of resonance energy transfer k_{FRET} can be written as

$$k_{FRET} = \frac{1}{\tau_D}\left(\frac{R_0}{r}\right)^6 \#(8)$$

An estimate of the FRET rate constants was determined using the above equation by substituting an 'r' value obtained from DFT calculations.

Table S1. Parameters used to calculate *i*-FRET rate constants for the conjugates Ru(CO)Pc-SubPz **1**, **2**, and **3**.

Compound	τ_D (ps)	Φ_D	J / 10^{15} (M^{-1} cm^{-1} nm^4)	R_0 (Å)	r (Å)	k_{FRET} / 10^{12} (s^{-1})
Ru(CO)Pc-SubPz **1**	226	0.019	1.149	25.06	7.48	6.22
Ru(CO)Pc-SubPz **2**	203	0.026	1.932	28.94	7.48	16.50

Ru(CO)Pc-SubPz **3**	311	0.015	7.521	33.20	7.19	31.11

9. DFT calculations

The molecular structures of the complexes were conformationally optimized using the global optimizer algorithm (GOAT, ORCA 6.0, GFN2-xTB).[16,17] These structures of the global minima were further optimized with density-functional theory (DFT) at the B3LYP-GD3BJ/6-31g*/LanL2DZ[18–22] level in Gaussian16.[23] The LANL2DZ basis set was used for the ruthenium atom and 6-31g* basis sets were used for other atoms in all complexes. The Avogadro program was used to visualize optimized structures.[24]

Figure S72. Optimized ground state geometries of the conjugates (a) Ru(CO)Pc-SubPz **1**, (b) Ru(CO)Pc-SubPz **2**, (c) Ru(CO)Pc-SubPz **3**, and (d) Ru(CO)Pc-SubPz **4**.

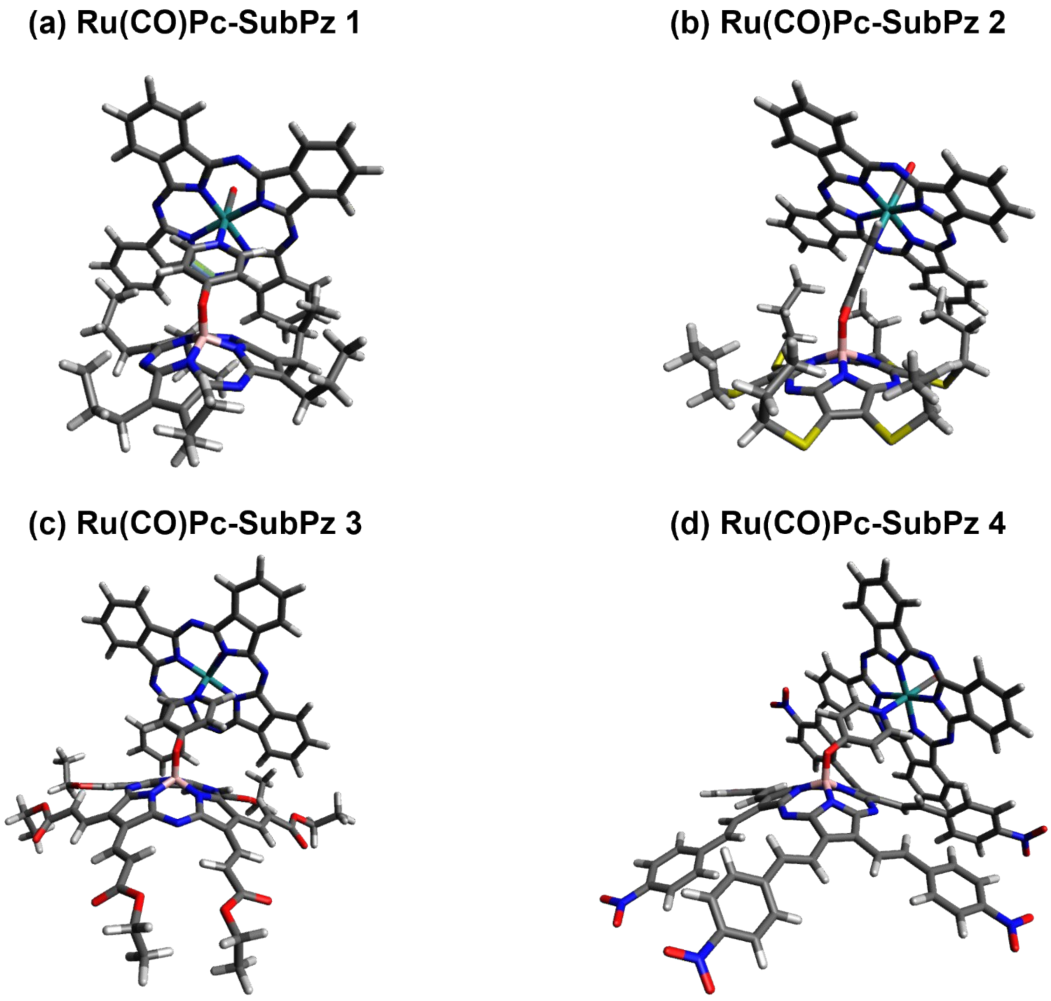

Figure S73. Orbital representations, energy levels of HOMO (bottom) and LUMO (top), and the corresponding energy gaps ΔE for (a) Ru(CO)Pc-SubPz **1**, (b) Ru(CO)Pc-SubPz **2**, (c) Ru(CO)Pc-SubPz **3**, and (d) Ru(CO)Pc-SubPz **4**.

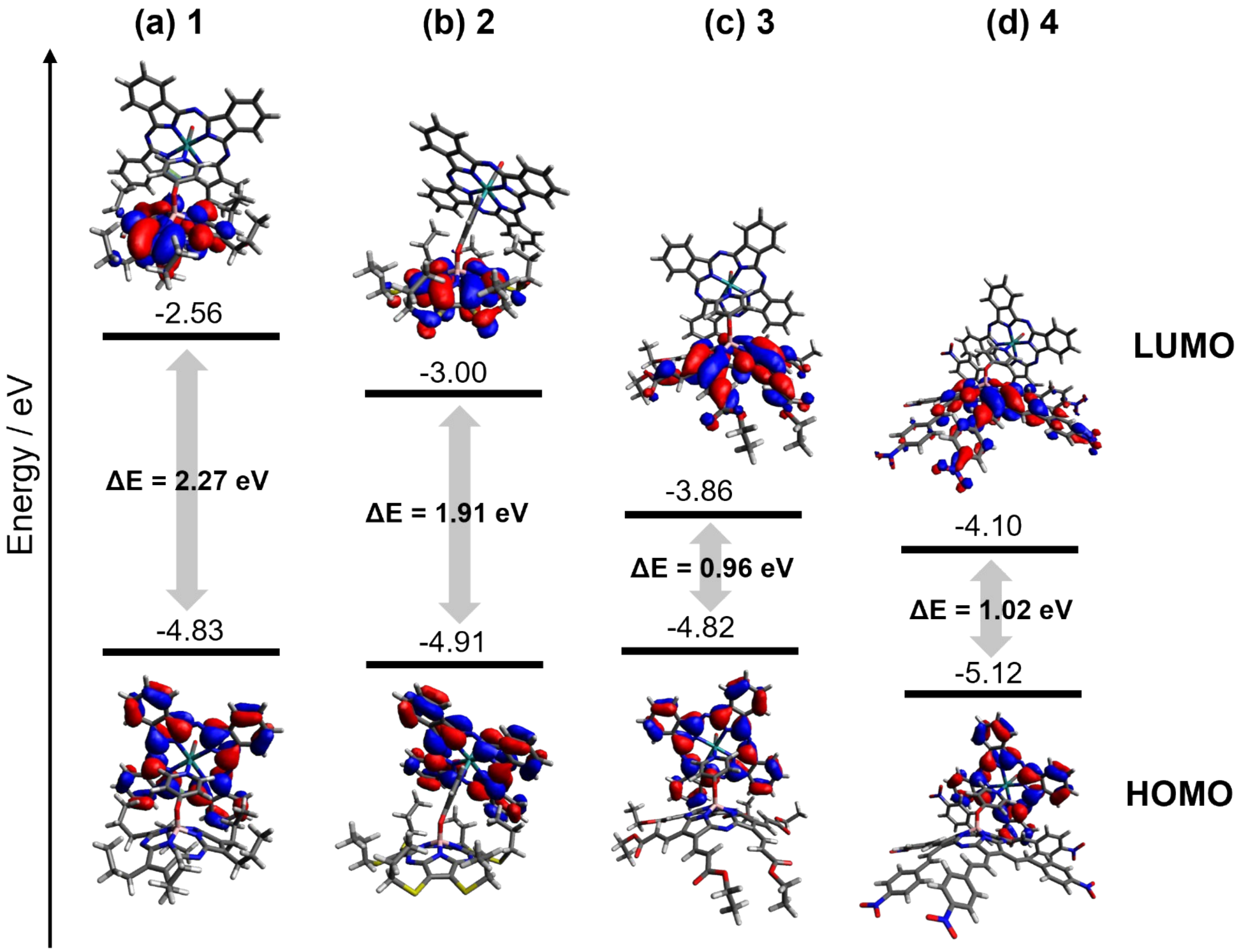

10. Optimized structures

RuPc-SubPz 1

149

C	4.48892	3.09978	-2.62411
C	4.05325	3.87474	-1.52397
C	3.66904	2.93351	-0.47108
N	3.88062	1.66508	-0.94754
C	4.36747	1.69528	-2.22737
C	4.60712	-2.92587	-2.84845
C	4.88261	-1.73895	-3.56638
C	4.55452	-0.62149	-2.67836
N	4.10868	-1.14695	-1.49518
C	4.11094	-2.51726	-1.53303
N	4.67238	0.66196	-3.00691
C	2.26094	-3.17198	2.70122
C	2.76162	-3.94778	1.62882
C	3.21150	-3.00671	0.60208
N	2.98558	-1.73881	1.07052
C	2.41261	-1.76892	2.31414
N	3.71889	-3.35789	-0.57827
C	2.19366	2.85023	2.94693
C	1.81596	1.66343	3.61847
C	2.19721	0.54857	2.75085
N	2.74337	1.07447	1.61013
C	2.75835	2.44287	1.65895
N	3.17578	3.28489	0.71478
N	2.04974	-0.73434	3.06736
C	1.76666	-3.77913	3.85393
C	1.77569	-5.17442	3.91473
C	2.27066	-5.94442	2.84738
C	2.76878	-5.33934	1.69220
C	4.81159	-4.17681	-3.42467
C	5.29998	-4.22128	-4.73217
C	5.57410	-3.04185	-5.44553
C	5.36746	-1.78610	-4.87065
C	2.01205	4.09692	3.54022
C	1.42502	4.13944	4.80651
C	1.02320	2.96224	5.46113
C	1.21893	1.70934	4.87612
C	4.91705	3.70556	-3.80246
C	4.90327	5.10061	-3.86206
C	4.46908	5.87039	-2.76917
C	4.03721	5.26572	-1.58700
Ru	3.50421	-0.03648	0.08942
C	-2.78333	1.67800	0.82513
N	-2.07446	0.80390	1.56100
C	-2.23945	-0.50210	1.29022
N	-2.96061	-0.92696	0.21076
N	-3.49025	1.30087	-0.28570
C	-3.45618	-2.18457	0.40009
N	-4.55798	-2.61301	-0.23984
C	-5.24150	-1.70989	-0.96809
N	-4.75683	-0.45609	-1.24695
C	-5.78535	0.43532	-1.41099
N	-5.65961	1.74760	-1.13969
C	-4.54266	2.14611	-0.50907

B	-3.36974	-0.04594	-0.91825
C	-6.66658	-1.67465	-1.25559
C	-7.00207	-0.35249	-1.52706
C	-3.24419	3.01750	1.17138
C	-4.33197	3.30205	0.35573
C	-2.78857	-2.70679	1.58767
C	-2.03837	-1.67263	2.13557
C	-5.23006	4.49857	0.35080
C	-2.65740	3.82999	2.28395
C	-5.07682	5.35226	-0.92618
C	-3.67690	5.94774	-1.08652
C	-1.11888	3.90745	2.22974
C	-0.57374	4.49410	0.92665
C	-8.35312	0.23272	-1.78887
C	-7.56746	-2.86394	-1.15371
C	-7.70516	-3.64221	-2.48181
C	-6.37569	-4.18424	-3.01131
C	-8.71106	0.29941	-3.29081
C	-7.73581	1.14484	-4.11280
C	-1.23610	-1.68930	3.39977
C	-2.91830	-4.12001	2.05555
C	-2.22810	-5.10268	1.08182
C	-0.73366	-4.82331	0.90705
C	-2.04784	-1.39415	4.67940
C	-2.60804	0.02859	4.74795
O	-2.46393	-0.12191	-2.07536
N	1.48578	-0.03622	-0.82451
C	0.89186	-1.19257	-1.17695
C	-0.41138	-1.25767	-1.64102
C	-1.15422	-0.07512	-1.75781
C	-0.50918	1.13431	-1.46583
C	0.79229	1.10481	-0.98888
C	5.21986	-0.04099	0.85803
O	6.27427	-0.04370	1.32747
H	1.39862	-3.17695	4.67836
H	1.39980	-5.67505	4.80248
H	2.26500	-7.02773	2.92583
H	3.15555	-5.92543	0.86479
H	4.59669	-5.08236	-2.86638
H	5.47192	-5.18256	-5.20795
H	5.95292	-3.11078	-6.46126
H	5.57507	-0.86999	-5.41414
H	2.31860	5.00185	3.02562
H	1.27196	5.09788	5.29442
H	0.56266	3.02969	6.44254
H	0.92806	0.79404	5.38171
H	5.24855	3.10308	-4.64201
H	5.23274	5.60179	-4.76772
H	4.47036	6.95380	-2.84798
H	3.69889	5.85169	-0.73841
H	-6.26983	4.15543	0.42182
H	-5.03348	5.11757	1.23331
H	-3.08534	4.83883	2.25972
H	-2.94741	3.38654	3.24680
H	-5.82293	6.15641	-0.89583
H	-5.32168	4.72887	-1.79501
H	-3.41984	6.58680	-0.23248
H	-2.91658	5.16189	-1.14838
H	-0.71092	2.90365	2.38275

H	-0.77678	4.50943	3.07732
H	-0.88228	3.89196	0.06370
H	-0.94098	5.51426	0.75991
H	-9.11864	-0.35200	-1.26478
H	-8.37586	1.24770	-1.37425
H	-8.56302	-2.54808	-0.81957
H	-7.16412	-3.53822	-0.38878
H	-8.16490	-2.98822	-3.23412
H	-8.40522	-4.47137	-2.31739
H	-6.53046	-4.79480	-3.90757
H	-5.69071	-3.37110	-3.27428
H	-9.72493	0.71033	-3.37947
H	-8.74968	-0.72036	-3.69528
H	-6.73363	0.70314	-4.11925
H	-8.07082	1.23019	-5.15227
H	-0.76411	-2.67240	3.49865
H	-0.41902	-0.96622	3.32052
H	-2.48219	-4.22249	3.05538
H	-3.98077	-4.38222	2.13392
H	-2.73949	-5.04743	0.11255
H	-2.37558	-6.12313	1.45789
H	-0.20391	-4.88232	1.86280
H	-0.27116	-5.54744	0.22810
H	-1.38614	-1.56665	5.53880
H	-2.86605	-2.12183	4.76439
H	-1.80688	0.76995	4.65056
H	-3.32116	0.21755	3.93965
H	1.47866	-2.09286	-1.05392
H	-0.87259	-2.21125	-1.87074
H	-1.03883	2.07373	-1.56562
H	1.29548	2.02008	-0.70623
H	-5.87484	-4.80334	-2.25800
H	-7.64519	2.15578	-3.69873
H	-3.60666	6.55724	-1.99390
H	0.52078	4.52178	0.93624
H	-3.12210	0.19858	5.70104
H	-0.56046	-3.82136	0.49799

RuPc-SubPz 2

155

C	-4.11244	2.5062	2.91609
C	-4.19827	3.58255	2.01008
C	-4.141	3.00386	0.67881
N	-4.01021	1.65856	0.79809
C	-3.99345	1.30398	2.10901
C	-3.21811	-3.19205	1.41253
C	-3.49672	-2.32344	2.48657
C	-3.68462	-1.00486	1.90587
N	-3.57323	-1.10664	0.55639
C	-3.29424	-2.38684	0.20647
N	-3.86591	0.09219	2.61533
C	-3.33418	-1.68985	-4.35445
C	-3.09442	-2.7403	-3.44615
C	-3.25301	-2.17516	-2.11748
N	-3.55675	-0.85875	-2.2398
C	-3.61266	-0.51177	-3.55123
N	-3.12107	-2.8715	-1.00696
C	-4.29903	3.99943	-2.85614
C	-4.19812	3.09793	-3.93443
C	-4.00834	1.78146	-3.34943
N	-4.00937	1.90437	-1.99757
C	-4.17632	3.20482	-1.64551
N	-4.21756	3.70999	-0.43048
N	-3.83165	0.68479	-4.05851
C	-3.27083	-1.9025	-5.72091
C	-2.96512	-3.17929	-6.16181
C	-2.72599	-4.2168	-5.26438
C	-2.78695	-4.01298	-3.89592
C	-2.96066	-4.53508	1.6303
C	-2.99324	-4.99651	2.93529
C	-3.29267	-4.14438	3.99501
C	-3.55099	-2.79956	3.78676
C	-4.48902	5.35265	-3.07668
C	-4.57603	5.78697	-4.38907
C	-4.47561	4.89612	-5.45428
C	-4.28456	3.54064	-5.24304
C	-4.14701	2.72455	4.28312
C	-4.26206	4.03139	4.72603
C	-4.34297	5.09479	3.83095
C	-4.31472	4.88575	2.46236
Ru	-3.85539	0.39046	-0.72304
N	-1.75656	0.694	-0.69711
C	-1.07812	0.68283	0.45303
C	0.29513	0.65168	0.53421
C	1.06055	0.59883	-0.63705
C	0.34489	0.68276	-1.84164
C	-1.02833	0.73142	-1.82036
C	2.27054	-1.08239	2.46588
N	2.34855	0.04154	3.17438
C	3.09412	1.0333	2.69488
N	3.63972	1.01074	1.4431
N	2.72685	-1.16268	1.18189
C	4.81504	1.70479	1.40449
N	5.78283	1.41179	0.54203
C	5.6786	0.28472	-0.15904
N	4.53879	-0.46274	-0.17929

C	4.79994	-1.77625	-0.43211
N	3.99136	-2.75202	-0.02635
C	3.03828	-2.44856	0.84691
B	3.24359	0.00835	0.40083
C	6.69917	-0.54525	-0.72856
C	6.16005	-1.82579	-0.8864
C	1.9792	-2.4295	2.86704
C	2.42362	-3.27259	1.84513
C	4.83723	2.49899	2.60478
C	3.75583	2.09612	3.3905
S	2.34041	-5.00278	1.86741
S	1.36676	-3.02815	4.37162
C	2.08682	-5.35404	0.08504
C	0.63218	-5.21051	-0.34473
C	0.13467	-3.77035	-0.34465
C	0.58193	-1.55727	5.112
C	-0.62737	-1.09967	4.31303
C	-1.50584	-0.14728	5.11436
S	7.1019	-3.18666	-1.39588
S	8.36807	-0.16427	-1.01263
C	8.27799	1.6207	-1.41262
C	8.02238	1.8746	-2.89161
C	6.62642	1.4512	-3.32947
C	5.83771	-4.48126	-1.64199
C	4.94325	-4.21308	-2.84204
C	5.71971	-4.22317	-4.15202
S	3.34612	2.72545	4.95432
S	6.0157	3.64424	3.1495
C	6.88747	4.07832	1.60638
C	6.00217	4.85615	0.64618
C	6.79204	5.31864	-0.57231
C	1.52203	2.5777	4.86316
C	0.93365	3.49533	3.80204
C	-0.57823	3.32645	3.69914
O	2.37537	0.48758	-0.68683
C	-5.67709	0.09032	-0.75358
O	-6.80735	-0.09986	-0.77896
H	-3.45974	-1.09413	-6.40942
H	-2.91223	-3.37824	-7.22228
H	-2.49137	-5.19964	-5.64671
H	-2.60673	-4.81278	-3.19474
H	-2.74809	-5.1917	0.80157
H	-2.79161	-6.0379	3.13859
H	-3.3236	-4.54313	4.9984
H	-3.79379	-2.13738	4.60263
H	-4.56803	6.0361	-2.24611
H	-4.72678	6.83695	-4.59368
H	-4.55074	5.27242	-6.46411
H	-4.20878	2.84349	-6.06251
H	-4.09182	1.89842	4.9744
H	-4.29387	4.23267	5.78698
H	-4.43389	6.10037	4.21486
H	-4.38283	5.70272	1.7616
H	-1.65515	0.70177	1.36963
H	0.76096	0.66604	1.50381
H	0.88052	0.6919	-2.77697
H	-1.56635	0.79623	-2.75793
H	2.42281	-6.38328	-0.03837
H	2.74235	-4.68762	-0.47003

H	0.54674	-5.61889	-1.35535
H	0.00297	-5.81086	0.31505
H	-0.86982	-3.7177	-0.7593
H	0.78923	-3.13674	-0.94262
H	0.09817	-3.37347	0.66812
H	0.28492	-1.89522	6.10489
H	1.3421	-0.78392	5.20085
H	-1.21642	-1.97326	4.02723
H	-0.29333	-0.61219	3.39478
H	-0.95056	0.73853	5.41408
H	-1.87309	-0.64016	6.01177
H	-2.35854	0.15476	4.50996
H	7.49355	2.03996	-0.78851
H	9.24525	2.02309	-1.11295
H	8.76953	1.34127	-3.48266
H	8.15255	2.9447	-3.07608
H	6.50358	0.37486	-3.2323
H	6.46063	1.71944	-4.36993
H	5.86783	1.9408	-2.72126
H	6.41736	-5.3932	-1.78352
H	5.26582	-4.54484	-0.7189
H	4.44524	-3.24989	-2.7128
H	4.1697	-4.98505	-2.86494
H	6.17963	-5.19538	-4.31447
H	6.50624	-3.47199	-4.13253
H	5.05832	-4.0101	-4.98795
H	7.7313	4.68386	1.9368
H	7.25013	3.15222	1.16697
H	5.58336	5.72195	1.16378
H	5.17272	4.22196	0.32574
H	7.20297	4.4663	-1.10781
H	7.61361	5.96489	-0.27211
H	6.1476	5.87363	-1.24937
H	1.29275	1.53389	4.66174
H	1.16912	2.84703	5.85782
H	1.38955	3.2668	2.8346
H	1.17641	4.53145	4.04633
H	-0.83441	2.30539	3.42678
H	-0.98462	3.99262	2.94206
H	-1.05938	3.55735	4.64647

RuPc-SubPz 3

173

C	1.57014	1.50608	-0.42993
N	1.95981	2.06034	-1.57468
C	2.59732	1.30373	-2.46147
N	2.67776	-0.05053	-2.3446
N	1.62876	0.16141	-0.22458
C	3.81013	-0.53242	-2.93102
N	4.42107	-1.63311	-2.50905
C	4.01851	-2.18789	-1.37114
N	2.89707	-1.77098	-0.72724
C	2.95559	-2.02705	0.60687
N	2.32649	-1.2764	1.50354
C	1.76866	-0.14088	1.0944
B	1.9	-0.82157	-1.32262
C	4.73406	-3.02876	-0.43445
C	4.05777	-2.94279	0.79705
C	1.36457	2.1076	0.86503
C	1.51233	1.08063	1.81916
C	4.33085	0.53961	-3.75055
C	3.57805	1.68964	-3.45134
C	1.49653	1.2236	3.23554
C	1.12382	3.49163	1.10993
C	1.73035	0.22378	4.10225
C	1.63143	0.44877	5.5506
O	1.96078	-0.66293	6.22495
C	0.89616	4.39764	0.14389
C	0.64841	5.80797	0.47016
O	0.35366	6.50299	-0.63744
C	4.40355	-3.5598	2.03643
C	5.93101	-3.75823	-0.69937
C	6.62512	-3.70526	-1.84873
C	7.84481	-4.51077	-2.01535
O	8.43009	-4.25628	-3.19386
C	3.6295	-3.51161	3.13489
C	4.0436	-4.16372	4.38547
O	3.11272	-3.99229	5.33674
C	3.78053	3.01392	-3.94306
C	5.44393	0.44082	-4.63726
C	6.06304	-0.71207	-4.94168
C	7.20457	-0.73534	-5.86914
O	7.64686	-1.98841	-6.03904
C	3.01661	4.06446	-3.59855
C	3.28897	5.41397	-4.11461
O	2.37528	6.27968	-3.65193
O	0.72522	-1.55716	-1.81192
O	0.70784	6.29216	1.5759
O	1.29053	1.48356	6.07442
O	5.07698	-4.7659	4.55301
O	8.26522	-5.3085	-1.21089
O	7.67853	0.23201	-6.41616
O	4.19588	5.71309	-4.85402
C	1.84762	-0.62616	7.64491
C	0.45198	-1.04196	8.0905
C	0.06728	7.88963	-0.47622
C	-1.38108	8.11382	-0.06337
C	3.3765	-4.56631	6.61479
C	2.98494	-6.03726	6.65886

C	9.62331	-4.97891	-3.48991
C	10.83705	-4.37584	-2.79533
C	8.77801	-2.1673	-6.8876
C	10.07528	-1.85408	-6.15504
C	2.52502	7.64839	-4.01804
C	3.43425	8.37997	-3.04002
C	-2.54818	-3.41923	3.35902
C	-1.74677	-2.3304	3.7576
C	-2.34012	-1.14513	3.16483
N	-3.4138	-1.52417	2.42432
C	-3.59775	-2.86773	2.52047
C	-7.12473	-3.11617	-0.45021
C	-6.43908	-3.95528	0.45132
C	-5.43211	-3.12965	1.09334
N	-5.53248	-1.86572	0.60299
C	-6.51976	-1.80181	-0.32783
N	-4.54037	-3.58671	1.94859
C	-6.01351	2.68829	-1.26819
C	-6.78096	1.59379	-1.71466
C	-6.36311	0.45043	-0.92279
N	-5.38969	0.85271	-0.06349
C	-5.15058	2.18096	-0.2162
N	-6.88281	-0.75262	-1.03398
C	-1.96968	2.5152	3.11698
C	-2.65083	3.35879	2.21618
C	-3.50969	2.4992	1.42073
N	-3.33089	1.21326	1.82396
C	-2.39856	1.16122	2.81109
N	-1.90599	0.07966	3.37505
N	-4.31124	2.92978	0.47007
C	-6.17696	3.94623	-1.82291
C	-7.11895	4.08994	-2.82795
C	-7.87795	3.0089	-3.26854
C	-7.72087	1.74701	-2.71949
C	-8.15836	-3.60516	-1.23068
C	-8.49387	-4.94186	-1.09284
C	-7.81609	-5.77063	-0.20248
C	-6.77951	-5.29104	0.58077
C	-1.13697	3.04172	4.0904
C	-0.96637	4.41741	4.11495
C	-1.61376	5.2478	3.20516
C	-2.47011	4.73105	2.24649
C	-2.23883	-4.71046	3.7519
C	-1.10993	-4.89424	4.53392
C	-0.31412	-3.81935	4.9212
C	-0.62576	-2.52312	4.5457
Ru	-4.46411	-0.32077	1.24033
N	-3.00983	-0.75509	-0.24765
C	-2.56614	-2.00595	-0.42402
C	-1.35018	-2.31161	-0.98828
C	-0.50306	-1.27548	-1.40201
C	-1.03556	0.02015	-1.35155
C	-2.2658	0.2252	-0.7698
C	-5.68803	0.05246	2.57269
O	-6.44418	0.28348	3.39964
H	1.27999	2.20742	3.62701
H	1.1006	3.82463	2.13889
H	1.98972	-0.76864	3.77288
H	0.88779	4.13564	-0.90105

H	5.33426	-4.1096	2.0866
H	6.30318	-4.40048	0.08886
H	6.33194	-3.07527	-2.6719
H	2.6831	-2.99628	3.14842
H	4.59774	3.17474	-4.6338
H	5.7963	1.3507	-5.10545
H	5.75674	-1.65635	-4.52292
H	2.18967	3.96873	-2.91504
H	2.07177	0.38719	7.99439
H	2.6047	-1.3217	8.01451
H	0.21019	-2.02981	7.70508
H	-0.28301	-0.3328	7.71713
H	0.40426	-1.05958	9.17555
H	0.74255	8.30764	0.27843
H	0.27226	8.33778	-1.45065
H	-1.55857	7.67173	0.91367
H	-1.58614	9.17931	-0.01171
H	-2.05211	7.65553	-0.78597
H	2.78281	-3.97888	7.31907
H	4.44207	-4.45507	6.84139
H	3.1462	-6.42775	7.65954
H	3.59428	-6.60254	5.95807
H	1.93709	-6.15928	6.39406
H	9.49431	-6.02048	-3.1763
H	9.72151	-4.92794	-4.57642
H	10.93435	-3.32171	-3.04546
H	10.73183	-4.47263	-1.71774
H	11.73471	-4.9002	-3.11017
H	8.73938	-3.21529	-7.19221
H	8.67296	-1.5192	-7.76443
H	10.11347	-0.79649	-5.90606
H	10.13102	-2.43537	-5.23843
H	10.92322	-2.09846	-6.78846
H	2.93538	7.70427	-5.03198
H	1.51423	8.06245	-4.00224
H	3.46755	9.43666	-3.28946
H	3.06306	8.26233	-2.0246
H	4.4409	7.97285	-3.09571
H	-5.58754	4.77899	-1.47343
H	-7.27178	5.059	-3.27977
H	-8.60423	3.16112	-4.05328
H	-8.30661	0.9055	-3.05347
H	-8.6788	-2.95687	-1.91745
H	-9.29785	-5.35256	-1.68563
H	-8.10786	-6.80743	-0.12244
H	-6.25061	-5.92583	1.27385
H	-0.65659	2.40363	4.81523
H	-0.32103	4.85628	4.86076
H	-1.44609	6.31216	3.25932
H	-2.99758	5.36527	1.55128
H	-2.85981	-5.53839	3.44829
H	-0.83959	-5.89222	4.84729
H	0.57096	-3.99616	5.51273
H	-0.01812	-1.68656	4.8466
H	-3.20577	-2.81128	-0.08596
H	-1.02375	-3.33528	-1.07291
H	-0.48704	0.85971	-1.74724
H	-2.66436	1.23147	-0.71958

RuPc-SubPz 4

191

C	-0.06691	0.37694	2.05347
N	0.01853	1.56457	2.63954
C	0.88468	2.4486	2.15484
N	1.54363	2.25314	0.98117
N	0.57243	0.09552	0.88572
C	2.73231	2.90849	0.95666
N	3.74773	2.4982	0.20406
C	3.66382	1.30733	-0.38102
N	2.51249	0.57902	-0.39622
C	2.78017	-0.75639	-0.40181
N	1.93514	-1.65446	0.10249
C	0.89782	-1.22196	0.80871
B	1.18943	1.1564	0.0228
C	4.73142	0.40012	-0.72725
C	4.17886	-0.90083	-0.72232
C	-0.46014	-0.89294	2.61948
C	0.14968	-1.89341	1.84957
C	2.71874	3.82419	2.07601
C	1.55478	3.53994	2.82241
C	0.07732	-3.30504	2.03144
C	-1.29058	-1.04072	3.78359
C	0.48533	-4.17859	1.08897
C	0.43892	-5.61767	1.18252
C	-2.29655	-0.20618	4.09482
C	-2.78551	0.85177	3.22379
C	4.86858	-2.13172	-0.91165
C	6.08907	0.76778	-0.93723
C	6.4929	2.05225	-1.05216
C	7.8416	2.51251	-1.26817
C	4.28764	-3.33948	-0.73955
C	4.91304	-4.62832	-0.89535
C	1.14668	4.13539	4.04955
C	3.74031	4.75961	2.40477
C	4.93102	4.81591	1.7703
C	6.00916	5.72752	2.06485
C	0.09478	3.69226	4.77103
C	-0.36163	4.23711	6.02606
C	-3.22954	2.06626	3.75447
C	-3.63975	3.09083	2.92801
C	-3.62518	2.90013	1.55088
C	-3.23416	1.68145	1.00636
C	-2.83291	0.6641	1.83753
C	0.52931	-6.37275	0.00335
C	0.46742	-7.74784	0.03446
C	0.32667	-8.39395	1.25846
C	0.25963	-7.66928	2.44453
C	0.31518	-6.29418	2.40362
C	6.19409	-4.80419	-1.43892
C	6.74186	-6.06059	-1.56245
C	6.01549	-7.17121	-1.14262
C	4.74012	-7.02658	-0.60468
C	4.19945	-5.767	-0.48705
C	8.04558	3.86896	-1.57081
C	9.30885	4.3672	-1.79501
C	10.40154	3.50951	-1.71503
C	10.2297	2.16376	-1.40434

C	8.96289	1.67371	-1.18306
C	-0.00241	5.51249	6.48456
C	-0.4679	5.98621	7.69042
C	-1.30443	5.18789	8.46485
C	-1.67875	3.91891	8.03259
C	-1.21287	3.45671	6.8236
C	7.28436	5.44672	1.54998
C	8.35196	6.27808	1.80089
C	8.15767	7.41937	2.57293
C	6.90128	7.72771	3.08598
C	5.8402	6.88829	2.83352
O	0.32818	1.70063	-1.0332
C	-0.64791	1.04353	-1.63454
C	-0.67823	-0.34487	-1.82114
C	-1.80905	-0.93853	-2.33267
C	-2.83174	1.07229	-2.63733
C	-1.74831	1.75615	-2.13596
N	-4.03811	3.95481	0.67215
O	-3.93803	3.76065	-0.52456
O	-4.45648	4.97889	1.16858
N	0.24642	-9.83089	1.29888
O	0.13679	-10.35592	2.38691
O	0.28611	-10.42454	0.2424
N	11.72452	4.01741	-1.96424
O	11.82881	5.18731	-2.26629
O	12.6519	3.24144	-1.86309
N	9.26852	8.29197	2.8453
O	10.35347	7.97413	2.40645
O	9.05143	9.28989	3.50082
N	-1.78982	5.67913	9.72541
O	-2.53302	4.95946	10.3606
O	-1.42668	6.78169	10.07842
N	6.5903	-8.48332	-1.26678
O	5.93213	-9.4245	-0.87595
O	7.6991	-8.56797	-1.75297
H	-0.37309	-3.64839	2.95131
H	-1.06028	-1.8674	4.4421
H	0.8506	-3.78026	0.15267
H	-2.80185	-0.31763	5.04479
H	5.90951	-2.0554	-1.19114
H	6.7978	-0.04183	-1.03374
H	5.73595	2.82216	-0.99973
H	3.25622	-3.35482	-0.41845
H	1.74261	4.96535	4.40177
H	3.52902	5.42445	3.23026
H	5.12194	4.0861	0.99683
H	-0.43515	2.82326	4.40639
H	-3.23479	2.21189	4.82402
H	-3.96882	4.0386	3.32565
H	-3.26058	1.55502	-0.06396
H	-2.58104	-0.29851	1.42169
H	0.62712	-5.86237	-0.94357
H	0.52524	-8.33768	-0.8676
H	0.16428	-8.20262	3.37822
H	0.27971	-5.73352	3.32403
H	6.75885	-3.95001	-1.77635
H	7.72631	-6.20723	-1.98006
H	4.19874	-7.90601	-0.29079
H	3.21299	-5.64631	-0.06454

H	7.19181	4.52679	-1.64157
H	9.47235	5.40713	-2.033
H	11.09849	1.52616	-1.34358
H	8.83904	0.63254	-0.93222
H	0.63037	6.14221	5.8799
H	-0.20111	6.96803	8.05084
H	-2.32811	3.32335	8.65614
H	-1.48274	2.46821	6.48329
H	7.42836	4.55452	0.95923
H	9.33643	6.06718	1.41189
H	6.78242	8.62501	3.67405
H	4.86554	7.14111	3.21919
H	0.1666	-0.96082	-1.56268
H	-1.82197	-2.01428	-2.45631
H	-3.68653	1.63606	-2.98985
H	-1.76718	2.8321	-2.07898
N	-2.90755	-0.26476	-2.6888
C	-3.12354	-3.86164	-5.75314
C	-2.82176	-4.59622	-4.58908
C	-3.31255	-3.81025	-3.47125
N	-3.87397	-2.66934	-3.95629
C	-3.79042	-2.64925	-5.31104
C	-5.95739	1.38653	-5.88344
C	-5.34078	0.39541	-6.6724
C	-4.85048	-0.6153	-5.75077
N	-5.15719	-0.23152	-4.48369
C	-5.82157	0.95392	-4.5032
N	-4.22752	-1.71103	-6.12361
C	-6.28296	1.3613	0.07265
C	-6.60941	2.08338	-1.09183
C	-6.1451	1.28326	-2.21131
N	-5.56078	0.156	-1.72802
C	-5.6178	0.14883	-0.37028
N	-6.26948	1.63706	-3.47235
C	-3.42101	-3.87062	0.20495
C	-3.97898	-2.844	0.99398
C	-4.50687	-1.85793	0.06937
N	-4.25179	-2.27206	-1.19804
C	-3.60676	-3.46852	-1.17694
N	-3.18622	-4.16818	-2.21082
N	-5.13004	-0.76017	0.44501
C	-6.582	1.86236	1.32852
C	-7.20372	3.0987	1.39757
C	-7.51814	3.81604	0.24636
C	-7.22798	3.31926	-1.01395
C	-6.53939	2.50015	-6.46349
C	-6.49579	2.60289	-7.84464
C	-5.88793	1.6232	-8.6246
C	-5.30139	0.50622	-8.05137
C	-2.86747	-4.99632	0.7916
C	-2.87545	-5.07312	2.17363
C	-3.40838	-4.04918	2.95327
C	-3.96838	-2.92224	2.37663
C	-2.79057	-4.34648	-7.0063
C	-2.15239	-5.57429	-7.07358
C	-1.85319	-6.29941	-5.92324
C	-2.18248	-5.82194	-4.66534
Ru	-4.76488	-1.28237	-2.84881
C	-6.38526	-2.16953	-2.94061

O	-7.38612	-2.71855	-2.9964
H	-6.32973	1.30197	2.21548
H	-7.44328	3.52091	2.36205
H	-7.99083	4.78144	0.34277
H	-7.46332	3.87085	-1.90944
H	-7.00949	3.25354	-5.85195
H	-6.94297	3.45845	-8.32852
H	-5.87569	1.73794	-9.69832
H	-4.82986	-0.25739	-8.6491
H	-2.46073	-5.78832	0.18324
H	-2.46724	-5.94794	2.65789
H	-3.39836	-4.14653	4.02915
H	-4.39846	-2.13211	2.97154
H	-3.02846	-3.78076	-7.89281
H	-1.88284	-5.98143	-8.0367
H	-1.35794	-7.25442	-6.01692
H	-1.96059	-6.38082	-3.76992

11. Crystallographic data

Figure S74. Unit cell and crystalline packing of SubPz **7a**.

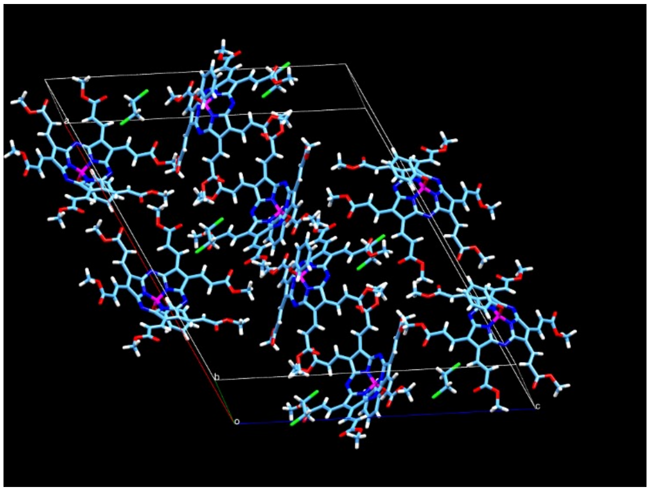

Table S2. Crystallographic data for **7a**

**Chemical formula**	$C_{42.75}H_{36.50}BCl_{0.75}N_6O_{13}$
**Formula weight**	879.67 g/mol
**Temperature**	200(2) K
**Wavelength**	0.71073 Å
**Crystal size**	0.015 x 0.124 x 0.164 mm
**Crystal habit**	Dark purple plate
**Crystal system**	Monoclinic
**Space group**	C 1 2/c 1
**Unit cell dimensions**	a = 41.747(3) Å α = 90°
	b = 7.8119(7) Å β = 120.236(3)°
	c = 30.271(3) Å γ = 90°
**Volume**	8529.1(13) Å^3
**Z**	8
**Density (calculated)**	1.370 g/cm^3
**Absorption coefficient**	0.147 mm^{-1}
**F(000)**	3654

	**x/a**	**y/b**	**z/c**	**U(eq)**
B1	0.36658(16)	0.6846(8)	0.4697(2)	0.0310(15)
C1	0.33183(14)	0.6575(7)	0.51715(19)	0.0314(13)
C2	0.29602(13)	0.5931(7)	0.5063(2)	0.0303(13)
C4	0.30579(14)	0.5297(7)	0.4401(2)	0.0337(13)
C5	0.34272(14)	0.4347(7)	0.40895(19)	0.0321(13)
C6	0.35944(15)	0.3050(7)	0.39275(19)	0.0353(14)
C7	0.39787(14)	0.3243(7)	0.42187(19)	0.0314(13)
C8	0.40469(14)	0.4665(7)	0.45605(19)	0.0305(13)
C9	0.43123(13)	0.5950(6)	0.53526(19)	0.0280(12)
C10	0.45439(13)	0.6049(6)	0.59082(19)	0.0293(13)
C11	0.43197(13)	0.6674(7)	0.60923(19)	0.0301(13)

	x/a	y/b	z/c	U(eq)
C12	0.39462(14)	0.6912(6)	0.56487(19)	0.0309(13)
C13	0.28170(14)	0.6024(7)	0.5411(2)	0.0340(13)
C14	0.29759(15)	0.6834(7)	0.5851(2)	0.0393(14)
C15	0.28154(17)	0.6811(8)	0.6182(2)	0.0462(16)
C16	0.2905(2)	0.7700(13)	0.6981(3)	0.101(3)
C17	0.28004(13)	0.5130(7)	0.45891(19)	0.0325(13)
C18	0.24503(14)	0.4230(7)	0.4346(2)	0.0392(14)
C19	0.22928(15)	0.3408(9)	0.3906(2)	0.0527(17)
C20	0.19199(17)	0.2648(9)	0.3711(3)	0.0599(19)
C21	0.1412(2)	0.1286(14)	0.3032(3)	0.125(4)
C22	0.34091(16)	0.1594(8)	0.3579(2)	0.0434(15)
C23	0.30635(17)	0.1310(9)	0.3291(2)	0.0534(17)
C24	0.2914(2)	0.9765(9)	0.2965(3)	0.0530(17)
C25	0.3033(2)	0.7099(12)	0.2729(4)	0.120(4)
C26	0.42593(15)	0.2206(7)	0.4196(2)	0.0356(14)
C27	0.46182(16)	0.2262(7)	0.4519(2)	0.0405(15)
C28	0.48933(18)	0.1265(7)	0.4463(2)	0.0407(15)
C29	0.50119(17)	0.9560(9)	0.3924(3)	0.0613(19)
C30	0.49179(13)	0.5494(7)	0.6205(2)	0.0345(13)
C31	0.51145(14)	0.4812(7)	0.6012(2)	0.0369(14)
C32	0.54909(16)	0.4154(8)	0.6336(3)	0.0463(16)
C33	0.59750(17)	0.2644(9)	0.6321(3)	0.072(2)
C34	0.44216(14)	0.6957(7)	0.6614(2)	0.0346(14)
C35	0.41907(16)	0.7494(8)	0.6766(2)	0.0486(16)
C36	0.43100(18)	0.7804(9)	0.7304(2)	0.0566(18)
C37	0.4093(2)	0.8594(14)	0.7856(3)	0.111(3)
C38	0.36482(14)	0.9853(7)	0.4760(2)	0.0349(14)
C39	0.33263(15)	0.0397(7)	0.4738(2)	0.0426(15)
C40	0.33436(18)	0.1643(8)	0.5072(3)	0.0524(17)
C41	0.36747(18)	0.2380(8)	0.5412(3)	0.0516(17)
C42	0.39935(17)	0.1899(7)	0.5423(2)	0.0463(16)
C43	0.39845(15)	0.0629(7)	0.5097(2)	0.0389(14)
N1	0.33341(11)	0.6340(5)	0.47390(16)	0.0320(11)
N2	0.30902(11)	0.4383(6)	0.40464(15)	0.0350(11)
N3	0.37129(11)	0.5382(5)	0.44176(15)	0.0303(11)
N4	0.43568(11)	0.5034(5)	0.50108(15)	0.0311(11)
N5	0.39823(10)	0.6655(5)	0.52331(15)	0.0272(10)
N6	0.36177(11)	0.6993(5)	0.56255(15)	0.0291(10)
O1	0.30384(11)	0.7622(6)	0.66256(16)	0.0645(13)
O2	0.25261(12)	0.6186(7)	0.60850(16)	0.0704(14)
O3	0.17858(12)	0.2007(8)	0.3248(2)	0.099(2)
O4	0.17626(13)	0.2601(8)	0.3936(2)	0.0932(19)
O5	0.31734(12)	0.8694(7)	0.30184(19)	0.0835(17)
O6	0.25989(14)	0.9502(6)	0.2696(2)	0.0818(16)
O7	0.47453(10)	0.0452(5)	0.40122(15)	0.0487(11)
O8	0.52146(11)	0.1223(5)	0.47772(16)	0.0507(11)
O9	0.56145(11)	0.3425(5)	0.60529(16)	0.0539(11)
O10	0.56649(12)	0.4199(7)	0.67935(19)	0.0862(17)
O11	0.40182(11)	0.8244(7)	0.73490(16)	0.0803(16)
O12	0.46172(12)	0.7695(7)	0.76567(16)	0.0740(15)
O13	0.36444(9)	0.8484(5)	0.44643(13)	0.0365(9)

	x/a	y/b	z/c	U(eq)
Cl1	0.44907(8)	0.2227(4)	0.67949(11)	0.1008(10)
C44	0.4807(2)	0.229(2)	0.7429(6)	0.190(10)

Table S4. Bond lengths (Å) for **7a**.

B1-O13	1.442(7)	B1-N3	1.493(7)
B1-N5	1.497(7)	B1-N1	1.506(7)
C1-N6	1.352(6)	C1-N1	1.356(6)
C1-C2	1.450(7)	C2-C17	1.389(7)
C2-C13	1.450(7)	C4-N2	1.350(6)
C4-N1	1.361(6)	C4-C17	1.455(7)
C5-N2	1.346(6)	C5-N3	1.367(6)
C5-C6	1.449(7)	C6-C7	1.396(7)
C6-C22	1.478(8)	C7-C8	1.446(7)
C7-C26	1.454(7)	C8-N3	1.356(6)
C8-N4	1.356(6)	C9-N4	1.346(6)
C9-N5	1.353(6)	C9-C10	1.460(7)
C10-C11	1.397(7)	C10-C30	1.421(7)
C11-C34	1.430(7)	C11-C12	1.470(7)
C12-N6	1.339(6)	C12-N5	1.355(6)
C13-C14	1.314(7)	C13-H13	0.95
C14-C15	1.461(8)	C14-H14	0.95
C15-O2	1.194(7)	C15-O1	1.345(7)
C16-O1	1.441(7)	C16-H16A	0.98
C16-H16B	0.98	C16-H16C	0.98
C17-C18	1.445(7)	C18-C19	1.318(7)
C18-H18	0.95	C19-C20	1.483(8)
C19-H19	0.95	C20-O4	1.160(7)
C20-O3	1.320(8)	C21-O3	1.469(8)
C21-H21A	0.98	C21-H21B	0.98
C21-H21C	0.98	C22-C23	1.274(7)
C22-H22	0.95	C23-C24	1.484(9)
C23-H23	0.95	C24-O6	1.163(7)
C24-O5	1.314(8)	C25-O5	1.465(9)
C25-H25A	0.98	C25-H25B	0.98
C25-H25C	0.98	C26-C27	1.314(7)
C26-H26	0.95	C27-C28	1.466(8)
C27-H27	0.95	C28-O8	1.192(6)
C28-O7	1.340(7)	C29-O7	1.448(6)
C29-H29A	0.98	C29-H29B	0.98
C29-H29C	0.98	C30-C31	1.334(7)
C30-H30	0.95	C31-C32	1.464(8)
C31-H31	0.95	C32-O10	1.198(7)
C32-O9	1.330(7)	C33-O9	1.437(7)
C33-H33A	0.98	C33-H33B	0.98
C33-H33C	0.98	C34-C35	1.329(7)
C34-H34	0.95	C35-C36	1.463(8)
C35-H35	0.95	C36-O12	1.189(7)
C36-O11	1.338(7)	C37-O11	1.430(7)
C37-H37A	0.98	C37-H37B	0.98
C37-H37C	0.98	C38-C39	1.378(7)

C38-O13	1.390(6)	C38-C43	1.392(7)
C39-C40	1.379(8)	C39-H39	0.95
C40-C41	1.368(9)	C40-H40	0.95
C41-C42	1.368(8)	C41-H41	0.95
C42-C43	1.386(8)	C42-H42	0.95
C43-H43	0.95	Cl1-C44	1.696(14)
C44-C44	1.447(18)	C44-H44A	0.99
C44-H44B	0.99		

Table S5. Bond angles (°) for **7a**.

O13-B1-N3	113.3(4)	O13-B1-N5	115.0(5)
N3-B1-N5	104.0(4)	O13-B1-N1	116.2(4)
N3-B1-N1	103.6(4)	N5-B1-N1	103.3(4)
N6-C1-N1	123.0(4)	N6-C1-C2	129.4(5)
N1-C1-C2	106.3(4)	C17-C2-C1	107.1(4)
C17-C2-C13	127.8(5)	C1-C2-C13	125.0(5)
N2-C4-N1	121.9(5)	N2-C4-C17	130.8(5)
N1-C4-C17	105.8(4)	N2-C5-N3	123.0(5)
N2-C5-C6	129.8(5)	N3-C5-C6	105.5(4)
C7-C6-C5	107.9(5)	C7-C6-C22	123.8(5)
C5-C6-C22	127.7(5)	C6-C7-C8	106.5(4)
C6-C7-C26	127.4(5)	C8-C7-C26	126.1(5)
N3-C8-N4	122.7(4)	N3-C8-C7	106.8(4)
N4-C8-C7	128.8(5)	N4-C9-N5	121.8(4)
N4-C9-C10	130.2(5)	N5-C9-C10	106.3(4)
C11-C10-C30	126.8(5)	C11-C10-C9	106.9(4)
C30-C10-C9	126.2(4)	C10-C11-C34	127.5(5)
C10-C11-C12	107.2(4)	C34-C11-C12	125.3(4)
N6-C12-N5	123.2(5)	N6-C12-C11	129.9(4)
N5-C12-C11	105.5(4)	C14-C13-C2	125.3(5)
C14-C13-H13	117.4	C2-C13-H13	117.3
C13-C14-C15	121.3(5)	C13-C14-H14	119.4
C15-C14-H14	119.4	O2-C15-O1	123.0(5)
O2-C15-C14	126.3(6)	O1-C15-C14	110.7(5)
O1-C16-H16A	109.5	O1-C16-H16B	109.5
H16A-C16-H16B	109.5	O1-C16-H16C	109.5
H16A-C16-H16C	109.5	H16B-C16-H16C	109.5
C2-C17-C18	125.6(5)	C2-C17-C4	107.4(4)
C18-C17-C4	126.9(5)	C19-C18-C17	127.9(5)
C19-C18-H18	116.0	C17-C18-H18	116.0
C18-C19-C20	119.0(6)	C18-C19-H19	120.5
C20-C19-H19	120.5	O4-C20-O3	123.3(6)
O4-C20-C19	124.9(7)	O3-C20-C19	111.8(6)
O3-C21-H21A	109.5	O3-C21-H21B	109.5
H21A-C21-H21B	109.5	O3-C21-H21C	109.5
H21A-C21-H21C	109.5	H21B-C21-H21C	109.5
C23-C22-C6	128.9(6)	C23-C22-H22	115.6
C6-C22-H22	115.6	C22-C23-C24	123.3(6)
C22-C23-H23	118.3	C24-C23-H23	118.3
O6-C24-O5	123.0(6)	O6-C24-C23	124.0(7)
O5-C24-C23	113.0(6)	O5-C25-H25A	109.5
O5-C25-H25B	109.5	H25A-C25-H25B	109.5
O5-C25-H25C	109.5	H25A-C25-H25C	109.5
H25B-C25-H25C	109.5	C27-C26-C7	126.2(5)
C27-C26-H26	116.9	C7-C26-H26	116.9
C26-C27-C28	124.3(5)	C26-C27-H27	117.9
C28-C27-H27	117.9	O8-C28-O7	123.8(5)
O8-C28-C27	123.7(6)	O7-C28-C27	112.5(5)
O7-C29-H29A	109.5	O7-C29-H29B	109.5
H29A-C29-H29B	109.5	O7-C29-H29C	109.5
H29A-C29-H29C	109.5	H29B-C29-H29C	109.5

C31-C30-C10	124.6(5)	C31-C30-H30	117.7
C10-C30-H30	117.7	C30-C31-C32	122.5(5)
C30-C31-H31	118.8	C32-C31-H31	118.8
O10-C32-O9	123.4(5)	O10-C32-C31	125.8(6)
O9-C32-C31	110.7(5)	O9-C33-H33A	109.5
O9-C33-H33B	109.5	H33A-C33-H33B	109.5
O9-C33-H33C	109.5	H33A-C33-H33C	109.5
H33B-C33-H33C	109.5	C35-C34-C11	124.6(5)
C35-C34-H34	117.7	C11-C34-H34	117.7
C34-C35-C36	122.6(5)	C34-C35-H35	118.7
C36-C35-H35	118.7	O12-C36-O11	123.5(6)
O12-C36-C35	126.6(6)	O11-C36-C35	109.8(5)
O11-C37-H37A	109.5	O11-C37-H37B	109.5
H37A-C37-H37B	109.5	O11-C37-H37C	109.5
H37A-C37-H37C	109.5	H37B-C37-H37C	109.5
C39-C38-O13	120.7(5)	C39-C38-C43	120.1(5)
O13-C38-C43	119.1(5)	C38-C39-C40	119.4(6)
C38-C39-H39	120.3	C40-C39-H39	120.3
C41-C40-C39	120.7(6)	C41-C40-H40	119.7
C39-C40-H40	119.7	C40-C41-C42	120.2(6)
C40-C41-H41	119.9	C42-C41-H41	119.9
C41-C42-C43	120.2(6)	C41-C42-H42	119.9
C43-C42-H42	119.9	C42-C43-C38	119.2(5)
C42-C43-H43	120.4	C38-C43-H43	120.4
C1-N1-C4	111.9(4)	C1-N1-B1	122.6(4)
C4-N1-B1	124.1(4)	C5-N2-C4	116.8(4)
C8-N3-C5	111.6(4)	C8-N3-B1	122.9(4)
C5-N3-B1	123.3(4)	C9-N4-C8	117.0(4)
C9-N5-C12	112.7(4)	C9-N5-B1	123.7(4)
C12-N5-B1	122.8(4)	C12-N6-C1	116.1(4)
C15-O1-C16	115.5(5)	C20-O3-C21	113.9(6)
C24-O5-C25	114.3(5)	C28-O7-C29	114.4(4)
C32-O9-C33	117.0(5)	C36-O11-C37	116.2(5)
C38-O13-B1	113.0(4)	C44-C44-Cl1	116.8(16)
C44-C44-H44A	108.1	Cl1-C44-H44A	108.1
C44-C44-H44B	108.1	Cl1-C44-H44B	108.1
H44A-C44-H44B	107.3		

Table S6. Torsion angles (°) for **7a.**

N6-C1-C2-C17	-158.8(5)	N1-C1-C2-C17	7.9(6)
N6-C1-C2-C13	17.3(9)	N1-C1-C2-C13	-176.1(5)
N2-C5-C6-C7	-157.4(5)	N3-C5-C6-C7	7.7(6)
N2-C5-C6-C22	13.7(9)	N3-C5-C6-C22	178.8(5)
C5-C6-C7-C8	0.0(6)	C22-C6-C7-C8	-171.6(5)
C5-C6-C7-C26	178.7(5)	C22-C6-C7-C26	7.2(8)
C6-C7-C8-N3	-7.8(5)	C26-C7-C8-N3	173.4(5)
C6-C7-C8-N4	157.6(5)	C26-C7-C8-N4	-21.1(8)
N4-C9-C10-C11	-159.7(5)	N5-C9-C10-C11	5.7(5)
N4-C9-C10-C30	16.1(8)	N5-C9-C10-C30	-178.6(5)
C30-C10-C11-C34	2.9(9)	C9-C10-C11-C34	178.7(5)
C30-C10-C11-C12	-174.2(5)	C9-C10-C11-C12	1.6(5)
C10-C11-C12-N6	158.2(5)	C34-C11-C12-N6	-18.9(9)
C10-C11-C12-N5	-8.2(5)	C34-C11-C12-N5	174.6(5)
C17-C2-C13-C14	-178.4(5)	C1-C2-C13-C14	6.4(9)
C2-C13-C14-C15	-178.1(5)	C13-C14-C15-O2	-4.9(10)
C13-C14-C15-O1	175.7(5)	C1-C2-C17-C18	176.6(5)
C13-C2-C17-C18	0.7(9)	C1-C2-C17-C4	-0.7(6)
C13-C2-C17-C4	-176.7(5)	N2-C4-C17-C2	159.0(5)
N1-C4-C17-C2	-6.6(6)	N2-C4-C17-C18	-18.3(9)
N1-C4-C17-C18	176.1(5)	C2-C17-C18-C19	-177.7(6)
C4-C17-C18-C19	-0.9(10)	C17-C18-C19-C20	-176.6(6)
C18-C19-C20-O4	-6.3(11)	C18-C19-C20-O3	174.3(6)
C7-C6-C22-C23	-178.7(6)	C5-C6-C22-C23	11.5(10)
C6-C22-C23-C24	-178.7(6)	C22-C23-C24-O6	-179.1(7)
C22-C23-C24-O5	2.4(9)	C6-C7-C26-C27	-170.2(6)
C8-C7-C26-C27	8.3(9)	C7-C26-C27-C28	-176.1(5)
C26-C27-C28-O8	-173.9(6)	C26-C27-C28-O7	8.1(8)
C11-C10-C30-C31	175.9(5)	C9-C10-C30-C31	0.9(9)
C10-C30-C31-C32	-175.8(5)	C30-C31-C32-O10	-2.5(10)
C30-C31-C32-O9	175.1(5)	C10-C11-C34-C35	-177.3(6)
C12-C11-C34-C35	-0.7(9)	C11-C34-C35-C36	-178.5(6)
C34-C35-C36-O12	3.4(12)	C34-C35-C36-O11	-176.7(6)
O13-C38-C39-C40	173.5(5)	C43-C38-C39-C40	-3.7(8)
C38-C39-C40-C41	2.1(9)	C39-C40-C41-C42	0.6(9)
C40-C41-C42-C43	-1.7(9)	C41-C42-C43-C38	0.0(8)
C39-C38-C43-C42	2.7(8)	O13-C38-C43-C42	-174.6(5)
N6-C1-N1-C4	155.0(5)	C2-C1-N1-C4	-12.7(6)
N6-C1-N1-B1	-12.1(8)	C2-C1-N1-B1	-179.8(4)
N2-C4-N1-C1	-155.0(5)	C17-C4-N1-C1	12.2(6)
N2-C4-N1-B1	11.9(8)	C17-C4-N1-B1	179.1(4)
O13-B1-N1-C1	-97.0(6)	N3-B1-N1-C1	138.0(5)
N5-B1-N1-C1	29.8(6)	O13-B1-N1-C4	97.4(6)
N3-B1-N1-C4	-27.6(6)	N5-B1-N1-C4	-135.8(5)
N3-C5-N2-C4	-10.4(7)	C6-C5-N2-C4	152.4(5)
N1-C4-N2-C5	9.5(7)	C17-C4-N2-C5	-154.1(5)
N4-C8-N3-C5	-153.2(5)	C7-C8-N3-C5	13.3(5)
N4-C8-N3-B1	10.4(7)	C7-C8-N3-B1	176.9(4)
N2-C5-N3-C8	153.2(5)	C6-C5-N3-C8	-13.1(5)
N2-C5-N3-B1	-10.3(8)	C6-C5-N3-B1	-176.7(5)
O13-B1-N3-C8	98.1(6)	N5-B1-N3-C8	-27.4(6)

N1-B1-N3-C8	-135.1(5)	O13-B1-N3-C5	-100.2(6)
N5-B1-N3-C5	134.3(5)	N1-B1-N3-C5	26.6(6)
N5-C9-N4-C8	-7.9(7)	C10-C9-N4-C8	155.5(5)
N3-C8-N4-C9	9.9(7)	C7-C8-N4-C9	-153.4(5)
N4-C9-N5-C12	155.3(5)	C10-C9-N5-C12	-11.6(6)
N4-C9-N5-B1	-14.6(7)	C10-C9-N5-B1	178.5(4)
N6-C12-N5-C9	-155.1(5)	C11-C12-N5-C9	12.5(6)
N6-C12-N5-B1	14.9(8)	C11-C12-N5-B1	-177.6(5)
O13-B1-N5-C9	-94.7(6)	N3-B1-N5-C9	29.7(6)
N1-B1-N5-C9	137.7(5)	O13-B1-N5-C12	96.4(6)
N3-B1-N5-C12	-139.1(5)	N1-B1-N5-C12	-31.1(6)
N5-C12-N6-C1	8.6(7)	C11-C12-N6-C1	-155.8(5)
N1-C1-N6-C12	-9.8(7)	C2-C1-N6-C12	154.9(5)
O2-C15-O1-C16	-0.3(10)	C14-C15-O1-C16	179.1(6)
O4-C20-O3-C21	2.3(12)	C19-C20-O3-C21	-178.2(7)
O6-C24-O5-C25	-2.7(11)	C23-C24-O5-C25	175.8(7)
O8-C28-O7-C29	-2.6(8)	C27-C28-O7-C29	175.4(5)
O10-C32-O9-C33	0.7(9)	C31-C32-O9-C33	-177.1(5)
O12-C36-O11-C37	0.2(11)	C35-C36-O11-C37	-179.8(7)
C39-C38-O13-B1	-84.0(6)	C43-C38-O13-B1	93.3(5)
N3-B1-O13-C38	-172.3(4)	N5-B1-O13-C38	-52.9(6)
N1-B1-O13-C38	67.9(6)		

$-2\pi^2[h^2 a^{*2} U_{11} + ... + 2 h k a^* b^* U_{12}]$

	**U_{11}**	**U_{22}**	**U_{33}**	**U_{23}**	**U_{13}**	**U_{12}**
B1	0.039(4)	0.027(4)	0.031(4)	0.002(3)	0.020(3)	0.003(3)
C1	0.037(3)	0.029(3)	0.030(3)	-0.001(3)	0.018(3)	0.007(3)
C2	0.022(3)	0.034(3)	0.035(3)	0.002(3)	0.015(2)	0.003(2)
C4	0.027(3)	0.039(4)	0.030(3)	0.001(3)	0.010(3)	0.000(3)
C5	0.029(3)	0.038(3)	0.029(3)	-0.003(3)	0.014(3)	-0.003(3)
C6	0.041(3)	0.038(4)	0.028(3)	-0.004(3)	0.019(3)	-0.006(3)
C7	0.037(3)	0.027(3)	0.033(3)	-0.003(3)	0.020(3)	-0.007(3)
C8	0.034(3)	0.033(3)	0.031(3)	0.001(3)	0.022(3)	0.001(3)
C9	0.022(3)	0.027(3)	0.038(3)	-0.001(3)	0.017(3)	-0.005(2)
C10	0.031(3)	0.029(3)	0.033(3)	-0.002(2)	0.019(3)	-0.006(2)
C11	0.031(3)	0.028(3)	0.033(3)	-0.006(3)	0.017(3)	-0.005(2)
C12	0.039(3)	0.026(3)	0.029(3)	0.000(2)	0.018(3)	0.000(2)
C13	0.023(3)	0.043(4)	0.040(4)	0.003(3)	0.018(3)	0.005(3)
C14	0.033(3)	0.054(4)	0.039(4)	-0.003(3)	0.024(3)	0.001(3)
C15	0.033(4)	0.066(5)	0.042(4)	0.000(3)	0.021(3)	0.006(3)
C16	0.097(6)	0.173(9)	0.055(5)	-0.032(5)	0.054(5)	-0.025(6)
C17	0.027(3)	0.037(4)	0.031(3)	0.002(3)	0.013(3)	0.003(3)
C18	0.030(3)	0.049(4)	0.035(4)	0.000(3)	0.014(3)	0.005(3)
C19	0.035(3)	0.080(5)	0.046(4)	-0.015(4)	0.022(3)	-0.006(3)
C20	0.034(4)	0.080(5)	0.063(5)	-0.016(4)	0.023(4)	-0.009(4)
C21	0.059(5)	0.176(10)	0.101(7)	-0.061(7)	0.012(5)	-0.053(6)
C22	0.041(4)	0.059(4)	0.034(3)	-0.005(3)	0.022(3)	-0.010(3)
C23	0.043(4)	0.060(5)	0.052(4)	-0.006(4)	0.021(3)	0.005(3)
C24	0.047(4)	0.056(5)	0.059(5)	-0.012(4)	0.028(4)	-0.013(4)
C25	0.069(5)	0.112(8)	0.139(8)	-0.082(7)	0.022(5)	0.002(5)
C26	0.043(4)	0.036(4)	0.038(3)	-0.004(3)	0.028(3)	-0.004(3)

	U_{11}	U_{22}	U_{33}	U_{23}	U_{13}	U_{12}
C27	0.041(4)	0.040(4)	0.042(4)	-0.007(3)	0.022(3)	0.003(3)
C28	0.046(4)	0.039(4)	0.042(4)	-0.002(3)	0.026(3)	-0.005(3)
C29	0.059(4)	0.058(5)	0.088(5)	-0.015(4)	0.052(4)	0.004(4)
C30	0.027(3)	0.044(4)	0.033(3)	0.002(3)	0.016(3)	0.000(3)
C31	0.028(3)	0.042(4)	0.042(3)	0.005(3)	0.019(3)	0.000(3)
C32	0.040(4)	0.055(4)	0.048(4)	0.004(3)	0.024(3)	0.003(3)
C33	0.054(4)	0.076(5)	0.105(6)	0.037(4)	0.055(4)	0.032(4)
C34	0.027(3)	0.044(4)	0.031(3)	-0.005(3)	0.014(3)	0.000(3)
C35	0.037(3)	0.076(5)	0.028(3)	-0.008(3)	0.012(3)	0.003(3)
C36	0.035(4)	0.092(6)	0.045(4)	-0.008(4)	0.021(3)	-0.001(4)
C37	0.089(6)	0.206(11)	0.057(5)	-0.038(6)	0.051(5)	0.003(6)
C38	0.035(3)	0.035(4)	0.039(3)	0.012(3)	0.021(3)	0.003(3)
C39	0.037(3)	0.039(4)	0.047(4)	0.006(3)	0.017(3)	0.004(3)
C40	0.055(4)	0.042(4)	0.072(5)	0.009(4)	0.040(4)	0.015(3)
C41	0.059(5)	0.030(4)	0.070(5)	-0.003(3)	0.036(4)	0.000(3)
C42	0.057(4)	0.034(4)	0.058(4)	-0.003(3)	0.036(3)	-0.014(3)
C43	0.034(3)	0.044(4)	0.049(4)	0.009(3)	0.028(3)	0.000(3)
N1	0.026(2)	0.038(3)	0.034(3)	-0.002(2)	0.017(2)	-0.001(2)
N2	0.032(3)	0.043(3)	0.031(3)	-0.002(2)	0.017(2)	-0.003(2)
N3	0.027(2)	0.036(3)	0.031(3)	-0.001(2)	0.016(2)	0.000(2)
N4	0.032(2)	0.036(3)	0.029(3)	-0.005(2)	0.018(2)	-0.006(2)
N5	0.028(3)	0.026(3)	0.031(3)	-0.001(2)	0.017(2)	-0.003(2)
N6	0.026(3)	0.031(3)	0.034(3)	0.000(2)	0.017(2)	0.002(2)
O1	0.054(3)	0.104(4)	0.045(3)	-0.018(3)	0.032(2)	-0.010(3)
O2	0.047(3)	0.115(4)	0.061(3)	-0.007(3)	0.036(2)	-0.011(3)
O3	0.045(3)	0.160(6)	0.072(4)	-0.059(4)	0.016(3)	-0.035(3)
O4	0.054(3)	0.148(5)	0.084(4)	-0.041(4)	0.040(3)	-0.044(3)
O5	0.045(3)	0.103(4)	0.086(4)	-0.057(3)	0.020(3)	-0.013(3)
O6	0.057(3)	0.074(4)	0.093(4)	-0.023(3)	0.022(3)	-0.009(3)
O7	0.044(2)	0.054(3)	0.055(3)	-0.013(2)	0.031(2)	-0.001(2)
O8	0.039(3)	0.054(3)	0.058(3)	-0.002(2)	0.023(2)	0.003(2)
O9	0.049(3)	0.055(3)	0.072(3)	0.011(2)	0.041(2)	0.018(2)
O10	0.051(3)	0.146(5)	0.047(3)	0.006(3)	0.013(2)	0.040(3)
O11	0.043(3)	0.157(5)	0.043(3)	-0.027(3)	0.023(2)	0.004(3)
O12	0.047(3)	0.135(5)	0.038(3)	-0.003(3)	0.020(2)	0.001(3)
O13	0.042(2)	0.036(2)	0.036(2)	0.0026(19)	0.0226(18)	0.0034(18)
Cl1	0.107(2)	0.084(2)	0.0722(19)	0.0031(16)	0.0161(17)	0.0024(17)
C44	0.203(19)	0.206(17)	0.072(10)	0.014(11)	0.004(13)	-0.073(13)

Table S8. Hydrogen atomic coordinates and isotropic atomic displacement parameters (Å^2) for **7a.**

	x/a	y/b	z/c	U(eq)
H13	0.2590	0.5454	0.5312	0.041
H14	0.3200	0.7447	0.5955	0.047
H16A	0.2684	0.8428	0.6840	0.152
H16B	0.3098	0.8180	0.7306	0.152
H16C	0.2841	0.6545	0.7038	0.152
H18	0.2318	0.4232	0.4524	0.047
H19	0.2416	0.3303	0.3715	0.063
H21A	0.1418	0.0319	0.3242	0.187
H21B	0.1325	0.0891	0.2682	0.187

	x/a	y/b	z/c	U(eq)
H21C	0.1243	0.2167	0.3028	0.187
H22	0.3570	0.0755	0.3568	0.052
H23	0.2893	0.2129	0.3285	0.064
H25A	0.2857	-0.3432	0.2812	0.18
H25B	0.3240	-0.3686	0.2821	0.18
H25C	0.2908	-0.2653	0.2362	0.18
H26	0.4175	0.1413	0.3922	0.043
H27	0.4705	0.2997	0.4807	0.049
H29A	0.5193	0.0380	0.3933	0.092
H29B	0.4883	-0.0998	0.3588	0.092
H29C	0.5140	-0.1306	0.4190	0.092
H30	0.5036	0.5617	0.6565	0.041
H31	0.5006	0.4752	0.5651	0.044
H33A	0.6163	0.3528	0.6502	0.107
H33B	0.6025	0.2061	0.6075	0.107
H33C	0.5982	0.1811	0.6568	0.107
H34	0.4672	0.6747	0.6869	0.041
H35	0.3938	0.7685	0.6516	0.058
H37A	0.4192	0.7563	0.8066	0.166
H37B	0.3863	0.8938	0.7844	0.166
H37C	0.4275	0.9521	0.8004	0.166
H39	0.3094	0.9916	0.4494	0.051
H40	0.3123	1.1994	0.5065	0.063
H41	0.3683	1.3230	0.5643	0.062
H42	0.4222	1.2436	0.5654	0.056
H43	0.4206	1.0292	0.5103	0.047
H44A	0.4759	0.3333	0.7574	0.228
H44B	0.4763	0.1293	0.7593	0.228

Table S9. Hydrogen bond distances (Å) and angles (°) for **7a.**

	Donor-H	Acceptor-H	Donor-Acceptor	Angle
C43-H43···O8	0.95	2.55	3.483(7)	166.8
C39-H39···O2	0.95	2.44	3.386(7)	174.5
C35-H35···N6	0.95	2.39	3.079(7)	129.0
C33-H33B···O13	0.98	2.65	3.570(8)	156.7
C31-H31···N4	0.95	2.42	3.093(7)	127.5
C27-H27···N4	0.95	2.44	3.120(7)	128.8
C25-H25A···O6	0.98	2.38	3.052(10)	125.6
C23-H23···N2	0.95	2.68	3.278(8)	121.6
C19-H19···N2	0.95	2.61	3.227(7)	123.3
C16-H16C···O6	0.98	2.49	3.226(10)	132.0
C14-H14···N6	0.95	2.43	3.082(6)	125.2

12. References

1 F. Berrée, A. M. Debache, Y. Marsac, B. Collet, P. Girard-Le Bleiz and B. Carboni, *Tetrahedron*, 2006, **62**, 4027–4037.

2 M. V. Chelliah, S. Chackalamannil, Y. Xia, K. Eagen, M. C. Clasby, X. Gao, W. Greenlee, H. S. Ahn, J. Agans-Fantuzzi, G. Boykow, Y. Hsieh, M. Bryant, J. Palamanda, T. M. Chan, D. Hesk and M. Chintala, *J. Med. Chem.*, 2007, **50**, 5147–5160.

3 E. Caballero, D. Guzmán, T. Torres and M. S. Rodríguez-Morgade, *J. Org. Chem.*, 2020, **85**, 1948–1960.

4 S. J. Lange, H. Nie, C. L. Stern, A. G. M. Barrett and B. M. Hoffman, *Inorg. Chem.*, 1998, **37**, 6435–6443.

5 M. S. Rodríguez-Morgade, C. G. Claessens, A. Medina, D. González-Rodríguez, E. Gutiérrez-Puebla, A. Monge, I. Alkorta, J. Elguero and T. Torres, *Chem. Eur. J.*, 2008, **14**, 1342–1350.

6 M. S. Rodríguez-Morgade, T. Torres, C. Atienza-Castellanos and D. M. Guldi, *J. Am. Chem. Soc.*, 2006, **128**, 15145–15154.

7 M. S. Rodríguez-Morgade, M. E. Plonska-Brzezinska, A. J. Athans, E. Carbonell, G. De Miguel, D. M. Guldi, L. Echegoyen and T. Torres, *J. Am. Chem. Soc.*, 2009, **131**, 10484–10496.

8 I. Solymosi, S. Krishna, E. Nuin, H. Maid, B. Scholz, D. M. Guldi, M. E. Pérez-Ojeda and A. Hirsch, *Chem. Sci.*, 2021, **12**, 15491–15502.

9 A. Weller, *Zeitschrift fur Phys. Chemie*, 1982, **133**, 93–98.

10 D. Veldman, S. M. A. Chopin, S. C. J. Meskers and R. A. J. Janssen, *J. Phys. Chem. A*, 2008, **112**, 8617–8632.

11 B. P. Karsten, R. K. M. Bouwer, J. C. Hummelen, R. M. Williams and R. A. J. Janssen, *Photochem. Photobiol. Sci.*, 2010, **9**, 1055–1065.

12 G. Zango, M. Krug, S. Krishna, V. Mariñas, T. Clark, M. V. Martinez-Diaz, D. M. Guldi and T. Torres, *Chem. Sci.*, 2020, **11**, 3448–3459.

13 K. M. Mullen and I. H. M. van Stokkum, *J. Stat. Softw.*, 2007, **18**, 1–46.

14 J. J. Snellenburg, S. Laptenok, R. Seger, K. M. Mullen and I. H. M. van Stokkum, *J. Stat. Softw.*, 2012, **49**, 1–22.

15 N. Medintz, I. L.; Hildebrandt, *FRET-Förster resonance energy transfer: from theory to applications.*, John Wiley & Sons, 2013.

16 C. Bannwarth, S. Ehlert and S. Grimme, *J. Chem. Theory Comput.*, 2019, **15**, 1652–1671.

17 F. Neese, *Wiley Interdiscip. Rev. Comput. Mol. Sci.*, 2022, **12**, 1–15.

18 P. J. Hay and W. R. Wadt, *J. Chem. Phys.*, 1985, **82**, 270–283.

19 M. M. Francl, W. J. Pietro, W. J. Hehre, J. S. Binkley, M. S. Gordon, D. J. Defrees, J. A. Pople, J. S. Binkley and M. S. Gordon, *J. Chem. Phys.*, 1982, **77**, 3654–3665.

20 A. D. Becke, *J. Chem. Phys.*, 1993, **98**, 5648–5652.

21 V. A. Rassolov, M. A. Ratner, J. A. Pople, P. C. Redfern and L. A. Curtiss, *J. Comput. Chem.*, 2001, **22**, 976–984.

22 S. Grimme, S. Ehrlich and L. Goerigk, *J. Comput. Chem.*, 2011, **32**, 1456–1465.

23 Gaussian 16, Revision A.03, M. J. Frisch, G. W. Trucks, H. B. Schlegel, G. E. Scuseria, M. A. Robb, J. R. Cheeseman, G. Scalmani, V. Barone, G. A. Petersson, H. Nakatsuji, X. Li, M. Caricato, A. V. Marenich, J. Bloino, B. G. Janesko, R. Gomperts, B. Mennucci, H. P. Hratchian, J. V. Ortiz, A. F. Izmaylov, J. L. Sonnenberg, D. Williams-Young, F. Ding, F. Lipparini, F. Egidi, J. Goings, B. Peng, A. Petrone, T. Henderson, D. Ranasinghe, V. G. Zakrzewski, J. Gao, N. Rega, G. Zheng, W. Liang, M. Hada, M. Ehara, K. Toyota, R. Fukuda, J. Hasegawa, M. Ishida, T. Nakajima, Y. Honda, O. Kitao, H. Nakai, T. Vreven, K. Throssell, J. A. Montgomery, Jr., J. E. Peralta, F. Ogliaro, M. J. Bearpark, J. J. Heyd, E. N. Brothers, K. N. Kudin, V. N. Staroverov, T. A. Keith, R. Kobayashi, J. Normand, K. Raghavachari, A. P. Rendell, J. C. Burant, S. S. Iyengar, J. Tomasi, M. Cossi, J. M. Millam, M. Klene, C. Adamo, R. Cammi, J. W. Ochterski, R. L. Martin, K. Morokuma, O. Farkas, J. B. Foresman, and D. J. Fox, Gaussian, Inc., Wallingford CT, 2016.

24 M. D. Hanwell, D. E. Curtis, D. C. Lonie, T. Vandermeersch, E. Zurek and G. R. Hutchison, *J. Cheminform.*, 2011, **4**, 17.
